# A unique mechanism involving cocatalysis of enzyme and nonenzyme to form β-carboline and spirotryprostatins in *Aspergillus fumigatus*

**DOI:** 10.1126/sciadv.adz2319

**Published:** 2025-10-10

**Authors:** Hai Gao, Deng Yu, Qiao Luo, Yanan Wang, Ling Lu, Weiming Zhu, Yi Wang

**Affiliations:** ^1^ School of Medicine and Pharmacy, Ocean University of China, Qingdao 266003, China.; ^2^ Laboratory for Marine Drugs and Bioproducts, Qingdao Marine Science and Technology Centre, Qingdao 266237, China.; ^3^ Key Laboratory of Marine Drugs, Ministry of Education of China, Ocean University of China, Qingdao 266003, China.

## Abstract

Environmental stress represents an effective strategy for discovering natural products from microorganisms, but the processes involved remain unclear. Under acidic conditions, a previously unidentified class of β-carbolines (βCs), termed secofumitremorgins (SFs), with potent antiangiogenic activity in zebrafish models was found and identified via nuclear magnetic resonance, Marfey’s analysis, and quantum calculations. Using gene knockout, heterologous expression, precursor feeding, isotope tracing, molecular docking, and site-directed mutagenesis, a unique biosynthetic pathway for SFs was characterized, beyond the Pictet-Spenglerase–mediated biosynthetic process. Furthermore, an unreported pH-regulated mechanism was unveiled, where P450 (FtmG) facilitated new fumitremorgin intermediate formation, enabling spontaneous, nonenzymatic generation of βCs and spirotryprostatins under distinct pH conditions. Notably, this work provides detailed experimental evidence and reaction mechanisms for synergistic enzymatic and nonenzymatic catalysis driving metabolite diversification under pH stress. The findings expand biosynthetic paradigms for indole alkaloids and highlight the therapeutic potential of pH-elicited βCs, advancing strategies for natural product discovery.

## Teaser

Low pH unveils antiangiogenic β-carbolines via unique enzyme-nonenzymatic biosynthesis, advancing natural product discovery.

## INTRODUCTION

Indole alkaloids form a substantial subclass of natural products, garnering attention due to their wide-ranging biological activities and extensive structural diversity (*1*, *2*). Among these, β-carbolines (βCs) and spirotryprostatins (STs) are particularly noteworthy. βCs have been reported to have a variety of bioactivities, including antibacterial, antifungal, antiviral, anti-inflammatory, and antitumor effects (*3*–*7*). Similarly, STs have demonstrated antibacterial and antitumor properties (*8*–*10*). Their diverse biological activities underscore their great potential in drug development. Therefore, exploring their biosynthetic pathways is of exceptional importance for advancing our understanding and utilization of these compounds.

The biosynthetic pathways of naturally occurring βCs have been scarcely documented. To date, only three enzymes involved in the formation of the βC skeleton have been identified, namely, STR, McbB, and KslB, all of which function as Pictet-Spenglerases in terms of function. STR was the first enzyme found to be capable of forming βCs, catalyzing the condensation of tryptamine and monoterpene aldehyde to produce these compounds (*11*–*17*). McbB facilitates a Pictet-Spengler reaction between l-tryptophan and sodium acetate, subsequently forming βCs through decarboxylation and oxidation (*18*). Similarly, KslB catalyzes the reaction between l-tryptophan and α-ketoglutaric acid, leading to βC production via decarboxylation and oxidation (*19*, *20*). On the basis of previous research, the sole known biosynthetic route for the naturally sourced βC skeleton involves Pictet-Spenglerases catalyzing reactions from tryptophan and its analogs. In the realm of natural product biosynthesis, other potential pathways for βC production have yet to be discovered, highlighting an area ripe for further investigation.

Reports on the biosynthetic pathway of STs are similarly limited. Now, only two enzymes involved in the formation of the helical skeleton of STs have been identified. One such enzyme is FqzB, which has been confirmed to catalyze the epoxidation of demethoxyfumitremorgin C (**11**), leading to the formation of the spiro carbon in spirotryprostatin A (**17**) through a semipinacol-type rearrangement (*21*) (fig. S1A). The other enzyme, FtmG, is involved in catalyzing the formation of spirotryprostatin G (**3**) from fumitremorgin C (**1**) and spirotryprostatin B (**13**) from demethoxyfumitremorgin C (**11**), respectively (*21*) (fig. S1B). During the precursor feeding experiment using **11** as the substrate, Tsunematsu *et al.* observed the generation of 7a-hydroxy demethoxyfumitremorgin C (**15**). From this observation, a hypothesis was proposed: FtmG oxidizes C-7a to form an intermediate monohydroxy fumitremorgins (Sh-FTs), prompting a radical migration from C-8 to C-1a. Hydroxylation at C-1a could then induce a semipinacol-type rearrangement, yielding dihydrogen STs (Db-STs) with double bonds at C-7a and C-8 positions (*21*). Although compound **15** was speculated to be a potential intermediate in this process, the hypothesis remains tentative due to a lack of experimental evidence to confirm it (fig. S1B).

**Fig. 1.**
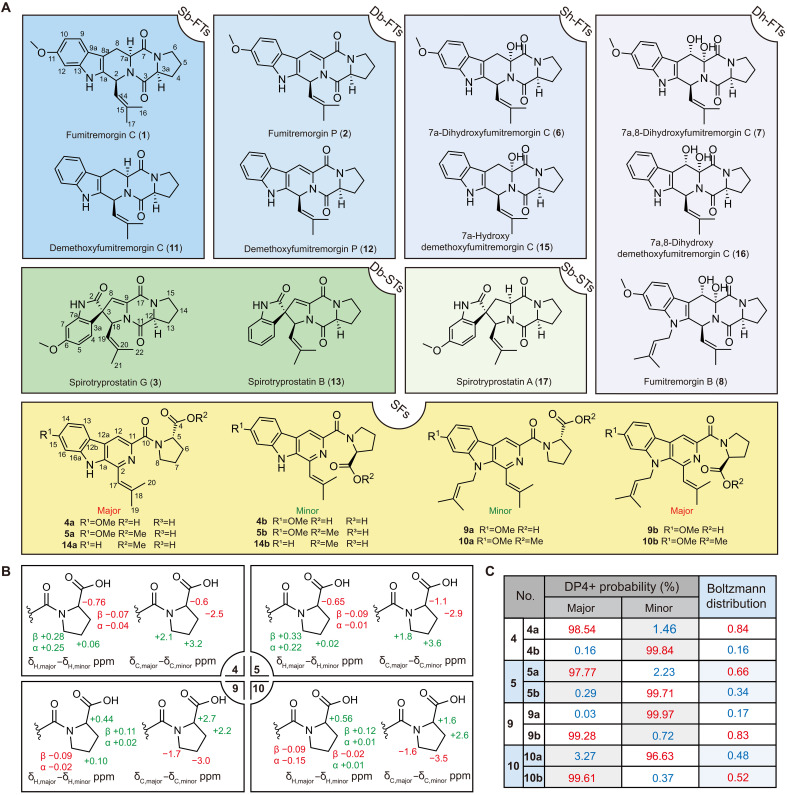
Overview chart of the compounds isolated in this study, together with the elucidation of the absolute configurations of SFs. (**A**) Among them, **14a/14b** were speculated on the basis of molecular weight. The color blue was used to represent FTs, with the shades changing from dark to light, representing Sb-FTs (**1** and **11**), Db-FTs (**2** and **12**), Sh-FTs (**6** and **15**), and Dh-FTs (**7**, **8** and **16**), respectively; green was used to represent SFs, with the shades changing from dark to light, representing Db-STs (**3** and **13**) and Sb-FTs (**17**), respectively; yellow was used to represent SFs (**4a/4b**, **5a/5b**, **9a/9b**, **10a/10b**, and **14a/14b**). (**B**) Δδ (δ_major_ − δ_minor_) values of the ^13^C and ^1^H NMR chemical shifts at C-5, C-6, C-7, and C-8 for compounds **4a/4b**, **5a/5b**, **9a/9b**, and **10a/10b**. (**C**) ^13^C NMR calculations coupled with DP4+ probability analysis and Gibbs free energy calculations coupled with Boltzmann distribution analysis for compounds **4a/4b**, **9a/9b**, and **10a/10b**.

To address the limited understanding of the biosynthetic pathway of βCs and the unclear role of FtmG in the biosynthetic pathway of STs, we undertook comprehensive research in this study. Under low pH stress, from *Aspergillus fumigatus* OUCMDZ-5210 (GenBank number MK393941.1), we successfully isolated a novel class of βC compounds termed secofumitremorgins (SFs) (Fig. 1A). These compounds were characterized by unique proline side chains linked via amide bonds and two distinct isomers resulting from the partial rotational restriction of the amide bond. The SFs include secofumitremorgin A/B (**5a/5b**, **5**) (*22*), as well as new compounds such as secofumitremorgin C/D (**4a/4b**, **4**), secofumitremorgin E/F (**9a/9b**, **9**), and secofumitremorgin G/H (**10a/10b**, **10**). In addition, FTs featuring double bonds at the C-7a and C-8 positions (Db-FTs) were isolated from natural sources, namely, fumitremorgin P (**2**) and demethoxyfumitremorgin P (**12**) (Fig. 1A). Using a combination of gene knockout, heterologous expression, precursor feeding, isotope tracing, and pH regulation experiments, we revealed that FtmG catalyzes the formation of two key intermediates, Db-FTs and FTs with double hydroxy groups at C-7a and C-8 positions (Dh-FTs). These intermediates are derived from STs with single bonds at C-7a and C-8 positions (Sb-FTs). Furthermore, we found that, under different pH conditions, these intermediates spontaneously and selectively form Db-STs or SFs. To better understand the catalytic mechanism of FtmG, molecular docking and site-directed mutagenesis were used, leading to the identification of two key residues, Arg^292^ and Cys^442^, critical for its function. In addition, bioassay results indicated that the ring opening of FTs may attenuate their cytotoxicity while imparting significant inhibitory activity against erythropoiesis and blood vessel growth. This finding sheds light on the biosynthetic complexities and potential biological applications of these compounds.

## RESULTS

### Discovery of new βCs (SFs)

When exploring the secondary metabolites of *A. fumigatus* OUCMDZ-5210, derived from mangrove sediment and cultivated under the acidic conditions to simulate the natural ecological environment, we isolated a unique class of βC compounds (Fig. 1A). From the acidic fermentation extract, we identified compounds **4a/4b** (figs. S14 to S21 and table S4), **5a/5b** (figs. S22 to S28 and table S5), **9a/9b** (figs. S34 to S41 and table S9), and **10a/10b** (figs. S42 to S48 and table S10). Among these, all the compounds were isolated (Fig. 1A), except for **5a/5b**, which were previously reported in 2022 (*22*). The tandem mass spectrometry (MS/MS)–based molecular networking (fig. S2) also indicated that OUCMDZ-5210 can produce more derivatives under low pH.

These compounds had a β-carboline ring as their nucleus, with an isobutenyl group substituted at the α-position and a carbonyl side chain at the γ-position, which connects to the nitrogen atom of proline. The absolute configuration of proline was determined to be l-proline by Marfey’s method (fig. S63). A notable structural feature was the presence of p-π conjugation between the nitrogen atom of the l-proline side chain and its adjacent amidocarbonyl group. This conjugation restricted free rotation of the C-N bond, resulting in two distinct orientations of the carboxyl group. These compounds existed as mixture in a ratio of 1 (major):0.7 (minor), which could not be separated using various chromatographic purification methods. The major and minor diastereomers of the SFs were principally distinguished by differences in the ^1^H and ^13^C nuclear magnetic resonance (NMR) chemical shifts of the proline residue. Specifically, in **4a/4b** and **5a/5b,** the major diastereomers displayed notably larger δ_C/H_ values at C-4 and C-5 and smaller δ_C/H_ values at C-6 and C-7 relative to their minor counterparts (Fig. 1B and tables S4 and S5); conversely, in **9a/9b** and **10a/10b,** the pattern of δ_C/H_ differences at C-4, C-5, C-6, and C-7 was essentially the inverse of that observed for **4a/4b** and **5a/5b** (Fig. 1B and tables S9 and S10). These suggested that N-1 isopentenylation directs divergent configurational preferences among the SFs. To assign the major and minor configurations, we calculated their δ_C_ at the B3LYP/6-31G(d) level and performed DP4+ probability analysis (Fig. 1C and tables S26 to S33). The results assigned the major configurations of **4a/4b** and **5a/5b** to **4a** and **5a** (with **4b** and **5b** as the minor), whereas for **9a/9b** and **10a/10b**, the major configurations were **9b** and **10b** (with **9a** and **10a** as the minor), in full agreement with our hypothesis. Moreover, Gibbs free energies were computed under standard conditions to derive Boltzmann distributions (Fig. 1C and table S22), which further validated these assignments. The final configurational designations of the major and minor SFs were depicted in Fig. 1A.

Naturally occurring βCs reported in the literature were derived from Pictet-Spenglerase reactions. However, whole-genome sequencing and sequence alignment analysis of *A. fumigatus* OUCMDZ-5210 did not reveal any sequences with high homology to known Pictet-Spenglerase enzymes. This led us to hypothesize that either an undiscovered Pictet-Spenglerase enzyme exists or that a novel biosynthetic mechanism distinct from the conventional pathway is responsible for the production of these βC compounds. Although this observation is intriguing, a lack of experimental evidence prevented us from conducting further investigation into this unique biosynthetic process.

### Discovery of Db-FTs and gene knockout analysis

Fortunately, by using the OSMAC (one strain many compounds) strategy, we successfully obtained two key pathway metabolites, Db-FTs, namely, compounds **2** (figs. S5 to S11 and table S2) and **12** (figs. S51 and S52 and table S12), from a neutral fermentation environment (fig. S61), distinct from the previously used acidic pH conditions. These metabolites were isolated as natural products (Fig. 1A). Given the extremely high chemical structural similarity between Db-FTs and SFs, we proposed a bold hypothesis that Db-FTs may undergo an oxidation reaction to form an aromatic ring, followed by hydrolysis that opens the amide bond at the C-3 position, thereby forming SFs. In addition, the double bond at the C-7a and C-8 positions in Db-FTs is likely formed by the oxidation of Sb-FTs. Thus, Db-FTs serve as a crucial intermediate connecting these two hypothetical pathways, providing valuable insights into the biosynthetic processes involved.

**Fig. 2.**
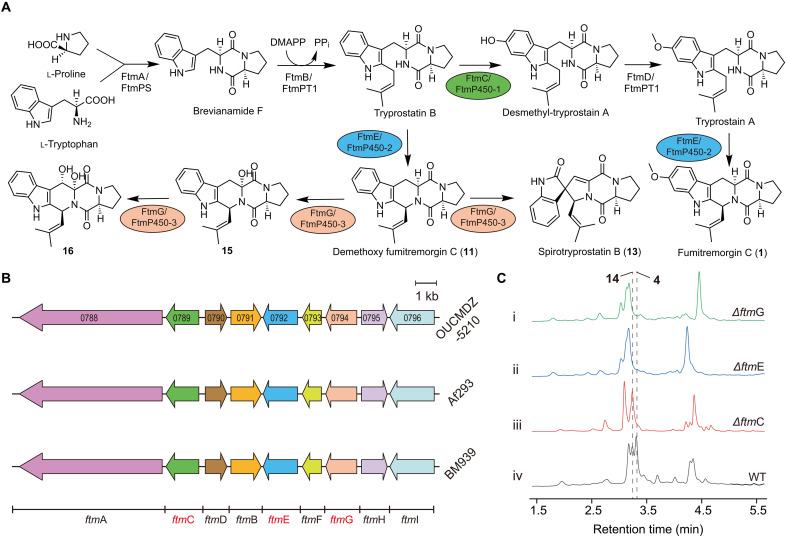
Gene knockout and bioinformatics analysis. (**A**) Currently reported biosynthetic pathways of indole diketopiperazine compounds and the enzymes involved. PP_i_, inorganic pyrophosphate. DMAPP, dimethylallyl pyrophosphate. (**B**) The genetic sequence similarity of FTM gene clusters of OUCMDZ-5210, Af293, and BM939 strains was compared, and the gene marked in red represented the cytochrome P450 gene. (**C**) Comparison of UPLC of metabolites of OUCMDZ-5210 wild-type (WT) strain and knockout strains at a wavelength of 280 nm. (i) was the *ftm*G gene knockout strain; (ii) was the *ftm*E gene knockout strain; (iii) was the *ftm*C gene knockout strain; (iv) was the wild-type strain.

Because Db-FTs are derivatives of FTs, we hypothesize that the genes responsible for the formation of SFs are likely to be located within the biosynthetic gene cluster involved in the synthesis of FTs and their downstream modifications gene cluster (FTM) (Fig. 2B). To investigate this, we performed multiple sequence alignments on the genes contained within the FTM gene cluster of OUCMDZ-5210 and compared them with those from *A. fumigatus* Af293 and *A. fumigatus* BM939, both of which have been previously reported to produce FTs. The results revealed that the amino acid sequences of nine proteins were highly similar, with over 99% sequence identity (Fig. 2B). This high level of similarity ruled out the possibility that the production of SFs is due to undiscovered genes outside of this cluster. Consequently, we propose that some of the already reported genes within the FTM gene cluster may have yet-to-be-unknown functions, which could potentially contribute to the biosynthesis of SFs. This hypothesis opens avenues for further exploration of the functional diversity within the FTM gene cluster.

P450 enzymes are considered among the most catalytically versatile biocatalysts in nature, and multiple P450 proteins have been implicated in the reported FT biosynthetic pathway (Fig. 2A), In this pathway, three genes encoding P450 enzymes were identified and designated as 0789 (*ftm*C), 0792 (*ftm*E), and 0794 (*ftm*G) (*21*) (Fig. 2B). To investigate their roles, these three genes were individually knocked out using the polyethylene glycol (PEG)–mediated protoplast transformation method. The results showed that the Δ*ftm*E and Δ*ftm*G strains were unable to produce SFs, whereas the Δ*ftm*C strain retained the ability to produce SFs (Fig. 2C). This finding indicates that the *ftm*E and *ftm*G genes are involved in the biosynthesis of SFs. In the previously reported FT biosynthetic pathway, *ftm*E was identified as being responsible for the formation of the piperidine ring within the FT core structure, and *ftm*G was responsible for the modifications to the FT nucleus (*21*). On the basis of this, we speculated that *ftm*G plays a crucial role in the conversion of FTs to SFs, likely mediating the key transformation steps required for SF biosynthesis. This highlights the potential functional significance of *ftm*G in the pathway.

### Sb-FTs precursor feeding analysis

**Fig. 3.**
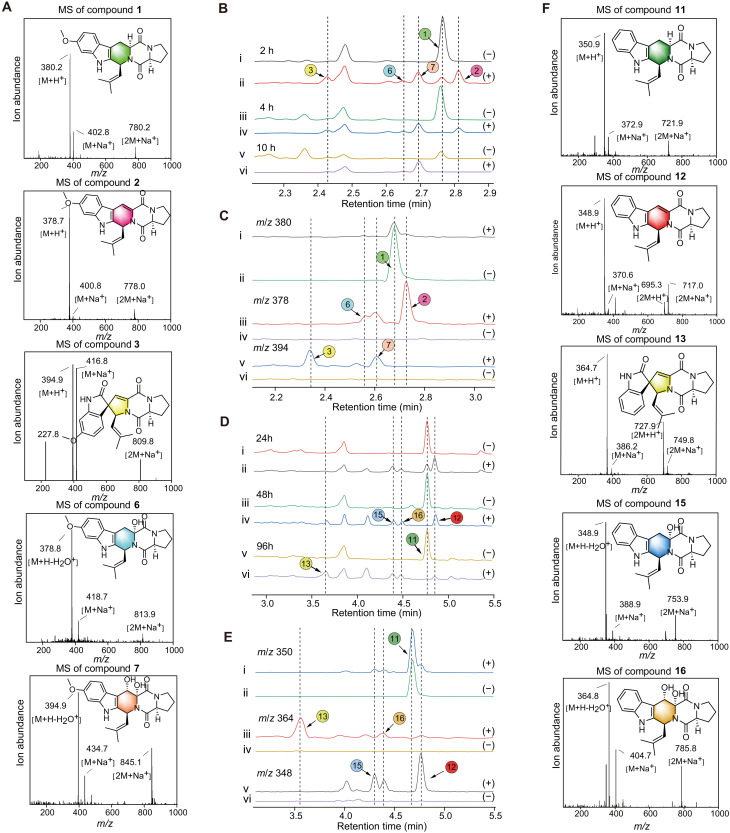
Precursor feeding experiment of Sb-FTs in *P. pastoris* expressing FtmG. (**A**) MS spectra and structural formulas of **1**, **2**, **3**, **6**, and **7**. (**B**) Precursor feeding experiment with **1** as the substrate. LC traces of metabolites at 2 hours (i and ii), 4 hours (iii and iv), and 10 hours (v and vi), monitored at 280 nm. h, hours. (**C**) Precursor LC traces extracted from the above figure at 2 hours corresponding to **1** (*m/z* = 380) (i and ii), **2**, **6**, and **7** (*m/z* = 378) (iii and iv), and **3**, **6**, and **7** (*m/z* = 394) (v and vi). (**D**) Precursor feeding experiment with **11** as the substrate. LC traces of metabolites at 24 hours (i and ii), 48 hours (iii and iv), and 96 hours (v and vi), monitored at 280 nm. (**E**) LC traces extracted from the above figure at 24 hours corresponding to **11** (*m/z* = 350) (i and ii), **13**, **15**, and **16** (*m/z* = 364) (iii and iv), and **12**, **15**, and **16** (*m/z* = 348) (v and vi). (−) represented the yeast containing the blank plasmid; (+) represented the recombinant expression yeast expressing FtmG. (**F**) MS spectra and structural formulas of **11**, **12**, **13**, **15**, and **16**.

To validate the above speculation, the *ftm*G gene was heterologously expressed in *Pichia pastoris* GS115, and feeding experiments were carried out. The intronless *ftm*G gene was cloned into the expression plasmid pPIC9K-His, and the recombinant plasmid was electroporated into *P. pastoris* GS115 for heterologous expression. The recombinant strain was cultured in 10 ml of Buffered Methanol-complex Medium (BMMY) supplemented with methanol to induce protein expression for 24 hours. Subsequently, 1.5 μM compound **1** was added to the culture. Ethyl acetate extracts of the culture broth were analyzed by liquid chromatography–mass spectrometry (LC-MS) at 2-, 4-, and 10-hour intervals. The results showed that, within 2 hours, the concentration of **1** notably decreased, whereas compound **3**, 7a-hydroxyfumitremorgin C (**6**), and 7a,8-dihydroxyfumitremorgin C (**7**) were detected. By 4 hours, compound **1** completely disappeared, compound **6** began to decline, and **7** showed a slight increase. After 10 hours, **6** was no longer detectable, and **7** levels further increased. Ultimately, only compounds **3** and **7** remained. These observations are consistent with a previous report (*21*). During the incubation process, we also observed the production and subsequent disappearance of **2**, a process that had not been reported before (Fig. 3, A to C). Similarly, when **11** was used as the substrate in the feeding experiment, analogous results were observed for compound **12**, which behaved similarly to **2** (Fig. 3, D to F). These findings suggest that Sb-FTs can be oxidized to Db-FTs under the catalytic action of FtmG, with Db-FTs potentially acting as key intermediates in the biosynthesis pathway. Although the formation and disappearance of Db-FTs were monitored during this process, the production of SFs was not observed. This raised an intriguing question about the fate of the disappeared Db-FTs, sparking curiosity about their subsequent transformations and their role in the overall biosynthetic pathway. These findings point to previously unknown intermediate steps that warrant further investigation to unravel the complete pathway leading to SFs.

### Discussion on the formation mechanism of Db-FTs

**Fig. 4.**
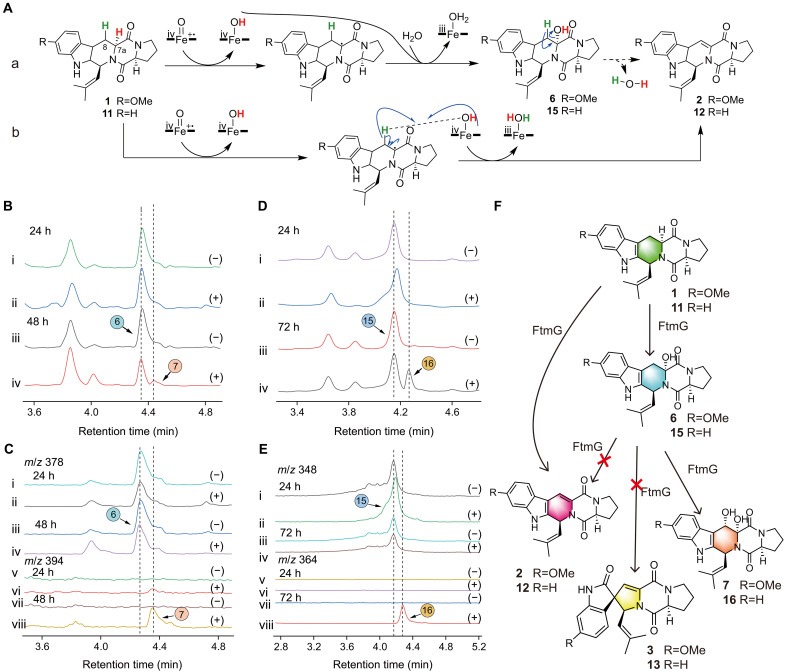
Precursor feeding experiment of Sh-FTs in *P. pastoris* expressing FtmG. (**A**) Two speculated mechanisms for the formation of Db-FTs from Sb-FTs, (a) and (b). (**B**) Precursor feeding experiment with **6** as the substrate. LC traces of products at 24 hours (i and ii) and 48 hours (iii and iv), monitored at 280 nm. (**C**) LC traces extracted from the above figure corresponding to **6** (*m/z* = 378) (i, ii, iii, and iv) and **7** (*m/z* = 394) (v, vi, vii, and viii). (**D**) Precursor feeding experiment with **15** as the substrate. LC traces of products at 24 hours (i and ii) and 72 hours (iii and iv), monitored at 280 nm. (**E**) LC traces extracted from the above figure corresponding to **15** (*m/z* = 348) (i, ii, iii, and iv) and **16** (*m/z* = 364) (v, vi, vii, and viii). (−) represented the yeast containing the blank plasmid; (+) represented the recombinant expression yeast expressing FtmG. (**F**) Hypotheses and judgments on three products generated by Sb-FTs through Sh-FTs.

Two hypotheses can be proposed for the mechanism of Sb-FT conversion to Db-FTs catalyzed by P450/FtmG. Initially, the superoxide molecule on the tetravalent iron of heme attacked and abstracted the hydrogen to form a radical at the C-7a position. Subsequently, there were two situations: Route a was that the substrate was oxidized to a single hydroxyl group at the 7a position by FtmG and then dehydrated to form a double bond; route b was that the substrate was extracted by FtmG to form a radical by extracting one molecule of hydrogen and then underwent another round of synergistic reaction of extracting hydrogen to form a double bond (Fig. 4A).

To verify route a, **6** as the substrate was added to the yeast expressing FtmG for incubation. The experimental results showed that there was no obvious difference at the 24th hour; at the 48th hour, a small amount of **7** was produced, but no **2** was observed (Fig. 4, B and C). Similar results were obtained with **15** as the substrate (Fig. 4, D and E). These experimental results indicated that Sh-FTs can be oxidized to Dh-FTs under the catalysis of FtmG, but the dehydration of Sh-FTs to form Db-FTs was impossible. On the basis of these results, we excluded route a and considered route b to be more reasonable. An intriguing phenomenon was observed that Sh-FTs cannot be converted to Db-STs under the catalysis of FtmG, which proved that the hypothesis of Tsunematsu *et al.* (*21*) was incorrect (Fig. 4F).

### Db-FTs precursor feeding analysis

**Fig. 5.**
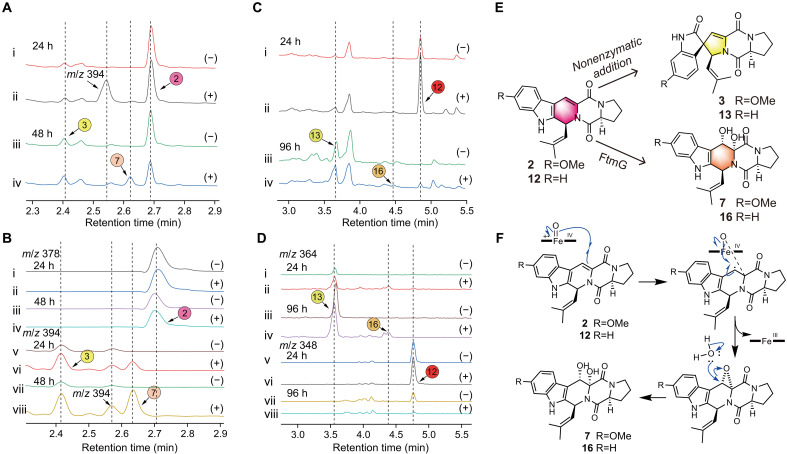
Precursor feeding experiment of Db-FTs and *P. pastoris* expressing FtmG. (**A**) Precursor feeding experiment with **2** as the substrate. LC traces of products at 24 hours (i and ii) and 48 hours (iii and iv), monitored at 280 nm. (**B**) LC traces extracted from the above figure corresponding to **2** (*m/z* = 378) (i, ii, iii, and iv) and **3** and **7** (*m/z* = 394) (v, vi, vii, and viii). (**C**) Precursor feeding experiment with **12** as the substrate. LC traces of products at 24 hours (i and ii) and 96 hours (iii and iv), monitored at 280 nm. (**D**) LC traces extracted from the above figure corresponding to **13** and **16** (*m/z* = 364) (i, ii, iii, and iv) and **12** (*m/z* = 348) (v, vi, vii, and viii). (−) represented the yeast containing the blank plasmid; (+) represented the recombinant expression yeast expressing FtmG. (**E**) Inference of enzymatic and nonenzymatic reaction products of Db-FTs. (**F**) Mechanism of the speculated formation of Dh-FTs from Db-FTs.

To further explore the reactions involving Db-FTs in the biosynthetic process, Db-FTs were incubated with host yeast expressing FtmG. The experimental results revealed that, when **2** was used as the substrate, a distinct compound with a mass/charge ratio (*m/z*) of 394 appeared at the 24-hour mark, compared to the blank control. By the 48th hour, this compound had largely disappeared, and **7** was produced. On the basis of its chemical structure and *m/z*, this differential compound is likely the epoxidation product at the C-7a and C-8a positions. Moreover, the production of **3** was detected in both the test and control groups (Fig. 5, A and B). Similar findings were observed when **12** was used as the substrate, although the peak with *m/z* 394 was not detected (Fig. 5, C and D). These results suggest that Db-FTs act as common precursors for both Db-STs and Dh-FTs. On the basis of these observations, a unidentified mechanism is proposed, involving the epoxidation of a double bond by FtmG followed by hydrolysis to form dihydroxy group (Fig. 5F). The generation of Db-STs (**3** and **13**) was detected in both the test and control groups across the experiments, indicating that the spirocyclization of Db-FTs to form Db-STs is a nonenzymatic process (Fig. 5E). This nonenzymatic pathway has not been previously reported. These findings challenge the pathway for Db-ST generation proposed by Tsunematsu *et al.*, which suggested that the spiro skeleton was formed by FtmG catalysis via the Sh-FT intermediate (*21*). The discovery of this nonenzymatic process opens up intriguing avenues for research into the biosynthetic pathways of these compounds. We are particularly interested in further exploring and understanding this unexpected nonenzymatic pathway.

### Nonenzymatic catalysis of Db-FTs

**Fig. 6.**
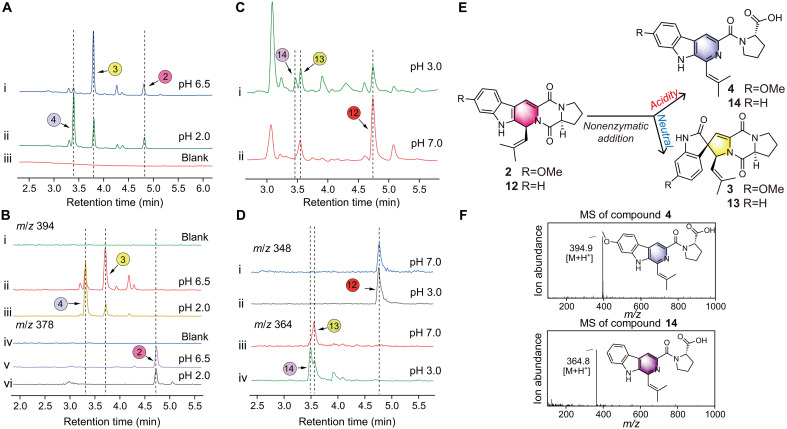
Nonenzymatic transformation experiment of Db-FTs. (**A**) Transformation experiment of **2** as the substrate in media with pH 6.5 (i) and 2.0 (ii) and LC trace of blank control (iii), monitored at 280 nm. (**B**) LC traces extracted from the above figure corresponding to **3** and **4** (*m/z* = 394) (i, ii, and iii) and **2** (*m/z* = 378) (iv, v, and vi). (**C**) LC traces of transformation experiment of **12** as the substrate in media with pH 3.0 (i) and 7.0 (ii), monitored at 280 nm. (**D**) LC traces extracted from the above figure corresponding to **12** (*m/z* = 348) (i and ii) and **13** and **14** (*m/z* = 364) (iii and iv). (**E**) Inference of nonenzymatic reaction products of Db-FTs at different pH levels. (**F**) MS spectra and structural formulas of **4** and **14**.

In the enzymatic experiments mentioned earlier, the production of SFs was not detected. Unexpectedly, it was observed that Db-FTs can undergo a nonenzymatic spirocyclization reaction. This led to the hypothesis that SFs might also be nonenzymatic catalytic products of Db-FTs. To investigate this, a simple nonenzymatic experiment was designed. Given that SFs were originally isolated from acidic culture conditions, we conducted nonenzymatic reactions with compound **2** as the substrate in culture medium at different pH levels. The results showed that, under both pH 6.5 and pH 2.0 conditions, **3** was produced, although the yield of **3** was lower under acidic conditions. At both pH levels, the formation of **4** was detected (Fig. 6, A, B, and F). Notably, the yield of **4** was higher under acidic conditions, which is in contrast to the higher yield of Db-SFs observed under neutral conditions. Similarly, when **12** was used as the substrate, analogous results were obtained (Fig. 6, C, D, and F). These findings suggest that SFs are also generated although the nonenzymatic catalysis of Db-FTs. The experiments imply that the ring opening of Db-FTs involves a nonenzymatic mechanism that includes proton participation (Fig. 6E). This finding adds a new dimension to our understanding of the biosynthetic processes, highlighting the role of nonenzymatic pathways in the formation of these compounds.

### Nonenzymatic catalytic mechanism of Db-FTs

The previously reported mechanism for the formation of STs from FTs involves epoxidation catalyzed by the oxidase FqzB, followed by spirocycle formation through a semipinacol rearrangement (*21*). On the basis of the previous hypothesis, we speculated that the aromatization of Db-FTs into SFs might also require an oxidizing agent. This led to the question of whether a specific component in the culture medium could provide the oxidizing conditions necessary for the production of SFs and Db-STs.

**Fig. 7.**
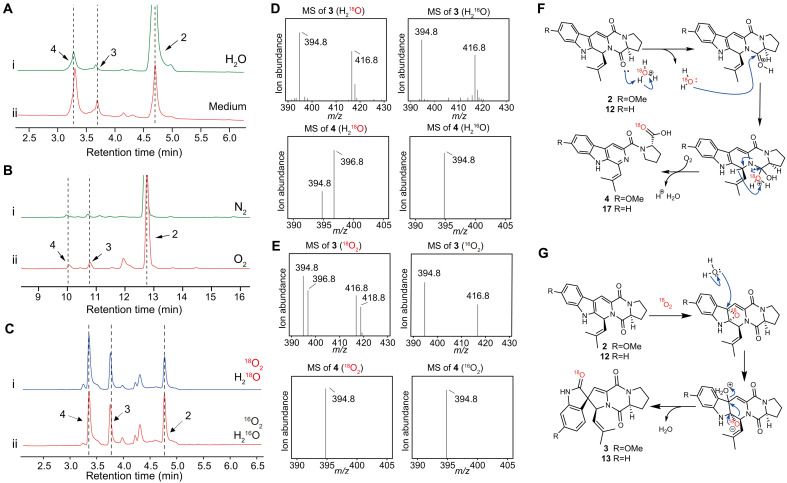
Isotope tracing experiment. (**A**) LC traces of nonenzymatic transformation of **2** as the substrate in water (i) and medium (ii) at pH 3.0. (**B**) LC traces of nonenzymatic reaction of **2** as the substrate under nitrogen (i) and oxygen (ii). (**C**) LC traces of nonenzymatic transformation of compound **2** under isotopic labeling with H_2_^18^O and ^18^O_2_. All were monitored at 280 nm. (**D**) MS spectrum of the nonenzymatic transformation of compound **2** under H_2_^18^O isotopic labeling. (**E**) MS spectrum of the nonenzymatic transformation of compound **2** under ^18^O_2_ isotopic labeling. (**F**) Speculation on the nonenzymatic mechanism of acidic oxidative ring opening of Db-FTs. (**G**) Speculation on the nonenzymatic mechanism of oxidative spirocyclization of Db-FTs.

To investigate this, the culture medium of the strain was used as the reaction environment, with water serving as the control group. The aim was to explore whether the culture medium could induce the spontaneous ring opening and spirocyclization of Db-FTs. Experimental results showed that both spirocyclic and ring-opened compounds were produced in the culture medium and in water, with a higher conversion rate observed in the culture medium (Fig. 7A). This indicates that the culture medium facilitated the transformation of Db-FTs but did not contain any special oxidizing component as initially hypothesized. After ruling out the presence of a special oxidizing agent in the culture medium, it was considered that atmospheric oxygen might be serving this role. To test this speculation, experiments were conducted by introducing nitrogen and oxygen into water at pH 3.0, using compound **2** as the substrate. The results showed that, in the oxygen-introduced group, **3** and **4** were clearly produced, whereas the nitrogen group exhibited little change (Fig. 7B). This confirmed that the spirocyclization and ring-opening processes of Db-FTs require the participation of oxygen, supporting the idea that atmospheric oxygen acts as the oxidizing agent.

On the basis of the chemical structure analysis, it was observed that both Db-ST and SF derivatives contain an additional oxygen atom compared to Db-FTs, with water and oxygen being the potential sources of this additional oxygen. To investigate the mechanisms of spirocyclization and ring opening, an isotopic labeling experiment was devised. Using **2** as the substrate, reactions were conducted under acidic conditions in both heavy oxygen water (H_2_^18^O) and heavy oxygen (^18^O_2_), respectively. In the H_2_^18^O labeling experiment, product **4** showed an *m/z* increase of 2 Da compared to the product formed in natural H_2_O, whereas product **3** exhibited no difference between the two conditions. In contrast, in the ^18^O_2_ labeling experiment, product **3** displayed an *m/z* that was 2 Da higher than that formed under ambient O_2_, whereas product **4** showed no mass shift (Fig. 7, C to E). These findings indicated that the ring-opening process fundamentally involved participation of H_2_O, whereas the additional oxygen atom in the spirocyclic products originated from O_2_. Therefore, a reaction mechanism was proposed in which Db-FTs undergo acid-catalyzed hydrolytic ring opening, followed by spontaneous oxidative aromatization by oxygen (Fig. 7F). In addition, an alternative mechanism was suggested, where Db-FTs undergo spontaneous epoxidation by oxygen, followed by hydrolysis and pinacol rearrangement to form the spirocyclic product (Fig. 7G). These proposed mechanisms offer a plausible explanation for experimental observations.

### Nonenzymatic catalysis of Dh-FTs

**Fig. 8.**
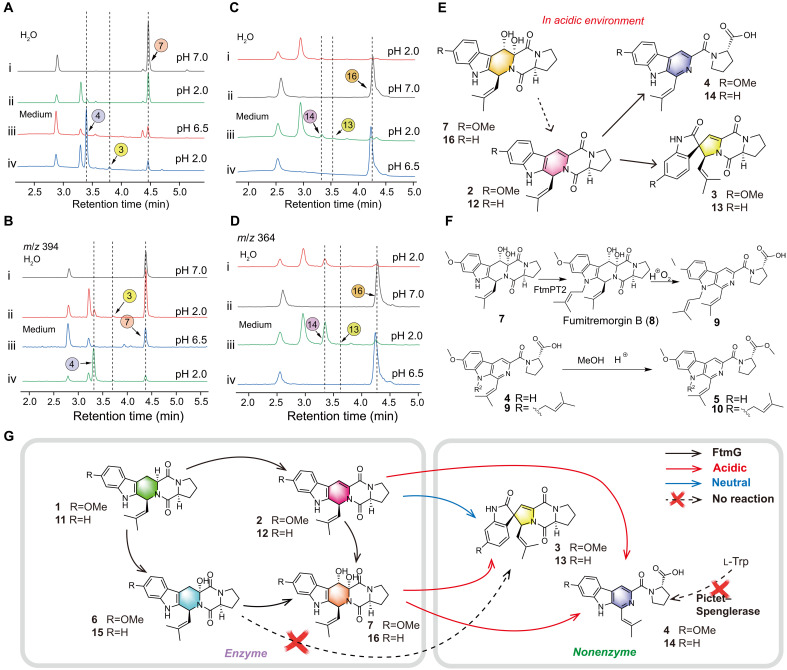
Nonenzymatic transformation of Dh-FTs and overview of the proposed biosynthetic pathways. (**A**) LC traces of transformation experiments with **7** as the substrate in water at pH 7.0 (i), water at pH 2.0 (ii), medium at pH 6.5 (iii), and medium at pH 2.0 (iv), monitored at 280 nm. (**B**) LC traces extracted from the above figure corresponding to **3**, **4**, and **7** (*m/z* = 394) (i, ii, iii, and iv). (**C**) LC traces of transformation experiments with **16** as the substrate in water at pH 7.0 (i), water at pH 2.0 (ii), medium at pH 7.0 (iii), and medium at pH 2.0 (iv), monitored at 280 nm. (**D**) LC traces extracted from the above figure corresponding to **13**, **14**, and **16** (*m/z* = 364) (i, ii, iii, and iv). (**E**) Inference of nonenzymatic reaction products of Dh-FTs under acidic conditions. (**F**) Speculation on the formation pathways of **5**, **9**, and **10**. (**G**) Biosynthetic scheme of FTs, STs, and SFs involving both enzymatic and nonenzymatic catalysis. The left panel represents enzyme-mediated reactions, whereas the right panel illustrates nonenzymatic transformations. Black arrows indicate FtmG-catalyzed steps; red arrows denote acid-promoted nonenzymatic transformations; blue arrows denote neutral nonenzymatic transformations; dashed arrows indicate reactions that do not occur.

Inspired by the nonenzymatic conversion of Db-FTs, we attempted to replicate fermentation environment using an acidic medium to explore the potential for nonenzymatic conversion of other FTs. When Dh-FTs were introduced as substrates into an acidic medium with a pH of 2.0, both SFs and Db-STs were produced. This represents a previously unreported nonenzymatic pathway. In our investigation of nonenzymatic reaction mechanisms, different media and pH levels were used as variables, with **7** serving as the substrate. The experiments demonstrated that Dh-FTs underwent transformation into both ring-opened and spirocyclic products only under acidic conditions, with the conversion rate of the ring-opened products exceeding that of the spirocyclic products (Fig. 8, A and B). Similarly, when 7a,8-dihydroxy fumitremorgin C (**16**) was used as the substrate, comparable results were observed (Fig. 8, C and D). The products obtained were consistent with those derived from Db-FTs under acidic conditions.

Therefore, it was hypothesized that Dh-FTs could transform into Db-FTs under acidic conditions, which would then undergo ring opening or spirocyclization (Fig. 8E). Although we attempted to identify intermediates using LC-MS, detecting Db-FTs proved to be challenging. This difficulty likely arose because the ring-opening and spirocyclization reactions occurred almost immediately after their formation. Given the presence of sequences in the genome of OUCMDZ-5210 with extremely high homology to the *N*-prenyltransferase FtmH/FtmPT2 (*23*), we speculated on the synthetic pathway of compound **9**. It was proposed that compound **7** is first catalyzed by FtmPT2 to form prenylated fumitremorgin B (**8**), which then spontaneously undergoes ring opening and aromatization to form compound **9** through a nonenzymatic pathway under acidic conditions. In addition, compounds **5** and **10** were identified as the carboxymethylated products of **4** and **9**, respectively. Because no enzymes within the FTM gene cluster have been reported to have carboxymethylation functionality, it is speculated that these compounds were produced through esterification reactions under acidic fermentation conditions or methanol extraction (Fig. 8F).

### The catalytic mechanism of FtmG

**Fig. 9.**
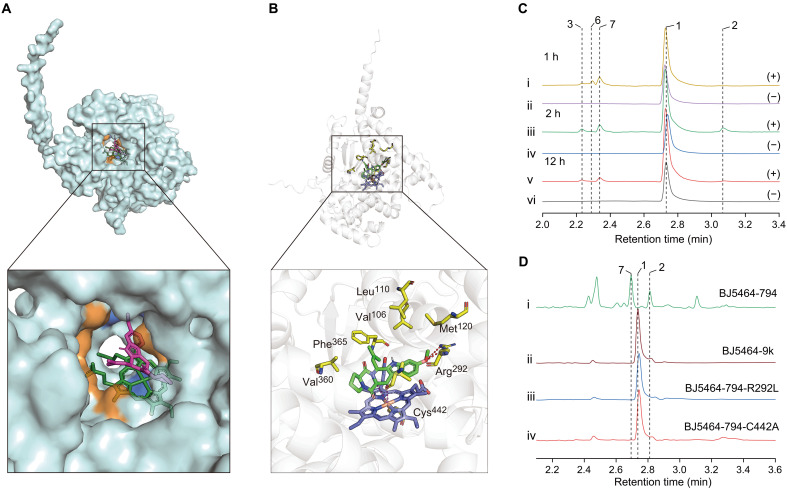
Molecular docking and site-directed mutagenesis experiment of FtmG. (**A**) Simulation docking results of **1** and FtmG protein and the enlarged surface diagram of the active pocket. Among them, orange represented amino acid residues with noncovalent bond forces; red was the ligand molecule; green was HEME. (**B**) Simulation docking results of **1** and FtmG protein and the enlarged cartoon diagram of the active pocket. Among them, yellow represented amino acid residues with noncovalent bond forces; green was the ligand molecule; blue was HEME. (**C**) Microsomal in vitro enzyme reaction experiment with **1** as the substrate. LC traces of products at 2 hours (i and ii), 4 hours (iii and iv), and 12 hours (v and vi), monitored at 280 nm. (−) represented the yeast containing the blank plasmid; (+) represented the recombinant expression yeast expressing FtmG. (**D**) HPLC traces of metabolites of the yeast with heterologous expression of FtmG protein (i), yeast containing the blank plasmid (ii), and amino acid at position 292 mutated from arginine to leucine (iii) and amino acid at position 442 mutated from cysteine to alanine (iv), monitored at 280 nm.

Microsomes from *P. pastoris* recombinantly expressing FtmG were extracted after 24 hours of induction culture with methanol for an in vitro enzymatic reaction assay. The addition of 1.5 μM **1** as a substrate produced results similar to those observed when **1** was incubated with yeast expressing FtmG (Fig. 9C). To investigate the function of FtmG protein and its catalytic mechanism, the Schrodinger 2021-3 software was used to simulate docking of the FtmG protein with compound **1** to understand their binding mechanism and identify key amino acid residues. As illustrated in Fig. 9, HEME was located in the center of the protein and ionically chelated with Cys^442^. Compound **1** was bound in the cavity above the HEME. The detailed interaction diagram revealed that the oxygen atom in the 6-OCH_3_ of **1** formed a hydrogen bond with Arg^292^ on the target protein. In addition, hydrophobic contacts were observed between **1** and the protein’s Leu^110^, Val^106^, Phe^365^, Val^360^, Arg^296^, and Met^120^ (Fig. 9, A and B). Hydrogen bonding is one of the strongest noncovalent interactions, and here, Arg^292^ formed two hydrogen bonds, making it a crucial amino acid for binding. Notably, the atoms at positions C-7 and C-8a of **1** were located directly above the HEME, with their distances to the iron atom measured at 3.7 and 3.8 Å, respectively. These relatively short distances indicated that these two atoms are highly susceptible to oxidation.

To further verify the docking results, point mutations were performed on the 292nd and 442nd amino acid residues of the FtmG protein. Experimental results showed that, when the amino acid at position 292 of FtmG was mutated from arginine to leucine, the protein lost the ability to convert **1** into **2**. Similarly, when the amino acid at position 442 was mutated from cysteine to alanine, FtmG also lost the ability to catalyze the conversion of **1** into **2** (Fig. 9D). On the basis of docking simulations and experimental data, it was concluded that arginine at position 292 and cysteine at position 442 are critical amino acid residues for the interaction between the FtmG protein and compound **1**, playing a key role in the catalytic mechanism.

### Biological activity testing

**Fig. 10.**
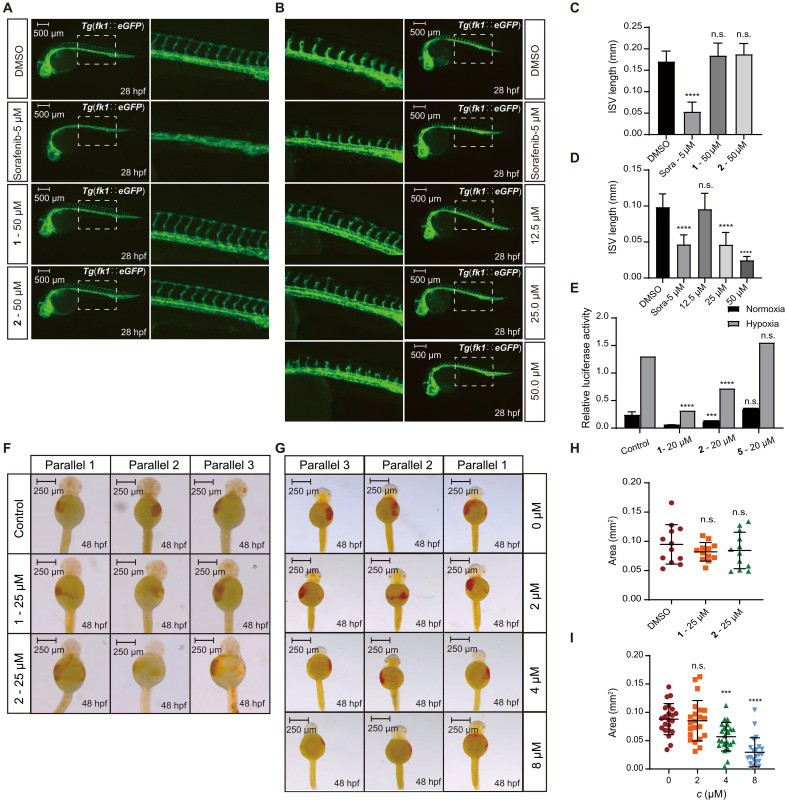
Effect of compounds 1, 2, and 5 on ISV angiogenesis and erythropoiesis in zebrafish embryos [*Tg*(*fk1∷eGFP*)] and on HIF-1α transcriptional activity in HCT-116. (**A**) Lateral views of zebrafish embryos with 50 μM **1** and **2** added at 24 hpf. (**B**) Lateral views of zebrafish embryos with 12.5 to 50 μM 5 added at 24 hpf. The blank control was DMSO, and the positive drug was 50 μM sorafenib. (**C**) Quantitative analysis of the effect of 50 μM **1** and **2** on zebrafish intersegmental vessel (ISV) angiogenesis. (**D**) Quantitative analysis of the effect of 12.5 to 50 μM **5** on zebrafish ISV angiogenesis. (**E**) Quantitative analysis of the effect of 20 μM **1**, **2**, and **5** on the transcriptional activity of HCT-116 under normoxic and hypoxic environments. (**F**) Dorsal views of zebrafish embryos with 0 to 8 μM compound **5** added at 48 hpf. (**G**) Dorsal views of zebrafish embryos with 25 μM compounds **1** and **2** added at 48 hpf. (**H**) Quantitative analysis of the effect of 0 to 8 μM **5** on the area of zebrafish red blood cells. (**I**) Quantitative analysis of the effect of 25 μM **1** and **2** on the area of zebrafish red blood cells. An analysis on the control group and the experimental group was conducted by using the two-sided one-sample *t* test. Error bars (means ± SD). n.s. (not significant) *P* > 0.05; ***P < 0.001; ****P < 0.0001.

Tumor cells consume a large amount of oxygen during rapid proliferation, often resulting in a hypoxic microenvironment within tumor tissues (*24*). Red blood cells are responsible for transporting oxygen (*25*), and blood vessels facilitate the transport of these red blood cells (*26*). Consequently, both can supply sufficient oxygen to tumor cells, thereby promoting their growth. In our study, we examined the effects of **1**, **2**, and **5** on angiogenesis and erythropoiesis. The results indicated that compounds **1** and **2** had effects on the red blood cells and blood vessels in zebrafish comparable to those observed in the control group, showing no significant activity (Fig. 10, A, C, F, and H). In contrast, compound **5** significantly damaged the blood vessels and red blood cells in zebrafish (Fig. 10, B, D, G, and I). This suggests that compound **5** effectively disrupts the normal growth of blood vessels and red blood cells, exhibiting inhibitory activity.

Hypoxia-inducible factor (HIF-1) is a nuclear protein with transcriptional activity found in human and mammalian cells. It includes oxygen-sensitive HIF-1α and continuously constitutively expressed HIF-1β, serving as a maker of hypoxia. HIF-1 is involved in biological effects such as angiogenesis, erythropoiesis, and tumor cell growth (*27*–*29*). It holds promising research potential in the development of noncytotoxic antitumor drugs. To determine whether the inhibitory activity of compound **5** on angiogenesis and erythrocyte growth was related to HIF-1α, we used a dual-luciferase reporter gene system to assess the effect of **1**, **2**, and **5** on the transcriptional activity of HIF-1α in HCT-116 cells. The results demonstrated that, compared with the control, the expression levels of HIF-1α for compounds **1** and **2** were significantly reduced, indicating HIF-1α transcriptional inhibitory activity. In contrast, the expression level of HIF-1α for compound **5** was not significantly different from that of the control group, suggesting that it had no effect on HIF-1α transcription (Fig. 10E). Therefore, the inhibitory effects of **5** on angiogenesis and erythrocyte growth were not related to HIF-1α. In addition, given that compound **1** has been reported to exhibit strong cytotoxicity (*30*, *31*), we tested the cytotoxic activity of **5** on 21 tumor cell lines, including HCT-116. The results showed that compound **5** displayed no significant cytotoxic activity (table S23).

From a structure-activity relationship perspective, compound **5** is the ring-opened product of **1**. The absence of cytotoxic activity in compound **5** may be attributable to the ring opening of the FT nucleus present in compound **1**. Because of its favorable properties, including strong anti-erythrocyte and antiangiogenic activities without cytotoxicity, compound **5** holds promise for development as a novel noncytotoxic antitumor drug.

## DISCUSSION

Our research reveals a unique mechanism involving the P450 enzyme FtmG (Fig. 8G), which demonstrates multiple catalytic properties in the first stage. It not only catalyzes the oxidative dehydrogenation of Sb-FTs to produce Db-FTs but also facilitates the dihydroxylation of Db-FTs to form Dh-FTs, which were not reported in early studies. During the second stage, SFs are spontaneously oxidized and undergo ring opening from Db-FTs or Dh-FTs in an acidic environment, rather than being produced by conventional PS enzymes. Furthermore, it is shown that Db-STs are spontaneously generated from Db-FTs under neutral conditions or from Dh-FTs under acidic conditions, contracting the previous assumption that they were catalyzed by FtmG from Sh-FTs (*21*) (Fig. 8G). The newly identified structural types of βCs displayed excellent potential for inhibiting angiogenic activity.

The biosynthetic pathways of SFs and Db-STs demonstrated that these two types of natural products can be directly generated from the intermediates Db-FTs or Dh-FTs by simply altering the pH level, without the involvement of any enzymes. Although similar hypotheses have been proposed previously, the generation of Db-FTs and SFs had not been documented in heterologous expression or precursor feeding experiments (*21*). This suggests that exploring pH regulation offers a promising approach for investigating the mechanism of biosynthesis. The cytochrome P450 enzyme FtmG catalyzes multistep reactions to generate different skeletons, indicating that this intriguing P450 oxidase may have additional yet undiscovered catalytic properties and mechanisms. This insight provides a more environmentally friendly and efficient method for synthesizing Db-FTs, which are crucial intermediates in chemical synthesis (*32*). As intermediates, Db-FTs and Dh-FTs are involved in forming various skeletons, highlighting their potential as synthons for constructing a range of βCs and STs. Our research exemplifies the diversity of natural product skeleton formation pathways and the possibility of catalytic mechanisms that involve both enzymatic and nonenzymatic processes.

## MATERIALS AND METHODS

### General

Electronic circular dichroism (ECD) spectra were determined using a JASCO J-815 spectropolarimeter. Ultraviolet (UV) spectra were collected from Waters 2487 detector. Infrared (IR) spectra were performed on a Bruker Tensor-27 spectrophotometer with KBr discs. A JASCO P-1020 digital polarimeter was used to obtain optical rotations. NMR spectra were carried out on an Agilent 500 MHz DD2 spectrometer and a Bruker AVANCE NEO 400 MHz with tetramethylsilane as an internal standard. High-resolution electrospray ionization–time-of-flight (ESI-TOF) mass spectra data were recorded using a Thermo Fisher Scientific LTQ Orbitrap XL mass spectrometer. Low-resolution LC/ESI-MS data were measured using a Waters ACQUITY SQD 2 UPLC/MS system with a reversed-phase C18 column (ACQUITY UPLC BEH C18, 2.1 mm by 50 mm, 1.7 μm). Semipreparative high-performance liquid chromatography (HPLC) was conducted with an ODS-C18 column (Waters, YMC-Pack ODS-A, 20 mm by 250 mm, 5 μm, 3 ml/min). Silica gel (200 to 300 or 100 to 200 mesh, Marine Chemical Factory of Qingdao) was used for vacuum-liquid chromatography (VLC). RP-18 silica gel (YMC ODS-A, 50 μm) and Sephadex LH-20 (Amersham Biosciences) were used for column chromatography (CC).

### Strains and culture conditions

Fungus OUCMDZ-5210 was isolated from a mangrove soil sample in Thailand in an acidic environment (pH 5.0), and the strain was identified as *A. fumigatus*. The gene sequence was deposited in GenBank with accession no. MK393941.1 (https://ncbi.nlm.nih.gov/nuccore/1556656878). The wild-type strain and transformants were maintained on potato dextrose agar (PDA) at 28°C. For the preparation of protoplasts, fresh mycelia of the strain were inoculated in 100 ml of sucrose-dextrose yeast soluble (SDYS) medium in 250-ml Erlenmeyer flasks and grown at 28°C and 180 rpm for 12 hours. *P. pastoris* GS115 was used for in vivo and in vitro biotransformation. The strains were maintained on a yeast extract, peptone, and dextrose (YPD) plate. *Escherichia coli* DH5α was used for cloning and plasmid propagation. Genomic DNA was extracted from the mycelia of OUCMDZ-5210 strain grown in potato dextrose broth (PDB; PDA medium without agar) after 5 days. The whole genome of it was sequenced using Illumina HiSeq PE150 at Beijing Allwegene Technology Co. Ltd. All the culture media used in the experiments were provided in table S20.

### Fermentation, extraction, and purification

The mycelia of fungal transformants grown on agar plates were inoculated into fungus no. 2 (100 ml) in 250-ml Erlenmeyer flasks and grown at 28°C and 180 rpm for 7 days. An equal volume of ethyl acetate was added to the culture and ultrasonicated for 30 min to extract the metabolites. Then, the supernatant and mycelia were separated and extracted with ethyl acetate. The crude extracts were dissolved in 1.5 ml of methanol and analyzed by LC-MS or HPLC. A 20.2-g extract from 15 liters of fungus no.2 (pH 3.0) was obtained and yielded new βCs. The detailed purification was described in Supplementary Texts 2 and 3. The data of new compounds were shown as below; data for other compounds are shown in Supplementary Text 29.

#### Fumitremorgin P (2)

Pale-green amorphous powder; chemical formula: C_22_H_23_N_3_O_3_; HR-ESI-MS: *m/z* 378.1805 [M+H]^+^ (calcd for C_22_H_24_N_3_O_3_^+^); ${\left[\alpha \right]}_{D}^{31}$ +64.2 (*c* 0.1, MeOH); UV (MeOH) $\lambda_{\text{max}}$(logε ) 203.5 (2.2) nm, 235.0 (2.0) nm, 266.5 (1.4) nm, 299.5 (1.1) nm, 376.5 (1.1) nm; ECD (MeOH, 0.13 mM) $\lambda_{\text{max}}$(Δε ) 215.0 (+9.1) nm, 262.5 (−1.3) nm, 272.5 (+0.7) nm, 300.0 (−2.3) nm, 366.0 (+2.7) nm; IR (KBr) $\nu_{\text{max}}$ 3396, 2962, 2926, 1680, 1607, 1450, 1381, 1261, 1031, 803 cm^−1^; for ^1^H NMR and ^13^C NMR data, see table S2.

#### Secofumitremorgins C/D (4a/4b)

Yellow oil; chemical formula: C_22_H_23_N_3_O_4_; HR-ESI-MS: *m/z* 394.1761 [M+H]^+^ (calcd for C_22_H_24_N_3_O_4_^+^); ${\left[\alpha \right]}_{D}^{31}$ −24.2 (*c* 0.1, MeOH); UV (MeOH) $\lambda_{\text{max}}$(logε ) 214.0 (0.8) nm, 283.0 (0.9) nm, 344.5 (0.4) nm; ECD (MeOH, 0.13 mM) $\lambda_{\text{max}}$(Δε ) 202.5 (+6.4) nm, 230.5 (−1.0) nm, 242.5 (−0.8) nm, 279.5 (−5.1) nm, 319.5 (+0.4) nm; IR (KBr) $\nu_{\text{max}}$ 3107, 1737, 1680, 1629, 1423, 1365, 1274, 1201, 752 cm^−1^; for ^1^H NMR and ^13^C NMR data, see table S4.

#### Secofumitremorgins E/F (9a/9b)

Pink oil; chemical formula: C_27_H_31_N_3_O_4_; HR-ESI-MS: *m/z* 462.2386 [M+H]^+^ (calcd for C_27_H_32_N_3_O_4_^+^); ${\left[\alpha \right]}_{D}^{31}$ −16.6 (*c* 0.1, MeOH); UV (MeOH) $\lambda_{\text{max}}$(logε ) 199.5 (1.6) nm, 282.0 (1.0) nm, 345.5 (0.3) nm; ECD (MeOH, 0.11 mM) $\lambda_{\text{max}}$(Δε ) 214.5 (+4.7) nm, 233.0 (−3.1) nm, 255.5 (−1.1) nm, 285.5 (−5.6) nm, 309.5 (+0.3) nm; IR (KBr) $\nu_{\text{max}}$ 2993, 1682, 1209, 1135, 732 cm^−1^; for ^1^H NMR and ^13^C NMR data, see table S9.

#### Secofumitremorgins G/H (10a/10b)

Pink oil; chemical formula: C_28_H_33_N_3_O_4_; HR-ESI-MS: *m/z* 476.2543 [M+H]^+^ (calcd for C_28_H_34_N_3_O_4_^+^); ${\left[\alpha \right]}_{D}^{31}$ −16.6 (*c* 0.1, MeOH); UV (MeOH) $\lambda_{\text{max}}$(logε ) 191.5 (1.4) nm, 283.0 (1.0) nm, 348.0 (0.3) nm; ECD (MeOH, 0.10 mM) $\lambda_{\text{max}}$(Δε ) 201.5 (+3.9) nm, 240.5 (−3.4) nm, 262.5 (−0.5) nm, 290.0 (−2.4) nm, 311.5 (+0.4) nm; IR (KBr) $\nu_{\text{max}}$ 1683, 1450, 1397, 1209, 1124, 798 cm^−1^; for ^1^H NMR and ^13^C NMR data, see table S10.

#### Demethoxyfumitremorgin P (12)

Pale-green amorphous powder; chemical formula: C_21_H_21_N_3_O_2_; ESI-MS: *m/z* 348.2 [M+H]^+^ (calcd for C_21_H_22_N_3_O_2_^+^); ${\left[\alpha \right]}_{D}^{31}$ +64.2 (*c* 0.1, MeOH); UV (MeOH) $\lambda_{\text{max}}$(logε ) 201.5 (1.6) nm, 236.3 (2.4) nm, 260.3 (1.2) nm, 282.3 (1.1) nm, 368.3 (1.2) nm; for ^1^H NMR and ^13^C NMR data, see table S12.

### Structural characterization of compounds

Detailed processes about structural analysis and absolute configuration identification of compounds were shown Supplementary Text 30.

### NMR and Gibbs free energy calculation

The structural formulas were drawn using ChemDraw. The conformational search was carried out using Spartan’14. For structural optimization, NMR, and Gibbs free energy calculation, Gaussian 16W and GaussView were used. For experimental procedures, theory, and calculation details, see Supplementary Texts 25 and 26 and tables S22 and S34 to S41. The DP4+ probability analysis can be found in tables S26 to S33.

### Marfey hydrolysis

Derivatization of standard amino acids: A standard amino acid sample of 0.1 mg was dissolved in 200 μl of distilled water. Sixty microliters of 1-Fluoro-2,4-dinitrophenyl-5-l-alanine amide (l-FDAA; 10 mg/ml), 200 μl of acetone, and 40 μl of NaHCO_3_ (1 M) were added sequentially, mixed well, and reacted in a water bath at 45°C for 2 hours. Then, the reaction was terminated by adding 20 μl of HCl (2 M). Acid hydrolysis and derivatization of compounds: 1 mg of compound was dissolved in 200 μl of distilled water, 40 μl of HCl (1 M) was added, and the hydrolysis reaction was carried out at room temperature for 0.5 hours. The derivatization method was the same as that of the amino acid standard. The results were analyzed by ultra-performance liquid chromatography (UPLC; fig. S63). The detailed process was described in Supplementary Text 15.

### Bioinformatics analysis

Analysis of secondary metabolite biosynthesis gene clusters in the genome of OUCMDZ-5210 was performed by the antiSMASH 5.0 fungal version. The predicted function of genes in the gene clusters was analyzed by BlastP (https://blast.ncbi.nlm.nih.gov/Blast.cgi). Multiple sequence alignments were performed by DNAMAN. Protein domains were predicted using the InterPro program and Conserved Domain Database (CCD) of the National Center for Biotechnology (NCBI). The detailed process was described in Supplementary Text 18.

### Transformation of *A. fumigatus* OUCMDZ-5210

Fresh OUCMDZ-5210 mycelia were cultured in 100 ml of SDYS medium (28°C, 180 rpm, overnight). Conidia were harvested via sterile Miracloth filtration, resuspended in lysis buffer [Yatalase (10 mg/ml) in TF1 solution], and digested at 30°C for 3 hours (100 rpm). Protoplasts were filtered, centrifuged (4°C, 3000*g*, 10 min), and resuspended in STC buffer [1 M sorbitol, 10 mM CaCl_2_, and 50 mM tris-HCl (pH 7.5)]. For transformation, 1 μg of plasmid was mixed with 100 μl of protoplasts (5 min, ice), treated with PTC buffer [40% PEG-6000, 50 mM CaCl_2_, and 50 mM tris-HCl (pH 7.5); 20 min, room temperature), combined with molten PDA soft agar (1 M sorbitol and 0.8% agar, 50°C), and overlaid on PDA plates (1.5% agar). After 3 to 5 days at 28°C, transformants were purified twice on PDA plates and verified by polymerase chain reaction (PCR) (primers: table S21).

The plasmid pPIC9K-00794 was constructed by inserting the ftmG gene [1552 bp (base pairs), intron-free] into the pPIC9K-His backbone (methanol-inducible AOX1 promoter, α-secretion signal, His-tag, and HIS4 marker). The *ftm*G fragment was amplified from OUCMDZ-5210 cDNA (primers 794-9k-F/R), whereas pPIC9K-His was linearized via inverse PCR (primers 9K-F/R) to generate 20-bp homologous arms. Gel-purified fragments were ligated using In-Fusion enzyme. This ensured efficient protoplast transformation and precise gene integration for functional studies in *A. fumigatus*. The detailed process was described in Supplementary Text 21.

### Transformation of *P. pastoris* GS115

Single colonies of *P. pastoris* GS115 were inoculated into 5 ml of YPD overnight. The overnight culture was then transformed into 50 ml of YPD and grown until OD_600_ (optical density at 600 nm) reached 0.8 to 1.5. Cells of yeast were harvested by centrifugation at 4°C and 1500*g* for 5 min and washed twice with sterile water. Then, the pellet was resuspended into 20 ml of LiAc buffer [0.6 M sorbitol, 0.1 M LiAc, 10 mM dithiothreitol, and 10 mM tris-HCl (pH 7.5)] and inoculated at 28°C for 30 min. The pellet was resuspended in 1 ml of precooled 1 M sorbitol buffer and washed three times. Per 80 μl of competent cells was mixed with 100 ng of the plasmid pPIC9K-794 linearized with SacI and incubated on ice for 5 min. The competent cells were electroporated using the procedures set for *P. pastoris* by a pulse cell transfection system. Electroporated cells were immediately recovered in 1 ml of precooled sorbitol and then spread on the SC medium with methanol as the sole carbon source and lacking histidine. Transformants with high copy number were selected on a yeast extract-peptone-dextrose-sorbitol (YPDS) plate with zeocin (4 mg/ml). The correct transformants were confirmed by PCR using primer pairs alpha-Factor-F/3’-AOX (table S21). The detailed process was described in Supplementary Text 22.

### Precursor feeding assay

A 0.1-mg compound was dissolved in 200 μl of methanol. One hundred microliters of this solution was aspirated and subsequently added to 150 ml of culture medium derived from a 24-hour induction culture of yeast that had recombinantly expressed a specific protein. Every 2 hours, 20 ml of the culture medium was collected and extracted with ethyl acetate for a duration of 0.5 hours. Following evaporation, 150 μl of methanol was added to dissolve the residue. The solution was then centrifuged, and the supernatant was analyzed by UPLC/MS. The detailed process was described in Supplementary Text 5.

### Nonenzymatic transformation

The compound at a concentration of 0.5 mg/ml in methanol solution was added into the vials containing fungus no. 2 medium with natural pH and pH 2.3, respectively. The natural pH medium without the compound was the blank control. All three were placed in a shaker at 180 rpm and 28°C for 7 days, respectively. An equal volume of ethyl acetate was used for extraction. The results were analyzed by UPLC. The detailed process was described in Supplementary Texts 6 to 9.

### Experiment on the effect of oxygen on 2

One milligram of compound **2** in 1 ml of methanol was prepared as a solution (1 mg/ml). A 0.5-ml solution was added to cillin vials separately, and then 0.5 ml of water was added to each. After sealing them well, oxygen and nitrogen were passed into them separately and the reaction was carried out for 7 days. The reaction solution was extracted once using an equal volume of ethyl acetate separately, and the ethyl acetate phase was collected and evaporated; the extract was dissolved in 200 μl of methanol and centrifuged at 1000 rpm for 5 min, and the supernatants were analyzed using HPLC. The detailed process was described in Supplementary Text 10.

### Isotope tracing experiment

A 0.2-mg compound **2** was dissolved with 100 μl of methanol, 50 μl of each was pipetted into an Eppendorf tube, and then 200 μl of each of heavy oxygen water and water was added, followed by 50 μl of each of pH 2.2 HCl acid water to adjust the pH to 3.0. The reaction was tightly wrapped with a microporous filter membrane and placed in a shaker at 28°C for 7 days for HPLC and MS monitoring.

A 0.2-mg compound **2** was dissolved in 100 μl of methanol. Two aliquots of 50 μl each were transferred into separate Eppendorf tubes. To each tube, 200 μl of pH 3.0 deoxygenated HCl-acidified water was added. One tube was then flushed with regular ^16^O_2_, whereas the other was flushed with ^18^O_2_. Each reaction mixture was sealed with a microporous membrane, incubated on a shaker at 28°C for 7 days, and subsequently analyzed by HPLC and MS.

### Cell proliferation inhibition was determined by CCK-8 assay

Cell Counting Kit-8 (CCK-8) was used to evaluate the proliferation inhibitory activity of the compounds (*33*–*38*). For specific experimental details and data, see Supplementary Text 27 and table S23.

### HIF transcriptional activity assay

For cellular transfection in 3.5-cm dishes, 80% confluent cells were transfected with polyethylenimine (PEI)–plasmid complexes prepared in 100 μl of serum-free Dulbecco’s modified Eagle’s medium (DMEM) (37°C, 15 min), diluted with 900 μl of DMEM, and applied after D’Hanks washing. Post–4- to 6-hour serum starvation, 20% FBS (fetal bovine serum)–DMEM was added. After 24 hours, compound-treated cells were subjected to hypoxia (1% O_2_/5% CO_2_/94% N_2_) for 24 hours. For dual-luciferase assays, cells in 24-well plates were cotransfected with HIF-responsive ENO1-luc (P2.1) and pRL-SV40 normalization plasmids. Posttransfection lysates (1×PLB) were centrifuged (12,000 rpm, 4°C, 5 min), with supernatants analyzed sequentially using LAR (firefly luc) and Stop & Glo (Renilla luc) reagents. HIF transcriptional activity was calculated as firefly/Renilla luminescence ratio. All procedures were conducted under sterile conditions with appropriate controls. The detailed process was described in Supplementary Text 12.

### Erythrocyte inhibitory activity test

All animal procedures were conducted according to the guidelines established by the University Committee on the Use and Care of Animals at the Ocean University of China (ethics review reference number: OUC-SMP-2022-08-07).

Purely congenic male and female fish and wild-type male and female fish were mated and spawned simultaneously, and the fertilized eggs laid by each were collected and placed in different petri dishes and incubated in an incubator at 28°C. After 48 hours of incubation, staining was performed to observe the synthesis of embryonic hemoglobin. At 48 hours postfertilization (hpf), the egg membrane was peeled off under the microscope and at least three groups of parallel experiments were performed, with 10 zebrafish/juveniles randomly selected from each group and fixed with 4% paraformaldehyde (PFA) for 30 min. After fixation, PFA was removed; embryos were washed three times in phosphate-buffered saline (PBS) for 3 min each time. Then, incubation in staining buffer [*o*-anisidine (0.6 mg/ml), 10 mM sodium acetate (pH 5.2), 0.65% hydrogen peroxide, and 40% ethanol, water fixation] was conducted for 15 min in the dark and washed three times in Phosphate-Buffered Saline with Tween 20 (PBST) for 3 min each time. The staining was conducted under microscopic observation and photography. The detailed process was described in Supplementary Text 13.

### Antiangiogenic activity test

All animal procedures were conducted according to the guidelines established by the University Committee on the Use and Care of Animals at the Ocean University of China (ethics review reference number: OUC-SMP-2022-08-07).

Adult male *Tg*(*lkl*::*eGFP*) zebrafish (two pairs per mating box, four boxes total) were spawned via light-triggered insert removal, with embryos collected every 20 min in embryo rearing solution (ERS) for developmental synchronization. Unfertilized embryos were discarded at 12 hpf; surviving embryos (10 per well, duplicate) were transferred to 24-well plates containing phenylthiourea (PTU)–supplemented medium to suppress melanogenesis. Test compounds were applied at 18 hpf. Vascular development was assessed at 28 hpf using fluorescence microscopy, with dimethyl sulfoxide (DMSO) controls confirming normal angiogenesis. For imaging, seven embryos per group were dechorionated, anesthetized in 0.8% tricaine (30 s), and dorsally mounted in 2% methylcellulose with standardized ocular alignment. Vascular analysis was repeated at 52 hpf under consistent imaging parameters to quantify developmental progression. The detailed process was described in Supplementary Text 14.

### Molecular docking

Molecular docking studies for compound **1** using Schrodinger 2021-3. The receptor (FtmG) used for docking was built by Swissmodel and Schrodinger 2021-3. PyMOL (version 4.6.0) was used for structural visualization and figure preparation. The detailed process was described in Supplementary Text 24.

### Site-directed mutagenesis of *ftm*G

For the construction of plasmids for site-directed mutation, pPIC9K-ftmG442A mutated the 442nd amino acid of FtmG protein from cysteine to alanine; PPIC9K-ftmG292L mutated the 292nd amino acid of FtmG protein from arginine to leucine. Construction of point mutation plasmid was performed by fusion PCR. Primer pair 9k-794-F/442c-a-1-R; 442C-A-2-F/9k-794-R; 9k-794-F292R-L-1-R; 292R-L-2-F9k-794-R were used to amplify gene fragments. The point mutation plasmid was constructed by connecting the two fragments with the skeleton plasmid pPIC9K by In-Fusion (primers: table S21). The detailed process was described in Supplementary Text 23.

### Experimental data and methods

For NMR data, see tables S1 to S16. For UPLC conditions, see tables S17 to S19. For medium composition, see table S20. For primer sequence, see table S21. For nucleotide sequences for genes and plasmid, see tables S24 and S25. For LC-MS/MS–based molecular networking, see fig. S2. For HR-ESI-MS and NMR spectra, see figs. S3 to S59. For ECD curves, see fig. S60. For UPLC fingerprint analysis of the crude extract of OUCMDZ-5210, see figs. S61 and S62.

## Supplementary Materials

### This PDF file includes:

Supplementary Text Figs. S1 to S63 Tables S1 to S41 References
