# Supplementary material for: A unique mechanism involving cocatalysis of enzyme and nonenzyme to form β-carboline and spirotryprostatins in Aspergillus fumigatus
Source: Sci Adv. 2025 Oct 10;11(41):eadz2319. doi: 10.1126/sciadv.adz2319 (PMC12513458; doi:10.1126/sciadv.adz2319)
Supplement: Supplementary file 1 — Supplementary Text Figs. S1 to S63 Tables S1 to S41 References [file sciadv.adz2319_sm.pdf]

Science Advances
AAAS

Supplementary Materials for

A unique mechanism involving cocatalysis of enzyme and nonenzyme to form β-carboline and spirotryprostatins in *Aspergillus fumigatus*

Hai Gao *et al.*

Corresponding author: Weiming Zhu, weimingzhu@ouc.edu.cn; Yi Wang, wangyi0213@ouc.edu.cn

Sci. Adv. **11**, eadz2319 (2025)
DOI: 10.1126/sciadv.adz2319

This PDF file includes:

Supplementary Text
Figs. S1 to S63
Tables S1 to S41
References

Supplementary Text

1. Experimental instruments

ECD spectra were determined using a JASCO J-815 spectropolarimeter. UV spectras were collected from Waters 2487 detector. IR spectra were performed on a Bruker Tensor-27 spectrophotometer with KBr discs. A JASCO P-1020 digital polarimeter was used to obtain optical rotations. NMR spectra were carried out on an Agilent 500 MHz DD2 spectrometer, and a Bruker AVANCE NEO 400 MHz with tetramethylsilane as an internal standard. High resolution ESI-TOF mass spectra data were recorded using a Thermo Scientific LTQ Orbitrap XL mass spectrometer. Low-resolution LC/ESI-MS data were measured using a Waters ACQUITY SQD 2 UPLC/MS system with a reversed-phase C18 column (ACQUITY UPLC BEH C18, 2.1 mm × 50 mm, 1.7 μm) at a flow rate of 0.4 mL/min. Semipreparative HPLC was conducted with an ODS-C18 column (Waters, YMC-Pack ODS-A, 20 × 250 mm, 5 μm, 3 mL/min). Silica gel (200–300 or 100–200 mesh, Marine Chemical Factory of Qingdao) was used for vacuum-liquid chromatography (VLC). RP-18 silica gel (YMC ODS-A, 50 μm) and Sephadex LH-20 (Amersham Biosciences) were used for column chromatography (CC).

^{1}H NMR used the residual solvent peak as a reference, $CDCl_3$ δ 7.26, CD_3OD δ 3.31 and DMSO-d_6 δ 2.50. ^{13}C NMR used the solvent peak as a reference, $CDCl_3$ δ 77.16, CD_3OD δ 49.00 and DMSO-d_6 δ 39.52. The mass spectra were recorded using a Waters liquid chromatography mass spectrometer.

2. Preparation of Compounds **1–3**, **6–8**, **11–13**, and **15–17**

The wild strain of *Aspergillus fumigatus* OUCMDZ-5210 was inoculated in 15 L fungus No.2 and incubated for 36 days at 28 °C with natural pH in a shaking bed for mass fermentation. The fermentation broth and mycelium were crushed using a cell crusher, then extracted with ethyl acetate at the ratio of organic phase: aqueous phase = 2:1, sonicated for 30 min, and the upper organic phase was evaporated using a rotary evaporator to collect the crude extract from the organic phase, and this was repeated three times to obtain about 20.2 g of crude extract. The fractions eluted between 50:1 and 10:1 were combined. Then, 20 fractions were collected using Sephadex LH-20 column chromatography (CH_2Cl_2:MeOH=1:1, MeOH) and prepared by HPLC for fraction 9 (ODS-A C18 column, 2.5 mL/min, 28 °C, CH_3CN: H_2O=40: 60) to obtain compounds **1** (7.0 mg), **2** (2.4 mg), **6** (1.0 mg), **7** (5.5 mg), **11** (2.1 mg), **12** (0.7 mg), **15** (0.5 mg) and **16** (2.1 mg), respectively, as Compounds **3**, **13** and **17** were obtained as 1.2 mg, 14.0 mg and 0.8 mg each, respectively. HPLC was prepared (ODS-A C18 column, 2.5 mL/min, 28 °C, CH_3CN: H_2O=50: 50) and compounds **8** were obtained as 2.6 mg.

3. Preparation of compounds **4**, **5**, **9** and **10**

The wild strain of Aspergillus fumigatus OUCMDZ-5210 was inoculated in 15 L fungus No.2 and incubated for 36 days at 28 °C with pH 3.0 in a shaking bed for mass fermentation. The fermentation broth and mycelium were crushed using a cell crusher, then extracted with ethyl acetate at the ratio of organic phase: aqueous phase = 2:1, sonicated for 30 min, and the upper organic phase was evaporated using a rotary evaporator to collect the crude extract from the organic phase, and this was repeated three times to obtain about 18.9 g of crude extract. The fractions eluted between 50:1 and 10:1 were combined. Ten fractions were then collected using Sephadex LH-20 column chromatography (CH_2Cl_2: MeOH=1:1, MeOH) and prepared by HPLC for fraction 6 (ODS-A C18 column, 2.5 ml/min, 28 °C, CH_3CN:H_2O=40: 60) to obtain compounds

4 and **5**, 3.5 mg and 1.7 mg each, respectively. Fraction 5 was prepared by HPLC (ODS-A C18 column, 2.5 ml/min, 28 °C, CH_3CN: H_2O=28: 72) and compounds **9** and **10** were obtained as 1.5 mg and 1.0 mg, respectively.

4. Semi-synthesis of Compound **5**

5 mg of compound **4** was dissolved by adding 2 ml of methanol and 200 μL of acetyl chloride in an ice bath, followed by heating reflux at 400 r/min, 66 °C for 5 h. The reaction solution was evaporated, dissolved with MeOH and prepared using HPLC (ODS-A C18 column, 2.5 mL/min, 28 °C, CH_3CN: H_2O=40: 60) , the methyl esterified compound **5** was obtained in a total of 2.8 mg with a calculated yield of 46 %. It was combined with the **5** obtained in isolation from the natural product for a total of 3.1 mg.

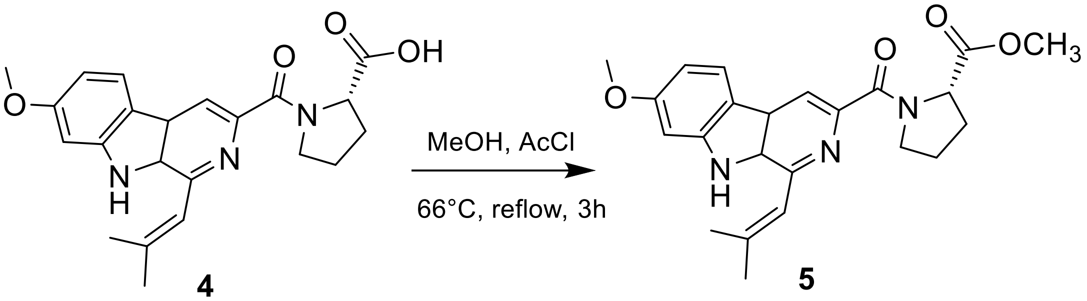

4 **5**

5. Precursor feeding method

0.1 mg of compound was taken in an EP tube, dissolved in 200 μL of methanol, and 100 μL of the solution was aspirated using a sterile dropper in an ultra-clean table, added to 150 mL of culture medium from the induction culture of recombinantly expressed yeast for 24 h. 20 mL of culture medium was taken every 2 h in a large 50 mL centrifuge tube, extracted by ultrasonic shaking with ethyl acetate for 0.5 h, evaporated, added to 150 μL methanol was added to dissolve, centrifuged, and analyzed by HPLC.

6. Non-enzymatic transformation of **2** to **3** and **4**

Take 1 mg of compound **2** and dissolve it in 1 mL of methanol to prepare a solution of 1 mg/mL. Take 5 mL of each of the prepared fungus No. 2 in two cilium bottles and set aside, then adjust the pH of the remaining medium to 2.30 and take 5ml in the cilium bottles. The three bottles were wrapped with microporous filter membrane and autoclaved in a sterilizer. After cooling, 0.5 mL of 1 mg/mL of solution **2** was added to each of the natural pH and pH 2.30 cillin bottles in the ultra clean table, and the remaining bottle of natural pH medium was used as a blank control. Then put into a shaker at 180 r/min, 28 °C, and reacted for 7 days. The culture solution was extracted once using an equal volume of ethyl acetate separately, the ethyl acetate phase was collected, evaporated, and the extract was dissolved in 200 μL of methanol, centrifuged at 1000 r/min for 5 min, and the supernatants were taken in liquid mass vials separately and monitored and analyzed using a liquid chromatography mass spectrometer at 280 nm.

7. Non-enzymatic transformation of **7** to **3** and **4**

Take 2 mg of compound **7** and dissolve it in 2 mL of methanol to make a solution of 1 mg/mL. Take 5 ml of water from each of the two cillin bottles and set aside, one of which was adjusted to pH 2.0. 5 mL of the prepared fungus No. 2 was taken in the cillin bottle, and then the remaining medium was adjusted to pH 2.30 and 5ml was taken in the cillin bottle. Then the four bottles were wrapped with microporous filter membrane and put into the sterilizer for autoclaving. After

cooling, 0.5 mL of 1 mg/mL of **7** solution was added to each of them in an ultra-clean table. the reaction was carried out in a shaker at 180 r/min, 28 °C, for 14 days. The culture solution was extracted once using an equal volume of ethyl acetate separately, the ethyl acetate phase was collected, evaporated, and the extract was dissolved in 200 μL of methanol, centrifuged at 1000 r/min for 5 min, and the supernatants were taken in liquid mass vials separately and monitored and analyzed using a liquid chromatography mass spectrometer at 280 nm.

8. Non-enzymatic transformation of **12** to **13**

0.2 mg of compound **12** was dissolved in 200 μL of methanol to prepare a 1 mg/mL solution and set aside. 5 mL of the prepared fungus No. 2 was taken in a cillin bottle and placed in an autoclave after wrapping it well with a microporous filter membrane to inactivate it. After cooling, 200 μL of 1 mg/mL of **12** solution was added in the ultra clean table. Mix well and take 1 mL of the mixed solution. The reaction solution was extracted once using an equal volume of ethyl acetate, the ethyl acetate phase was collected, evaporated, and the extract was dissolved in 200 μl of methanol and centrifuged at 1000 r/min for 5 min, and the supernatant was taken in a liquid mass vial and monitored and analyzed at 280 nm using a liquid chromatography mass spectrometer. The remaining solution was placed in a shaker at 180 r/min, 28 °C for 48 h. The reaction solution was extracted once with an equal volume of ethyl acetate, the ethyl acetate phase was collected, evaporated, and the extract was dissolved in 200 μL of methanol, centrifuged at 1000 r/min for 5 min, and the supernatant was taken in a liquid vial and analyzed by liquid chromatography mass spectrometry at 280 nm.

9. Non-enzymatic transformation of **16** to **13** and **14**

Take 2 mg of compound **16** and dissolve it in 2mL of methanol to make a solution of 1mg/mL. Take 5mL of water from each of the two cillin bottles and set aside, one of which was adjusted to pH 2.0. 5 mL of the prepared fungus No. 2 was taken in the cillin bottle, and then the remaining medium was adjusted to pH 2.30 and 5 mL was taken in the cillin bottle. Then the four bottles were wrapped with microporous filter membrane and put into the sterilizer for autoclaving. After cooling, 0.5 mL of 1 mg/mL of **16** solution was added to each of them in an ultra-clean table. the reaction was carried out in a shaker at 180 r/min, 28°C, for 14 days. The culture solution was extracted once using an equal volume of ethyl acetate separately, the ethyl acetate phase was collected, evaporated, and the extract was dissolved in 200 μL of methanol, centrifuged at 1000 r/min for 5 min, and the supernatants were taken in liquid mass vials separately and monitored and analyzed using a liquid chromatography mass spectrometer at 280 nm.

10. Experiment on the effect of oxygen on **2**

Prepare a 1 mg/mL solution by dissolving 1 mg of compound **2** in 1 mL of methanol. Add 0.5ml to each of the 2 cillin vials separately, and then add 0.5 mL of water to each. After sealing them well, oxygen and nitrogen were passed into them separately and the reaction was carried out for 7 days. The reaction solution was extracted once using an equal volume of ethyl acetate separately, the ethyl acetate phase was collected, evaporated, and the extract was dissolved in 200 μL of methanol, centrifuged at 1000 r/min for 5 min, and the supernatants were taken in liquid vials separately and monitored for analysis using full-band HPLC at 280 nm.

11. Cytotoxic activity test method

Principle of detection:

CCK-8 reagent contains WST-8, which is reduced to a highly water-soluble yellow methanogenic product (Formazan) by dehydrogenase in cell mitochondria in the presence of the electron carrier 1-methoxy-5-methylphenidate sulfate (1-Methoxy PMS). The amount of formazan produced is proportional to the number of living cells.

Experimental methods:

(1) Inoculation of cells: cells were prepared into individual cell suspensions with culture medium containing 10% fetal bovine serum, and 96-well plates were inoculated with 90 μL of 5×104/mL of walled cells and 9×104/mL of suspended cells per well, and pre-cultured for 24 h at 5% CO_2, 37°C.

(2) Add the sample solution to be tested: 10 μL of sample solution was added to each well, 1 concentration was set for each sample of the active primary sieve, and 3 replicate wells were set; 8 concentrations (including 0 concentration) were determined for IC_{50}, and 3 replicate wells were set for each concentration; incubated in the incubator for 48 h. Blank group (Blank), control group (Control) and drug group (Drug) were set for the experiment.

(3) Color development: walled cells were aspirated from the old medium and drug solution (suspension cells were directly added to 10 μL CCK-8 solution stock solution), and 100 μL of CCK-8 solution diluted tenfold was added to each well and incubated for 1-4 h at 37°C with 5% CO_2 (operated under protection from light and observed in real time).

(4) Assay: Measure the absorbance at 450 nm with an enzyme marker and record the raw data results.

(5) Apply Excel software to standardize the raw data, and calculate the cell proliferation inhibition rate (formula = (ODControl-ODDrug)/(ODControl-ODBlank) × 100%) by OD value per well for the primary screening and statistical inhibition rate. IC_{50} was calculated by GraphPad Prism 8 (version 8.0.2. GraphPad Software Inc), and the experimental results were expressed as ± SD.

(6) Positive control: adriamycin hydrochloride Doxorubicin (Dox)

12. <u>HIF</u> <u>transcriptional</u> <u>activity</u> <u>assay</u>

Cell transfection (3.5 cm culture dish as an example), compound addition and hypoxia:

(1) When the cells grow to about 80 %, add the plasmid and transfection reagent (PEI) to 100 μL of serum-free DMEM according to the experimental requirements, gently mix them into the incubator at 37°C for 15 min in an air bath, remove them and then add 900 μL of serum-free DMEM, gently mix them with a pipette gun, then take out the cells to be transfected, wash them with D' Hanks and then add DMEM mixed with plasmid into the incubator. After 4-6 h of starvation treatment, add 1 ml of DMEM medium with 20 % FBS to continue the culture.

(2) After 24 h of transfection, different doses of compounds were dissolved into the medium, added to the transfected cells, and then put into the cellular hypoxia box, ventilated with mixed gas (1 % O_2+5 % CO_2 + 94 % N_2) for 10 min, and the venting port of the hypoxia box was fastened and put into the incubator at 37 °C. After 24 h of anoxia, the anoxic box was quickly opened and the cells were removed for the next treatment. (The moment the anoxia box was opened, the sound of gas leakage was heard to prove the success of anoxia).

Dual luciferase reporter gene (Luciferase) assay

(1) Cells were divided into 24-well plates and transfected with two luciferase reporter plasmids when the cells reached 80% growth. Two plasmids were used in this study, P 2.1 (ENO1-luc), which detects HIF transcriptional activity, and pRL-SV40, a sea kidney luciferase reporter plasmid, which serves as an internal reference.

(2) After 24 h of transfection, the cells were removed from the 37 °C incubator, the medium was aspirated and washed once with D' Hanks, 1×PLB lysis solution was added, and the cells were lysed for 15 min in a constant temperature shaker. After lysis was completed, the cells were blown off with a pipette or scraped off with a cell scraper, and the lysate and cell debris were completely transferred to a 1.5 mL EP tube. Place in a pre-cooled 4 °C centrifuge and centrifuge at 12000 rpm for 5 min.

(3) Add 40 μL of chromogenic solution LAR to a clean 96-well plate, then add 40 μL of cell lysate supernatant, mix well with a gun tip, put it into a pre-warmed enzyme marker, follow the procedure to detect the activity of firefly luciferase, and save the data. Then 40 μL of SG chromogenic solution was added to the mixture, and the sea kidney luciferase activity was detected and the data were saved, and the ratio of the two represented the transcriptional activity of HIF.

13. Erythrocyte inhibitory activity test

All animal procedures were conducted according to the guidelines established by the University Committee on the Use and Care of Animals at the Ocean University of China. Ethics Review Reference Number: OUC-SMP-2022-08-07.

Principle of solid blue (0-dianisidine) staining:

Hemoglobin has a peroxidase-like activity, which can catalyze the release of oxygen from hydrogen peroxide, causing 0-dianisidine to change color and eventually take on a rusty color, thus observing the synthesis of hemoglobin in zebrafish embryos.

The specific experimental steps are as follows.

(1) Purely congenic male and female fish and WT male and female fish were mated and spawned simultaneously, and the fertilized eggs laid by each were collected and placed in different Petri dishes and incubated in an incubator at 28 °C. After 48 h of incubation, 0-diani-sidine staining was performed to observe the synthesis of embryonic hemoglobin.

(2) At 48 hpf, the egg membrane was peeled off under the microscope and at least three groups of parallel experiments were performed, with 10 zebrafish/juveniles randomly selected from each group and fixed with 4 % paraformaldehyde (PFA) for 30 min (the fixation time should be the same for each group, otherwise the embryos become lighter in color with longer fixation time).

(3) After fixation, remove PFA; embryos are washed 3 times in PBS for 3 min each time; (PBS: maintain optimal conditions to ensure biologically active substances and maintain their integrity.)

(4) Then incubate in staining buffer (0.6 mg/mL o-anisidine, 10 mM sodium acetate (pH 5.2), 0.65 % hydrogen peroxide and 40 % ethanol, water fixation) for 15 min in the dark. (Recipe as above, avoid light throughout, ready to use)

(5) Wash 3 times in PBST for 3 min each time; (terminate the staining)

(6) Microscopic observation of staining and photography.

14. Anti-angiogenic activity test

All animal procedures were conducted according to the guidelines established by the University Committee on the Use and Care of Animals at the Ocean University of China. Ethics Review Reference Number: OUC-SMP-2022-08-07.

Zebrafish embryo dosing treatment:

(1) The night before, two healthy adult male *Tg*(*lkl*::*eGFP*) zebrafish were placed in one side of the mating box and two healthy adult *Tg*(*lkl*::*eGFP*) zebrafish were placed in the other side, and about four boxes of zebrafish were mated each time to ensure the quantity and quality of embryos.

(2) The next day, within 5 min after the light of the fish room was turned on, the inserts of the mating boxes were removed and the spawning was observed every 20 min.

(3) When zebrafish start to spawn, collect zebrafish embryos in batches, taking care not to exceed 20 min between each batch of embryos.

(4) After collecting embryos, pick out the dead embryos and feces, add the appropriate amount of embryo culture medium (ERS), indicate the time of embryo collection on the embryo culture dish, and put them into the embryo culture incubator.

(5) Prepare compound solutions according to different experimental needs; at this time, dilute compounds with medium containing PTU, add PTU to remove zebrafish melanin so as not to affect the observation of embryonic vascular fluorescence; when preparing solutions with different concentrations of the same compound, pay attention to the use of equal concentration gradient dilution method to reduce experimental errors, and set up two complex wells for each group of compounds; when preparing combined drug solutions, prepare 2 times the concentration of each drug solution (2) When preparing the combined drug solution, prepare 2 times the concentration of each drug solution, and then use equal concentrations of DMSO as the negative control and 5 nM of Sorafenib as the positive control;

(6) When the embryos developed to 12 hpf, the embryos were removed, and the unfertilized embryos were obviously whitened, so the unfertilized embryos were picked out and transferred into clean 24-well plates with about 10 embryos per well, and two replicate wells were set up for each group.

(7) When the embryos developed to 18 hpf, the embryos were removed, the embryo medium was carefully aspirated and discarded, and the embryos were treated with the prepared compound solution and placed in the embryo incubator for further incubation.

(8) When the embryos developed to 28 hpf, the embryos were removed and placed under fluorescence microscope to observe the development of blood vessels in the DMSO group, and the preparation of photographs was started when the embryos in the DMSO group had fully developed blood vessels;

(9) The embryos were aspirated from the 24-well plate and the embryonic membranes were carefully removed under the microscope, taking care not to injure the embryos at this time, about 7 embryos were stripped from each group, and the remaining embryos continued to be cultured.

(10) Place the membrane-removed embryos in 0.8% tricaine under anesthesia for about 30 s, remove the embryos and place them in 2 % methylcellulose;

(11) The embryos were carefully turned over in position in methylcellulose so that the two eyes of the zebrafish embryos were perfectly coincident when the head was left and the tail was right, and the dorsal belly was lowered from the top when viewed vertically downward;

(12) Place the posed zebrafish under a fluorescence microscope to take pictures and record the vascular development status, taking pictures of all groups of zebrafish embryos in turn;

(13) When the embryos have developed to 52 hpf, remove the embryos and repeat steps 10, 11 and 12.

(14) Measure the intersegmental vessel length of each zebrafish embryo using image ProPlus6, and count the results.

(15) Calculate the inhibition rate of angiogenesis per zebrafish (inhibition rate = 100 % - intersegmental vessel length per embryo / average vessel length of control group × 100 %);

(16) The angiogenesis inhibition rate of zebrafish embryos at the same concentration was statistically analyzed and the inhibition rate of each compound was compared.

15. Marfey Hydrolysis

Derivatization of standard amino acids:

A standard amino acid sample of 0.1 mg was taken in an EP tube and dissolved in 200 μL distilled water. 60 μL L-FDAA (10 mg/mL), 200 μL acetone, 40 μL $NaHCO_3$ (1 mol/L) were added sequentially, mixed well and reacted in a water bath at 45 °C for 2 h. Then the reaction was terminated by adding 20 μL HCl (2 mol/L), centrifuged, and HPLC analysis.

Acid hydrolysis and derivatization of compounds:

The compound was then dissolved in 200 μL distilled water, and 60 μL L-FDAA (10 mg/mL), 200 μL acetone, 40 μL $NaHCO_3$ (1 mol/L), and 40 μL HCl (1 mol/L) were added sequentially. $NaHCO_3$ (1 mol/L), mixed well and reacted in a water bath at 45 °C for 2 h. The reaction was then terminated by the addition of 20 μL HCl (2 mol/L), centrifuged, and analyzed by HPLC.

16. Isotope tracing experiment

0.2 mg of compound **2** was dissolved with 100 μL of methanol, 50 μL of each was pipetted into an EP tube, and then 200 μL of each of heavy oxygen water and water was added, followed by 50 μL of each of pH 2.22 HCl acid water to adjust the pH to 3. The reaction was tightly wrapped with a microporous filter membrane and placed in a shaker at 28 °C for 7 days for HPLC and MS monitoring.

0.2 mg of compound **2** was dissolved in 100 μL methanol. Two aliquots of 50 μL each were transferred into separate EP tubes. To each tube, 200 μL of pH 3.0 deoxygenated HCl-acidified water was added. One tube was then flushed with regular $^{16}O_2$, while the other was flushed with $^{18}O_2$. Each reaction mixture was sealed with a microporous membrane, incubated on a shaker at 28 °C for 7 days, and subsequently analyzed by HPLC and MS.

17. Strains and culture conditions

OUCMDZ-5210 was isolated from a mangrove soil sample in Thailand in an acidic environment (pH5), and ITS strain was identified as Aspergillus fumigatus. The wild-type strain and transformants were maintained on PDA (20 % potato, 2 % glucose, 2 % agar) at 28 °C. For the preparation of protoplasts, fresh mycelia of Aspergillus fumigatus 5210 were inoculated in 100

mL SDYS medium (0.6% yeast extract, 0.3 % casein enzymatic hydrolysate, 0.3% casein acids hydrolysate, 1 % sucrose) in 250 mL Erlenmeyer flasks and grown at 28 °C, 180 rpm for 12 h.

Pichia pastoris GS115 were used for in vivo and in vitro biotransformation. The strains were maintained on YPD ((1 % yeast extract, 2 % peptone, 2 % dextrose, 1.5 % agar) plate.

Escherichia coli DH5α was used for cloning and plasmid propagation.

Genomic DNA was extracted from the mycelia of Aspergillus fumigatus 5210 strain grown in PDB (PDA medium without agar) after 5 days. The whole genome of Aspergillus fumigatus 5210 was sequenced using Illumina HiSeq PE150 at Beijing Allwegene Technology Co., Ltd.

18. Bioinformatics analysis

Analysis of secondary metabolite biosynthesis gene clusters in the genome of *Aspergillus fumigatus* OUCMDZ-5210 was performed by antiSMASH 5.0 fungal version. The predicted function of genes in the gene clusters were analyzed by BlastP (https://blast.ncbi.nlm.nih.gov/Blast.cgi). Multiple sequence alignments were performed by DNAMAN. Protein domains were predicted using InterPro program and Conserved Domain Database (CCD) of the National Center for Biotechnology (NCBI).

19. Fermentation and extraction

The mycelia of fungal transformants grown on agar plates were used as inoculum to inoculate the Fungus No. 2 (Supplementary Table 20) (100 mL) in 250 mL Erlenmeyer flasks and grow at 28 °C, 180 rpm for 7 days. An equal volume of ethyl acetate was added to the culture and ultrasonicated for 30 min to extract the metabolites. Then the supernatant and mycelia were separated, and extracted with ethyl acetate one more time. filtered, and concentrated in vacuo. The crude extracts were dissolved in 1.5 mL methanol and analyzed by LC-MS or HPLC.

20. Construction of vectors for gene disruption

For disruption of gene 789 (*ftm*C), flanking sequences of gene789 (1.0-1.5 kb LB and RB) were amplified using primer pairs 789-S-F/789-S-R, 789-X-F/789-X-R (Supplementary Table 21) using genomic DNA as template. The Hygromycin B -resistant gene expression cassette (hph) was amplified from plasmid pAG1-H3 with primer pairs 789-hph-F /789-hph-R. The three fragments were fused together using infusion to obtain the homologous recombination fragment for disruption of gene789 in *Aspergillus fumigatus* 5210.

For disruption of gene 792 (*ftm*E), flanking sequences of gene792 (1.0-1.5 kb LB and RB) were amplified using primer pairs 792-S-F/792-S-R,792-X-F/792-X-R (Supplementary Table 21) using genomic DNA as template. The Hygromycin B -resistant gene expression cassette (hph) was amplified from plasmid pAG1-H3 with primer pairs 792-hph-F /792-hph-R. The three fragments were fused together using infusion to obtain the homologous recombination fragment for disruption of gene792 in *Aspergillus fumigatus* 5210.

For disruption of gene 794 (*ftm*G), flanking sequences of gene794 (1.0-1.5 kb LB and RB) were amplified using primer pairs 794-S-F/794-S-R,794-X-F/794-X-R (Supplementary Table 21) using genomic DNA as template. The Hygromycin B -resistant gene expression cassette (hph) was amplified from plasmid pAG1-H3 with primer pairs 794-hph-F /794-hph-R. The three fragments were fused together using infusion to obtain the homologous recombination fragment for disruption of gene794 in *Aspergillus fumigatus* OUCMDZ-5210.

21. Transformation of *Aspergillus fumigatus* OUCMDZ-5210

For the preparation of protoplasts from Aspergillus fumigatus 5210, fresh mycelia were inoculated in 100 mL SDYS medium in 250 mL Erlenmeyer flasks and grown at 28 °C, 180 rpm overnight. Conidia were collected by filtering with sterile miracloth, and then re-suspended in 10 mL of sterilized lysis buffer (10 mg mL-1 Yatalase enzyme in TF1 solution) and incubated at 30°C, 100 rpm for 3 h. The protoplasts were collected by filtration and centrifuged at 4 °C, 3000 g, 10 min. The pellet was then resuspended in STC (1 M Sorbitol, 10 mM $CaCl_2$, 50 mM Tris-HCl pH 7.5). 1 μg plasmids were mixed with 100 μL protoplasts and incubated on ice for 5 min. Then 1 mL PTC (40% PEG6000, 50 mM $CaCl_2$, 50 mM Tris-HCl pH 7.5) was slowly added to the mixture and incubated at room temperature for 20 min. 10 mL molten (50 °C) PDA soft agar (PDA with 1 M sorbitol, 0.8% agar) was added to the mixture, then overlaid on PDA plate (PDA with 1.5% agar). After incubation at 28 °C for 3-5 days, transformants were picked and screened on PDA plate for two more rounds. Transformants were confirmed by PCR using primer pairs listed in Table S21.

The plasmid pPIC9K-00794 is composed of the backbone plasmid pPIC9K-His and the target gene ftmG (g00794). The pPIC9K-His vector carries the methanol-induced AOX1 promoter, the alpha secretion factor, the His-tagged protein, as well as the HIS4 selection marker. Using the cDNA of strain 5210 as a template, the intron-free g00794 fragment with a length of 1552 bp was amplified by PCR with the primer pair 794-9k-F/794-9k-R. The pPIC9K-His plasmid was subjected to inverse PCR with the primer pair 9K-F/9K-R to obtain the linearized plasmid. There is a 20-bp homologous arm between the primers and the plasmid. After gel recovery and purification of the two fragments, they were ligated by In-Fusion enzyme.

22. Transformation of *P. pastoris* GS115

Single colonies of P. pastoris GS115 were inoculated into 5 mL YPD overnight. The overnight culture was then transformed into 50 mL YPD and grown until OD600 reached 0.8-1.5. Cells of yeast were harvested by centrifugation at 4 °C, 1500 g, 5 min, washed with sterile water twice. Then the pellet was resuspended into 20 mL LiAc buffer (0.6 M sorbitol, 0.1 M LiAc, 10 mM DTT, 10 mM Tris-HCl pH 7.5) and inoculated at 28 °C for 30 min. The pellet was resuspended in 1 mL pre-cooled 1M sorbitol buffer and washed three times. Per 80 μL of competent cells was mixed with 100 ng plasmid pPIC9K-794 linearized with SacI and incubated on ice for 5 min. The competent cells were electroporated using the procedures set for P. pastoris by Pulse cell transfection system. Electroporated cells were immediately recovered in 1 mL pre-cooled sorbitol and then spread on the SC medium with methanol as the sole carbon source and lacking histidine. Transformants with high copy number were selected on YPDS plate with 4 mg/mL zeocin. The correct transformants were confirmed by PCR using primer pairs alpha-Factor-F/3'-AOX (Table S21).Feeding experiments using P. pastoris GS115 transformants Single colonies of P. pastoris GS115 transformants were picked and grown into YPD overnight, culture was then inoculated in 20 mL BMGY (1% yeast extract, 2% peptone, 2% dextrose, 100 mM potassium phosphate buffer pH 6.0, 1.34 % YNB (containing ammonium sulfate), 0.04% biotin, 1% glycerol) and shaken until the OD600 reached 1.5-2.0. Cells were centrifuged at 3000 g, 5 min, washed with sterile water twice. The supernatant was discarded and then the pellet was resuspended in 100 mL BMMY (1 % yeast extract, 2 % peptone, 2 % dextrose, 100 mM potassium phosphate buffer pH 6.0, 1.34 % YNB (containing ammonium sulfate), 0.04 % biotin, 0.5 % methanol) and grown at 30 °C, 200 rpm. 0.5 % (v/v) methanol was supplemented daily. Cells were harvested by centrifugation after 4

days and resuspended in 40 mL BMMY. Compound **1** was added into 20 mL culture at a final concentration of 0.1 mM and incubated for one day. The culture was extracted with ethyl acetate and used for LC-MS or HPLC analysis.

23. Site-directed mutagenesis of *ftm*G

For the construction of plasmids for site-directed mutation, pPIC9K-ftmG442A mutated the 442nd amino acid of FtmG protein from cysteine to alanine; PPIC9K-ftmG292L mutated the 292nd amino acid of FtmG protein from arginine to leucine. Construction of point mutation plasmid by fusion PCR. Primer pair 9k-794-f/442c-a-1-r; 442C-A-2-F/9k-794-R; 9k-794-F292R-L-1-R; 292R-L-2-F9k-794-R amplified gene fragment. The point mutation plasmid was constructed by connecting the two fragments with the skeleton plasmid pPIC9K by In-Fusion.

24. Modeling and docking

In this study, the Swissmodel server was employed to conduct the template protein retrieval work based on the protein sequence file. It was found that the CYP46A1 protein with high homology (PDB ID: 7LRL) also contains HEME (heme). Further, the Protein Structure Alignment module of the Schrodinger 2021-3 software was used to superimpose the target protein and the CYP46A1 protein in order to obtain the target protein-HEME complex. Subsequently, the Protein Preparation Workflow module was utilized to obtain the target protein-HEME complex with accurate valence bonds and without structural conflicts. This complex was used for the subsequent docking work with the small molecule compound **1**.

In this study, the Glide module of the Schrodinger 2021-3 software was used to construct the binding mode of compound 1 and the target protein. The target protein-HEME complex was used as the docking receptor. Before docking, the LigPrep module was used to calculate the charge state and the lowest energy conformation of the target compound in the physiological environment, and the confgen module generated 12 different conformations of the small molecule. In addition, the grid file defining the docking pocket was generated by the Receptor Grid Generation module in Maestro. The centroid of the amino acids around the active site was selected as the center of the active pocket, and a grid file with a volume of $10\times10\times10$ Å^3 was calculated and generated. Finally, the prepared files were subjected to molecular docking by the SP precision algorithm of the Glide docking module.

25. Gibbs free energy and Boltzmann distribution calculation

Gaussian16W was used to carry out the calculation of quantum chemistry. Firstly, the free energy thermal correction of the compound was calculated under the key words *opt freq b3lyp/6-311g (d) scrf = (SMD, solvent = DMSO) em=gd3bj*. Then, under this structure, the high-precision single-point energy of the compound can be obtained by using the keyword *m062x/6-311+g (2d, p) scrf=(SMD, solvent=DMSO) em=gd3*, and then the Gibbs free energy of the compound at standard temperature and pressure can be obtained by adding them, and its Boltzmann distribution can be calculated.

26. NMR Calculation and DP4+ Analysis

Take **4a**/**4b** as an example. The magnetic shielding tensor was calculated using the *GIAO Method* in the NMR keyword provided by the Gaussian 16W program. Then, a molecule selected as a reference substance in an actual experiment was calculated. By subtracting the calculated magnetic shielding values of the same atomic nuclei in the two molecules, the chemical shift value that could

be directly compared with the experimental value was obtained. First, Spartan'14 was used to search for low-energy conformations under the MMFF force field. The low-energy conformations obtained after screening were subjected to structural optimization in the gas phase at *B3LYP/6-31G(d)*. Subsequently, the magnetic shielding values were calculated at *B3LYP/6-31G(d) scrf(PCM, solvent=DMSO)*. The overall magnetic shielding value was weighted by calculating the weight of each conformation among all conformations according to the free energy of each conformation through the Boltzmann distribution. Then, it was converted into the chemical shifts of hydrogen and carbon using the scaling method.

The calculation methods for **9a**/**9b** and **10a**/**10b** were the same as above.

27. Cell proliferation inhibition was determined by CCK-8 assay

I Experimental purpose

Cell counting Kit (CCK-8) was used to evaluate the proliferation inhibitory activity of the compounds [33-38].

II Materials and reagents

Fetal bovine serum (FBS) 04-001-1acs), Biological Industries; PBS Phosphate buffer (PB180327)，Penicillin streptomycin sulfate double antibody mixture (100×) (PB180120)，RPMI1640 culture medium (PM150110)，DMEM high glucose culture medium (PM150110), MEM culture medium (PM150410), IMDM culture medium (PM150510), McCoy's 5A culture medium (PM150710) and Leibovitz's L-15 culture medium (PM151010), Procell Life Science&Technology Co.,Ltd.; Cell Counting Kit (CCK-8), Biosharp Life Sciences; Trypsin EDTA digestion solution (05200-056), GIBCO; Cell grade DMSO (d2650-100ml), Sigma; 96 well cell culture plate, 25 and 75 cm2 cell culture bottles, Corning Life Sciences (Wujiang) Co., Ltd.; Blood cell counting plate, Shanghai Qiujing biochemical reagent Instrument Co., Ltd.; Adriamycin hydrochloride (D8740), Solarbio Life Sciences;

III Instruments and equipment

Super clean workbench (sw-cj-2fd), Sujing Aetna; Microscope (nib-100), Ningbo Yongxin Optical Co., Ltd; Carbon dioxide cell incubator (MCO-18AC), Phcbi; Electric constant temperature water bath pot (HWS-24), Shanghai Yiheng Technology Co., Ltd; Multifunctional microplate reader (Multiskan MK3), Thermo; One ten thousandth balance (MS105DU), METTLER; Centrifuge (TD4N), Changsha Yingtai Instrument Co., Ltd; Mixer (SCI-VS), Selo Czech Republic, USA.

IV Preparation of sample

The samples were dissolved in the cell grade DMSO after accurately weighing to generate the 10 mM drug solution. The drug solution was further diluted to tenfold detection concentration by cell culture medium.

V Standard Operation Procedure

1.Detection principle of cytotoxicity (CCK-8 method)：

The detection principle is that the CCK-8 reagent contains WST-8, which is reduced to a highly water-soluble yellow methyl product (formazan) by dehydrogenase in the cell mitochondria under the action of electron carrier 1-methoxy-5-methylphenazine dimethyl sulfate (1-methoxy PMS). The number of the nail products produced is directly proportional to the number of living cells.

2.Experimental method：

(1) Inoculate cells: The cells were prepared into single cell suspension with the culture medium containing 10% fetal bovine serum, and the 96 well plates were inoculated with 90 μL cell culture medium (Adherent cell viewed 5×104/mL and Suspension cell viewed 9×104/mL) per well, then cultured at 5% CO_2 and 37 °C for 24 hours.

(2) Add the sample solution to be tested: Add 10μL sample solution to each well. One concentration was set for each sample during preliminary screening and three multiple holes were set for each concentration. Eight concentration gradients were set for each sample for IC50 determination and three multiple holes were set for each concentration. The 96 well plates were cultured at 5 % CO_2 and 37 °C for 48 hours. The experiment was divided into blank group, control group and drug group.

(3) Color development: The old culture medium and drug solution of adherent cells was sucked out, then 100 μL of CCK-8 solution (diluted ten times with the basic medium) was added and the suspension cells was directly added 10 μL of CCK-8 stock solution. Culture at 37 °C with 5% CO_2 for 1-4 h (dark operation, real-time observation).

(4) Result detection: The absorbance was measured at 450 nm with an enzyme labeling instrument and the original data and results were recorded.

(5) The toxicity is expressed by cell inhibition, and the calculation formula is as follow:

Cell inhibition (%) =(ODControl-ODDrug)/(ODControl-ODBlank)×100%. The IC50 was calculated by software graphpad prism 8 (version 8.0.2, from GraphPad Software Inc)， and the experimental results are expressed in ± SD.

(6) Positive control: Doxorubicin hydrochloride.

28. Identification of the strain OUCMDZ-5210

GenBank access number: MK393941.1
https://www.ncbi.nlm.nih.gov/nuccore/1556656878

29. Characterization of compounds

Fumitremorgin C (**1**). White amorphous powder; Chemical formula: $C_{22}H_{25}N_3O_3$; ESI-MS: *m/z* 380.2 $[M+H]^+$; $[\alpha]_D^{31}$ –79.2 (*c* 0.1, MeOH); UV(MeOH) $\lambda_{max}(log\varepsilon)$ 224.7 (1.42) nm, 272.1 (0.38) nm, 297.0 (0.41) nm; for ^{1}H NMR and ^{13}C NMR data, see Table S1.

Fumitremorgin P (**2**). Pale-green amorphous powder; Chemical formula: $C_{22}H_{23}N_3O_3$; HRESIMS: *m/z* 378.1805 $[M+H]^+$ (calacd for $C_{22}H_{24}N_3O_3{}^+$); $[\alpha]_D^{31}$ +64.2 (*c* 0.1, MeOH); UV(MeOH)$\lambda_{max}(log\varepsilon)$ 203.5 (2.2) nm, 235.0 (2.0) nm, 266.5 (1.4) nm, 299.5 (1.1) nm, 376.5 (1.1) nm; ECD (MeOH, 0.13 mM) $\lambda_{max}(\Delta\varepsilon)$ 215.0 (+9.1) nm, 262.5 (–1.3) nm, 272.5 (+0.7) nm, 300.0 (–2.3) nm, 366.0 (+2.7) nm; IR(KBr) ν_{max} 3396, 2962, 2926, 1680, 1607, 1450, 1381, 1261, 1031, 803 cm^{-1}; for ^{1}H NMR and ^{13}C NMR data, see Table S2.

Spirotryprostatin G (**3**). White amorphous powder; Chemical formula: $C_{22}H_{23}N_3O_4$; ESI-MS: *m/z* 394.6 $[M+H]^+$; $[\alpha]_D^{31}$ –15.7(*c* 0.1, MeOH); UV(MeOH) $\lambda_{max}(log\varepsilon)$ 229.1(1.45) nm, 293.1(0.85) nm; for ^{1}H NMR and ^{13}C NMR data, see Table S3.

Secofumitremorgins C/D (**4a**/**4b**). yellow oily; Chemical formula: $C_{22}H_{23}N_3O_4$; HRESIMS: *m/z* 394.1761 $[M+H]^+$ (calacd for $C_{22}H_{24}N_3O_4^+$); $[\alpha]_D^{31}$ –24.2 (*c* 0.1, MeOH); UV(MeOH) $\lambda_{max}(log\varepsilon)$ 214.0 (0.8) nm, 283.0 (0.9) nm, 344.5 (0.4) nm; ECD (MeOH, 0.13 mM) $\lambda_{max}(\Delta\varepsilon)$ 202.5 (+6.4) nm, 230.5 (–1.0) nm, 242.5 (–0.8) nm, 279.5 (–5.1) nm, 319.5 (+0.4) nm; IR(KBr) ν_{max}3107, 1737, 1680, 1629, 1423, 1365, 1274, 1201, 752 cm^{-1}; for ^1H NMR and ^{13}C NMR data, see Table S4.

Secofumitremorgins A/B (**5a**/**5b**). yellow oily; Chemical formula: $C_{23}H_{26}N_3O_4$; ESI-MS: *m/z* 408.2 $[M+H]^+$; $[\alpha]_D^{31}$ –34.2 (*c* 0.1, MeOH); UV(MeOH) $\lambda_{max}(log\varepsilon)$ 224.0 (0.8) nm, 293.0 (0.9) nm, 341.5 (0.4) nm; ECD (MeOH, 0.13 mM) $\lambda_{max}(\Delta\varepsilon)$ 212.5 (+6.4) nm, 230.5 (–1.0) nm, 242.5 (–0.8) nm, 279.5 (–5.1) nm, 323.5 (+0.4) nm; IR(KBr) ν_{max}3107, 1737, 1680, 1629, 1423, 1365, 1274, 1201, 752 cm^{-1}; for ^1H NMR and ^{13}C NMR data, see Table S5.

7a-Dihydroxyfumitremorgin C (**6**). White amorphous powder; Chemical formula: $C_{21}H_{21}N_3O_3$; ESI-MS: *m/z* 378.8 $[M+H–H_2O]^+$; $[\alpha]_D^{31}$ +19.8 (*c* 0.1, MeOH); UV(MeOH) $\lambda_{max}(log\varepsilon)$ 224.7 (1.66) nm, 271.9 (0.45) nm, 292.0 (0.48) nm; for ^1H NMR and ^{13}C NMR data, see Table S6.

7a,8-Dihydroxyfumitremorgin C (**7**). White amorphous powder; Chemical formula: $C_{21}H_{23}N_3O_4$; ESI-MS: *m/z* 394.8 $[M+H-H_2O]^+$; $[\alpha]_D^{31}$ +12.3 (*c* 0.1, MeOH); UV(MeOH) $\lambda_{max}(log\varepsilon)$ 224.5 (1.47) nm, 270.5 (0.32) nm, 291.6 (0.35) nm; for ^1H NMR and ^{13}C NMR data, see Table S7.

Fumitremorgin B (**8**). White amorphous powder; Chemical formula: $C_{27}H_{33}N_3O_5$; ESI-MS: *m/z* 462.5 $[M+H-H_2O]^+$; $[\alpha]_D^{31}$ +17.1 (*c* 0.1, MeOH); UV(MeOH)$\lambda_{max}(log\varepsilon)$ 222.3 (1.45) nm, 273.1 (0.35) nm, 289.2 (0.47) nm; for ^1H NMR and ^{13}C NMR data, see Table S8.

Secofumitremorgins E/F (**9a**/**9b**). Pink oily; Chemical formula: $C_{27}H_{31}N_3O_4$; HRESIMS: *m/z* 462.2386 $[M+H]^+$ (calacd for $C_{27}H_{32}N_3O_4^+$); $[\alpha]_D^{31}$ –16.6 (*c* 0.1, MeOH); UV(MeOH)$\lambda_{max}(log\varepsilon)$ 199.5 (1.6) nm, 282.0 (1.0) nm, 345.5 (0.3) nm; ECD (MeOH, 0.11 mM) $\lambda_{max}(\Delta\varepsilon)$ 214.5 (+4.7) nm, 233.0 (–3.1) nm, 255.5 (–1.1) nm, 285.5 (–5.6) nm, 309.5 (+0.3) nm; IR(KBr) ν_{max} 2993, 1682, 1209, 1135, 732 cm^{-1}; for ^1H NMR and ^{13}C NMR data, see Table S9.

Secofumitremorgins G/H (**10a**/**10b**). Pink oily; Chemical formula: $C_{28}H_{33}N_3O_4$; HRESIMS: *m/z* 476.2543 $[M+H]^+$ (calacd for $C_{28}H_{34}N_3O_4^+$); $[\alpha]_D^{31}$ –16.6 (*c* 0.1, MeOH); UV(MeOH) $\lambda_{max}(log\varepsilon)$ 191.5 (1.4) nm, 283.0 (1.0) nm, 348.0 (0.3) nm; ECD (MeOH, 0.10 mM) $\lambda_{max}(\Delta\varepsilon)$ 201.5 (+3.9) nm, 240.5 (–3.4) nm, 262.5 (–0.5) nm, 290.0 (–2.4) nm, 311.5 (+0.4) nm; IR(KBr)ν_{max} 1683, 1450, 1397, 1209, 1124, 798 cm^{-1}; for ^1H NMR and ^{13}C NMR data, see Table S10.

Demethoxyfumitremorgin C (**11**). White amorphous powder; Chemical formula: $C_{20}H_{18}O_2N_3$; ESI-MS: *m/z* 350.2 $[M+H]^+$; $[\alpha]_D^{31}$ –79.0 (*c* 0.1, MeOH); UV(MeOH) $\lambda_{max}(log\varepsilon)$ 222.2 (1.48) nm, 272.6 (0.42) nm; for ^1H NMR and ^{13}C NMR data, see Table S11.

Demethoxyfumitremorgin P (**12**). Pale-green amorphous powder; Chemical formula: $C_{21}H_{21}N_3O_2$; ESI-MS: *m/z* 348.2 $[M+H]^+$ (calacd for $C_{21}H_{22}N_3O_2^+$); $[\alpha]_D^{31}$ +64.2 (*c* 0.1, MeOH); UV(MeOH) $\lambda_{max}(log\varepsilon)$ 201.5(1.6) nm, 236.3 (2.4) nm, 260.3 (1.2) nm, 282.3 (1.1) nm, 368.3 (1.2) nm; for ^1H NMR and ^{13}C NMR data, see Table S12.

Spirotryprostatin B (**13**). White amorphous powder; Chemical formula: $C_{21}H_{24}ON_3$; ESI-MS: *m/z* 364.2 $[M+H]^+$; $[\alpha]_D^{31}$ –15.7 (*c* 0.1, MeOH); UV(MeOH) $\lambda_{max}(log\varepsilon)$ 229.2 (1.52) nm, 294.1 (1.05) nm; for ^1H NMR and ^{13}C NMR data, see Table S13.

7a-Hydroxy demethoxyfumitremorgin C (**15**). White amorphous powder; Chemical formula: $C_{21}H_{23}N_3O_3$; ESI-MS: *m/z* 348.9 $[M+H–H_2O]^+$; $[\alpha]_D^{31}$ +16.0 (c 0.1, MeOH); UV(MeOH) $\lambda_{max}(log\varepsilon)$ 222.0 (1.48) nm, 271.9 (0.38) nm; for ^{1}H NMR and ^{13}C NMR data, see Table S14.

7a,8-Dihydroxy demethoxyfumitremorgin C (**16**). White amorphous powder; Chemical formula: $C_{21}H_{23}N_3O_4$; ESI-MS: *m/z* 364.8 $[M+H–H_2O]^+$; $[\alpha]_D^{31}$ +9.6 (c 0.1, MeOH); UV(MeOH) $\lambda_{max}(log\varepsilon)$ 222.3 (1.55) nm, 280.0 (0.43) nm; for ^{1}H NMR and ^{13}C NMR data, see Table S15.

Spirotryprostatin A (**17**). White amorphous powder; Chemical formula: $C_{22}H_{26}N_3O_4$; ESI-MS: *m/z* 396.5 $[M+H–H_2O]^+$; $[\alpha]_D^{31}$ –69.6 (c 0.1, MeOH); UV(MeOH) $\lambda_{max}(log\varepsilon)$ 220.1 (0.52) nm, 293.1 (0.35) nm; for ^{1}H NMR and ^{13}C NMR data, see Table S16.

30. Structural Analysis of New Compounds

Compound **2** is a compound obtained for the first time from a natural product. Based on HRESIMS m/z 378.1805 $[M+H^+]$ (calculated as $C_{22}H_{24}O_3N_3$), the molecular formula was suggested to be $C_{22}H_{23}O_3N_3$ with an unsaturation of 13 (Fig. S10). Detailed comparative analysis of 1D and 2D NMR data showed that compound **2** was similar to the known compound fumitremorgin C (**1**) (Figs. S5–S9). However, in contrast to compound **1**, neither C-7a nor C-8 methyl is present, but two additional sp^2 hybridized carbon signals are current. The above observations suggest compound **2** may be a derivative of compound fumitremorgin C with double bond formation between C-7a and C-8. The HMBC signals associated with H-8 to C-1a, C-7, and C-7a confirmed this inference. At this point, the planar structure of compound **2** was determined.

New Compounds **4a**/**4b** were isolated as a mixture at a ratio of 1:0.7 (major: minor). We try all my best to apply chiral columns with different packing as well as various ratio to separate these two isomers, unfortunately, it was found that no resolution could be achieved. HRESIMS gave a $[M+H^+]$ quasi molecular ion peak at *m/z* 394.1751 (calculated for $C_{22}H_{24}O_4N_3^+$), suggestive of a molecular formula $C_{22}H_{24}O_4N_3$ with 13 degrees of unsaturation (Fig. S20). The ^{1}H-NMR and ^{13}C-NMR spectra of the mixture showed that their planar structures all contain 4 methyl groups (including 1 methoxy group), 3 aliphatic methyl groups, 6 methyl groups (including 5 sp^2 hybridized and 1 connected to heteroatoms), 10 quaternary carbons (including 2 carbonyls). These indicated that **4a**/**4b** had the same planar structure. Detailed analysis and comparison of the 1D and 2D NMR data revealed that **4a**/**4b** shared the same planar structure, which is similar to the known compounds secofumitremorgins A/B (**5a**/**5b**)[3] (Figs. S14–S19). However, signals for the 4-OCH_3 ($\delta_{C/H,\ major}$ 51.6/3.67 and $\delta_{C/H,\ minor}$ 51.4/3.46) in **4a**/**4b** were absent. This indicates that **4a**/**4b** does not have 4-OCH_3. Therefore, the planar structures of compounds **4a**/**4b** were determined.

Marfey hydrolysis was used to determine the configuration of the proline residue in **4a**/**4b**. The proline residue was first partially removed from the molecule by an acid hydrolysis procedure. The hydrolyzed proline was simultaneously derivatized with D-proline and L-type proline standards. The retention times of these proline derivatives were then examined by LC-MS and HPLC with detection at λ 340 nm. The results demonstrated that the proline derivative from **4a**/**4b** had the same retention time to the L-proline standard, as shown in Fig. S63, indicating *L*-configuration of proline in **4a**/**4b**.

The primary variations between **4a** (major) and **4b** (minor) lie in the chemical shifts of proline. Specifically, compared to **4b**, **4a** exhibited larger chemical shifts at C-7, C-8, while showing smaller chemical shifts at C-5 and C-6. Given that **4a** and **4b** could not be separated on a chiral

column and the tertiary amide nature of the compounds, we proposed that **4a** and **4b** represent a pair of rotamers airing from restricted rotation around the amide single bond (C-10–N-9). Furthermore, the anisotropy effect of the amide carbonyl group tended to increase the chemical shifts of α-proton in the proline moiety. This observation suggested an *S-cis* configuration for **4a** (major) and *S-trans* configuration for **4b** (minor). NMR calculations, in conjunction with DP4+ analysis (Table S26 and S27), and the Boltzmann distribution obtained from Gibbs free energy (Table S22), were employed to validate this conlusion.

The new compounds **9a**/**9b** were also isolated as a rotamer mixture with a ratio of 1:0.7 (major: minor) and the same molecular formular of $C_{27}H_{31}O_4N_3$, according to the HRESIMS data at *m/z* 462.2386 [M+H$^+$] (calculated for $C_{27}H_{32}O_4N_3^+$) (Fig. S40). The similar ^{1}H and ^{13}C NMR spectra to **4a**/**4b**, indicating the similar structures (Figs. S35–S39). However, compounds **9a**/**9b** displayed two additional methyl groups, one methylene group, a sp^2-hybrid methine group and a sp^2-hybrid quaternary carbon. These features formed an isopentenyl group, which was attached to the N-1 atom as indicated by the HMBC correlations from H-21 to C-23. Based on the same reasoning and analytical methods, compound **9a** (minor) was deduced as *S-cis* configuration while **9b** (major) was determined to be *S-trans* configuration. NMR calculations, in conjunction with DP4+ analysis (Table S30 and S31), and the Boltzmann distribution obtained from Gibbs free energy (Table S22), were employed to validate this conlusion.

The new compounds **10a**/**10b** were likewise isolated as a rotamer mixture with a ratio of 1:0.7 (major: minor), and determined to have the molecular formula of $C_{28}H_{33}O_4N_3$, according to the HRESIMS data at *m/z* 476.2543 [M+H]$^+$ (calculated for $C_{28}H_{34}N_3O_4^+$) (Fig. S47). The ^{1}H and ^{13}C NMR spectra are very similar to compounds **9a**/**9b**, indicating the similar structures (Figs. S42–S46). However, compounds **10a**/**10b** showed additional methoxy signals, implying that compounds **10a**/**10b** could be the methyl ester of **9a**/**9b**. Like **9a**/**9b**, the configuration of **10a** (minor) and **10b** (major) were determined to be *S-cis* and *S-trans*, respectively. NMR calculations, in conjunction with DP4+ analysis (Table S32 and S33), and the Boltzmann distribution obtained from Gibbs free energy (Table S22), were employed to validate this conlusion.

Compound **12** is a known compound that was isolated from the natural source for the first time. Compound **12** displayed a MS peak at *m/z* 348 [M+H$^+$] and similar NMR data to compound **2**. However, compound **12** did not show methoxy signals ($\delta_{C/H}$ 55.7/3.84) but with additional aromatic proton signal (δ_H 7.20), indicating a 11-demethoxy derivative of compound **2**. This was confirmed by COSY from H-9 to H-12 in sequence.

Fig. S1. The hypothesis of Tsunematsu et al.

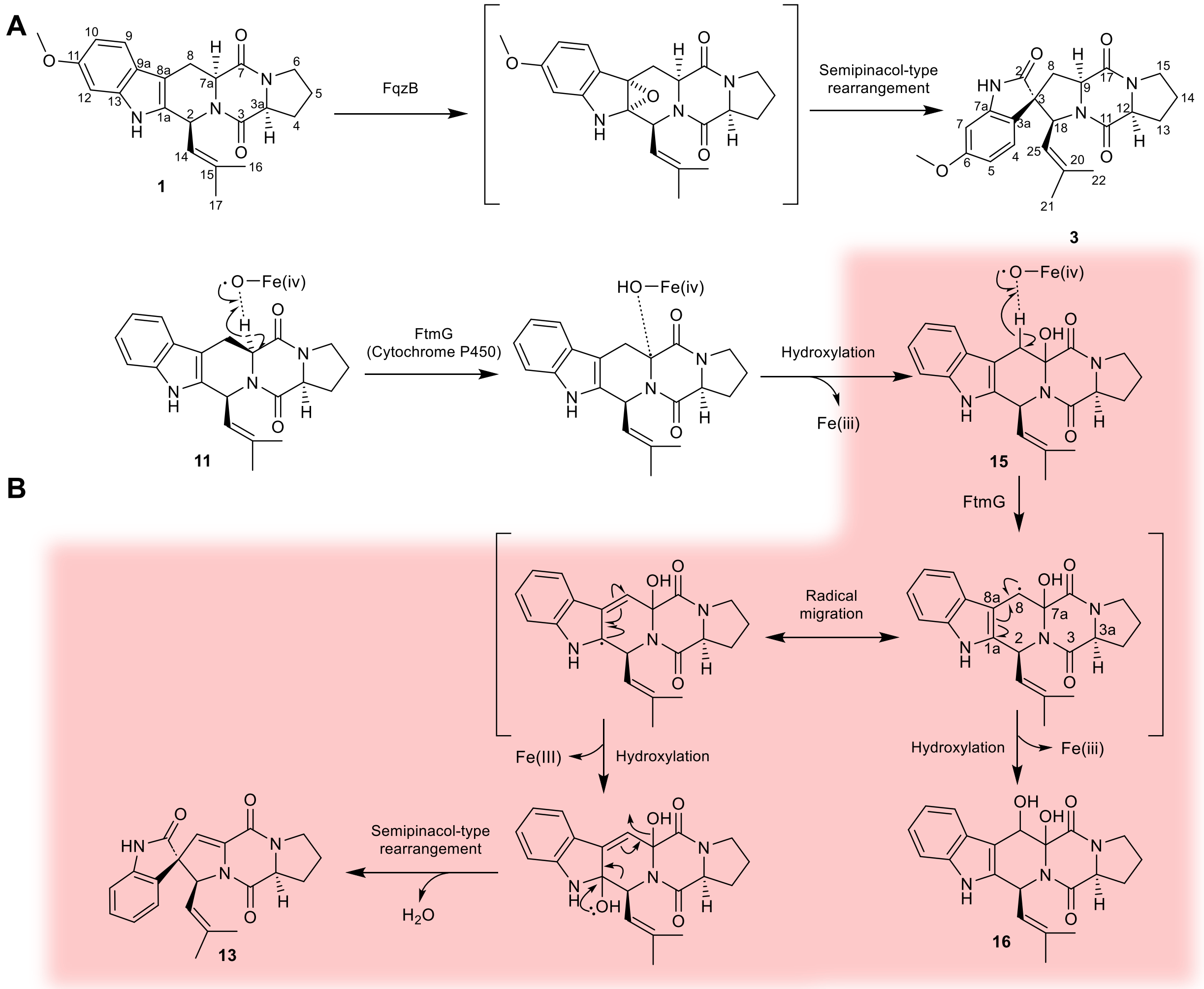

The hypothesis proposed by Tsunematsu et al[21] (A) The hypothesis was proposed based on the experimental phenomenon that **1** was incubated with the strain heterologous expressing FqzB. (B) The hypothesis was proposed based on the experimental phenomenon that **11** was incubated with the strain heterologous expressing FtmG. The red part hadn't been experimentally confirmed.

Fig. S2. Molecular networking of OUCMDZ-5210.

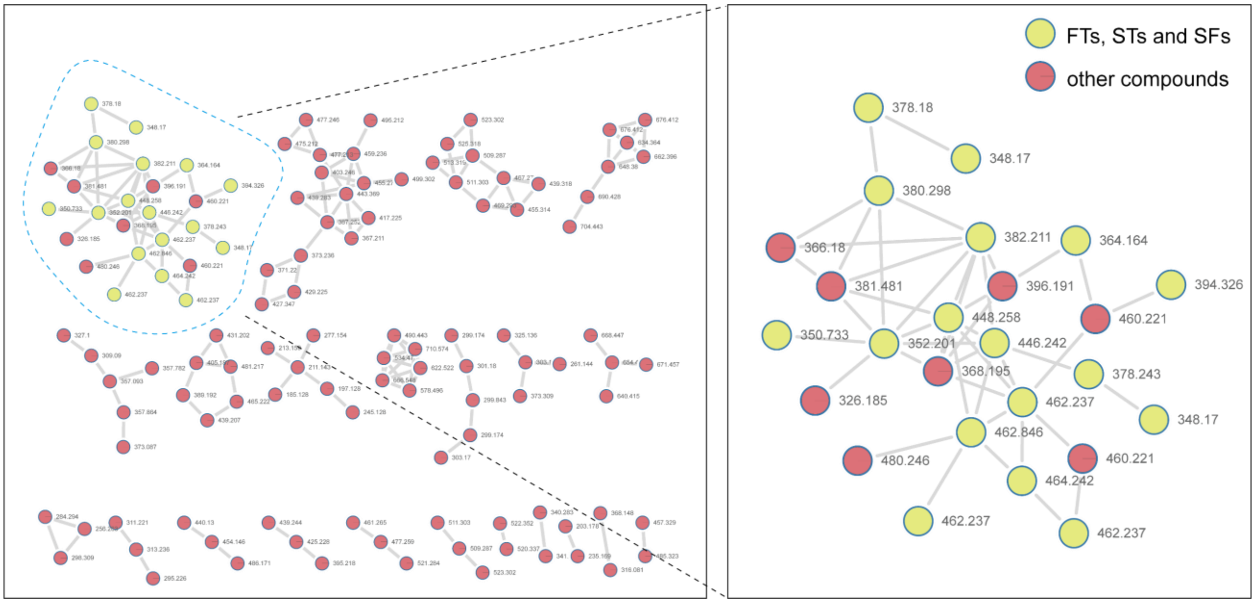

LC-MS/MS-based molecular networking of OUCMDZ-5210. Yellow highlighting corresponds to fumitremorgins (FTs), secofumitremorgins (SFs), and spirotryprostatins (STs), along with their derivatives; red denotes other compound classes.

Fig. S3. ^{1}H NMR spectrum of **1** in $CDCl_3$ (400 MHz).

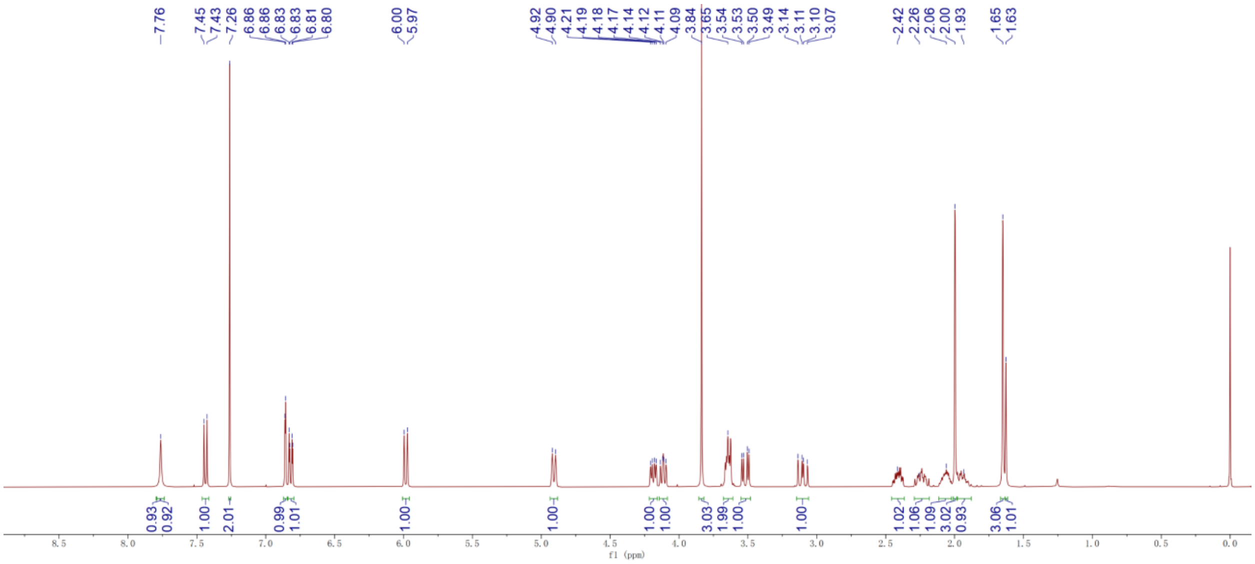

Fig. S4. ^{13}C NMR spectrum of **1** in $CDCl_3$ (100 MHz).

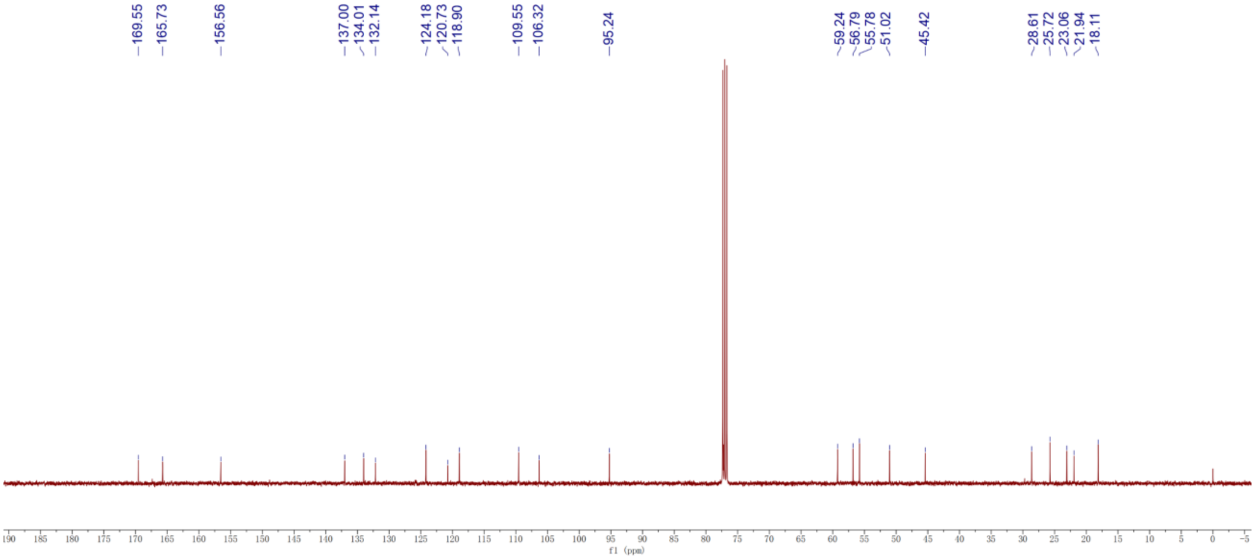

Fig. S5. ^{1}H NMR spectrum of **2** in $CDCl_3$ (400 MHz).

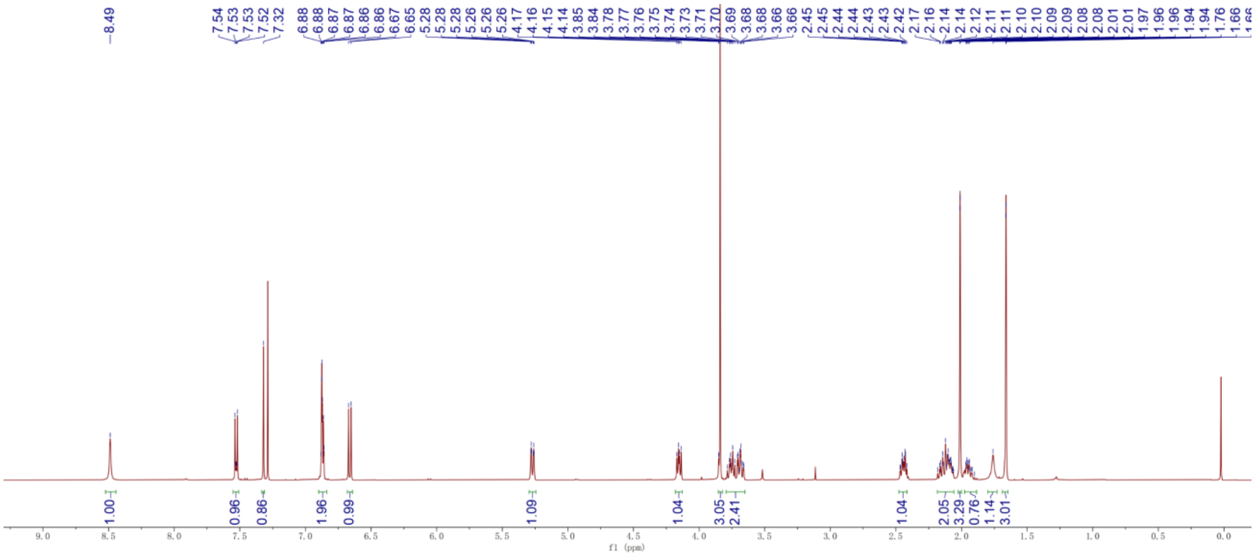

Fig. S6. ^{3}C NMR spectrum of **2** in $CDCl_3$ (100 MHz).

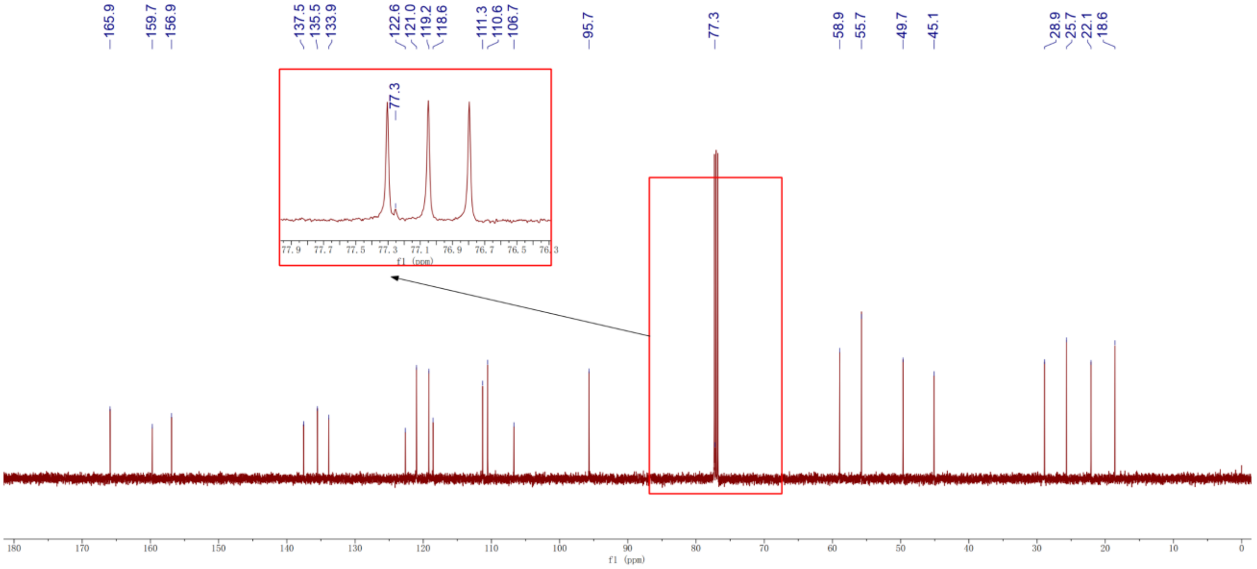

Fig. S7. COSY spectrum of **2** in $CDCl_3$ (500 MHz, 500 MHz).

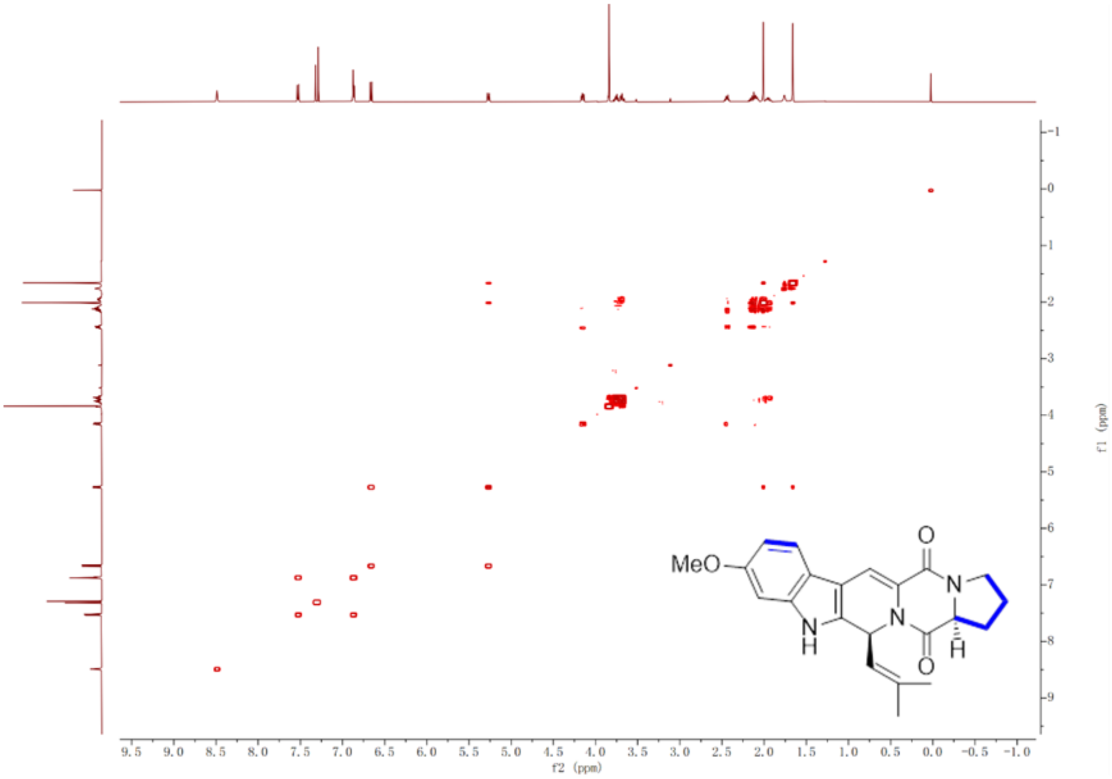

Fig. S8. HSQC spectrum of **2** in $CDCl_3$ (500 MHz, 125 MHz).

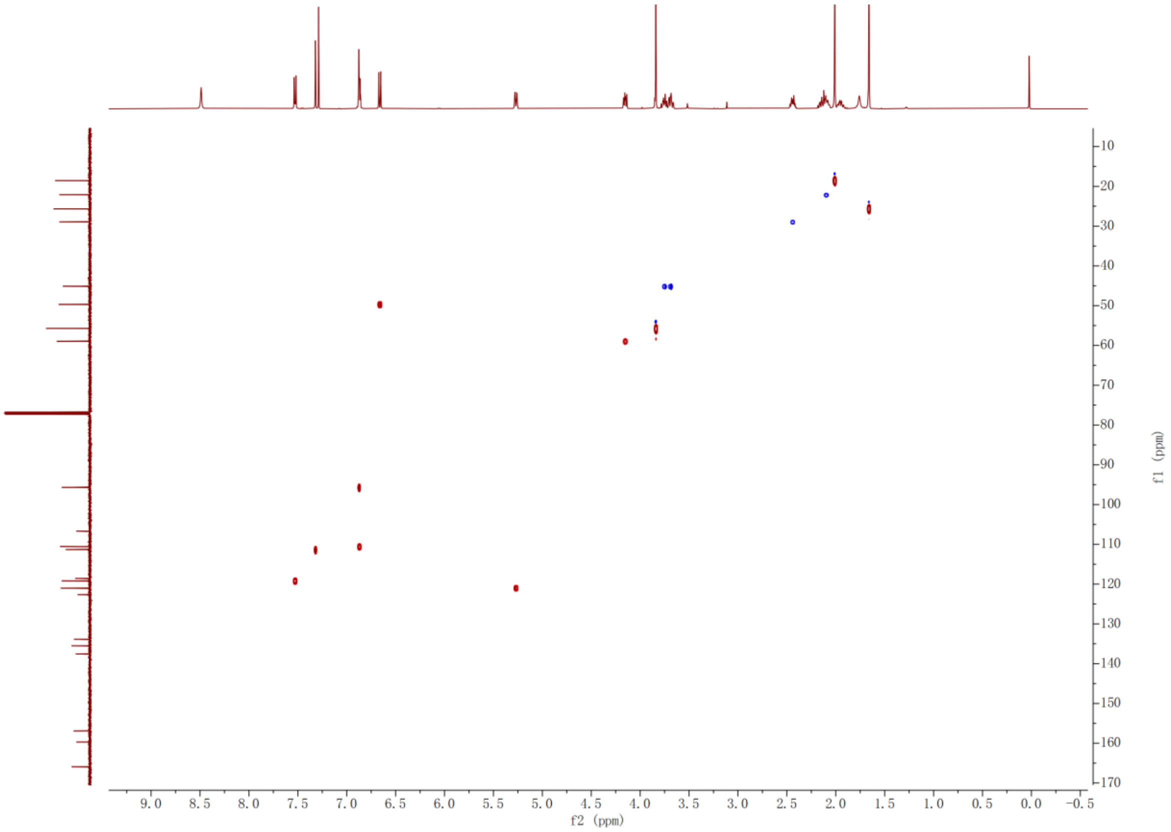

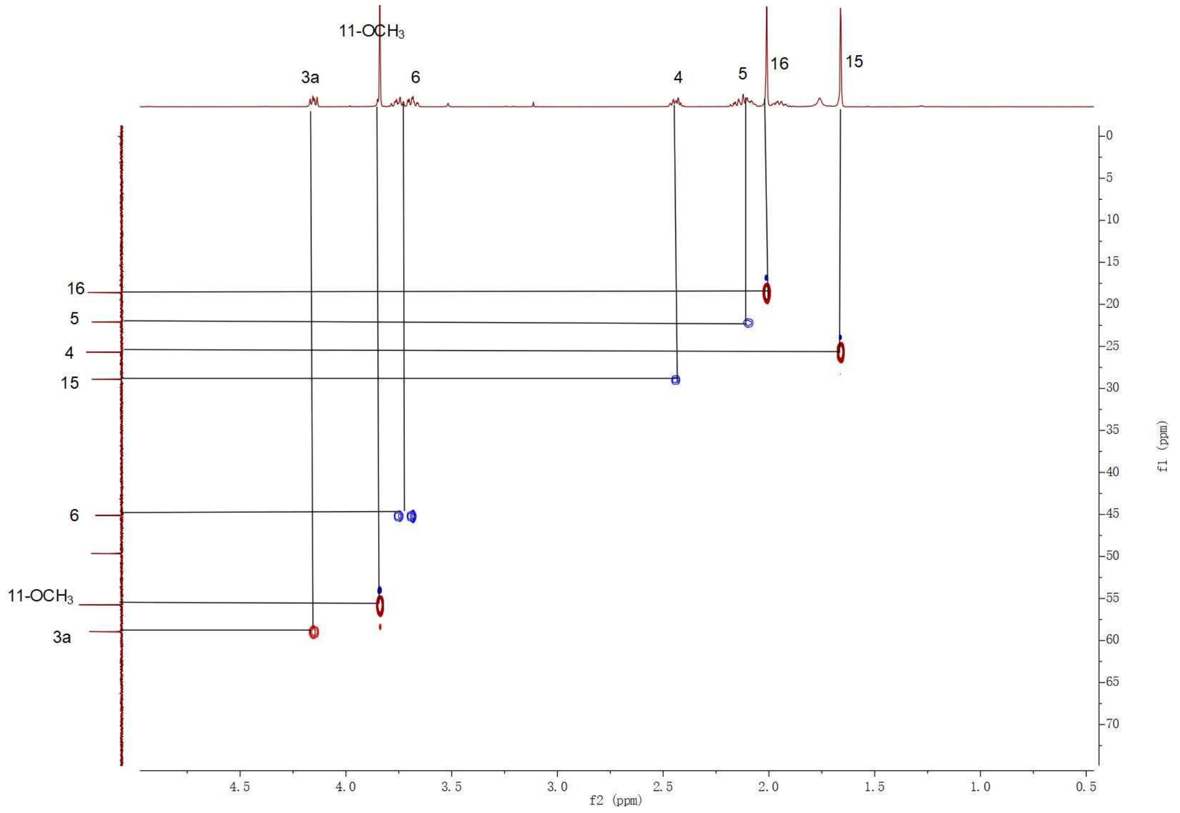

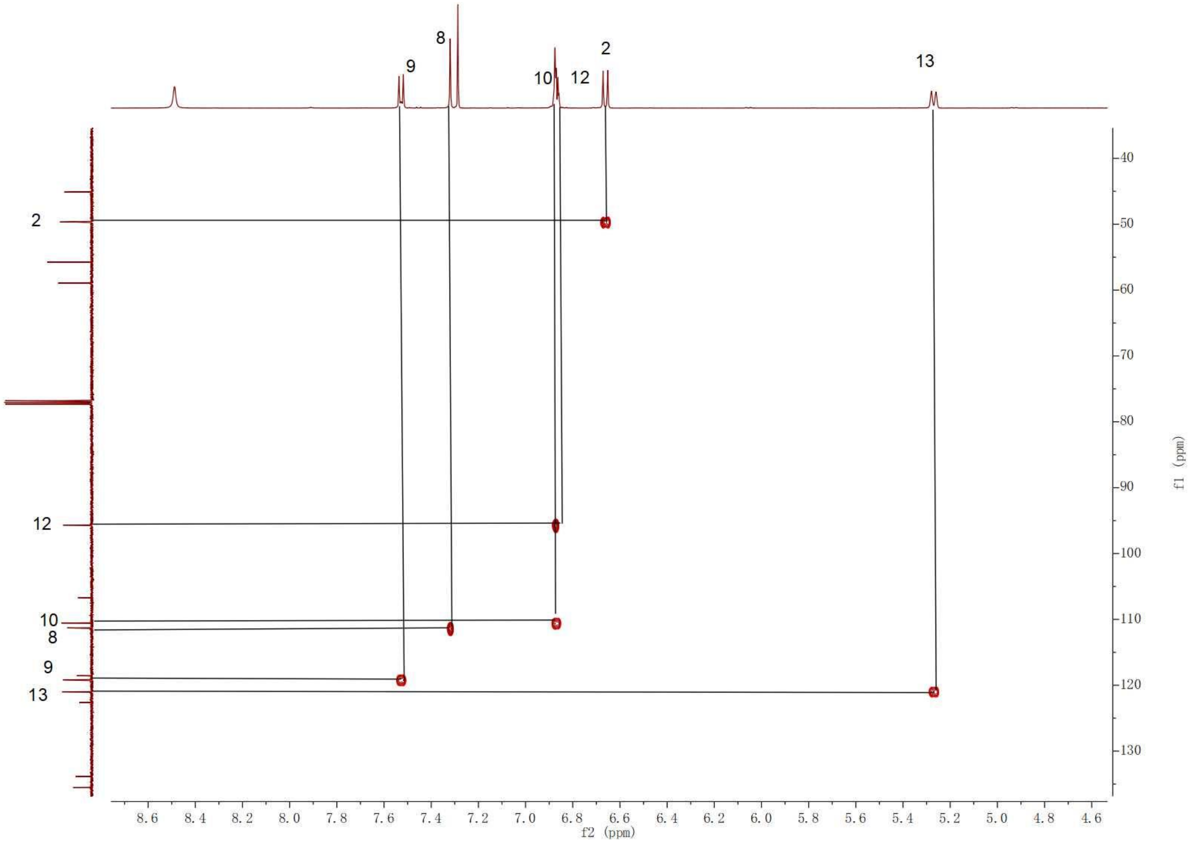

8
9
10
12
2
13
2
12
10
8
9
13
f2 (ppm)
f1 (ppm)

Fig. S9. HMBC spectrum of **2** in $CDCl_3$ (500 MHz, 125 MHz).

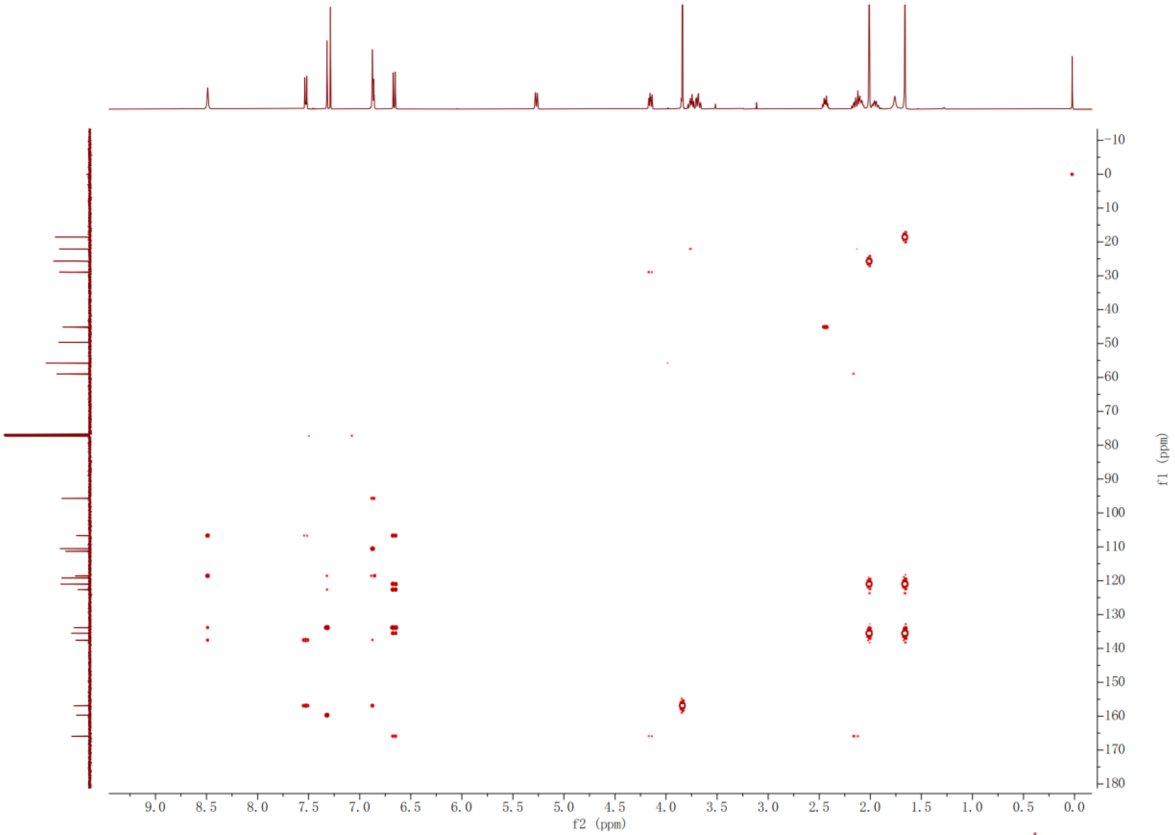

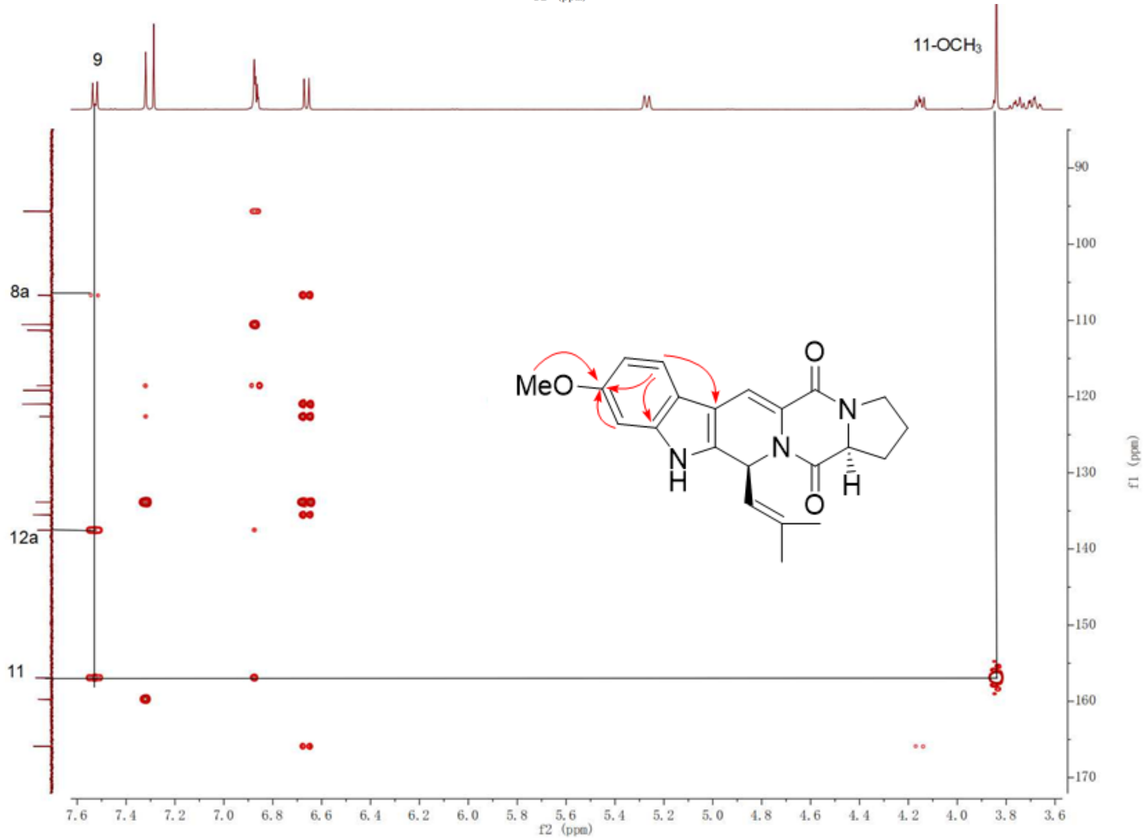

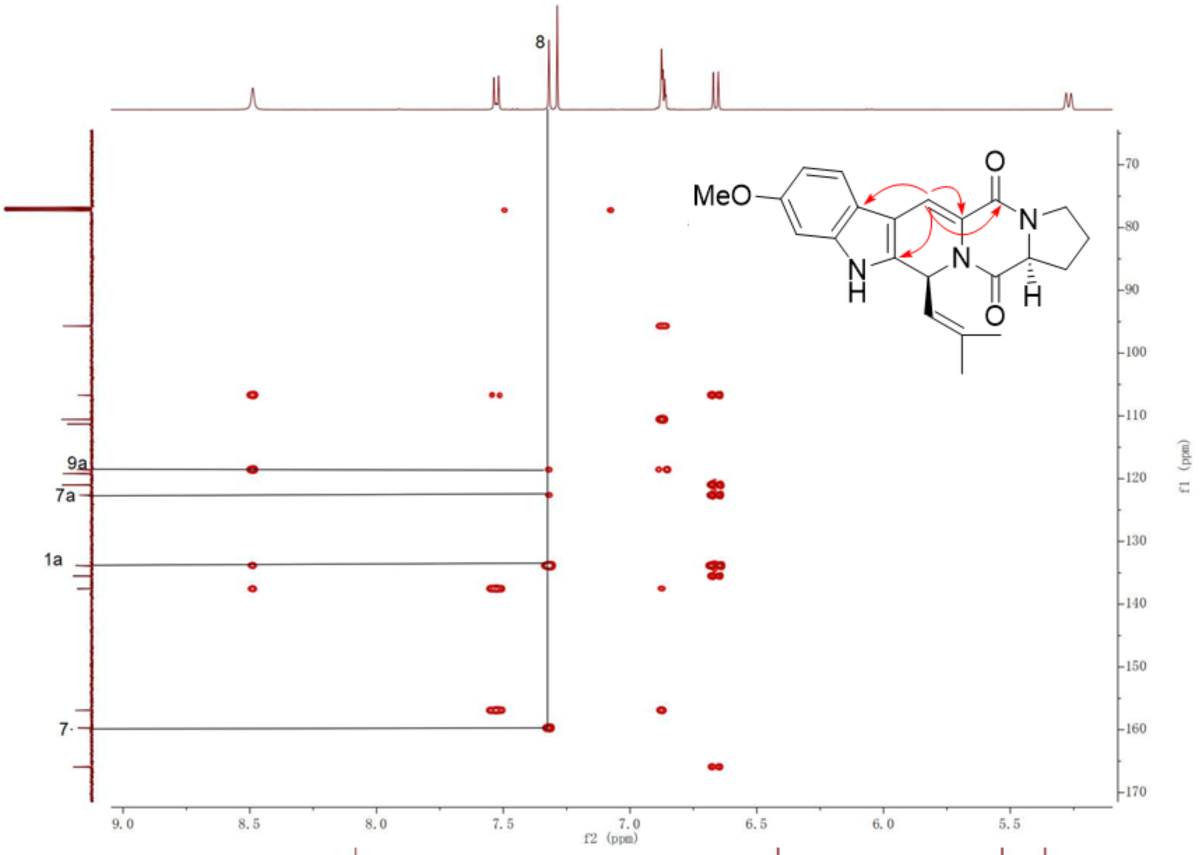
8
MeO
N
N
H
O
O
9a
7a
1a
7
f1 (ppm)
f2 (ppm)

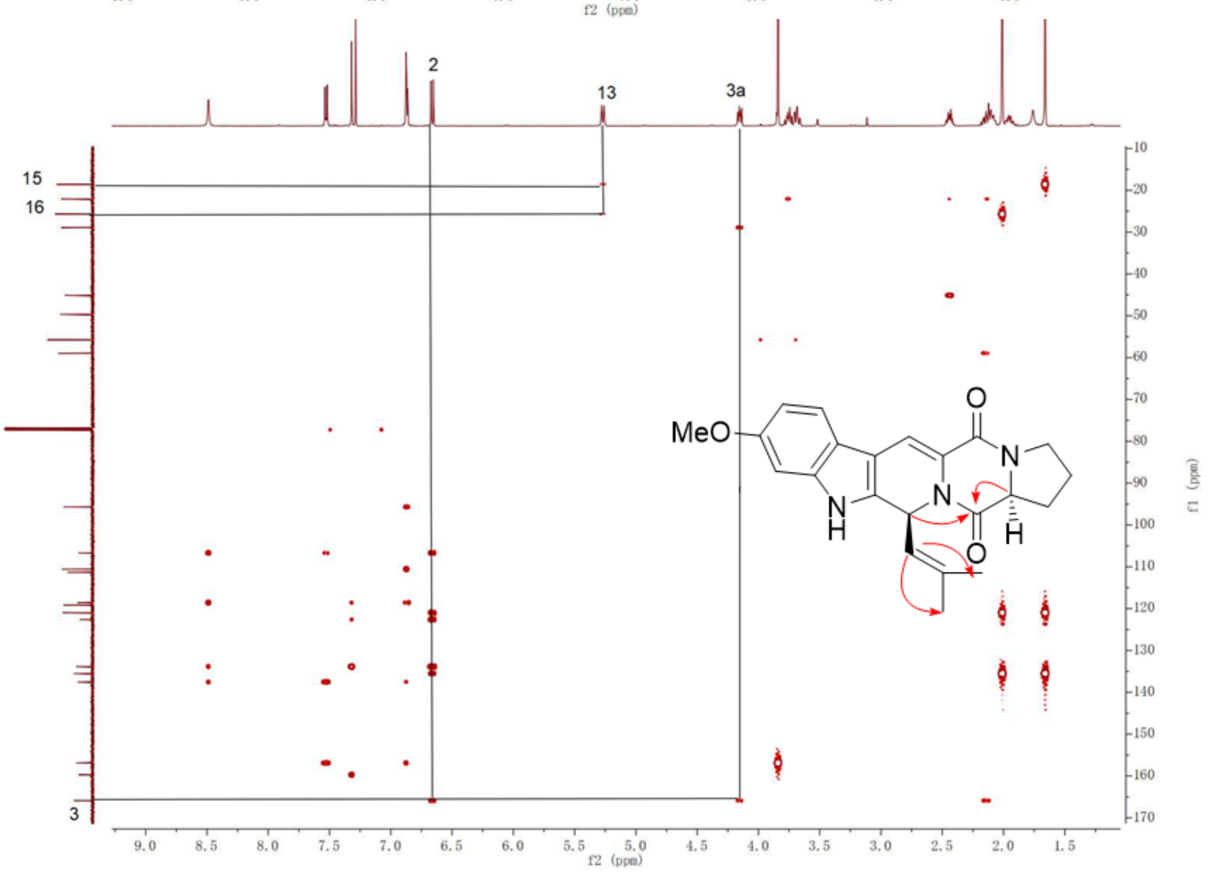
2
13
3a
15
16
3
MeO
N
N
H
O
O
f1 (ppm)
f2 (ppm)

Fig. S10. HRESIMS spectrum of **2**.

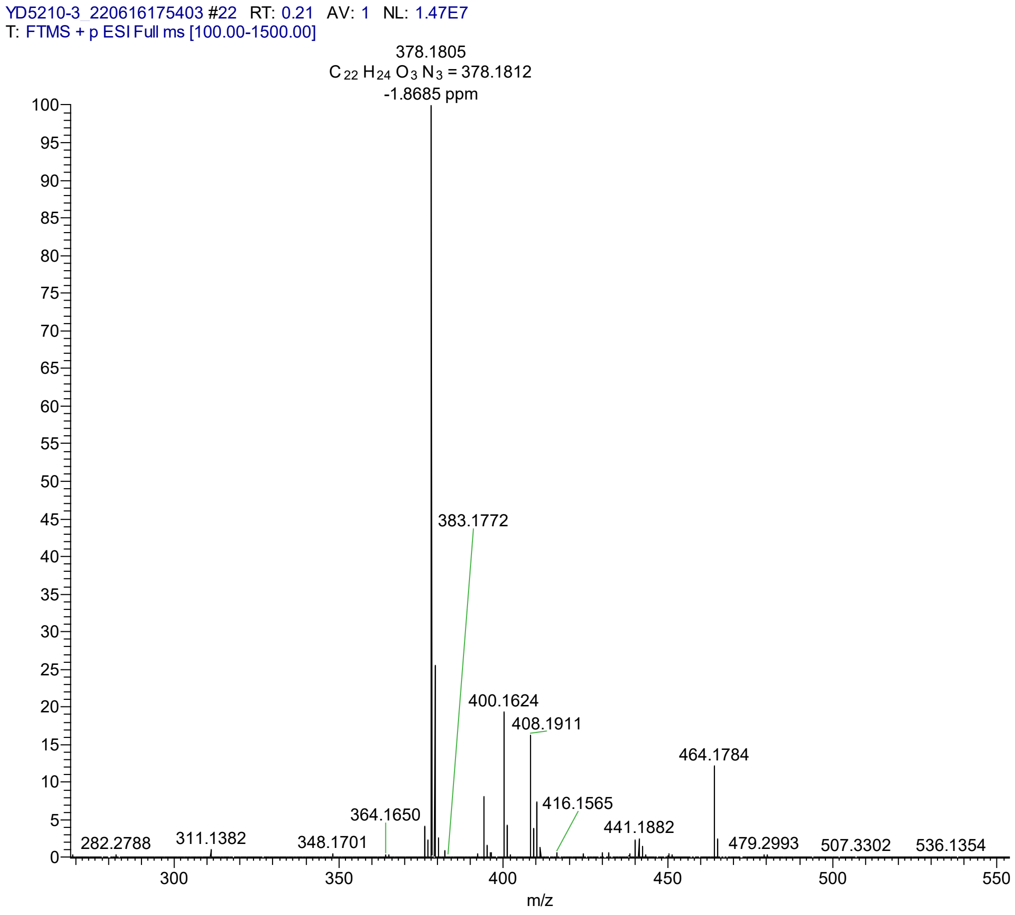

Fig. S11. IR spectrum of **2**.

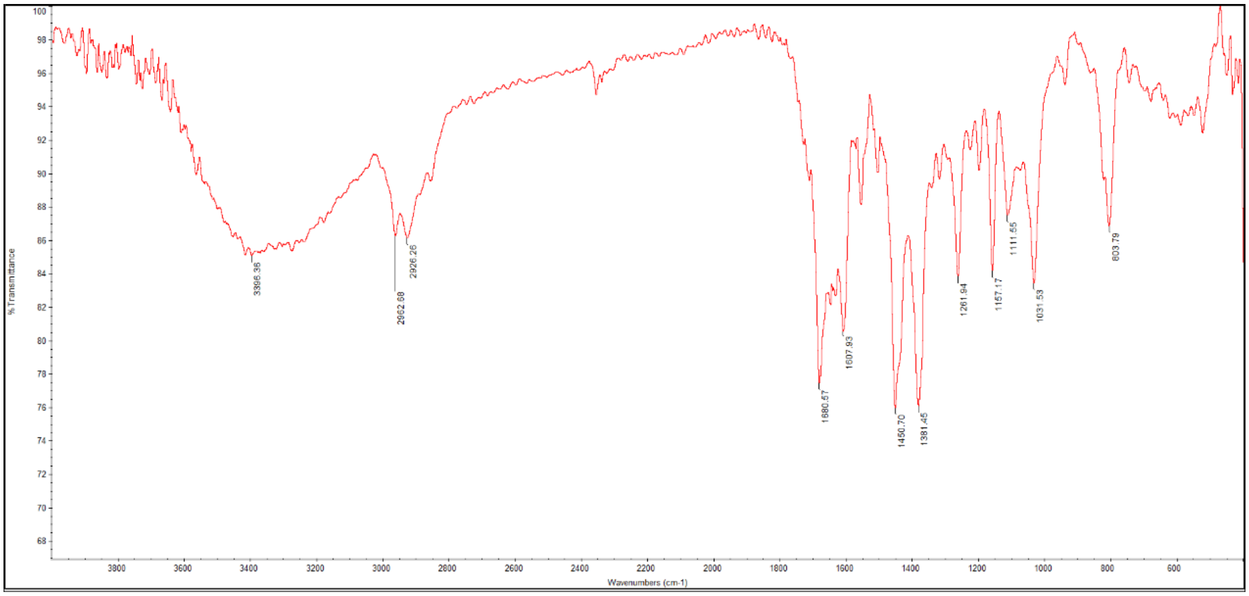

Fig. S12. ^{1}H NMR spectrum of **3** in $CDCl_3$ (400 MHz).

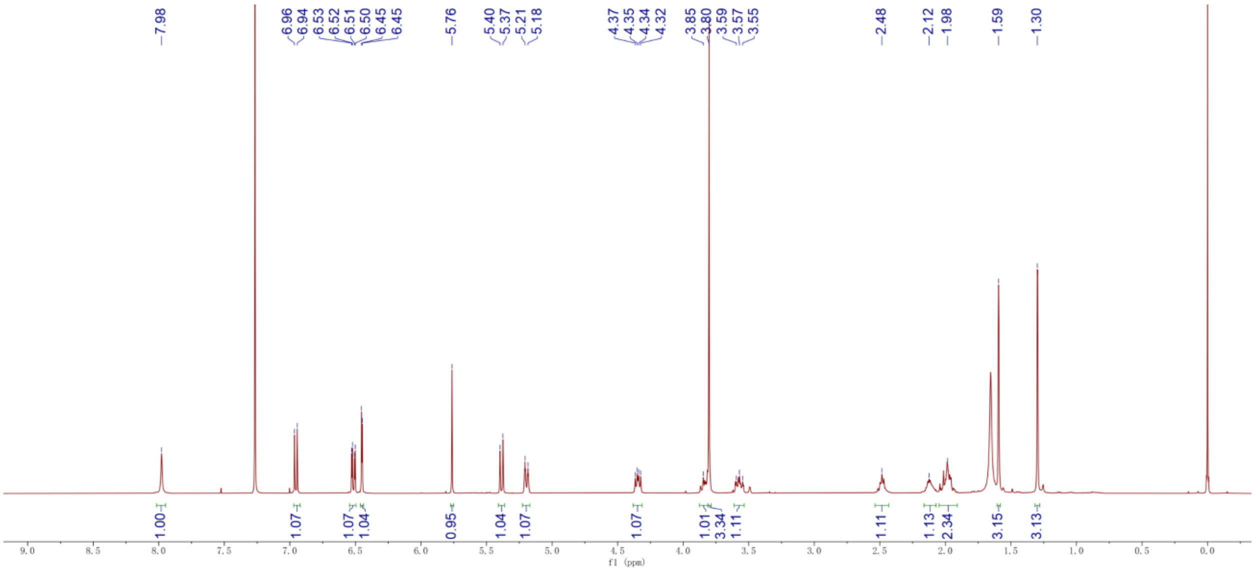

Fig. S13. ^{13}C NMR spectrum of **3** in $CDCl_3$ (100 MHz).

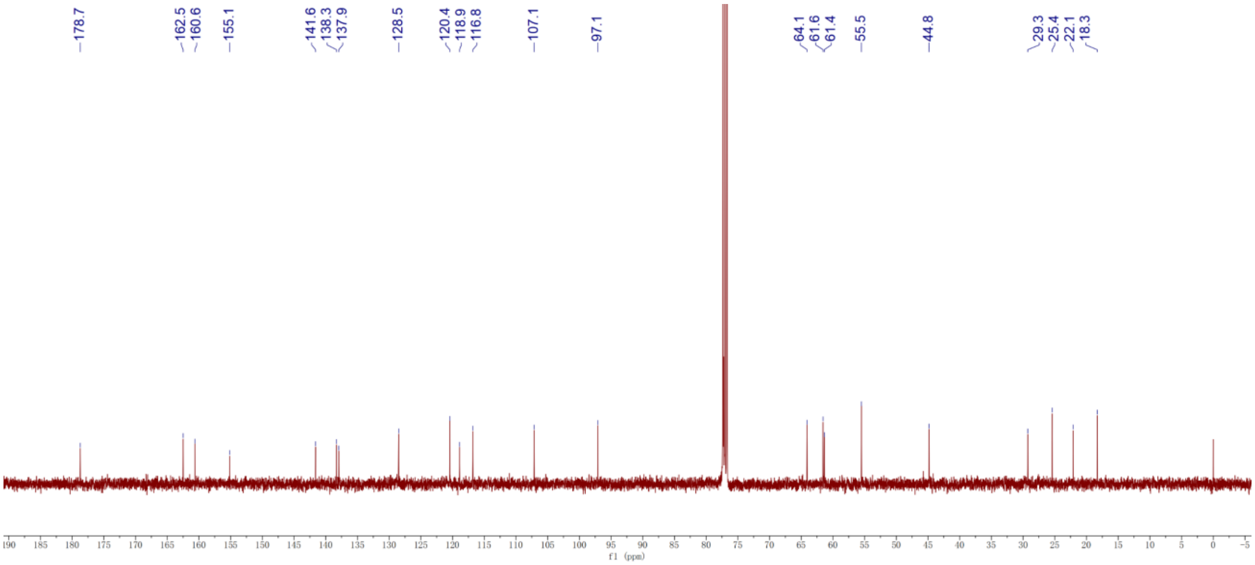

Fig. S14. ^{1}H NMR spectrum of **4** in DMSO-d_6 (400 MHz).

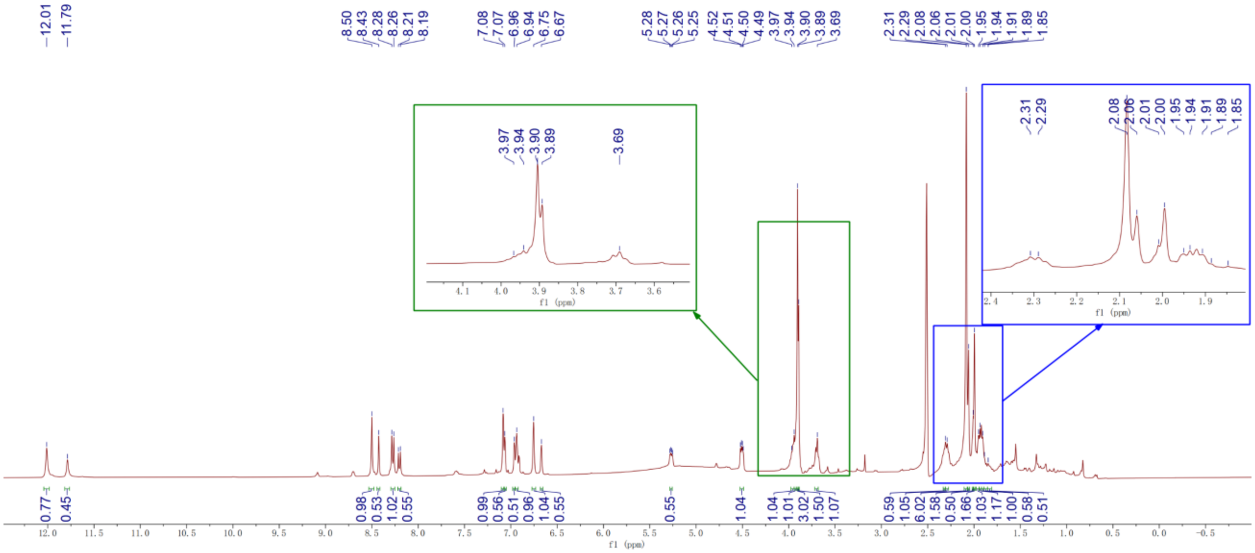

Fig. S15. ^{13}C NMR spectrum of **4** in DMSO-d_6 (100 MHz).

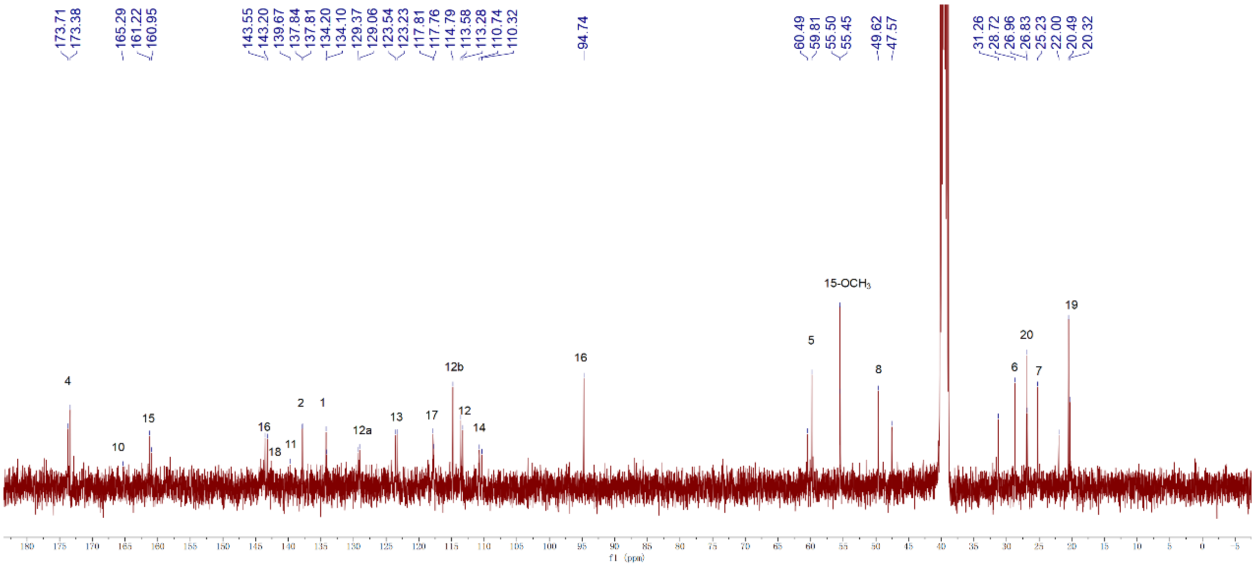

Fig. S16. COSY spectrum of **4** in DMSO-d_6 (500 MHz, 500 MHz).

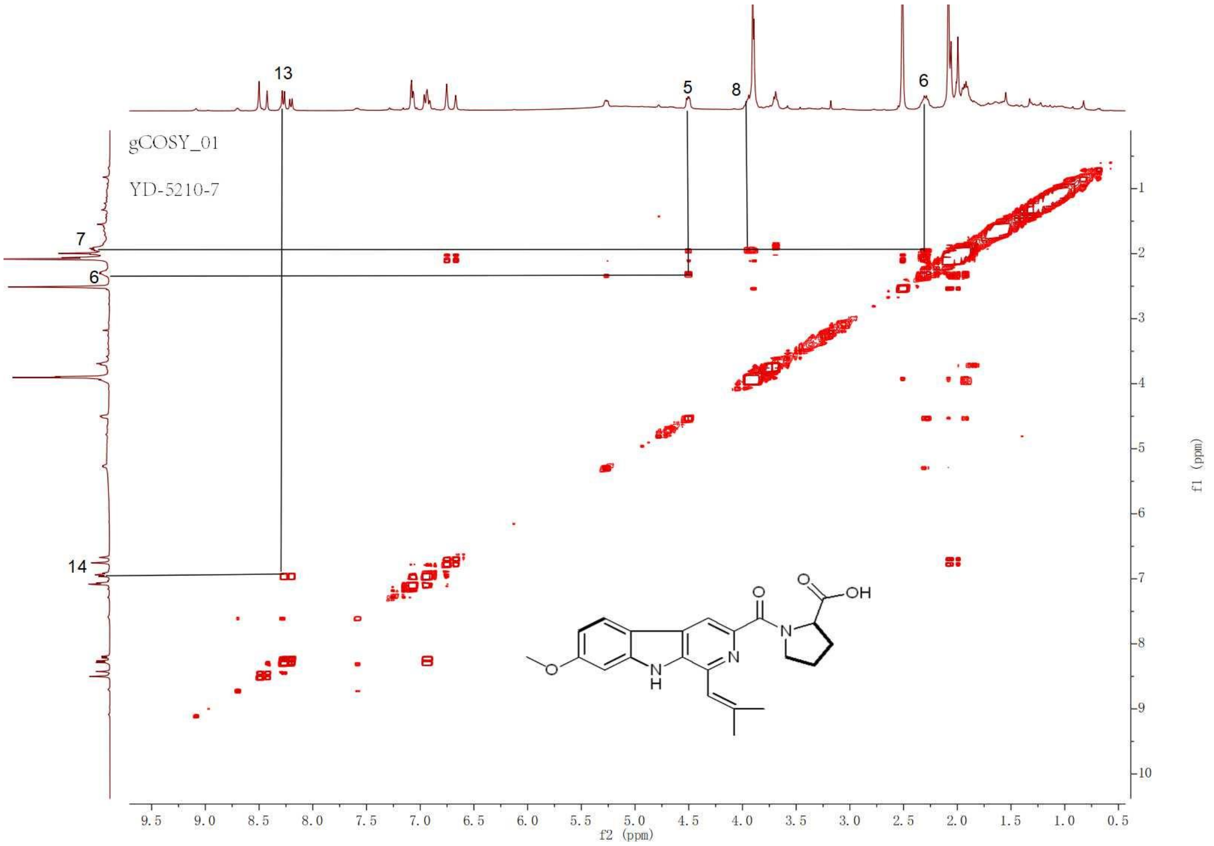

Fig. S17. HSQC spectrum of **4** in DMSO-d_6 (500 MHz, 125 MHz).

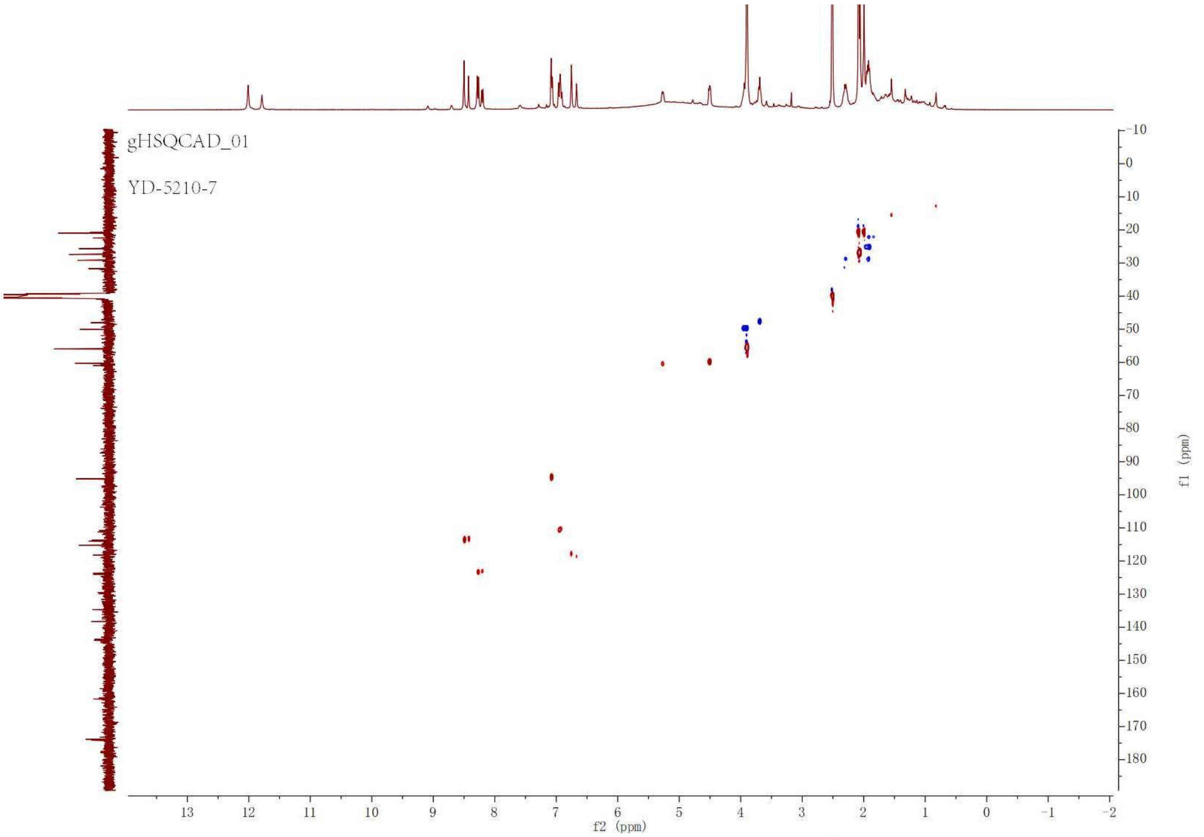

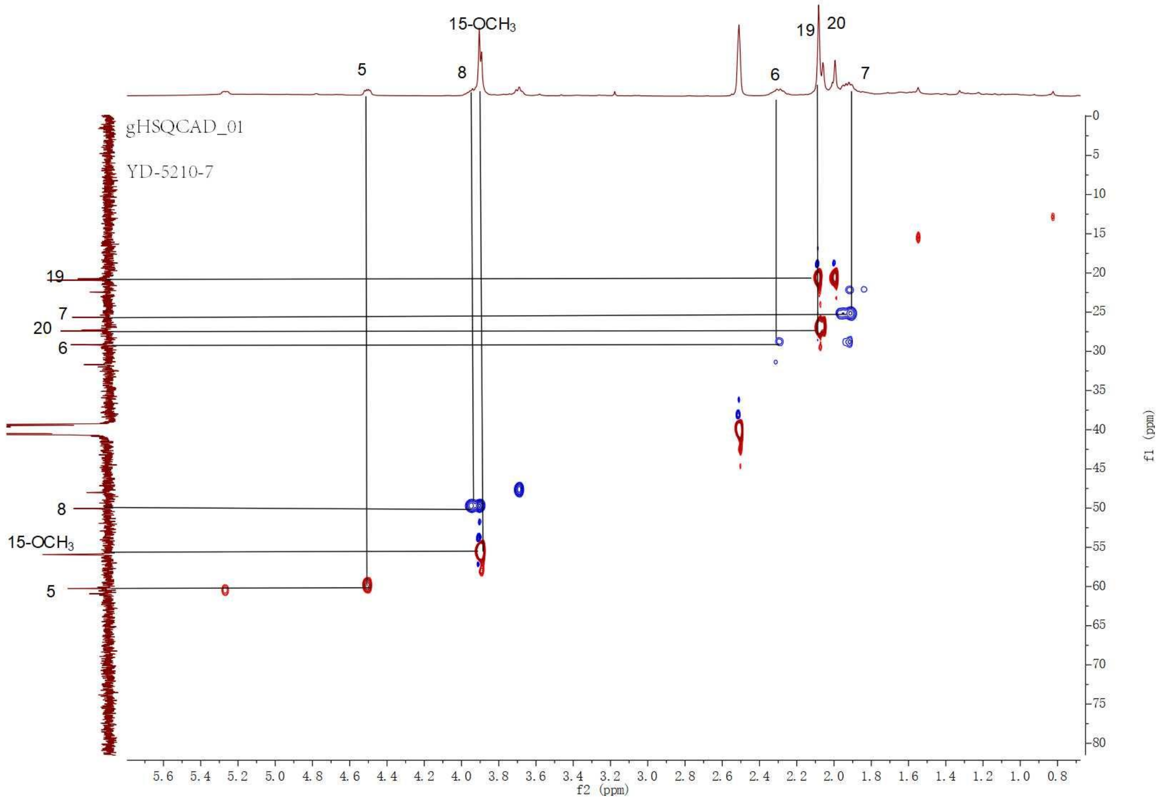

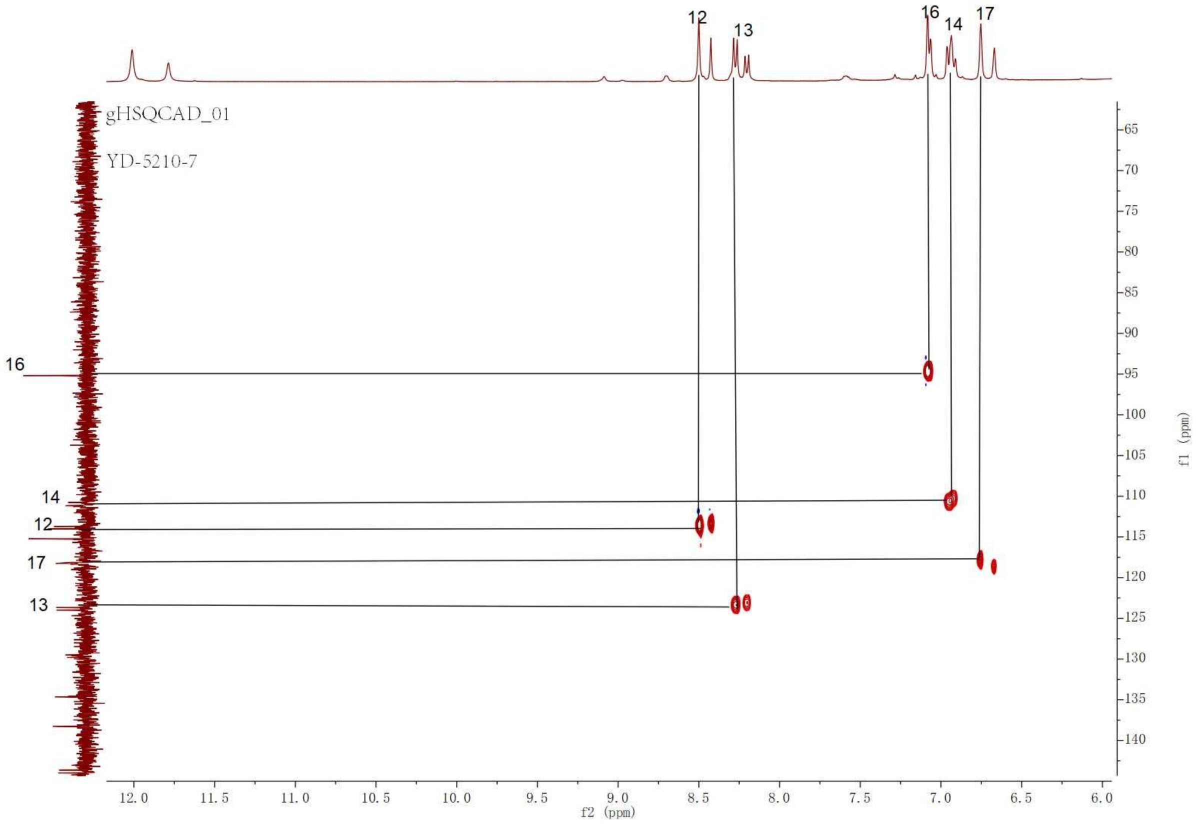
gHSQCAD_01
YD-5210-7
12
13
16
14
17
16
14
12
17
13
f1 (ppm)
f2 (ppm)

Fig. S18. HMBC spectrum of 4 in DMSO-d_6 (500 MHz, 125 MHz).

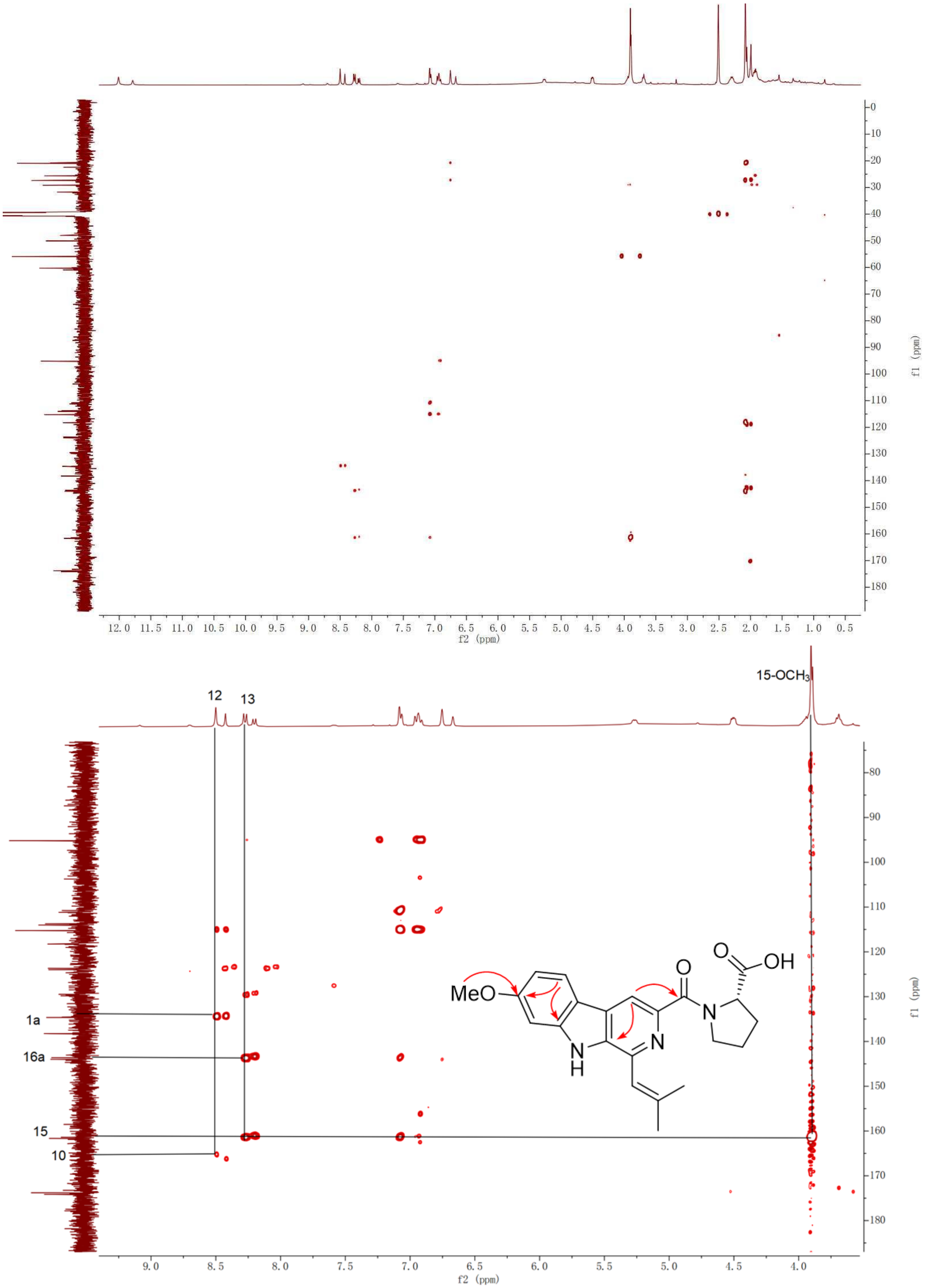

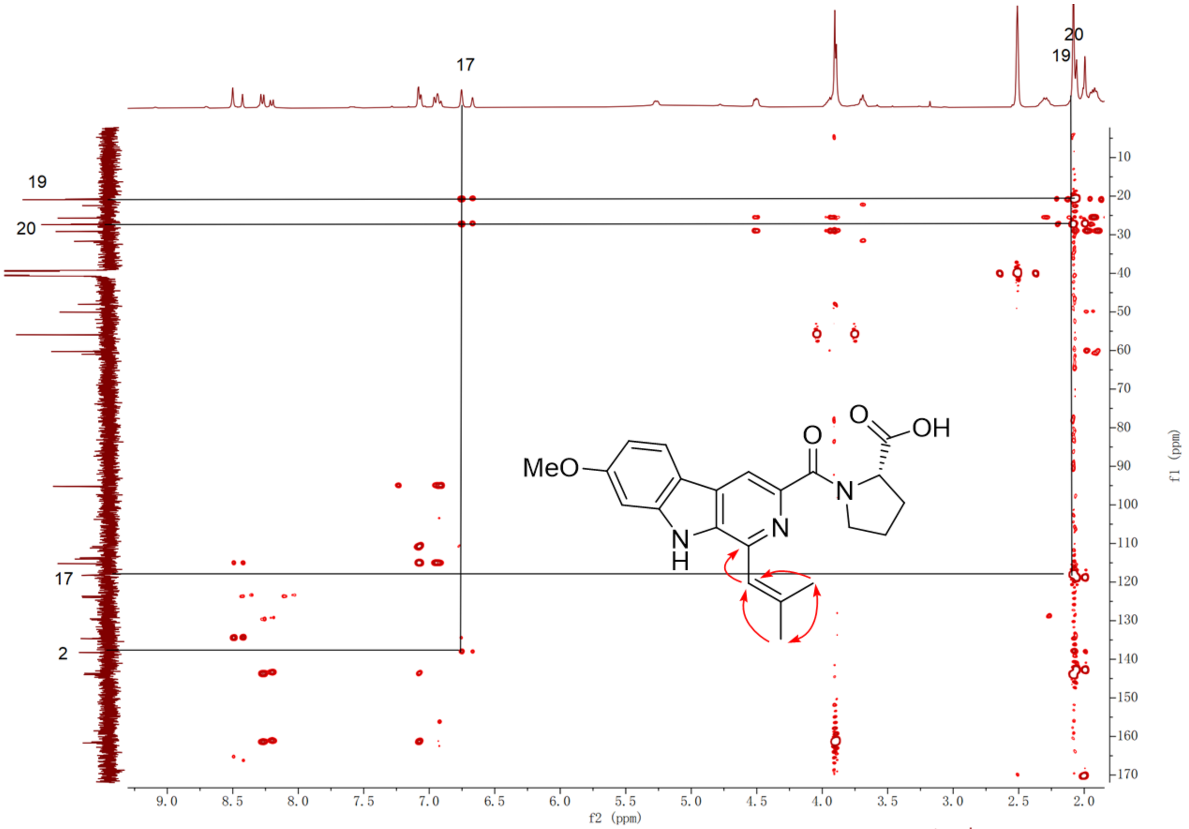
17
20
19
19
20
17
2
MeO
O
O
OH
N
N
H
N
f2 (ppm)
f1 (ppm)

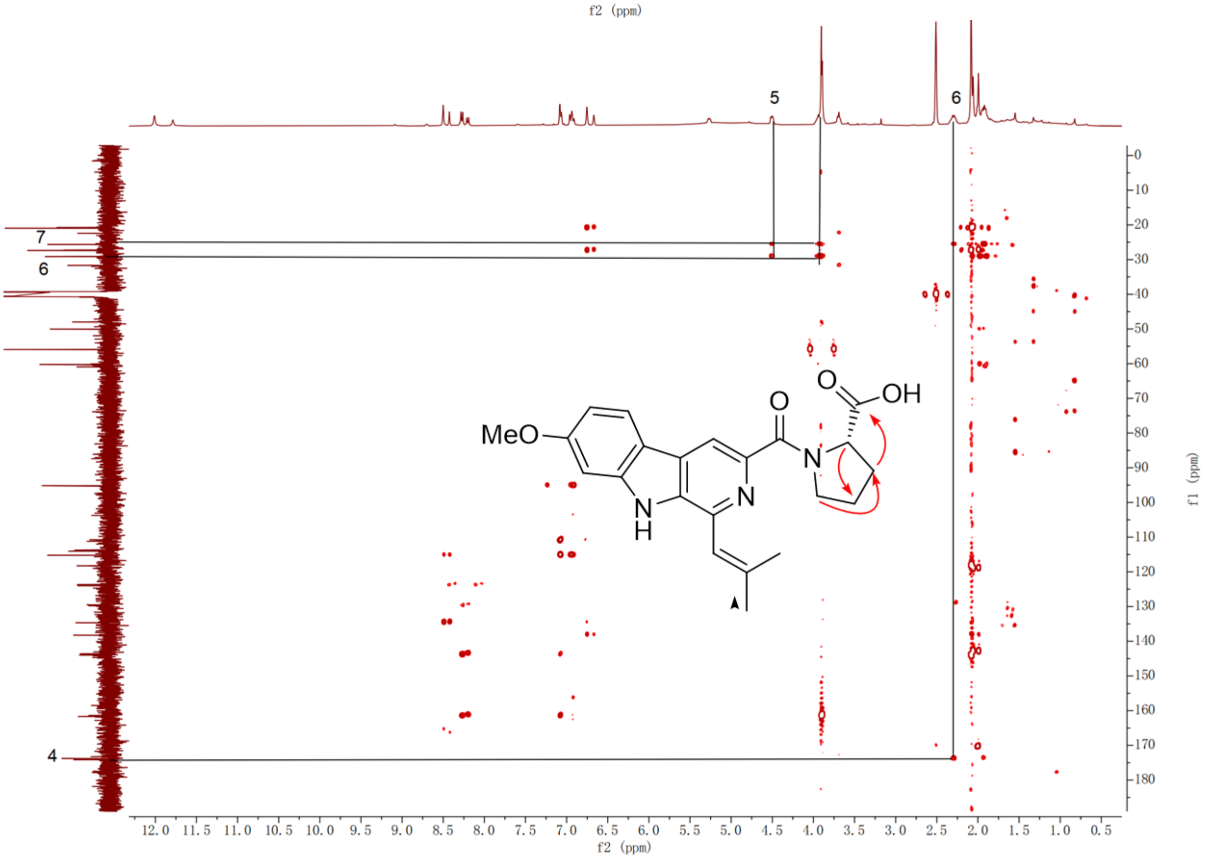
5
6
7
6
4
MeO
O
O
OH
N
N
H
N
f2 (ppm)
f1 (ppm)

Fig. S19. NOESY spectrum of 4 in CD_3OD-d_4 (500 MHz, 500 MHz).

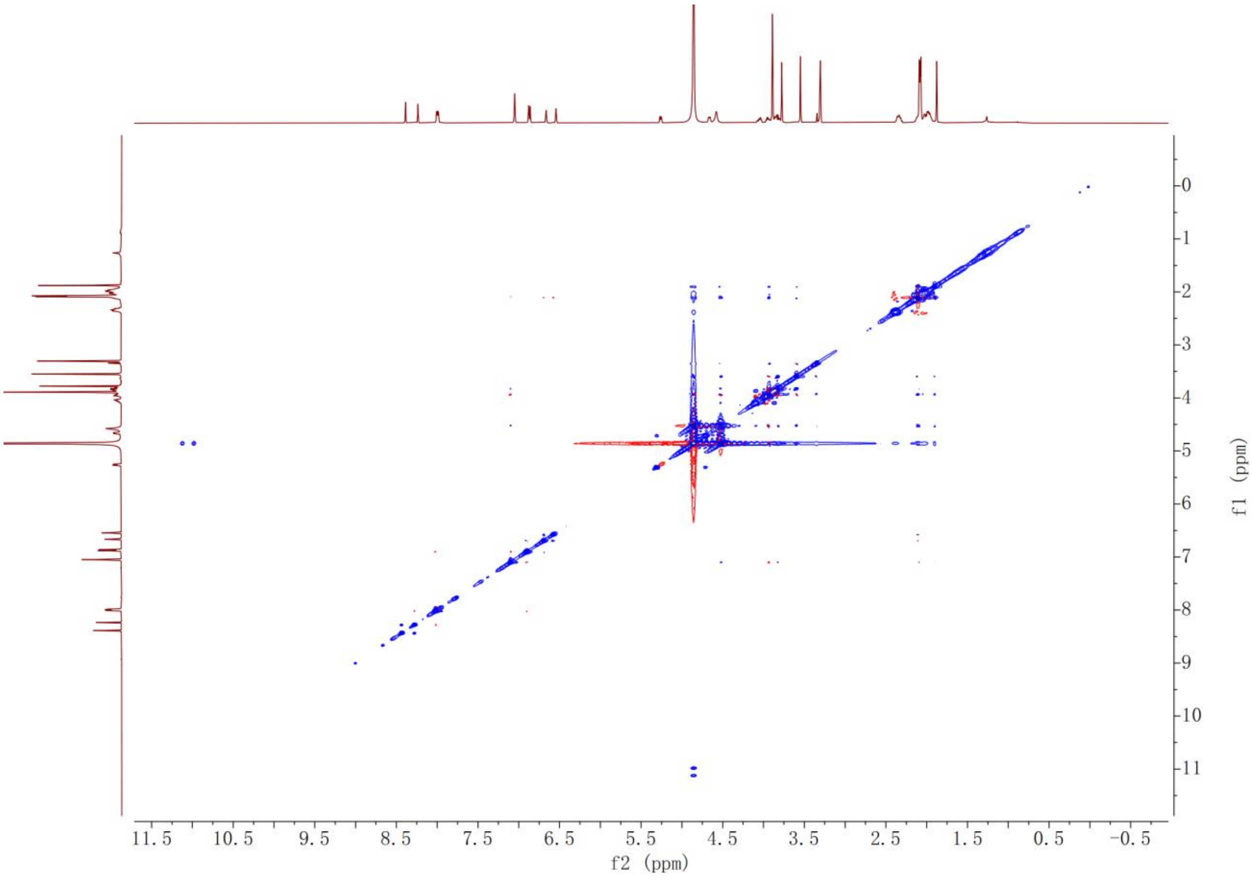

Fig. S20. HRESIMS spectrum of 4.

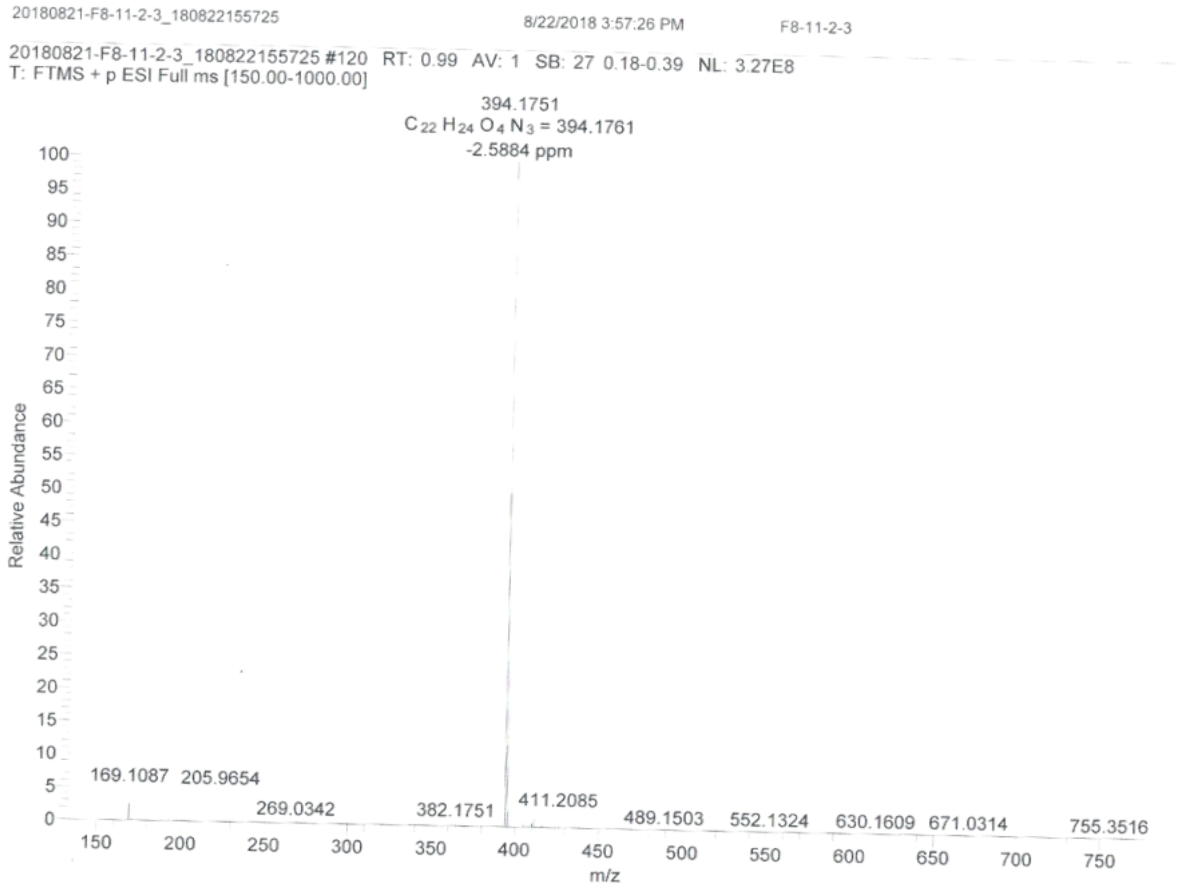

Fig. S21. IR spectrum of 4.

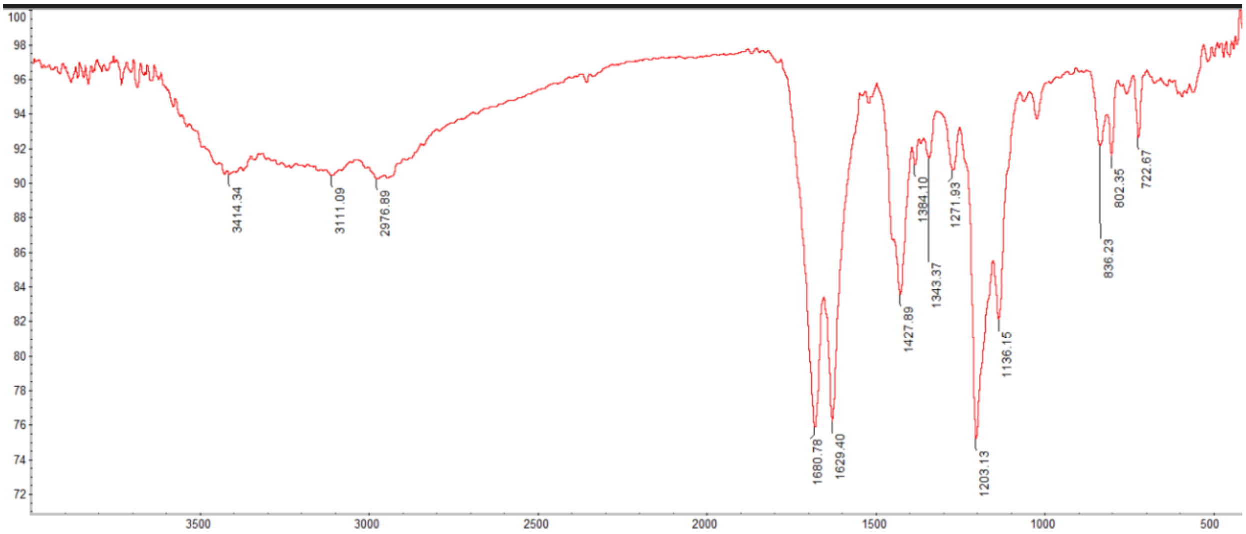

Fig. S22. ^{1}H NMR spectrum of **5** in DMSO-d_6 (400 MHz).

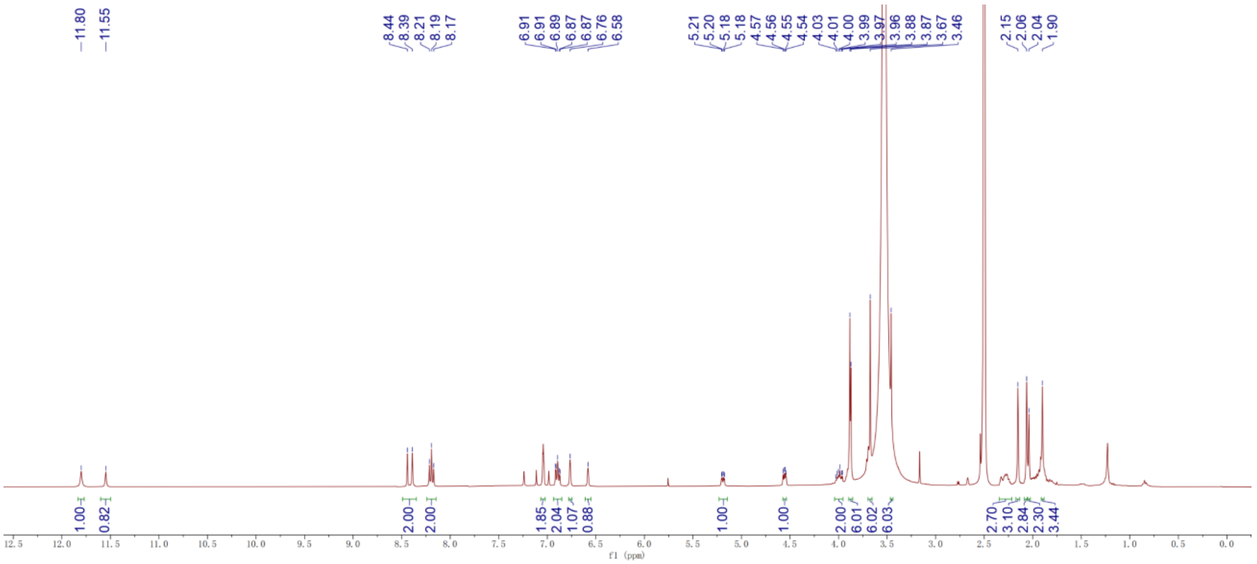

Fig. S23. ^{13}C NMR spectrum of **5** in DMSO-d_6 (100 MHz).

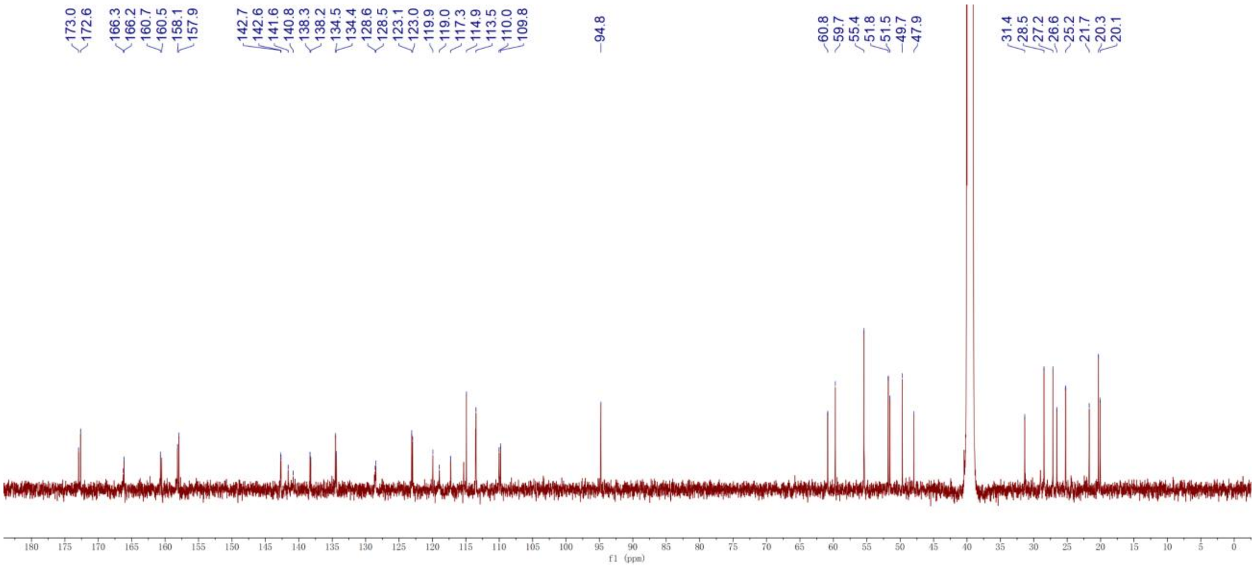

Fig. S24. COSY spectrum of **5** in DMSO-d_6 (500 MHz, 500 MHz).

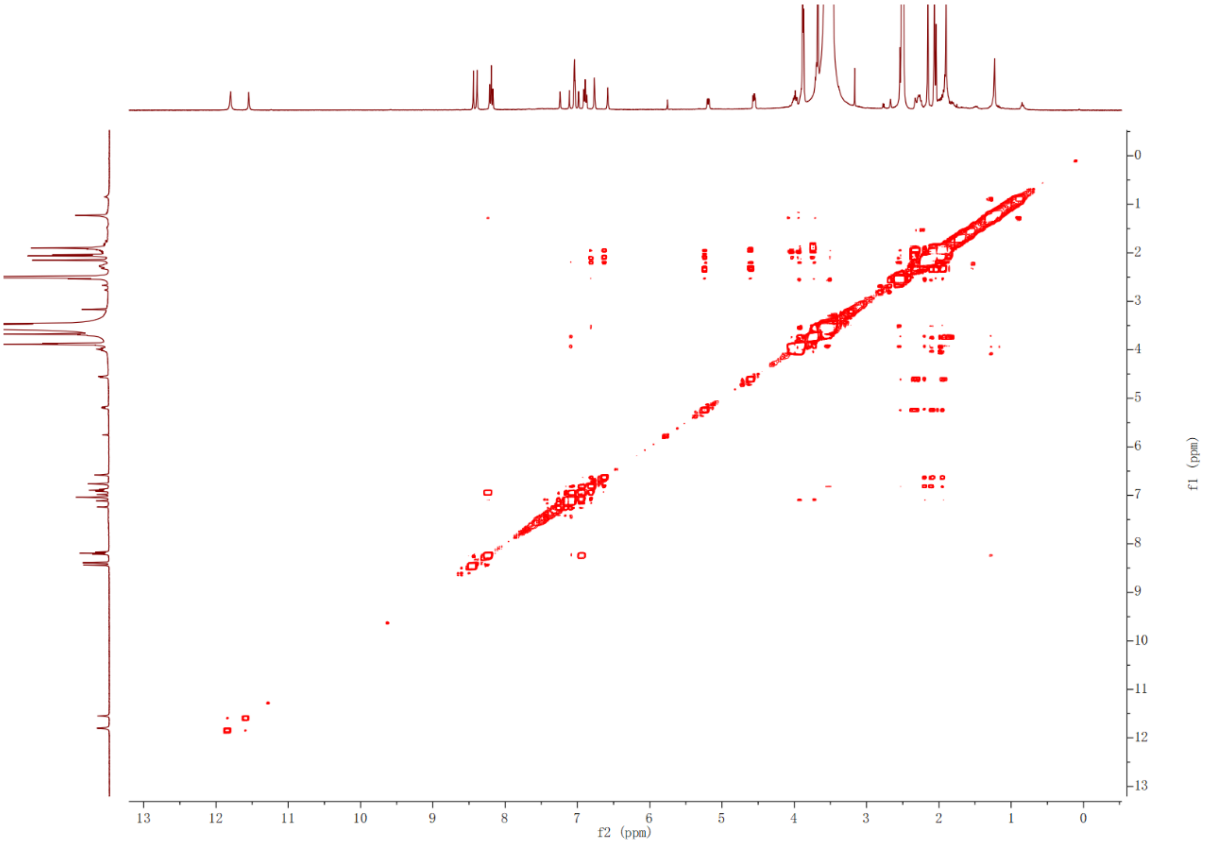

Fig. S25. HSQC spectrum of **5** in DMSO-d_6 (500 MHz, 125 MHz).

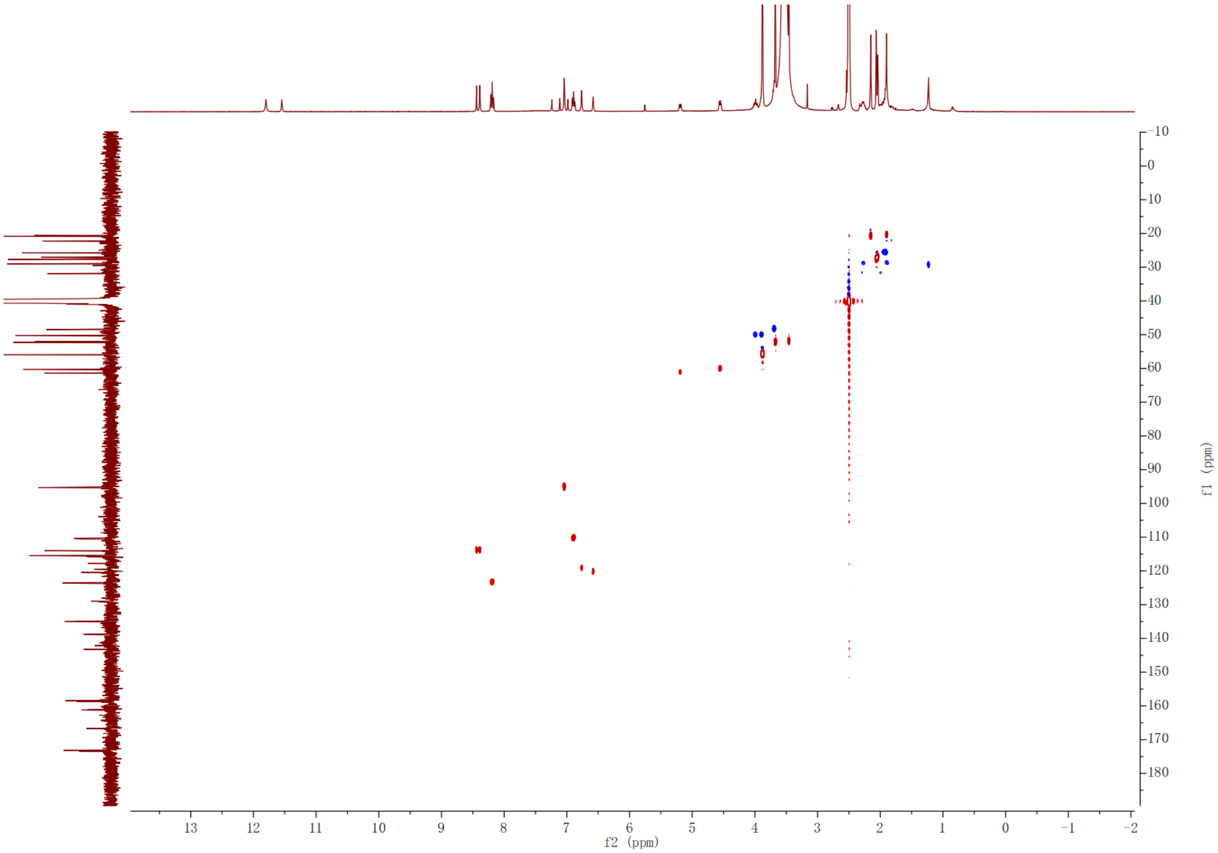

Fig. S26. HMBC spectrum of **5** in DMSO-d_6 (500 MHz, 125 MHz).

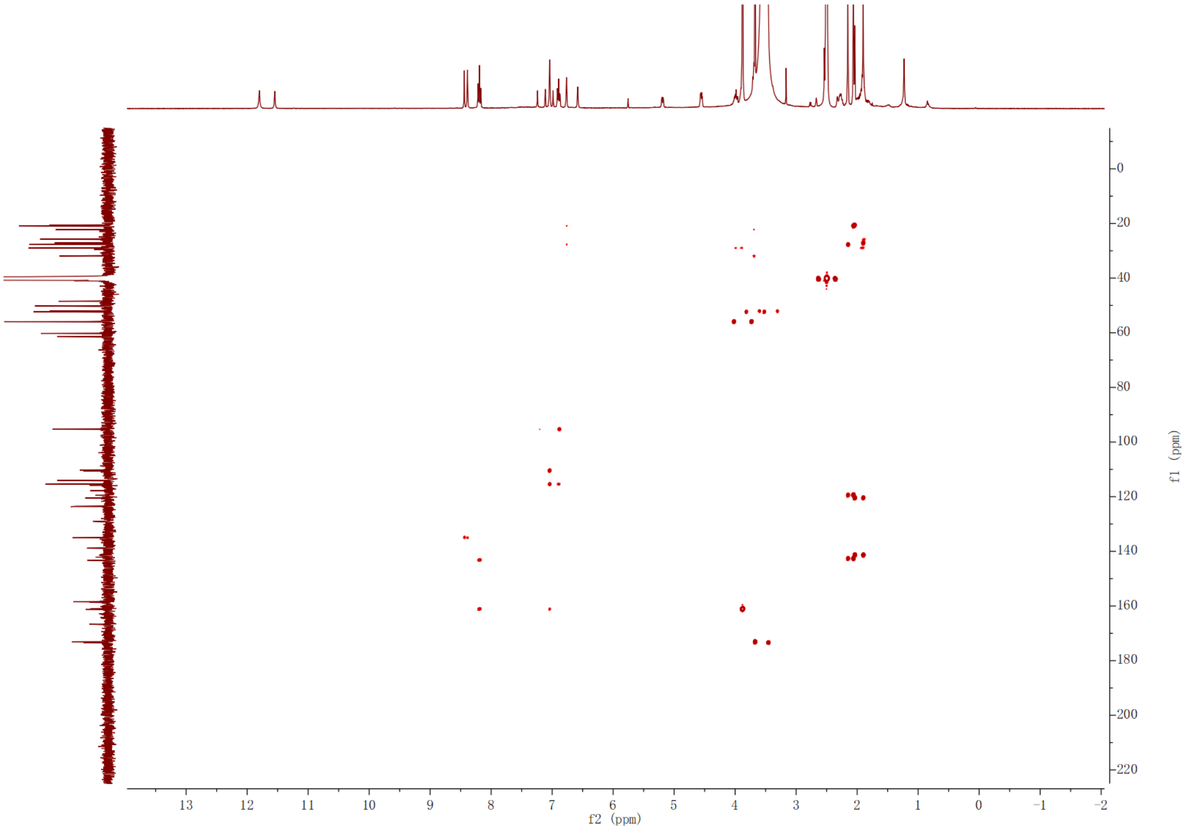

Fig. S27. HRESIMS spectrum of **5**.

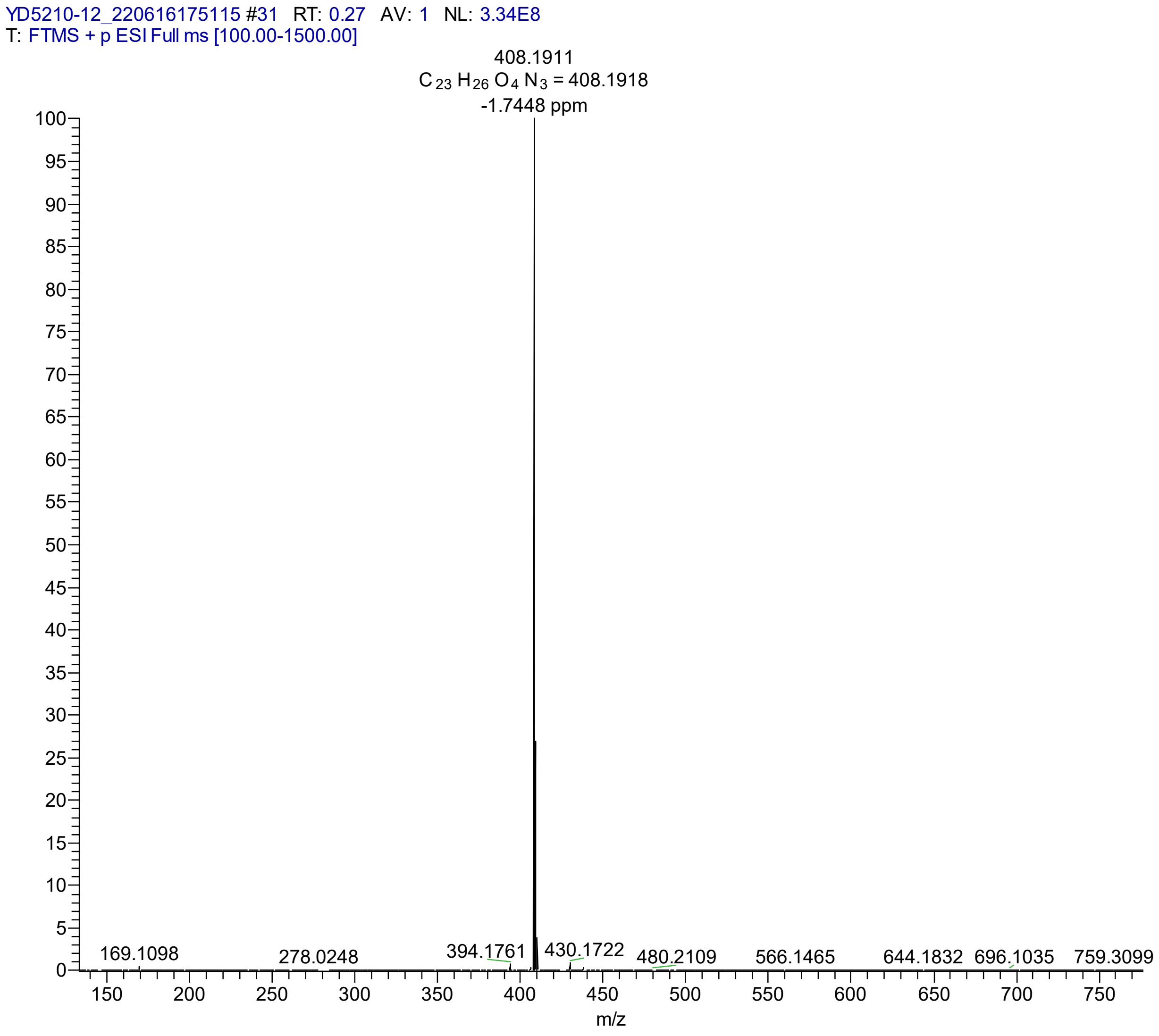

Fig. S28. IR spectrum of **5**.

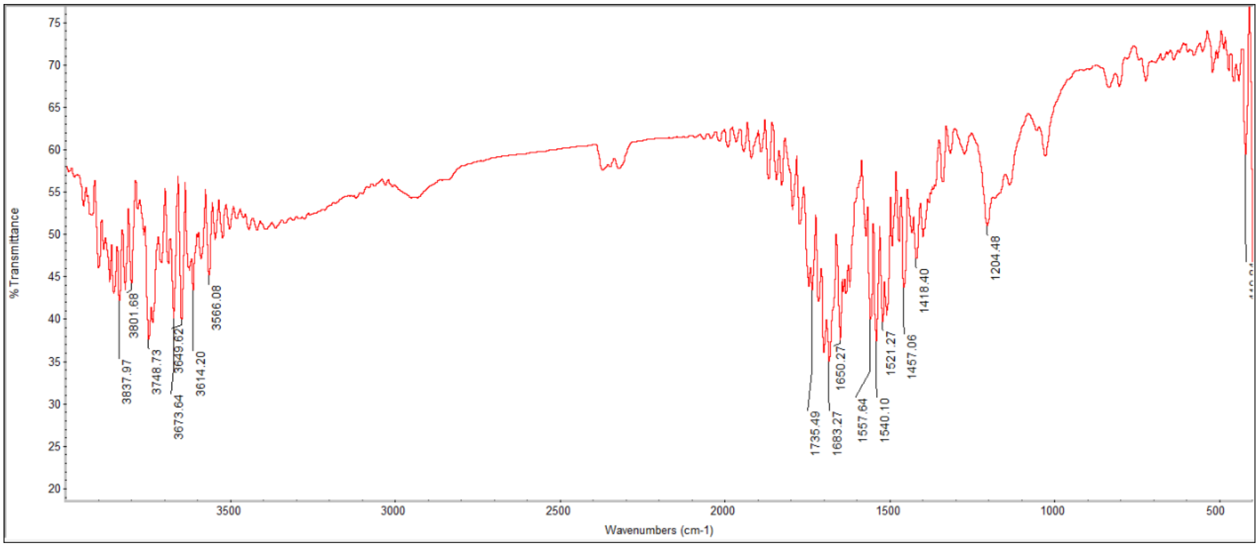

Fig. S29. ^{1}H NMR spectrum of **6** in $CDCl_3$ (400 MHz).

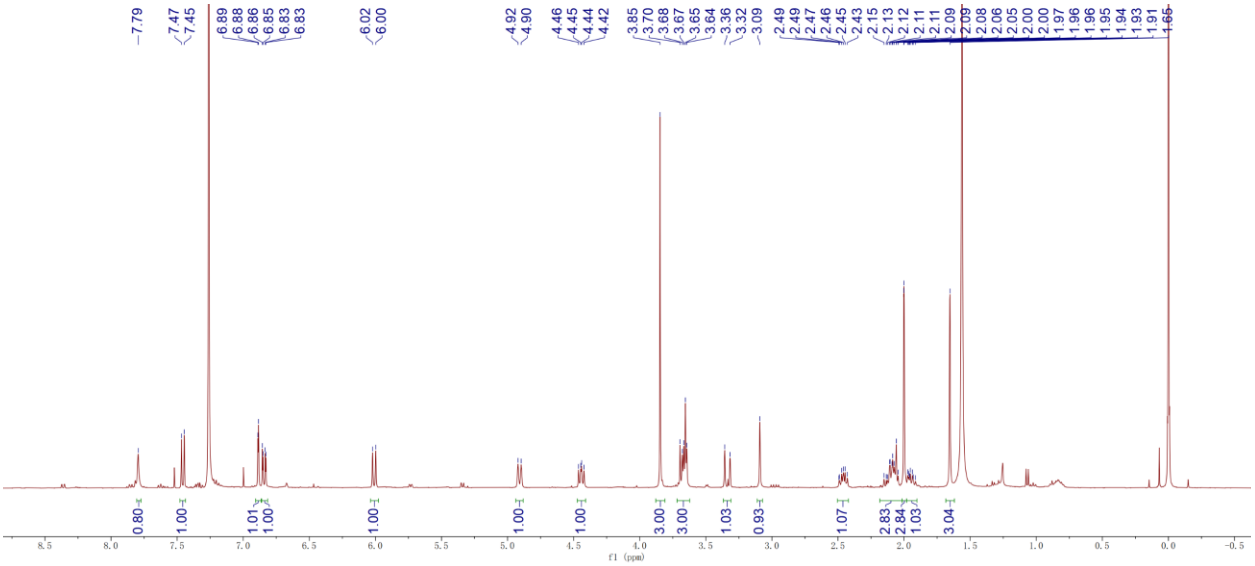

Fig. S30. ^{13}C NMR spectrum of **6** in $CDCl_3$ (100 MHz).

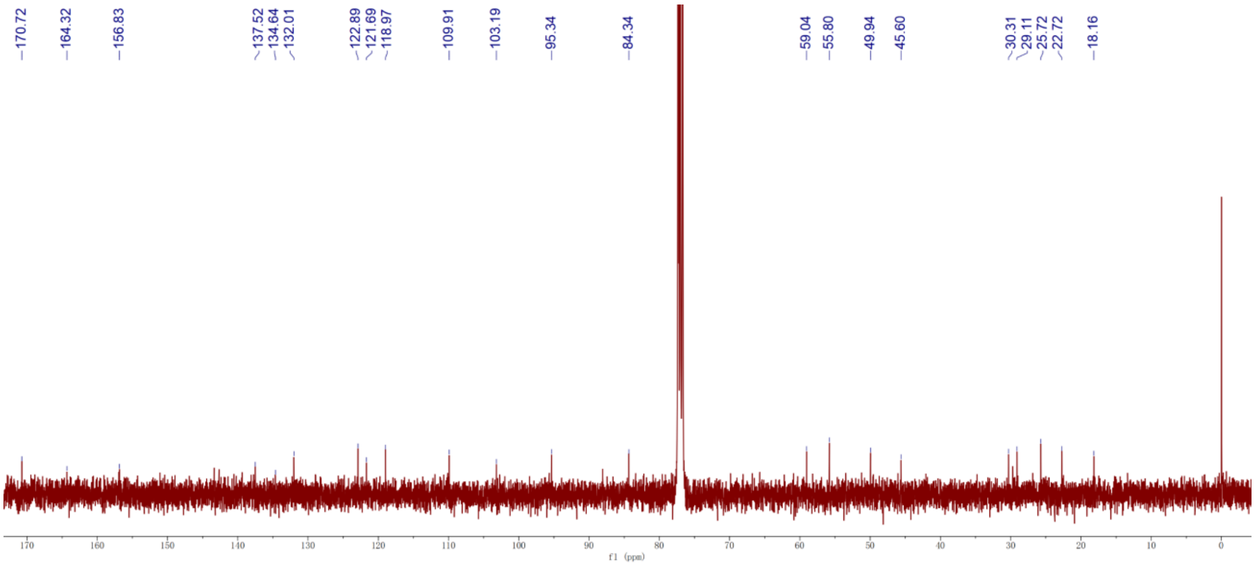

Fig. S31. ^{1}H NMR spectrum of **7** in $CDCl_3$ (400 MHz).

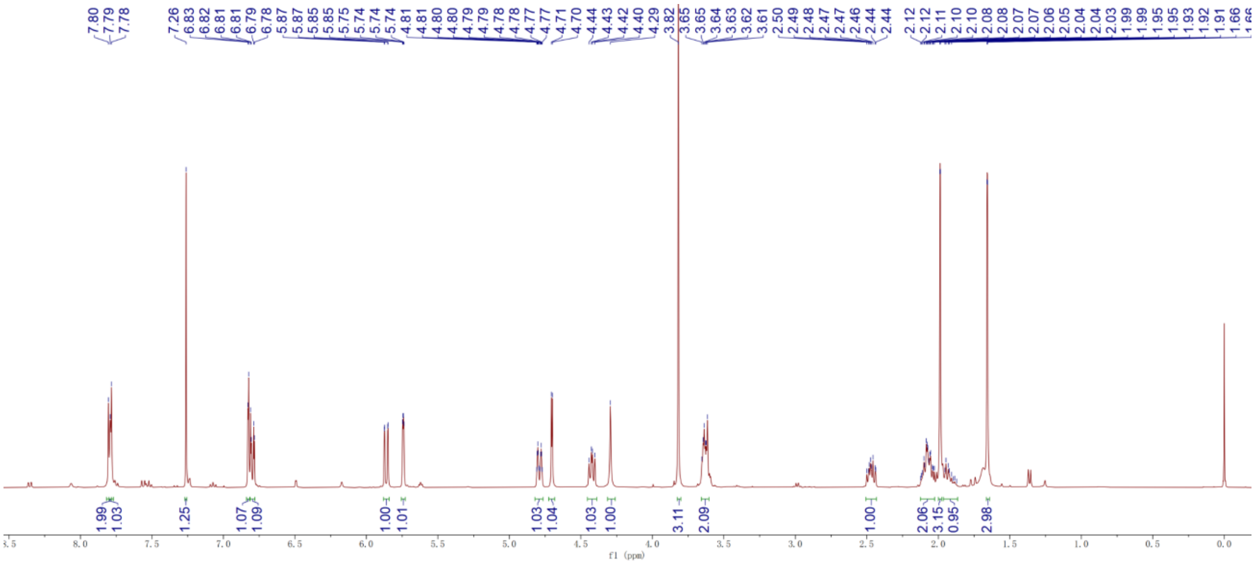

Fig. S32. ^{13}C NMR spectrum of **7** in $CDCl_3$ (100 MHz).

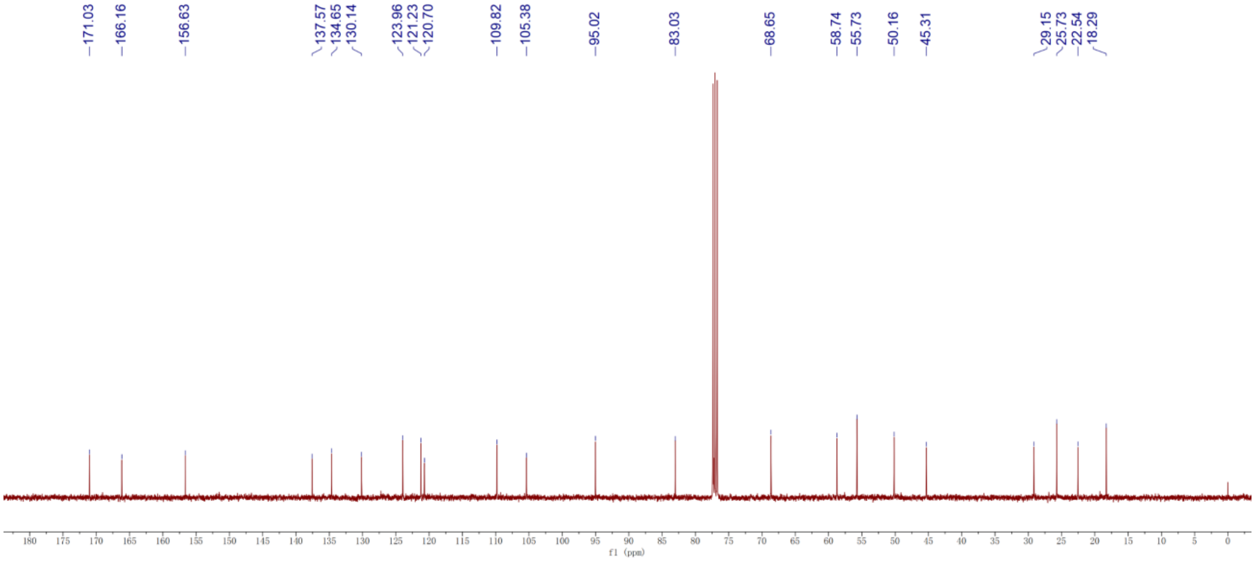

Fig. S33. ^{1}H NMR spectrum of **8** in $CDCl_3$ (400 MHz).

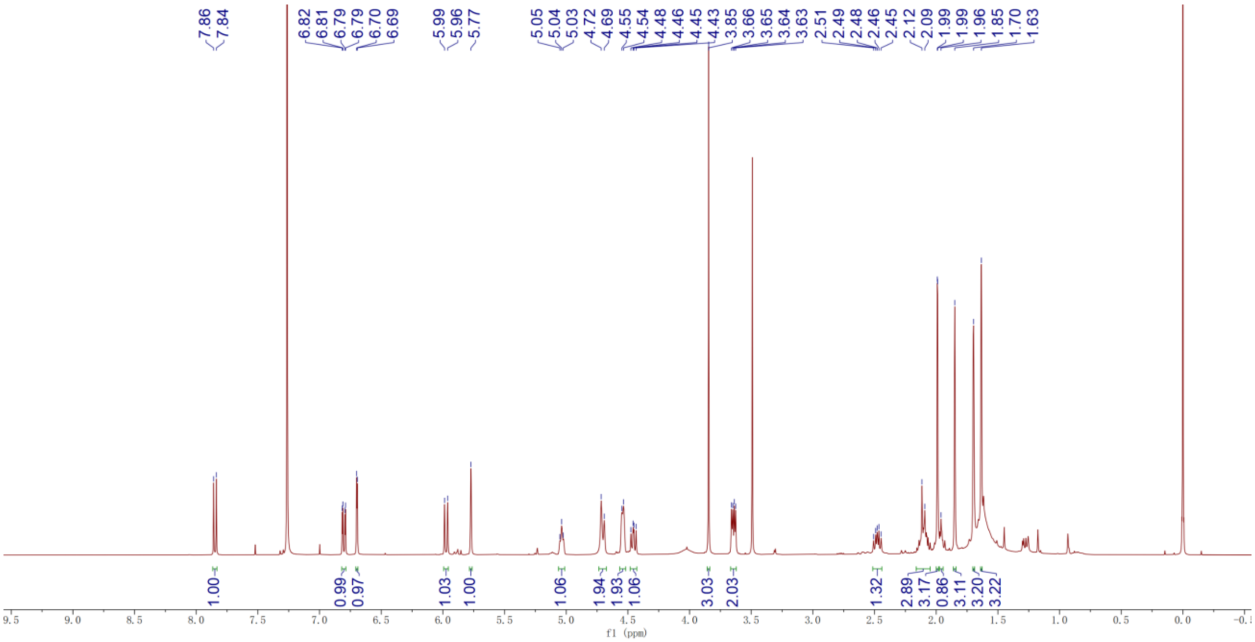

Fig. S34. ^{13}C NMR spectrum of **8** in $CDCl_3$ (150 MHz).

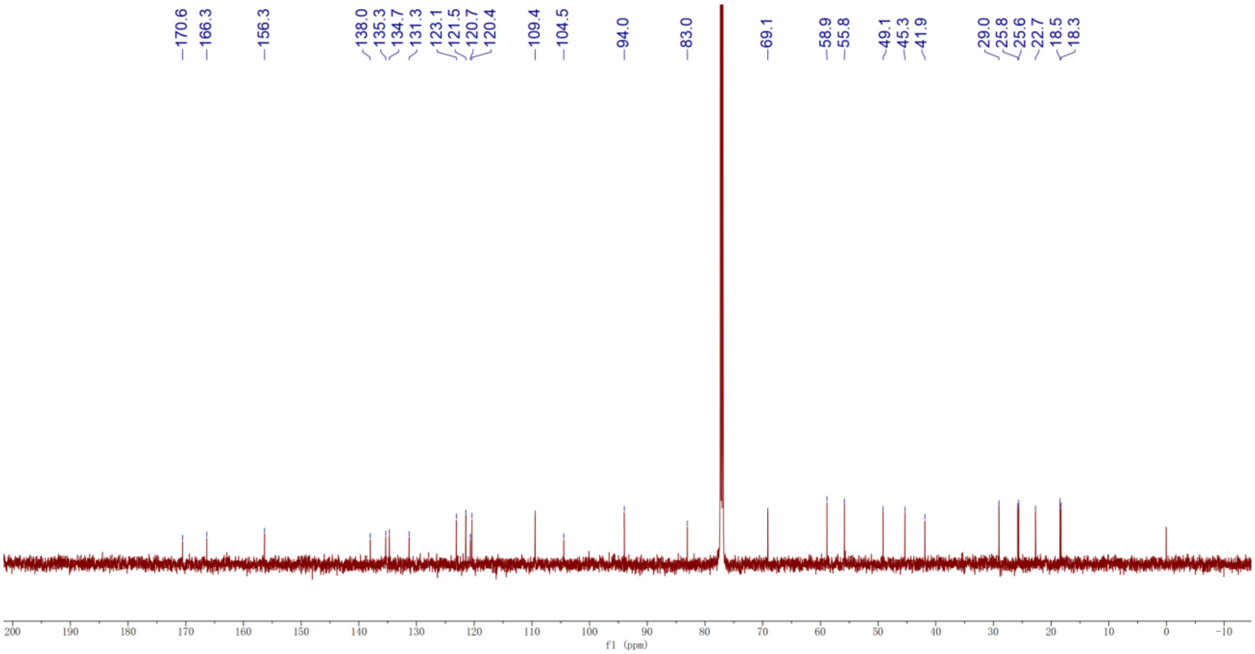

Fig. S35. ^{1}H NMR spectrum of **9** in DMSO-d_6 (400 MHz).

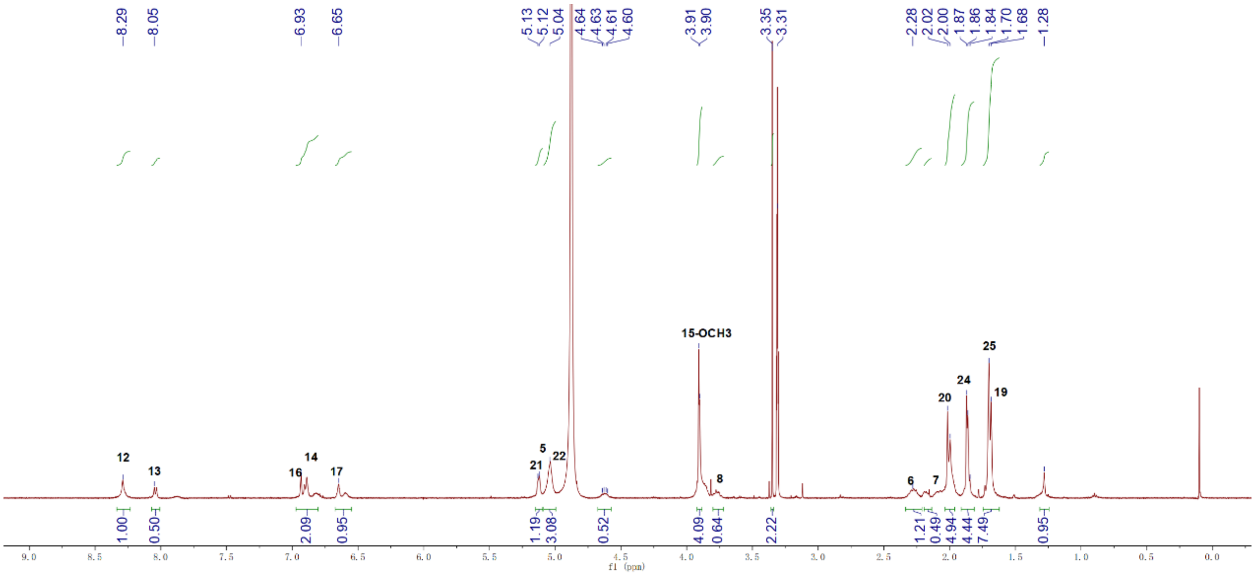

Fig. S36. ^{13}C NMR spectrum of **9** in DMSO-d_6 (100 MHz).

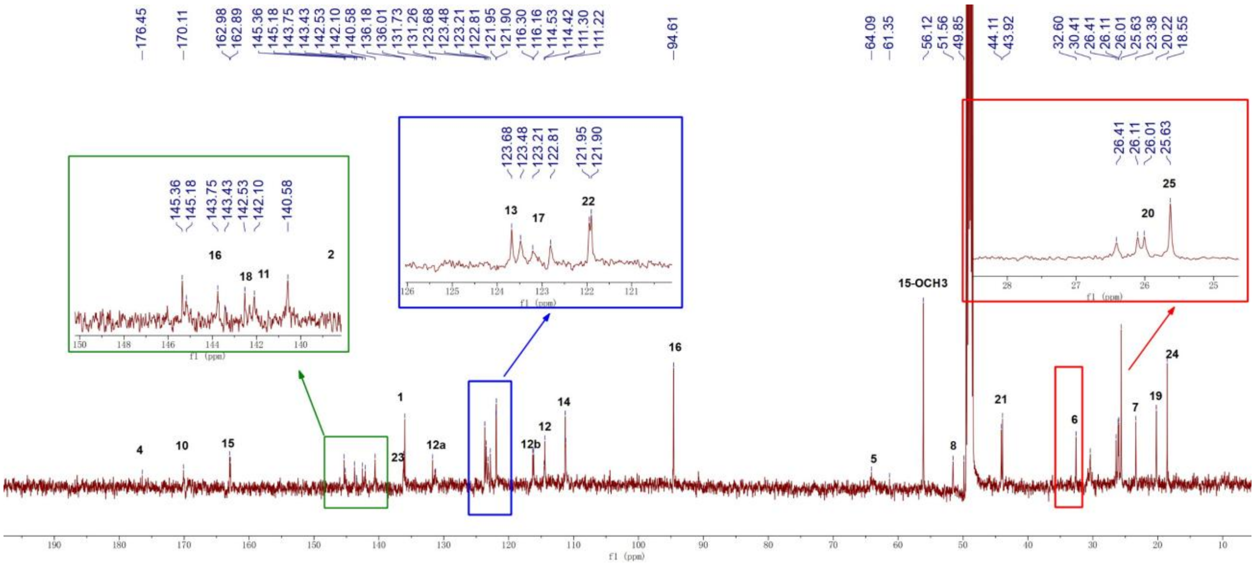

Fig. S37. COSY spectrum of **9** in DMSO-d_6 (500 MHz, 125 HMz).

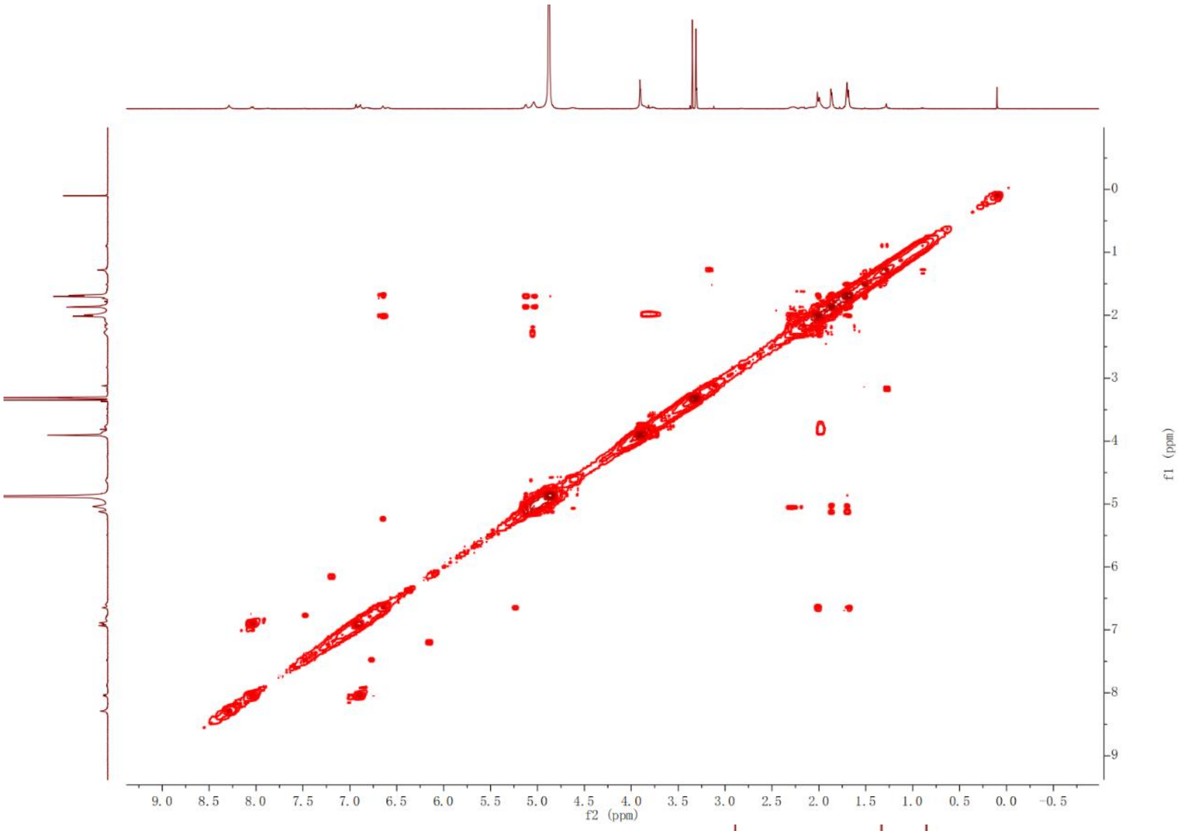

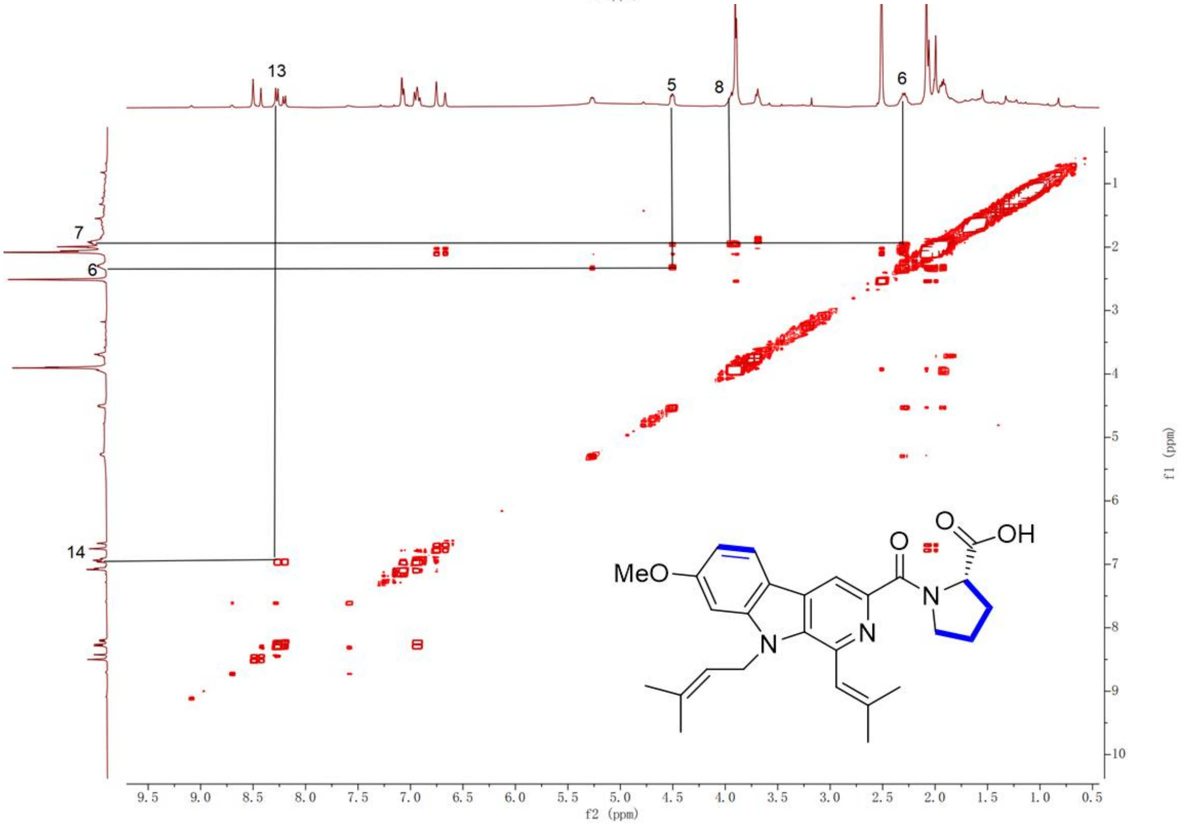

Fig. S38. HSQC spectrum of **9** in DMSO-d_6 (500 MHz, 125 MHz).

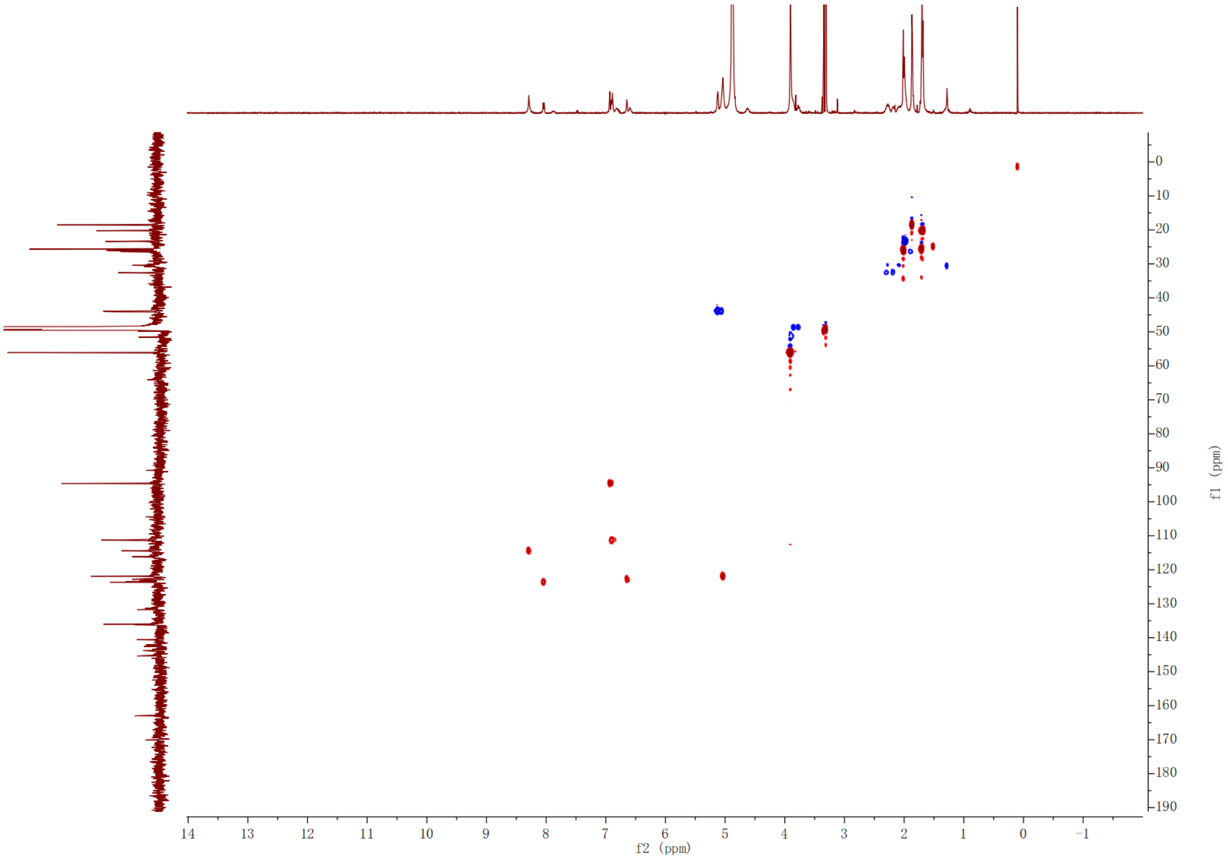

Fig. S39. HMBC spectrum of **9** in DMSO-d_6 (500 MHz, 125 MHz).

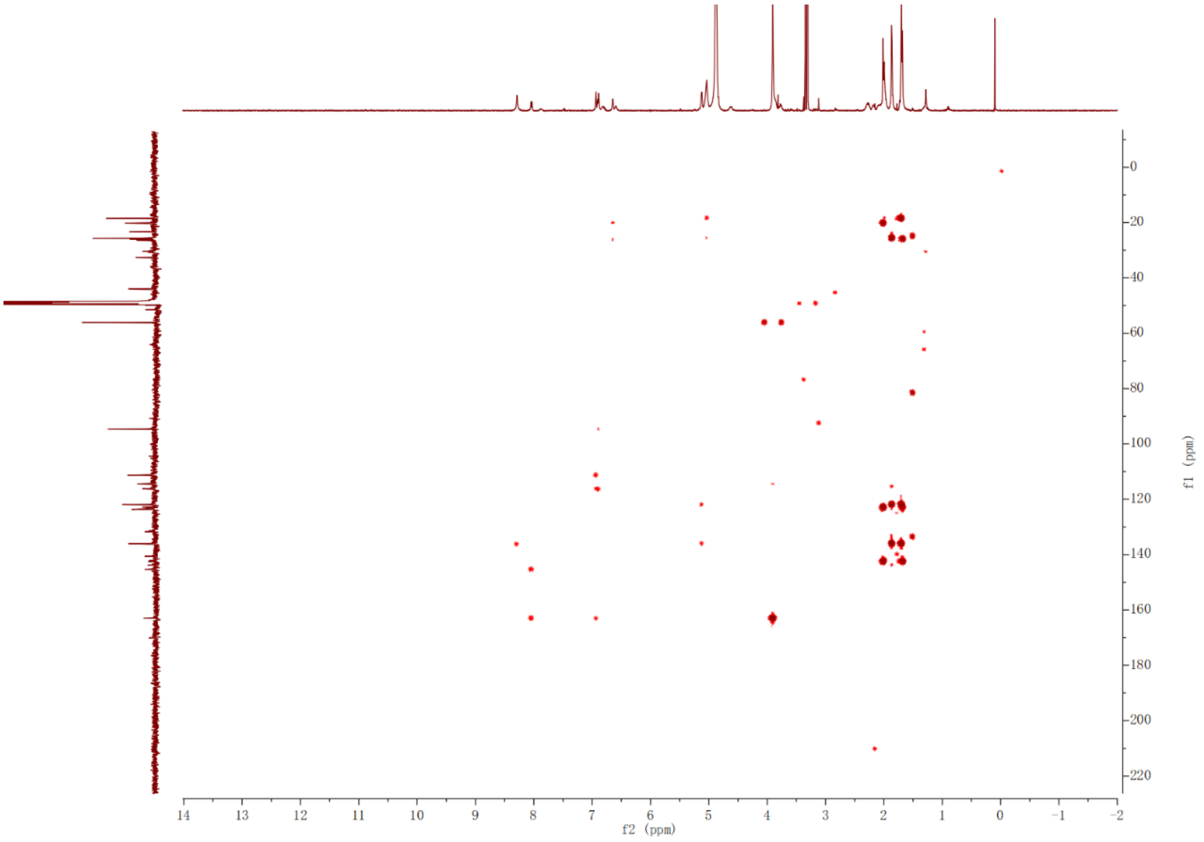

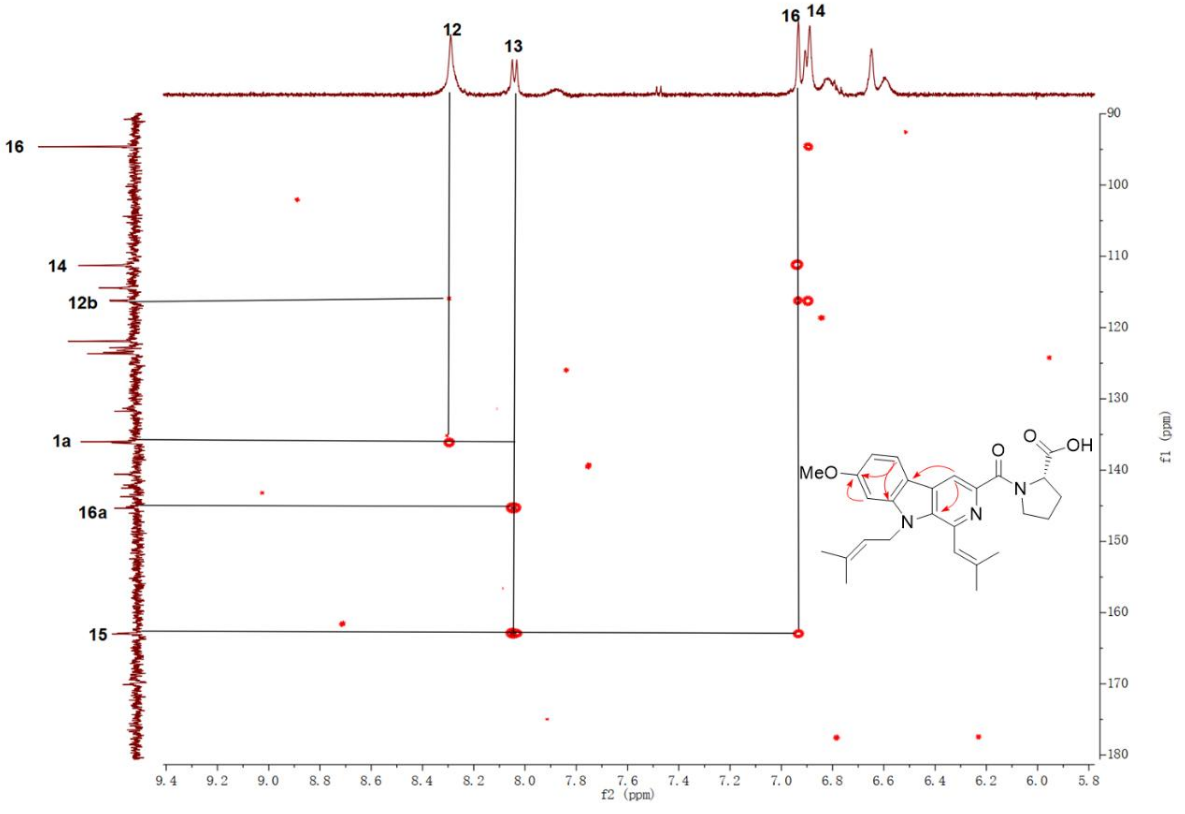

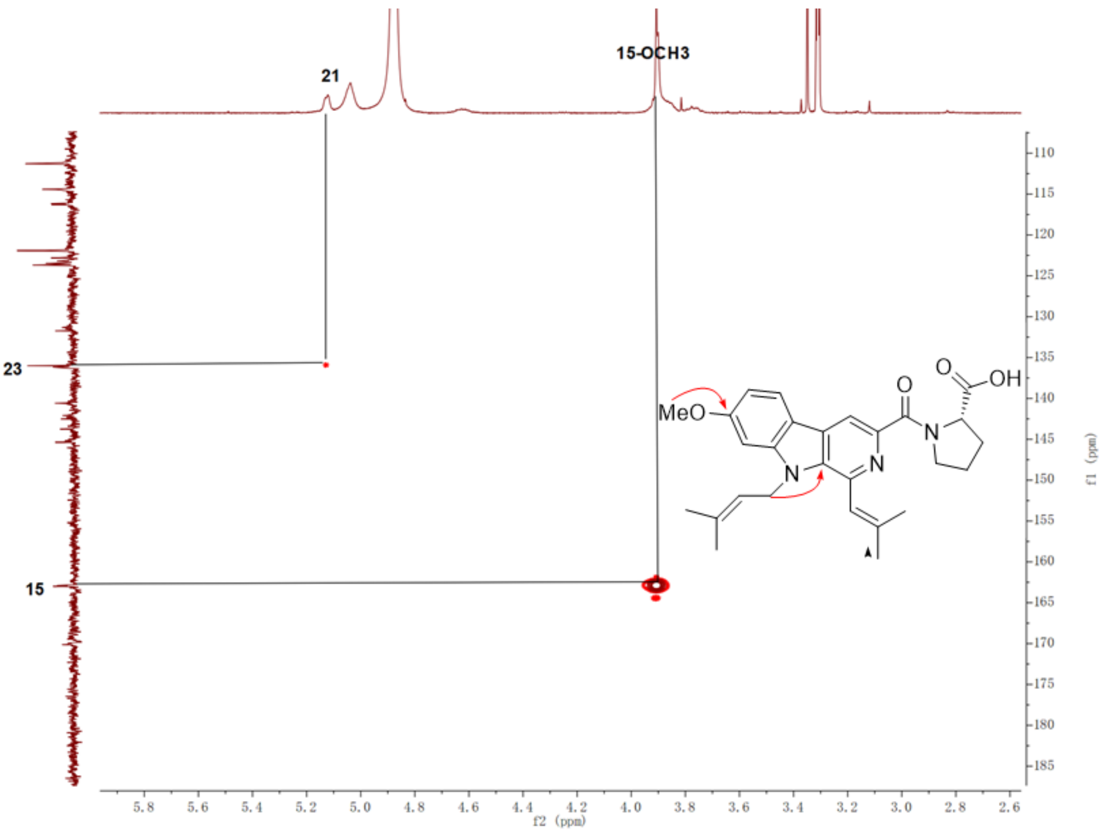
21
15-OCH3
23
15
MeO
N
N
O
N
O
OH
f2 (ppm)
f1 (ppm)

Fig. S40. HRESIMS spectrum of **9**.

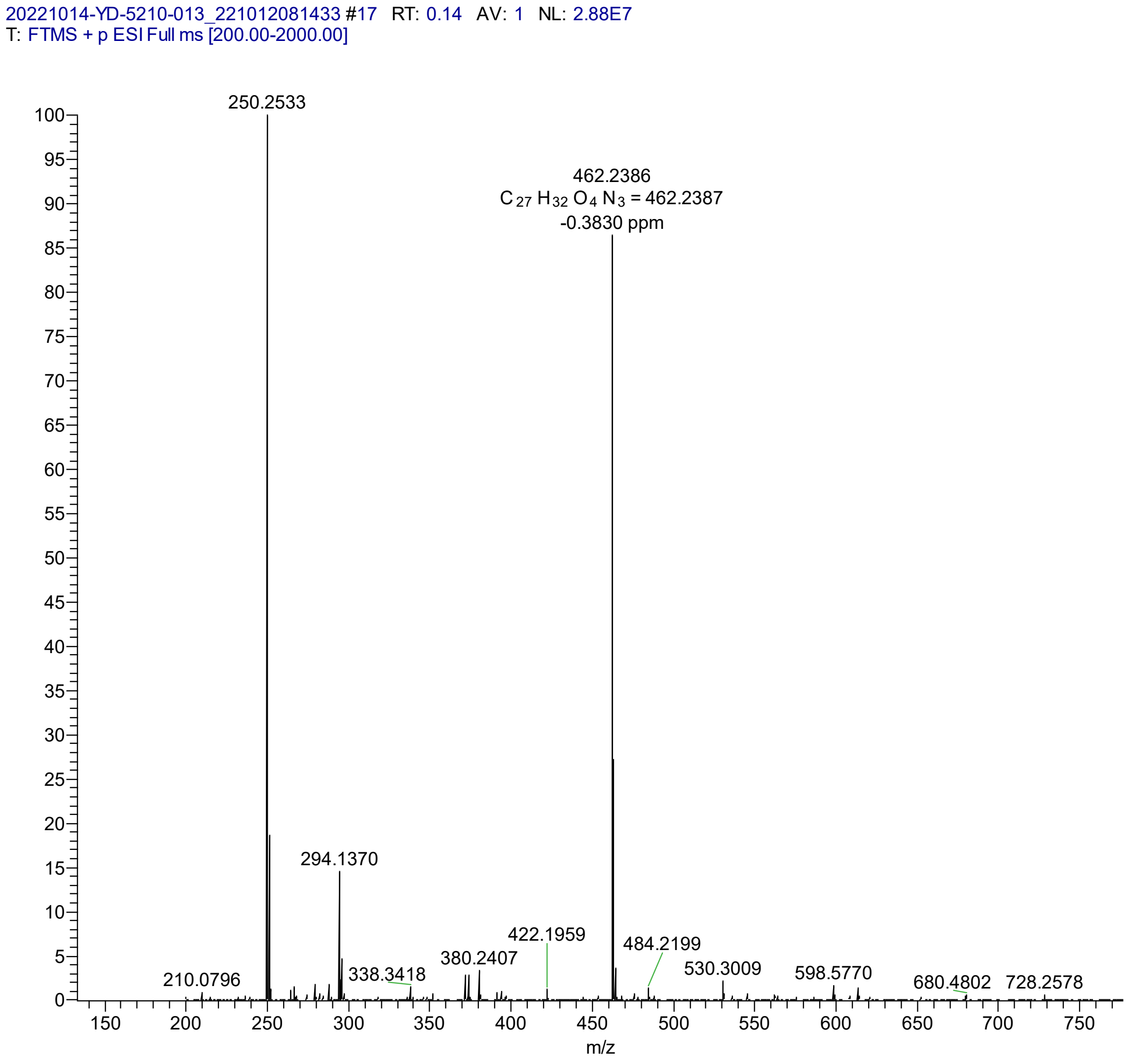

Fig. S41. IR spectrum of **9**.

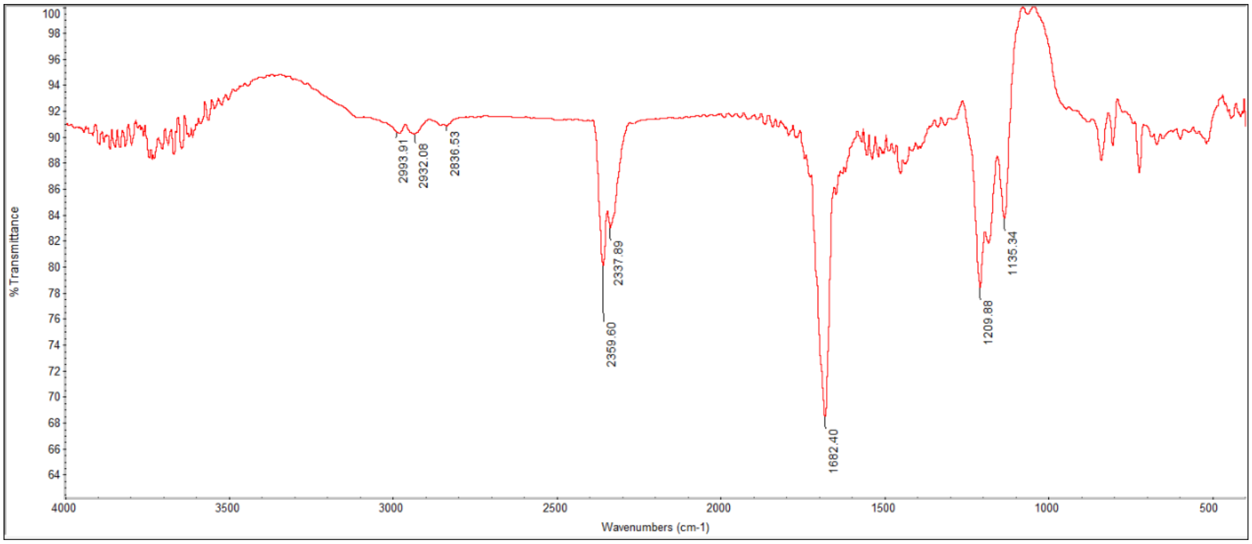

Fig. S42. ^{1}H NMR spectrum of **10** in DMSO-d_6 (400 MHz).

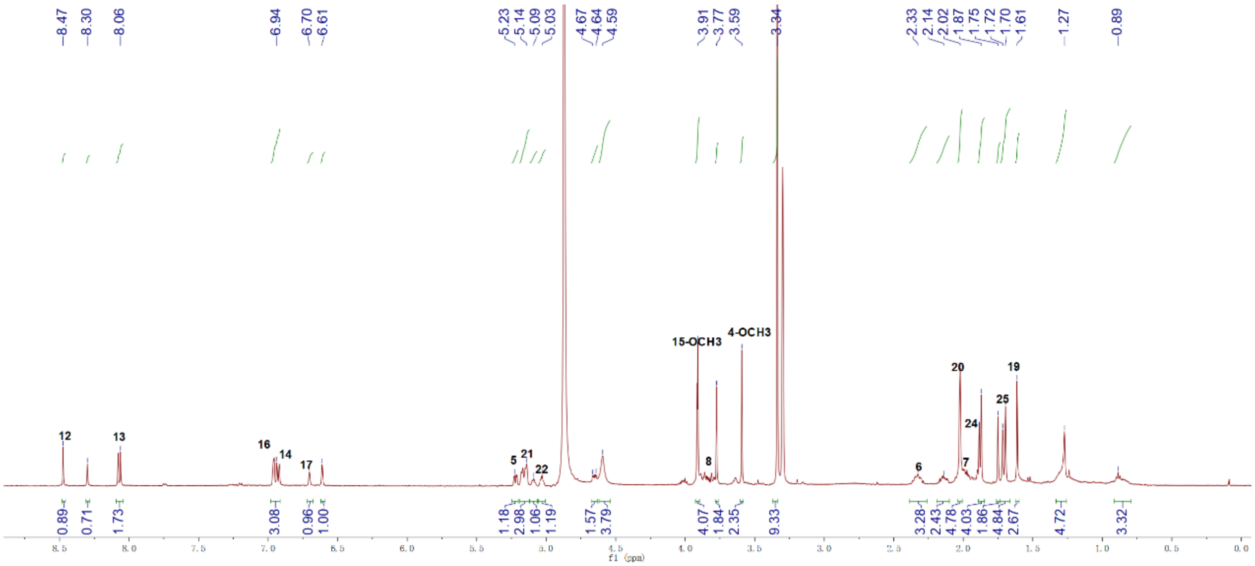

Fig. S43. ^{13}C NMR spectrum of **10** in DMSO-d_6 (100 MHz).

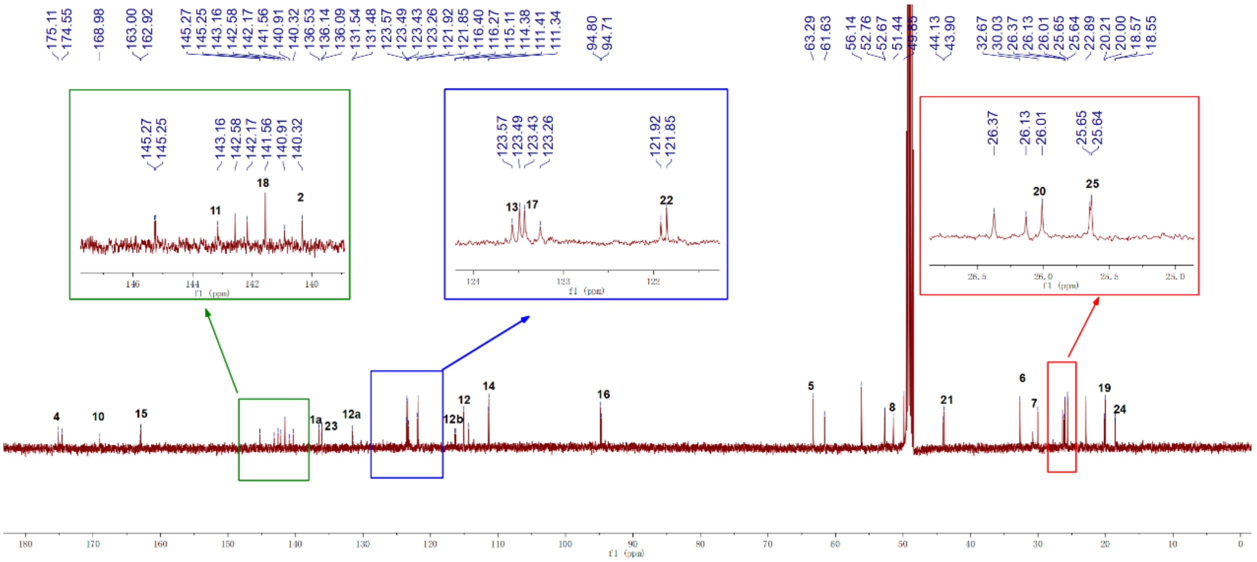

Fig. S44. COSY spectrum of **10** in DMSO-d_6 (500 MHz, 500 MHz).

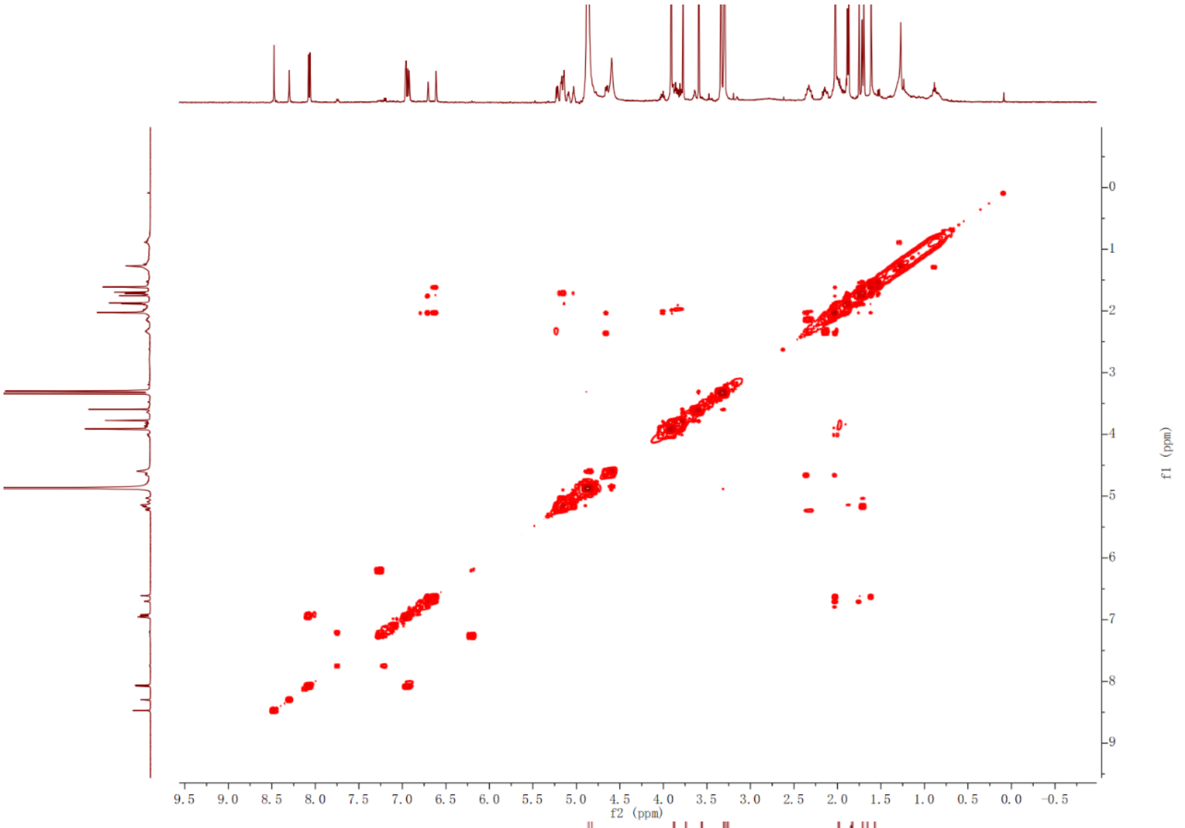

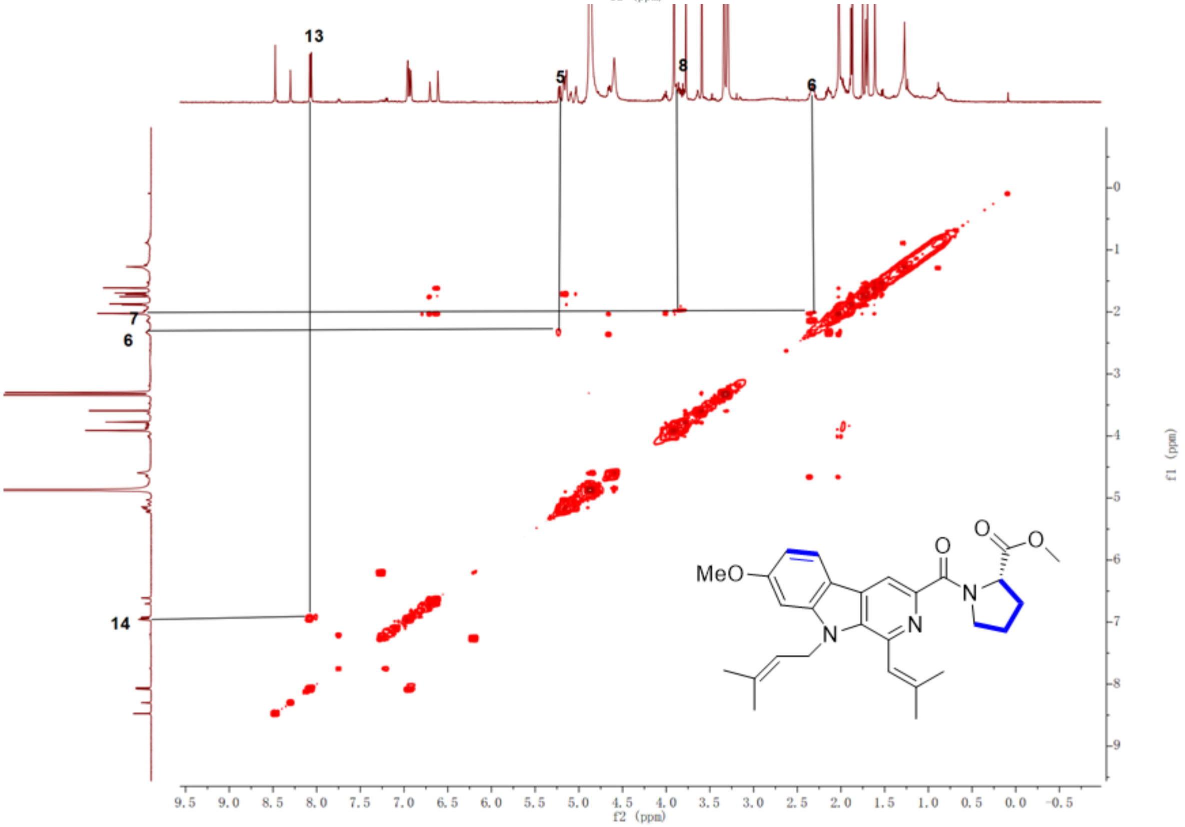

Fig. S45. HSQC spectrum of **10** in DMSO-d_6 (500 MHz, 125 MHz).

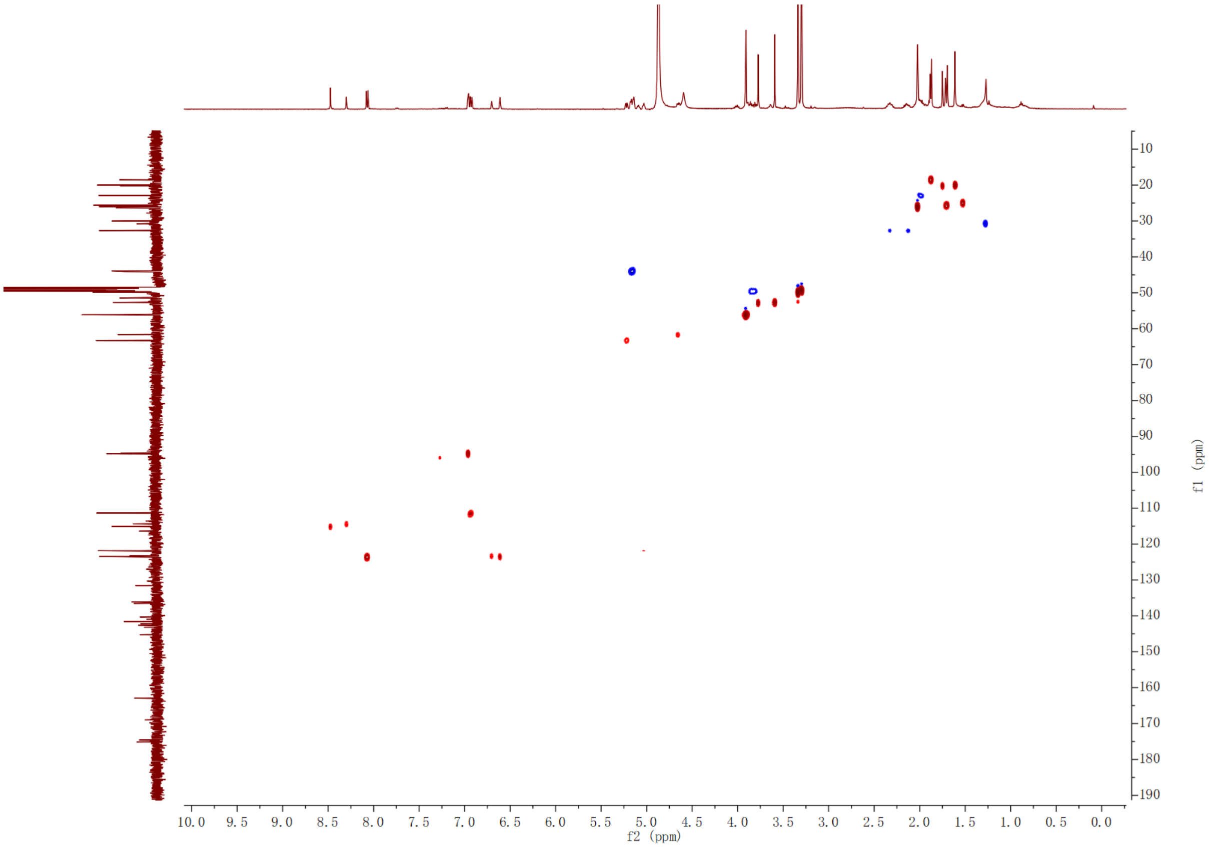

Fig. S46. HMBC spectrum of **10** in DMSO-d_6 (500 MHz, 125 MHz).

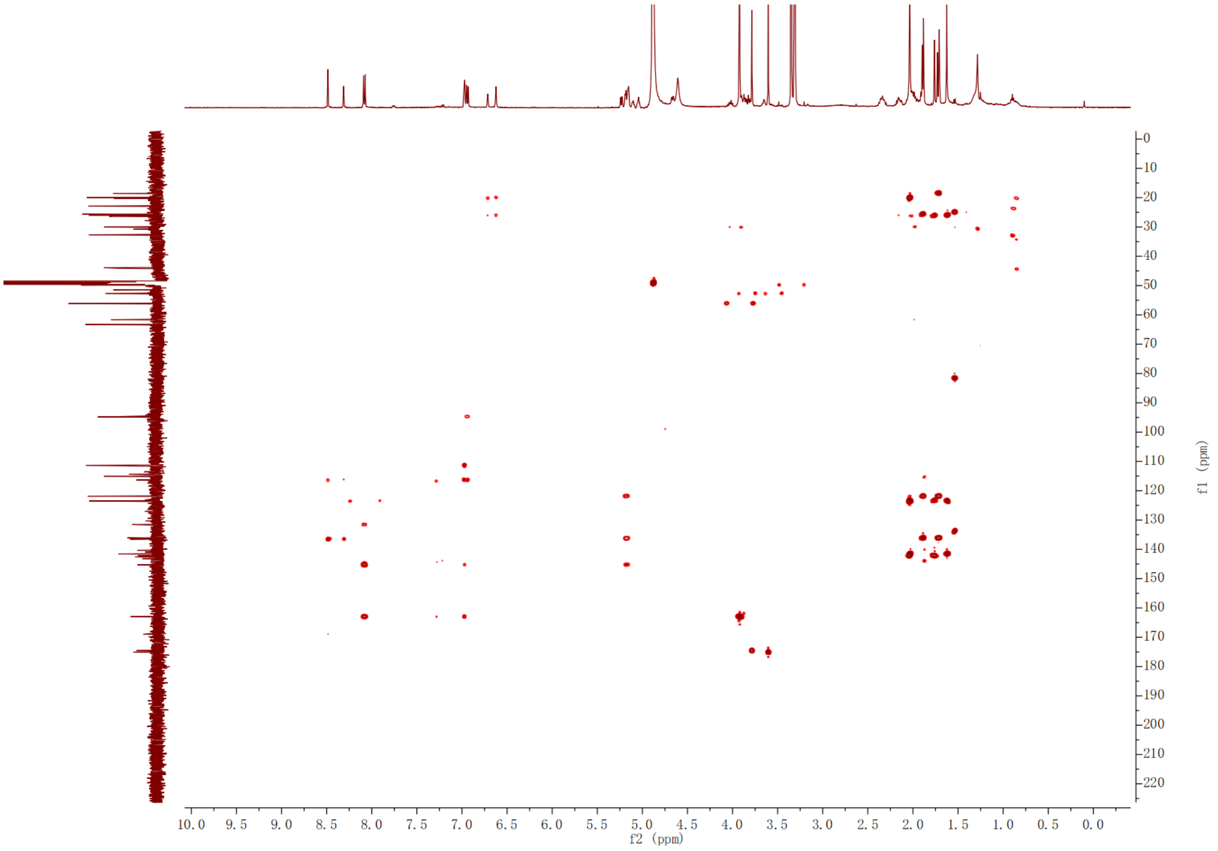

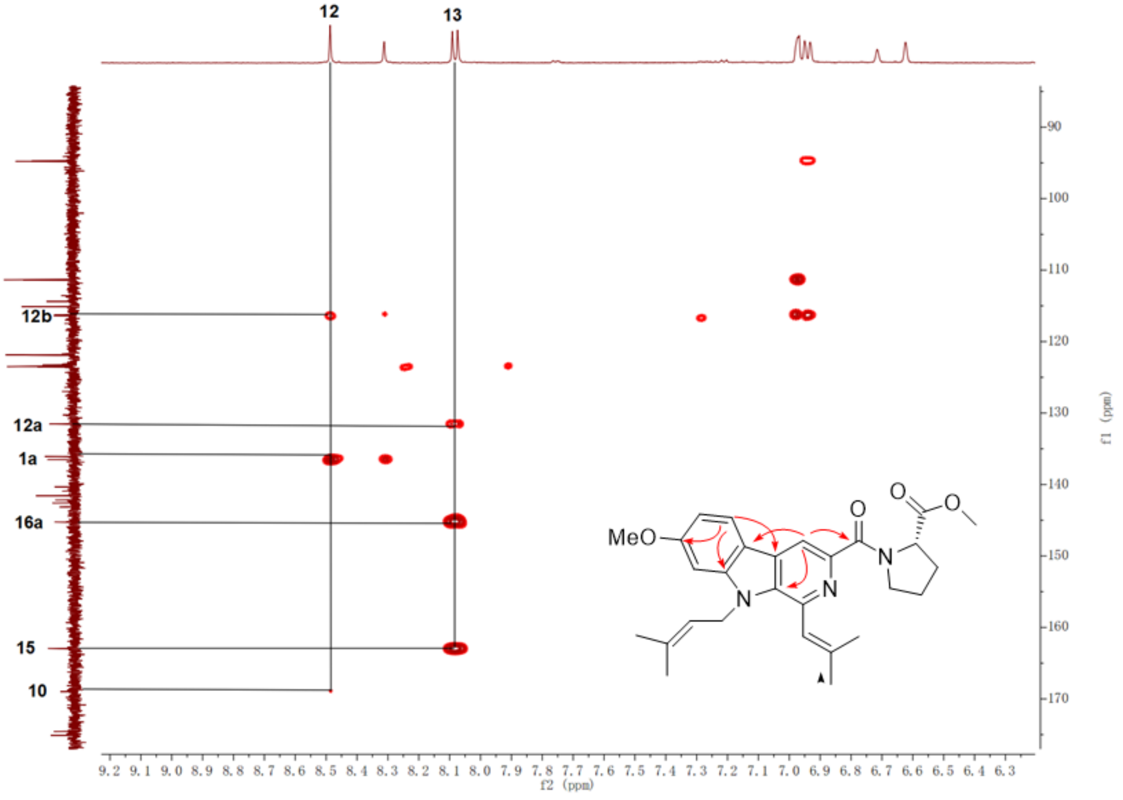

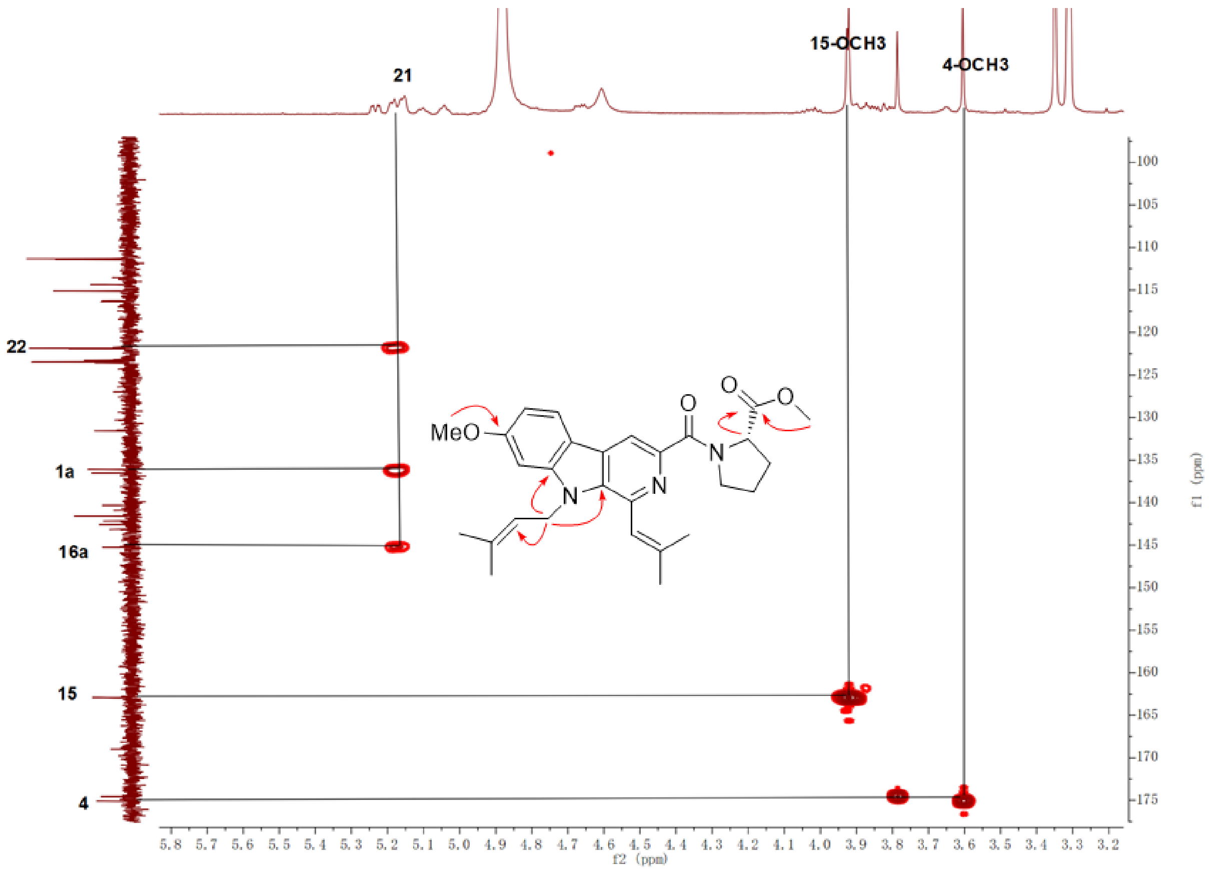

21
15-OCH3
4-OCH3
22
1a
16a
15
4
MeO
N
N
O
O
O
f2 (ppm)
f1 (ppm)

Fig. S47. HRESIMS spectrum of **10**.

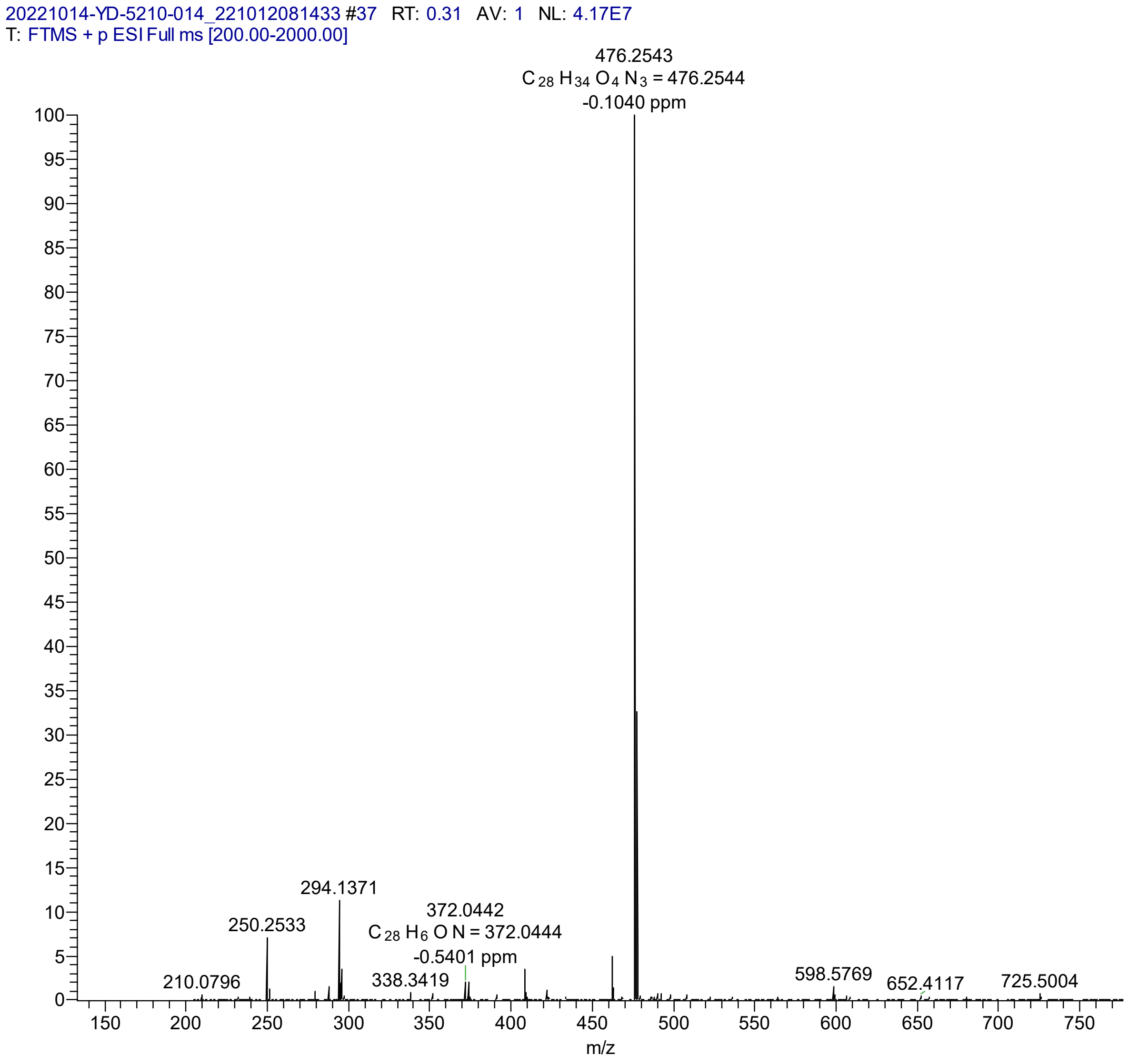

Fig. S48. IR spectrum of **10**.

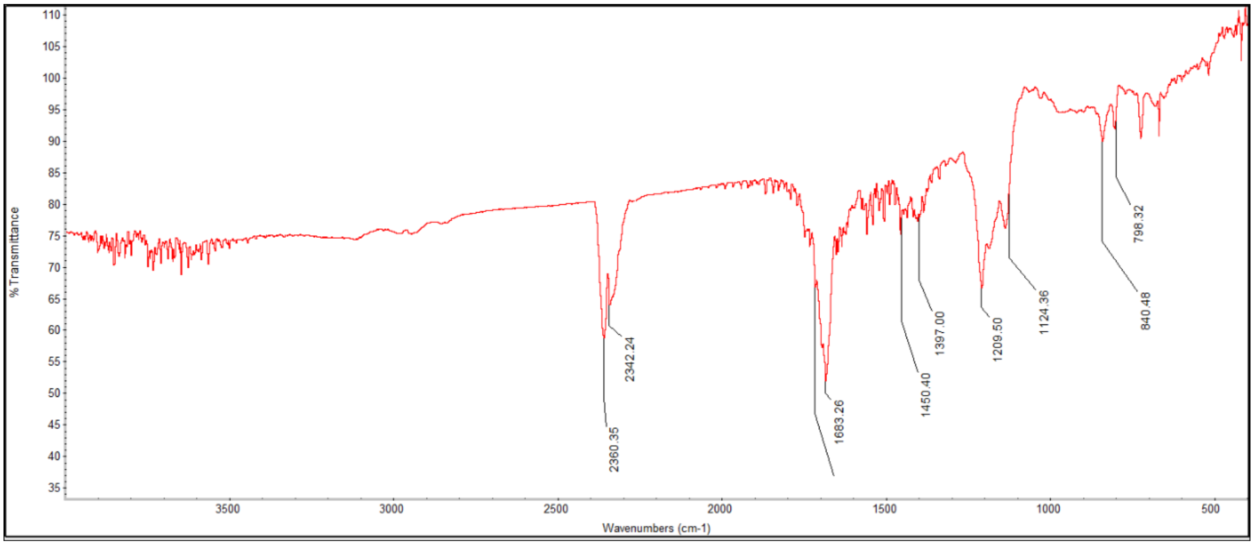

Fig. S49. ^{1}H NMR spectrum of **11** in $CDCl_3$ (400 MHz).

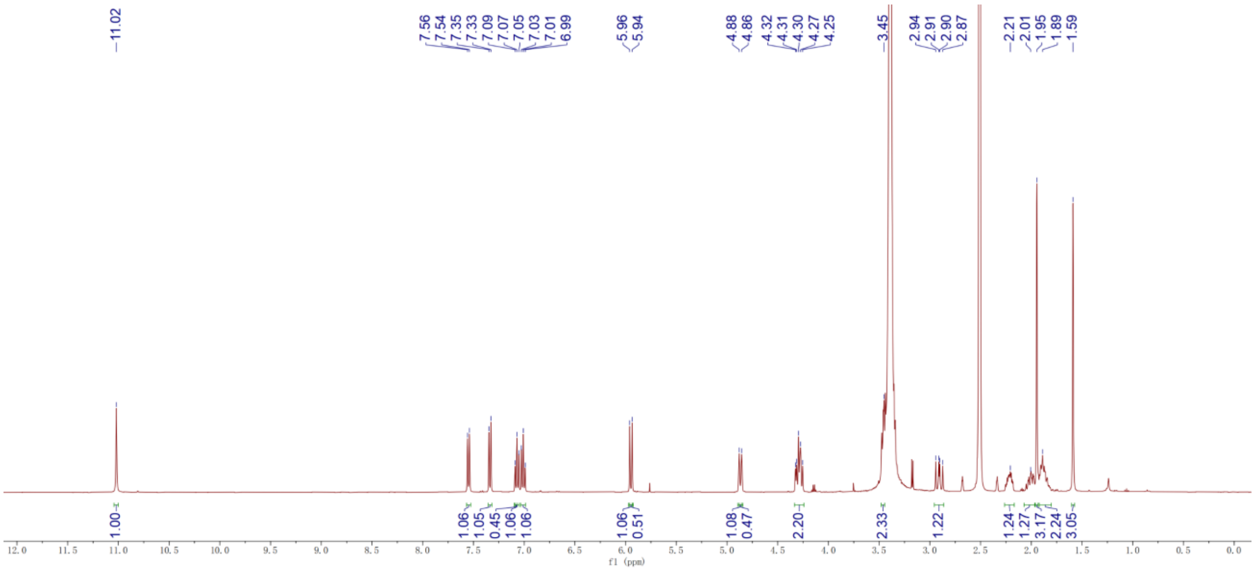

Fig. S50. ^{13}C NMR spectrum of **11** in $CDCl_3$ (100 MHz).

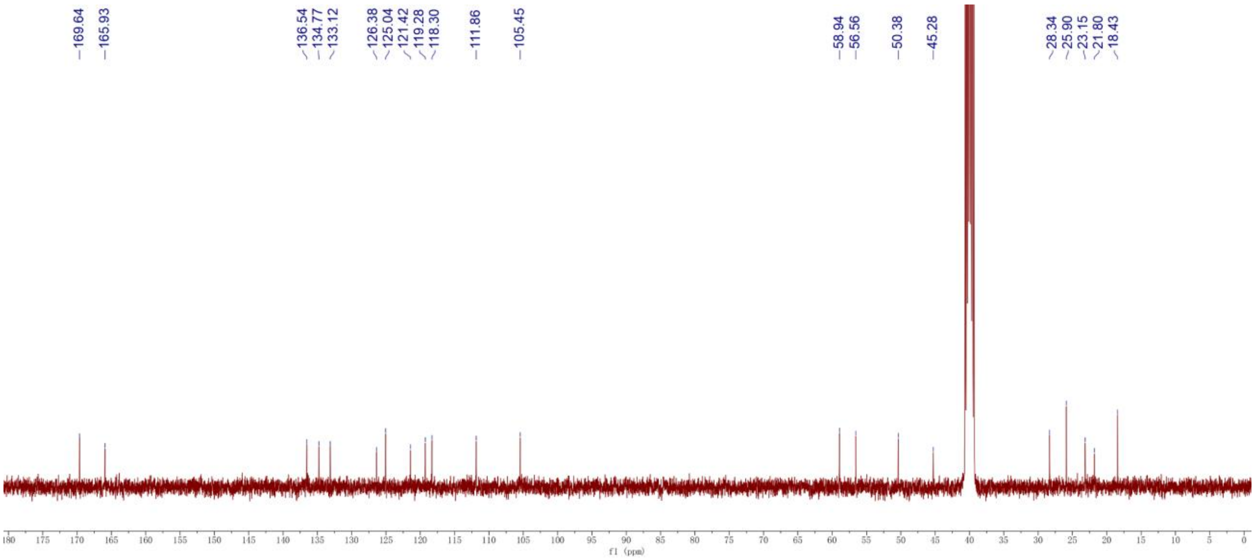

Fig. S51. ^{1}H NMR spectrum of **12** in $CDCl_3$ (400 MHz).

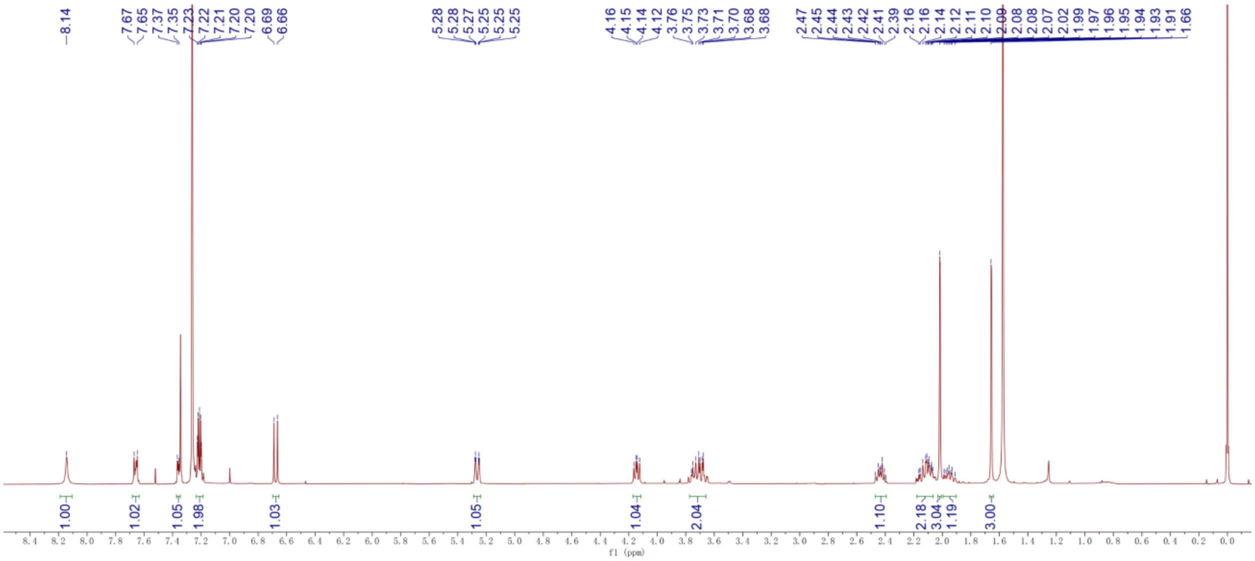

Fig. S52. ^{13}C NMR spectrum of **12** in $CDCl_3$ (150 MHz).

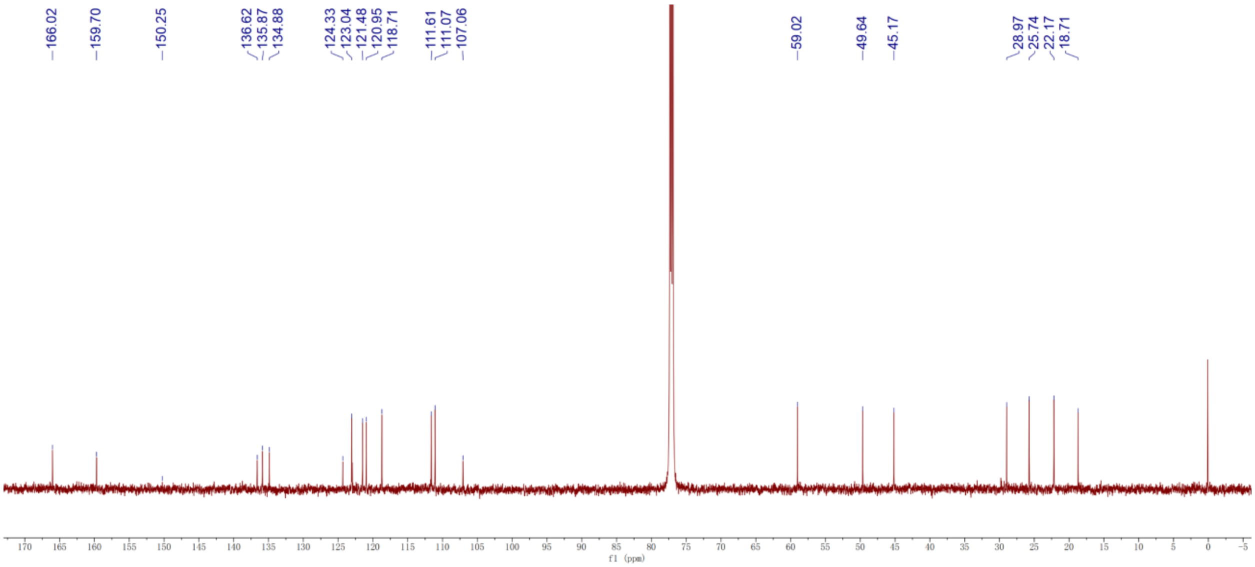

Fig. S53. ^{1}H NMR spectrum of **13** in $CDCl_3$ (400 MHz).

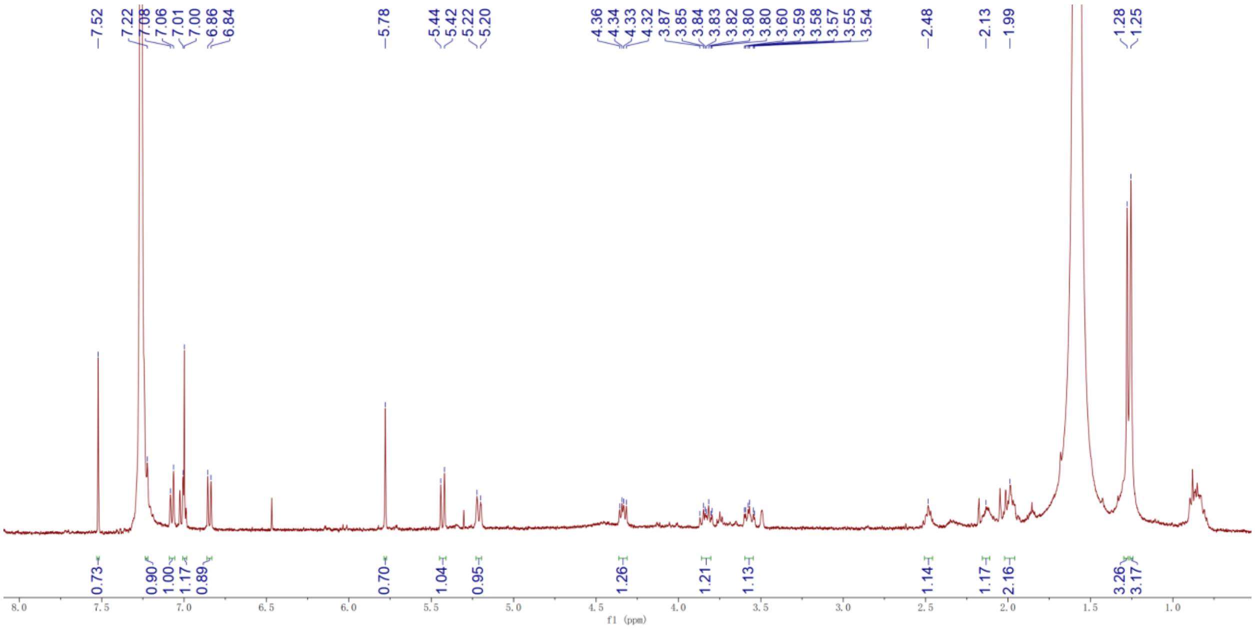

Fig. S54. ^{1}H NMR spectrum of **15** in $CDCl_3$ (400 MHz).

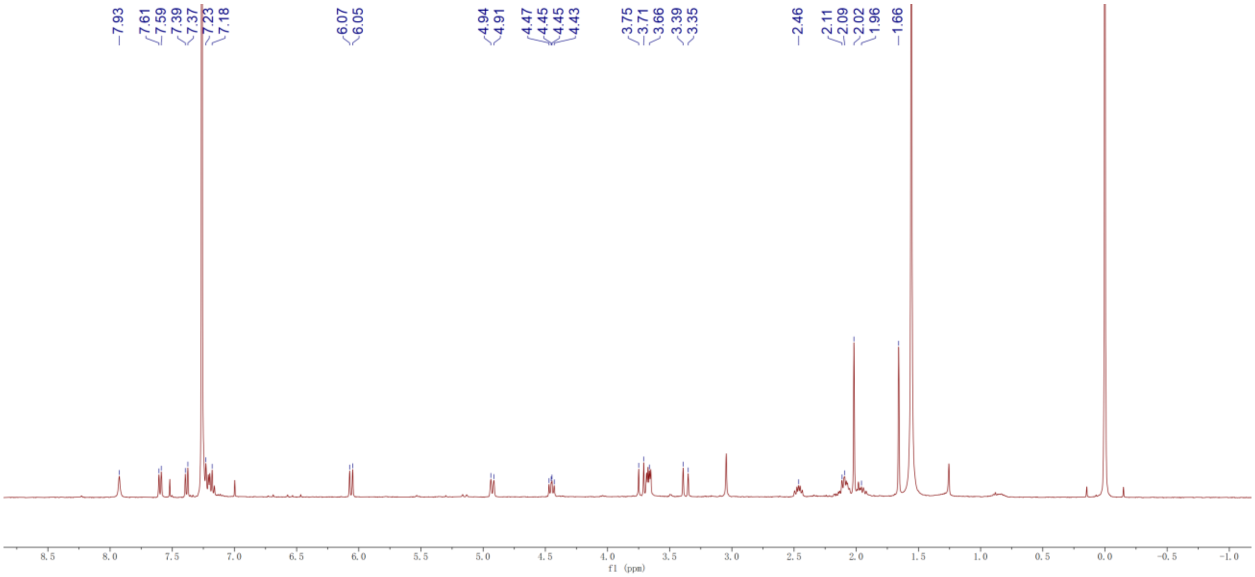

Fig. S55. ^{13}C NMR spectrum of **15** in $CDCl_3$ (150 MHz).

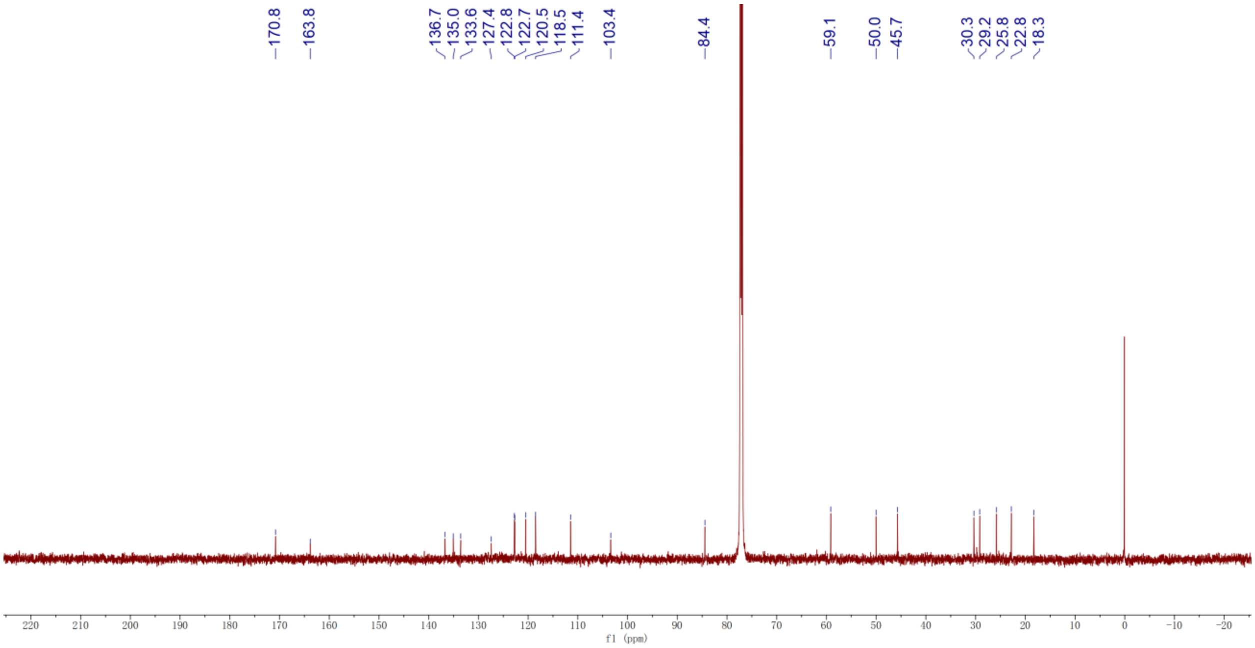

Fig. S56. ^{1}H NMR spectrum of **16** in $CDCl_3$ (400 MHz).

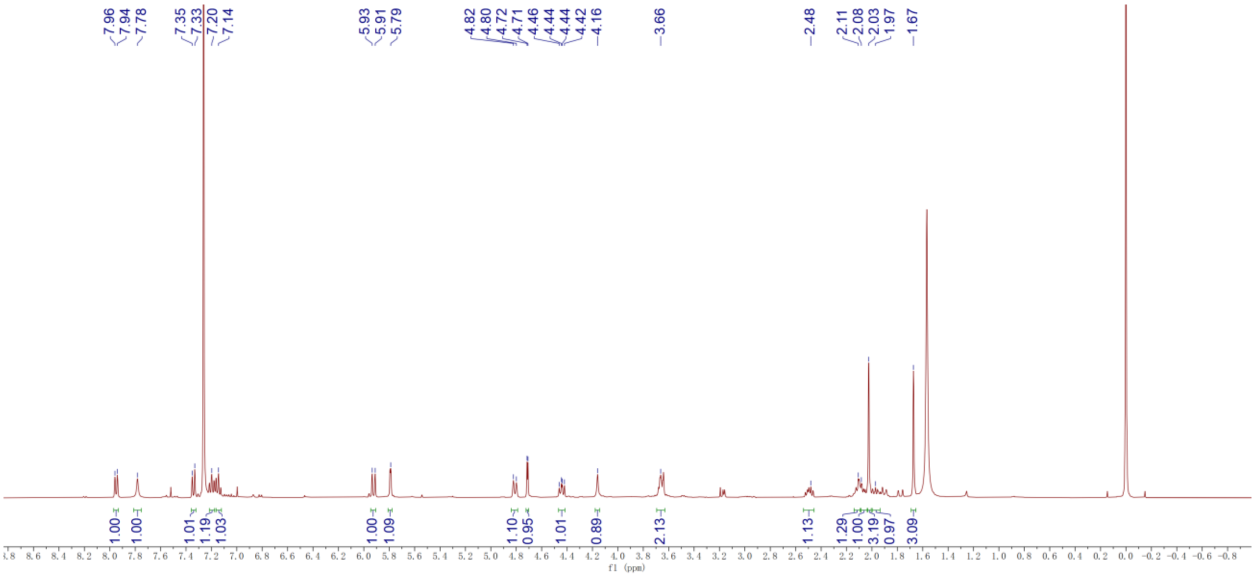

Fig. S57. ^{13}C NMR spectrum of **16** in $CDCl_3$ (150 MHz).

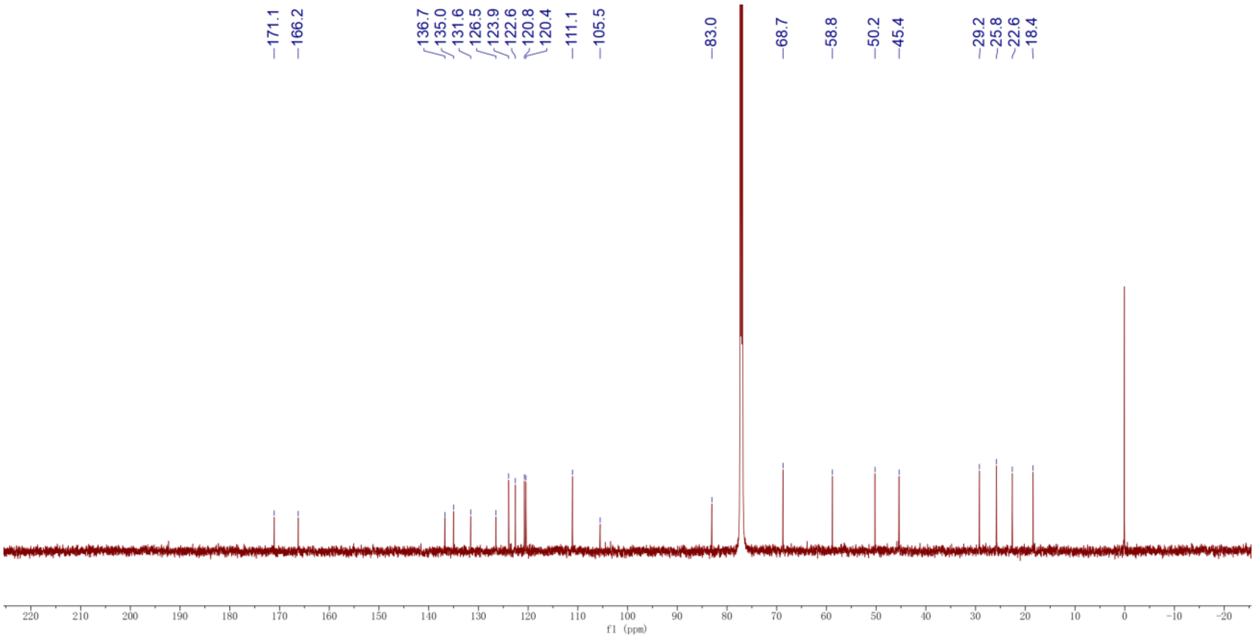

Fig. S58. ^{1}H NMR spectrum of **17** in $CDCl_3$ (400 MHz).

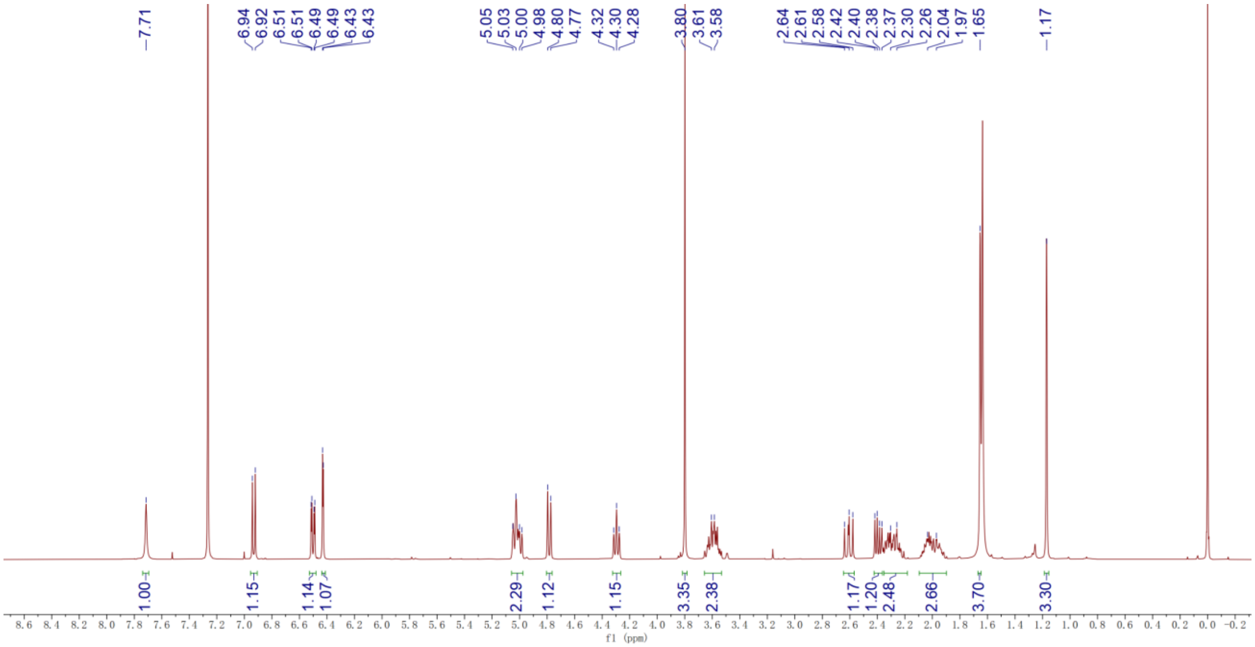

Fig. S59. ^{13}C NMR spectrum of **17** in $CDCl_3$ (100 MHz).

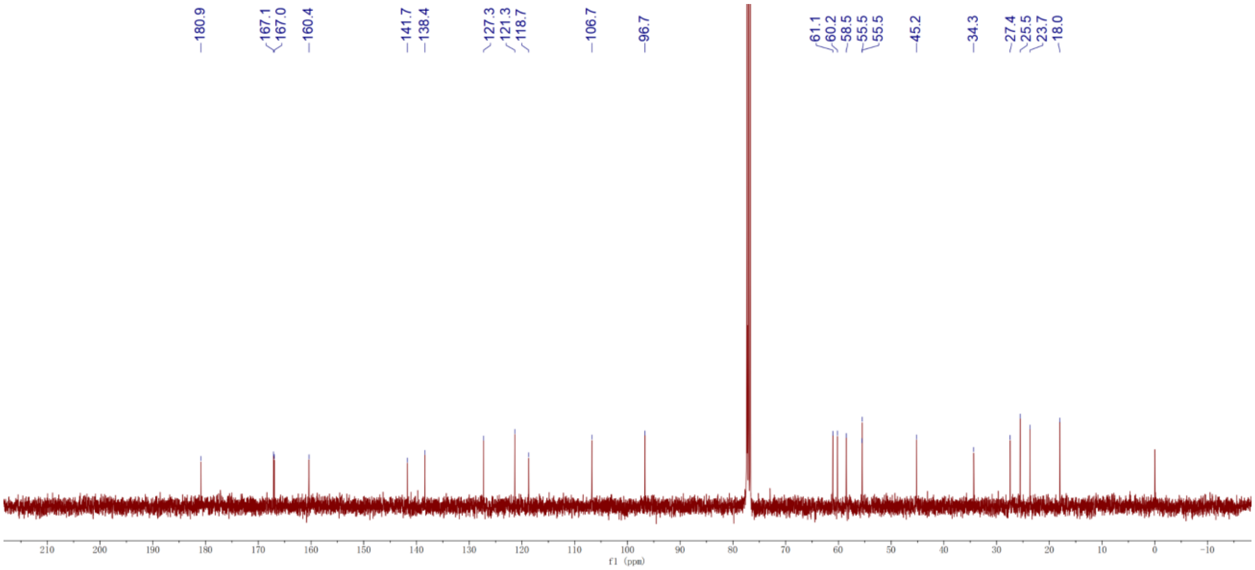

Fig. S60. ECD spectra for compounds **4**, **5**, **9** and **10**.

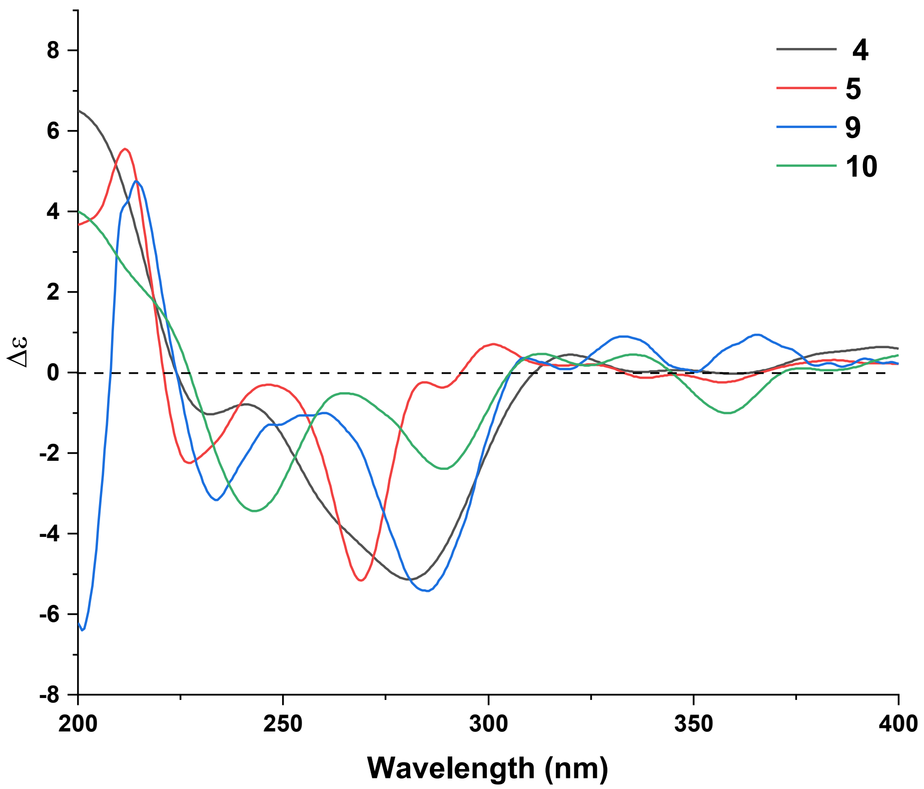

Fig. S61. UPLC trace of OUMDZ-5210 (natural pH) at λ 280 nm.

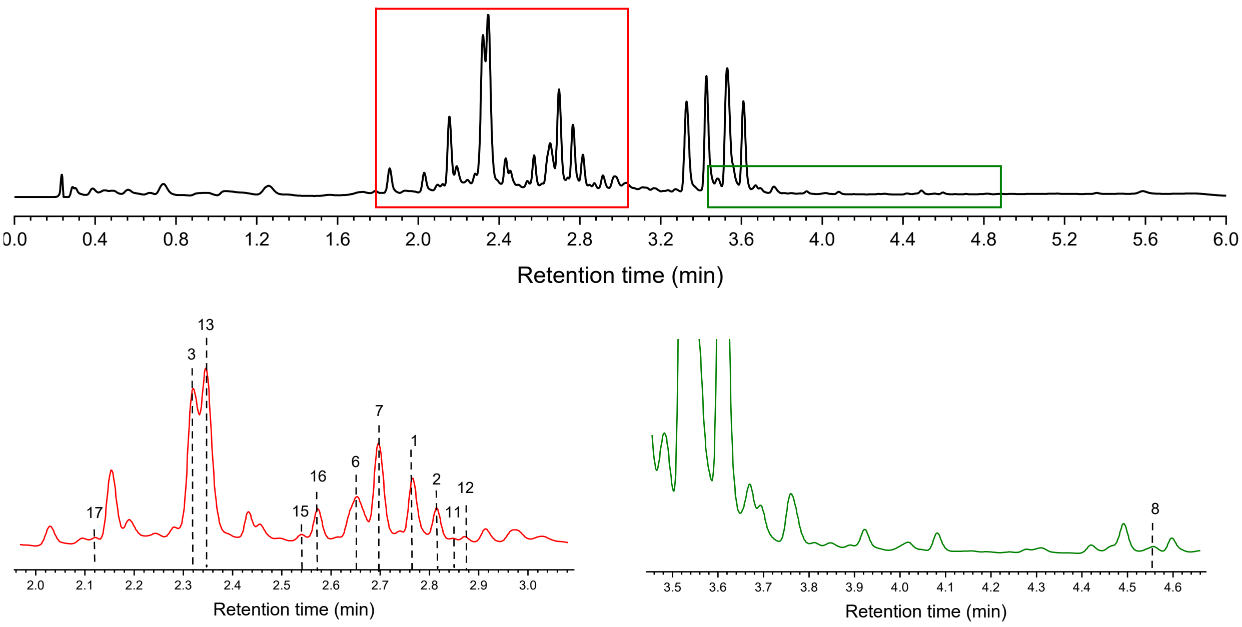

Fig. S62. UPLC trace of OUMDZ-5210 (pH 3.0) at λ 280 nm.

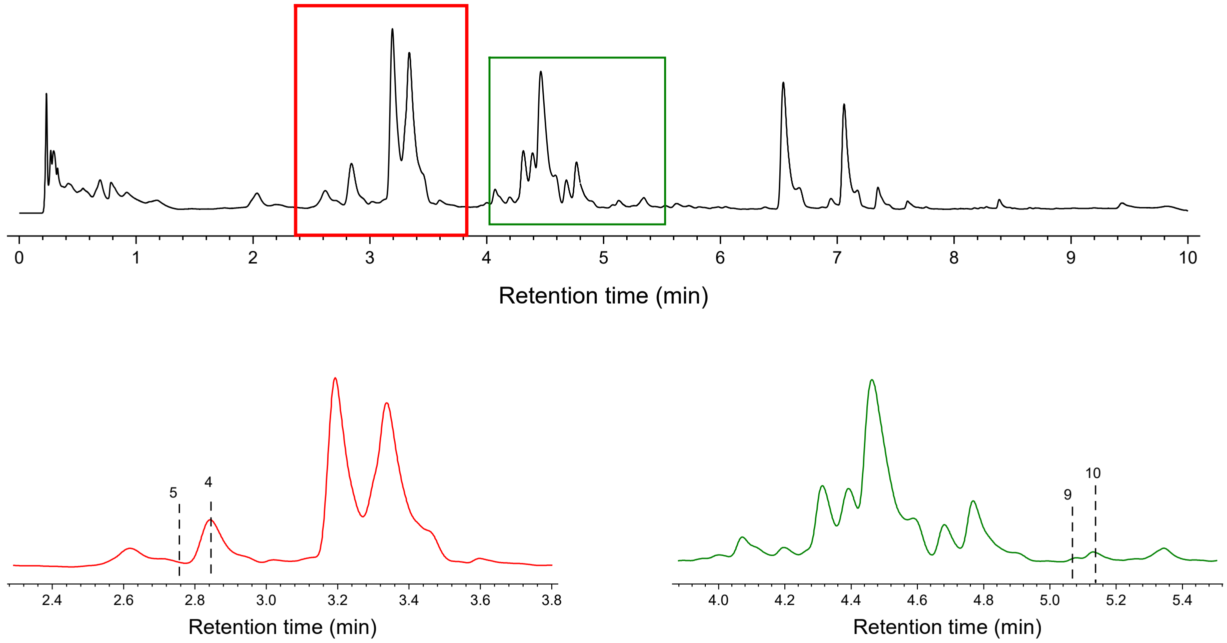

Fig. S63. UPLC trace of Amino Acid residues by Marfey's Method.

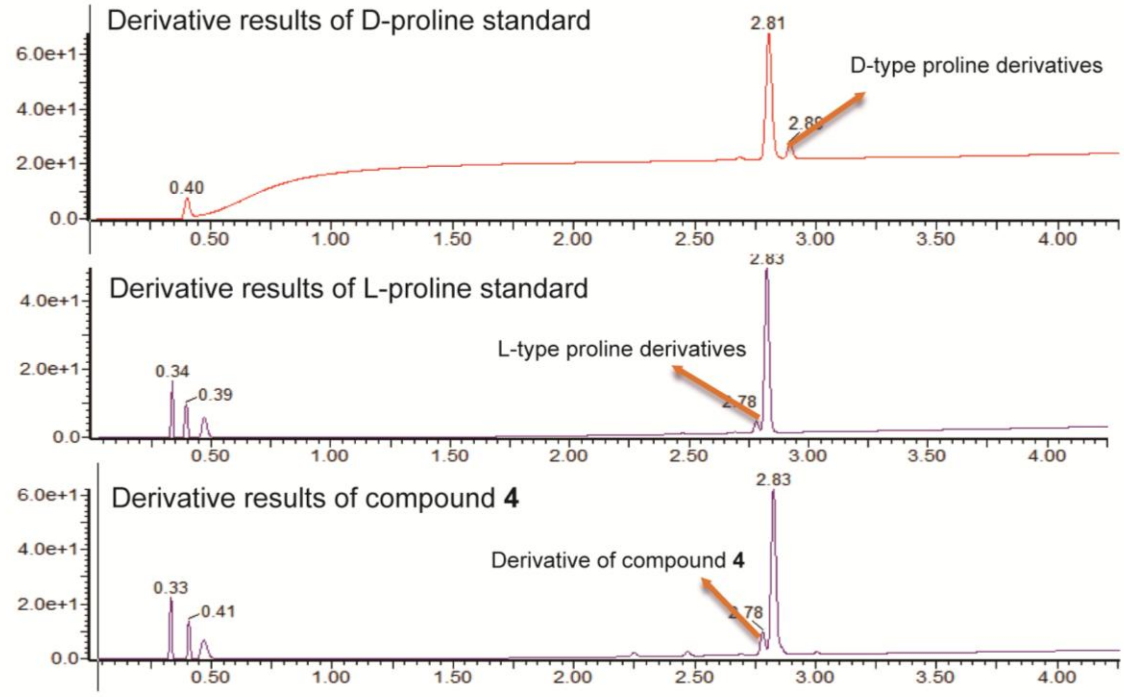

Table S1. NMR data of fumitremorgin C (**1**) in $CDCl_3$.

Position	Compound 1			
	δ_C [1)]	δ_C [a)]	δ_H [1)] (*J* in Hz)	δ_H [b)] (*J* in Hz)
1			7.89, s	7.76， s
1a	132.2, C	132.1, C		
2	51.0, CH	51.0, CH	5.98, d (9.5)	5.99, d (9.6)
3	169.55, C	169.6, C		
3a	59.3, CH	59.2, CH	4.10, dd（9.5, 7.5）	4.11, dd (10.0, 7.4)
4	28.6, CH_2	28.6, CH_2	2.40, m; 2.23, m	2.42, m; 2.23, m
5	23.1, CH_2	23.1, CH_2	2.06, m; 1.94, m	2.06, m; 1.93, m
6	45.4, CH_2	45.4, CH_2	3.65, m	3.65, m
7	165.8, C	165.6, C		
7a	56.8, CH	56.8, CH	4.18, dd (11.5, 5.0)	4.18, dd (11.0, 5.1)
8	21.9, CH_2	21.9, CH_2	3.51, dd (16.0, 5.0); 3.10, ddd (16.0, 11.5. 1.0)	3.52, dd (16.1, 5.5); 3.10, dd (15.7, 11.4)
8a	106.3, C	106.3, C		
9	118.9, CH	118.9, CH	7.43, d (9.0)	7.44, d (9.4)
9a	120.8, C	120.7, C		
10	109.5, CH	109.6, CH	6.81, dd (9.0, 2.2)	6.82, dd (8.5, 2.1)
11	156.6, C	156.6, C		
12	95.3, CH	95.2, CH	6.85, d (2.2)	6.86, d (2.1)
12a	137.1, C	137.,0, C		
13	124.2, CH	124.2, CH	4.91, dm (9.5)	4.91, dt (9.5, 1.4)
14	134.0, C	134.0, C		
15	25.7, CH_3	25.7, CH_3	1.64, s	1.65, s
16	18.1, CH_3	18.1, CH_3	1.99, s	2.00, s
11-OCH_3	55.8, CH_3	55.8, CH_3	3.83, s	3.84, s

a) Recorded at 100 MHz. b) Recorded at 400 MHz.

1) Reported ^{1}H and ^{13}C NMR data for **1**[(8)].

Table S2. NMR data of **2** in $CDCl_3$.

Position	Compound 2			
	δ_C 1)	δ_C a)	δ_H 1) (*J* in Hz)	δ_H b) (*J* in Hz)
1			8.82, s	8.49, s
1a	134.1, C	133.9, C		
2	49.7, CH	49.7, CH	6.64, d (9.8)	6.66, d (9.6)
3	166.00, C	165.9, C		
3a	59.0, CH	59.0, CH	4.11, m	4.15, dd (10.0, 7.4)
4	29.00, CH_2	28.9, CH_2	2.41, dt (11.2, 7.2); 2.11, m	2.44, m; 2.11, m
5	22.2, CH_2	22.1, CH_2	2.07, m; 1.91, m	2.09, m; 1.95, m
6	45.2, CH_2	45.1, CH_2	3.72, m; 3.64, m	3.75, m; 3.69, m
7	159.9, C	159.7, C		
7a	122.6, C	122.6, C		
8	111.6, CH	111.3, CH	7.31, s	7.32, s
8a	106.7, C	106.7, C		
9	119.2, CH	119.2, CH	7.49, m	7.52, d (9.4)
9a	118.6, C	118.6, C		
10	110.6, CH	110.6, CH	6.83, s	6.87, d (2.13)
11	156.9, C	156.6, C		
12	95.8, CH	95.7, CH	6.83, s	6.86, s
12a	137.7, C	137.5, C		
13	121.0, CH	121.0, CH	5.23, d (9.8)	4.91, d (9.52)
14	135.6, C	135.5, C		
15	25.8, CH_3	25.7, CH_3	1.61, s	1.66, s
16	18.6, CH_3	18.6, CH_3	1.96, s	2.01, s
11-OCH_3	55.8, CH_3	55.7, CH_3	3.78, s	3.84, s

a) Recorded at 100 MHz. b) Recorded at 400 MHz.

1) Reported ^1H and ^{13}C NMR data for **2**(32).

Table S3. NMR data of spirotryprostatin G (**3**) in $CDCl_3$.

Position	Compound 3			
	δ_C 1)	δ_C a)	δ_H 1) (J in Hz)	δ_H b) (J in Hz)
1			8.49, brs	7.98, s
2	179.0, C	178.7, C		
3	61.4, C	61.4, C		
3a	118.8, C	118.9, C		
4	128.4, CH	128.5, CH	6.95, d (8.2)	6.95, d (8.5)
5	107.1, CH	107.1, CH	6.51, dd (8.3, 2.3)	6.52, dd (8.6, 2.4)
6	160.6, C	160.6, C		
7	97.1, CH	97.1, CH	6.47, d (2.3)	6.45, d (2.4)
7a	141.7, C	141.6, C		
8	116.9, CH	116.8, CH	5.76, s	5.76, s
9	137.9, C	138.0, C		
10				
11	162.5, C	162.5, C		
12	61.6, CH	61.6, CH	4.34, dd (10.6, 5.9)	4.34, dd (10.0, 5.9)
13	29.2, CH_2	29.3, CH_2	α 2.49; β 1.99, m	α 2.48; β 1.98, m
14	22.1, CH_2	22.1, CH_2	α 2.13; β 1.99, m	α 2.12; β 1.97, m
15	44.8, CH_2	44.8, CH_2	α 3.84; β 3.58, m	α 3.85; β 3.57, m
16				
17	155.1, C	155.2, C		
18	64.0, CH	64.1, CH	5.39, d (9.2)	5.38, d (9.2)
19	120.4, CH	120.4, CH	5.20, dt (9.2)	5.19, dt (9.2, 1.8)
20	138.3, C	138.3, C		
21	25.4, CH_3	25.4, CH_3	1.59, s	1.59, s
22	18.3, CH_3	18.3, CH_3	1.29, s	1.30, s
6-OCH_3	55.5, CH_3	55.5, CH_3	3.79, s	3.80, s

a) Recorded at 100 MHz. b) Recorded at 400 MHz.
1) Reported ^{1}H and ^{13}C NMR data for **3**[(8)].

Table S4. NMR data of **4** in DMSO-d_6.

Position	Compound 4a (Major)		Compound 4b (Minor)	
	δ_C a)	δ_H b) （J in Hz）	δ_C a)	δ_H b) （J in Hz）
1		12.01, brs		11.79, brs
1a	134.2, C		134.1, C	
2	137.8, C		137.8, C	
3				
4	173.3, C		173.7, C	
5	59.8, CH	4.50, q (4.5)	60.4, CH	5.26, q (4.5)
6	28.7, CH_2	α 2.28; β 1.94, m	31.2, CH_2	α 2.32; β 2.01, m
7	25.2, CH_2	α 1.91; β 1.95, m	22.0, CH_2	α 1.85; β 1.89, m
8	49.6, CH_2	α 3.94; β 3.97, m	47.5, CH_2	3.69, m
9				
10	165.2, C		165.2, C	
11	139.6, C		139.6, C	
12	113.5, CH	8.50, s	113.2, CH	8.43, s
12a	129.0, C		129.3, C	
12b	114.7, C		114.8, C	
13	123.2, CH	8.27, d (8.8)	123.5, CH	8.20, d (8.8)
14	110.7, CH	6.94, d (9.7)	110.3, CH	6.96, d (9.7)
15	161.2, C		160.9, C	
16	94.7, CH	7.08, s	94.7, CH	7.07, s
16a	143.5, C		143.5, C	
17	117.7, CH	6.75, s	117.8, CH	6.67, s
18	143.2, C		143.2, C	
19	20.5, CH_3	2.08, s	20.3, CH_3	2.06, s
20	26.9, CH_3	2.08, s	26.8, CH_3	2.00, s
15-OCH_3	55.5, CH_3	3.94, s	55.4, CH_3	3.89, s

a) Recorded at 150 MHz. b) Recorded at 400 MHz.

Table S5. NMR data of 5 in DMSO-d_6.

Position	Compound 5a (Major)			
	δ_C[1)]	δ_C[a)]	δ_H[1)] (*J* in Hz)	δ_H[b)] (*J* in Hz)
1			11.75, brs	11.80, brs
1a	134.6, C	134.5, C		
2	138.5, C	138.3, C		
3				
4	172.6, C	172.6, C		
5	59.6, CH	59.7, CH	4.55, dd (8.5, 4.6)	4.55, q (4.5)
6	28.3, CH_2	28.5, CH_2	*β* 1.87, m; α 2.25, m	*β* 1.89, m; α 2.31, m
7	25.2, CH_2	25.3, CH_2	1.89, m	1.91, m
8	49.6, CH_2	49.7, CH_2	*β* 4.01, m; α 3.89, m	*β* 4.01, m; α 3.90, m
9				
10	166.7, C	166.2, C		
11	141.9, C	141.4, C		
12	113.3, CH	113.5, CH	8.33, s	8.44, s
12a	128.1, C	128.5, C		
12b	114.9, C	114.9, C		
13	122.7, CH	123.1, CH	8.17, d (8.7)	8.19, d (8.8)
14	109.6, CH	109.8, CH	6.88, d (9.7)	6.89, d (9.7)
15	160.4, C	160.7, C		
16	94.8, CH	94.8, CH	7.04, d (2.2)	7.04, s
16a	142.3, C	141.6, C		
17	119.5, CH	117.3, CH	6.79, s	6.78, s
18	142.3, C	142.6, C		
19	20.2, CH_3	20.3, CH_3	2.19, s	2.06, s
20	27.1, CH_3	27.2, CH_3	2.06, s	2.06, s
4-OCH_3	51.6, CH_3	51.8, CH_3	3.67, s	3.66, s
15-OCH_3	55.3, CH_3	55.4, CH_3	3.88, s	3.87, s

Position	Compound 5b (Minor)			
	δ_C[1)]	δ_C[a)]	δ_H[1)] (*J* in Hz)	δ_H[b)] (*J* in Hz)
1			11.55, brs	11.55, brs
1a	134.4, C	134.4, C		
2	138.2, C	138.2, C		
3				
4	172.9, C	173.0, C		
5	60.7, CH	60.8, CH	5.21, dd (8.6, 4.5)	5.20, q (4.5)
6	31.3, CH_2	31.4, CH_2	*β* 1.97, m; α 2.28, m	*β* 1.98, m; α 2.32, m
7	21.6, CH_2	21.7, CH_2	1.83, m	1.89, m
8	47.8, CH_2	47.9, CH_2	3.69, m	3.68, m
9				
10	166.2, C	166.3, C		

11	141.8, C	141.2, C		
12	113.4, CH	113.5, CH	8.43, s	8.39, s
12a	128.3, C	128.6, C		
12b	114.9, C	114.9, C		
13	122.7, CH	123.0, CH	8.17, d (8.6)	8.19, d (8.8)
14	109.6, CH	110.0, CH	6.87, dd (8.6, 2.2)	6.87, d (9.7)
15	160.4, C	160.5, C		
16	94.8, CH	94.8, CH	7.04, d (2.2)	7.04, s
16a	142.5, C	140.8, C		
17	120.0, CH	117.3, CH	6.59, s	6.58, s
18	140.4, C	142.7, C		
19	20.0, CH_3	20.1, CH_3	1.91, s	1.91, s
20	26.5, CH_3	26.6, CH_3	2.03, s	2.03, s
4-OCH_3	51.4, CH_3	51.5, CH_3	3.46, s	3.47, s
15-OCH_3	55.3, CH_3	55.4, CH_3	3.87, s	3.87, s

a) Recorded at 150 MHz. b) Recorded at 400 MHz.
1) Reported ^{1}H and ^{13}C NMR data for **5**[(22)].

Table S6. NMR data of **6** in $CDCl_3$.

Position	Compound 6			
	δ_C 1)	δ_C a)	δ_H 1) (*J* in Hz)	δ_H b) (*J* in Hz)
1			7.85, brs	7.79, brs
1a	132.2, C	132.0, C		
2	50.1, CH	49.9, CH	6.00, d (9.5)	6.01, d (9.5)
3	170.9, C	170.7, C		
3a	59.2, CH	59.0, CH	4.44, dd (9.5, 6.6)	4.44, dd (9.7, 7.0)
4	29.2, CH_2	29.1, CH_2	2.45, m; 2.10, m	2.46, m; 2.09, m
5	22.9, CH_2	22.7, CH_2	2.09, m; 1.90, m	2.09, m; 1.96, m
6	45.7, CH_2	45.6, CH_2	3.64, m	3.64, m
7	164.0, C	164.3, C		
7a	84.5, C	84.3, C		
8	30.4, CH_2	30.3, CH_2	3.65, m; 3.33, m	3.65, d (16.2); 3.32, d (16.2)
8a	103.4, C	103.2, C		
9	119.1, CH	119.0, CH	7.45, d (8.6)	7.46, d (8.6)
9a	121.9, C	121.7, C		
10	110.0, CH	109.9, CH	6.83, dd (8.6, 2.1)	6.84, dd (8.6, 2.2)
11	157.5, C	156.8, C		
12	95.5, CH	95.3, CH	6.87, d (2.1)	6.89, d (2.1)
12a	137.7, C	137.5, C		
13	123.1, CH	122.9, CH	4.90, d (9.5)	4.91, d (9.6)
14	137.7, C	134.6, C		
15	25.8, CH_3	25.7, CH_3	1.64, s	1.65, s
16	18.4, CH_3	18.2, CH_3	1.99, s	1.99, s
11-OCH3	55.5, CH_3	55.8, CH_3	3.83, s	3.85, s

a) Recorded at 100 MHz. b) Recorded at 400 MHz.
1) Reported ^1H and ^{13}C NMR data for **6**[(39)].

Table S7. NMR data of **7** in $CDCl_3$.

Position	Compound 7			
	δ_C 1)	δ_C a)	δ_H 1) (J in Hz)	δ_H b) (J in Hz)
1			7.67, brs	7.79, brs
1a	130.2, C	130.1, C		
2	50.2, CH	50.2, CH	5.87, dd (9.5, 1.2)	5.86, dd (9.6, 1.2)
3	166.2, C	166.2, C		
3a	58.7, CH	58.7, CH	4.42, dd (9.1, 6.6)	4.42, dd (9.8, 6.8)
4	29.2, CH_2	29.2, CH_2	2.49, m; 1.99, m	2.47, m; 1.93, m
5	22.6, CH_2	22.5, CH_2	2.08, m; 1.99, m	2.12, m; 2.06, m
6	45.3, CH_2	45.3, CH_2	3.65, m	3.63, m
7	171.0, C	171.0, C		
7a	83.0, C	83.0, C		
8	68.7, CH	68.7, CH	5.75, dd (2.8, 1.3)	5.75, dd (2.8, 1.2)
8a	105.5, C	105.4, C		
9	121.3, CH	121.2, CH	7.80, d (8.5)	7.80, d (8.5)
9a	120.8, C	120.7, C		
10	109.8, CH	109.8, CH	6.81, dd (8.5, 1.2)	6.80, dd (8.2, 2.7)
11	156.8, C	156.6, C		
12	95.1, CH	95.0, CH	6.84, d (2.2)	6.83, d (1.9)
12a	137.,6, C	137.,6, C		
13	124.0, CH	124.0, CH	4.79, dm (9.5)	4.79, m
14	134.6, C	134.7, C		
15	25.7, CH_3	25.7, CH_3	1.66, s	1.66, s
16	18.3, CH_3	18.3, CH_3	2.01, s	1.99, s
11-OCH3	55.8, CH_3	55.7, CH_3	3.82, s	3.82, s
7a-OH			4.10, s	4.29, s
8-OH			4.67, d (2.8)	4.70, d (2.9)

a) Recorded at 100 MHz. b) Recorded at 400 MHz.

1) Reported ^1H and ^{13}C NMR data for **7**[(8)].

Table S8. NMR data of fumitremorgin B (**8**) in $CDCl_3$.

Position	Compound 8			
	δ_C[1)]	δ_C[a)]	δ_H[1)] (*J* in Hz)	δ_H[b)] (*J* in Hz)
1				
1a	131.1, C	131.3, C		
2	49.0, CH	49.1, CH	5.98, d (12.0)	5.98, d (10.4)
3	166.2, C	166.4, C		
3a	58.8, CH	58.8, CH	4.45, t (8.0, 8.0)	4.45, q (9.8, 6.6)
4	28.9, CH_2	29.0, CH_2	2.47, m; 2.08, m	2.48, m; 2.09, m
5	22.6, CH_2	22.7, CH_2	2.10, m; 1.93, m	2.12, m; 1.96, m
6	45.3, CH_2	45.4, CH_2	3.63, d (8.0)	3.65, q (5.0)
7	170.5, C	170.6, C		
7a	83.0, C	83.0, C		
8	69.0, CH	69.1, CH	5.77, s	5.77, s
8a	104.4, C	104.5, C		
9	121.3, CH	121.4, CH	7.85, d (8.0)	7.85, d (8.7)
9a	120.5, C	120.6, C		
10	109.3, CH	109.4, CH	6.79, d (8.0)	6.80, dd (8.0, 2.5)
11	156.2, C	156.3, C		
12	93.8, CH	94.0, CH	6.69, s	6.69, d (2.9)
12a	137.9, C	138.0, C		
13	123.0, CH	123.1, CH	4.71, d (12.0)	4.71, d (10.0)
14	135.2, C	135.3, C		
15	25.6, CH_3	25.6, CH_3	1.63, s	1.63, s
16	18.4, CH_3	18.5, CH_3	1.99, s	1.99, s
17	41.8, CH_2	41.9, CH_2	4.53, s	4.54, d (6.2)
18	120.3, CH	120.4, CH	5.03, s	5.04, t (6.2)
19	134.6, C	134.7, C		
20	25.7, CH_3	25.8, CH_3	1.85, s	1.85, s
21	18.2, CH_3	18.3, CH_3	1.70, s	1.70, s
11-OCH_3	55.7, CH_3	55.8, CH_3	3.84, s	3.85, s
7a-OH			4.74, s	4.72, s
8-OH			4.17, s	4.03, s

a) Recorded at 150 MHz. b) Recorded at 400 MHz.

1) Reported ^1H and ^{13}C NMR data for **8**[(8)].

Table S9. NMR data of **9** in DMSO-d_6.

Position	Compound 9b (Major)		Compound 9a (Minor)	
	δ_C [a)]	δ_H [b)] （J in Hz）	δ_C [a)]	δ_H [b)] （J in Hz）
1				
1a	136.2, C		136.3, C	
2	140.5, C		140.6, C	
3				
4	176.4, C		172.1, C	
5	64.0, CH	5.06, dd (9.6, 5.2)	61.3, CH	4.62, dd (9.6, 5.2)
6	32.6, CH_2	α 2.30; β 2.19, m	30.4, CH_2	α 2.28; β 2.08, m
7	23.4, CH_2	α 2.01; β 1.98, m	26.4, CH_2	α 1.91; β 1.88, m
8	49.8, CH_2	α 3.89; β 3.78, m	51.5, CH_2	α 3.91; β 3.87, m
9				
10	170.1, C		169.9, C	
11	143.7, C		143.4, C	
12	114.4, CH	8.30, s	114.5, CH	8.30, s
12a	131.7, C		131.2, C	
12b	116.1, C		116.3, C	
13	123.6, CH	8.04, d (8.6)	123.4, CH	8.04, d (8.6)
14	111.3, CH	6.89, d (9.9)	111.2, CH	6.85, d (9.9)
15	162.9, C		162.8, C	
16	94.6, CH	6.94, s	94.61, CH	6.94, s
16a	145.3, C		145.1, C	
17	122.8, CH	6.65, s	123.2, CH	6.70, s
18	142.5, C		142.1, C	
19	20.2, CH_3	1.68, s	20.2, CH_3	1.68, s
20	26.0, CH_3	2.01, s	26.4, CH_3	2.01, s
21	43.9, CH_2	5.12, d (5.6)	44.1, CH_2	5.05, d (5.6)
22	121.9, CH	5.04, s	121.9, CH	5.04, s
23	136.0, C		136.1, C	
24	18.5, CH_3	1.87, s	18.5, CH_3	1.87, s
25	25.5, CH3	1.70, s	26.1, CH3	2.01, s
15-OCH3	56.1, CH3	3.92, s	56.1, CH3	3.92, s

a) Recorded at 125 MHz. b) Recorded at 500 MHz.

Table S10. NMR data of **10** in DMSO-d_6.

Position	Compound 10b (Major)		Compound 10a (Minor)	
	δ_C[a)]	δ_H[b)] (*J* in Hz)	δ_C[a)]	δ_H[b)] (*J* in Hz)
1				
1a	136.5, C		136.5, C	
2	140.3, C		140.9, C	
3				
4	175.1, C		174.5, C	
5	63.2, CH	5.22, dd (8.8, 3.9)	61.6, CH	4.66, dd (8.8, 4.2)
6	32.6, CH_2	α 2.33; *β* 2.13, m	30.0, CH_2	α 2.34; *β* 2.01, m
7	22.8, CH_2	α 1.99; *β* 1.94, m	26.3, CH_2	α 1.98; *β* 1.96, m
8	49.8, CH_2	α 3.85; *β* 3.80, m	51.4, CH_2	α 4.00; *β* 3.89, m
9				
10	168.9, C		168.9, C	
11	142.5, C		143.1, C	
12	115.1, CH	8.47, s	114.3, CH	8.30, s
12a	131.5, C		131.4, C	
12b	116.4, C		116.3, C	
13	123.4, CH	8.07, d (8.7)	123.2, CH	8.07, d (8.7)
14	111.3, CH	6.93, d (8.7)	111.4, CH	6.91, d (8.7)
15	162.9, C		163.0, C	
16	94.8, CH	6.96, s	94.7, CH	6.96, s
16a	145.2, C		145.2, C	
17	123.4, CH	6.65, s	123.5, CH	6.65, s
18	141.5, C		142.1, C	
19	20.0, CH_3	1.61, s	20.2, CH_3	1.75, s
20	26.0, CH_3	2.03, s	26.1, CH_3	2.03, s
21	43.9, CH_2	5.14, d (5.4)	44.1, CH_2	5.17, d (5.8)
22	121.8, CH	5.03, t (5.4)	121.9, CH	5.09, t (5.4)
23	136.1, C		136.1, C	
24	18.5, CH_3	1.87, s	18.5, CH_3	1.87, s
25	25.6, CH_3	1.70, s	25.6, CH_3	1.72, s
4-OCH_3	52.6, CH_3	3.60, s	52.7, CH_3	3.75, s
15-OCH_3	56.1, CH_3	3.91, s	56.1, CH_3	3.91, s

a) Recorded at 125 MHz. b) Recorded at 500 MHz.

Table S11. NMR data of demethoxyfumitremorgin C (**11**) in $CDCl_3$.

Position	Compound 11			
	δ_C[1)]	δ_C[a)]	δ_H[1)] (*J* in Hz)	δ_H[b)] (*J* in Hz)
1			7.92， s	11.02， s
1a	133.5, C	133.1, C		
2	51.0, CH	50.4, CH	6.03, d (9.8)	5.95, d (9.4)
3	169.5, C	169.6, C		
3a	59.3, CH	58.9, CH	4.12, dd (8.0, 7.5)	4.27, dd (7.8, 8.2)
4	28.6, CH_2	28.3, CH_2	2.41, m; 2.24, m	2.44, m; 2.01, m
5	23.1, CH_2	23.2, CH_2	2.06, m; 1.94, m	2.06,m; 1.89， m
6	45.4, CH_2	45.3, CH_2	3.64, m	3.45, m
7	165.7, C	165.9, C		
7a	56.8, CH	56.6, CH	4.19, dd (11.7, 5.0)	4.31, dd (10.0, 5.0)
8	21.9, CH_2	21.8, CH_2	3.57, dd (15.7, 5.0); 3.13, dd (15.7, 11.7)	2.90， dd (15.8, 10.2); 3.5, m
8a	106.4, C	105.5, C		
9	118.3, CH	118.3, CH	7.58, d (7.5)	7.34, d (7.5)
9a	126.4, C	126.4, C		
10	120.1, CH	119.3, CH	7.15, t (7.5)	6.86, dd (8.5, 2.1)
11	122.2, CH	121.4, CH	7.20, t (7.5)	7.01, t (7.5)
12	111.2, CH	111.9, CH	7.34, d (7.5)	7.55, d (7.5)
12a	136.2, C	136.5, C		
13	124.0, CH	125.0, CH	4.92, d (9.8)	4.87, t (9.8)
14	134.3, C	134.8, C		
15	25.7, CH_3	25.9, CH_3	1.65, s	1.59, s
16	18.2, CH_3	18.4, CH_3	2.01, s	1.95, s

a) Recorded at 100 MHz. b) Recorded at 400 MHz.

1) Reported ^1H and ^{13}C NMR data for **11**[(10)].

Table S12. NMR data of **12** in $CDCl_3$.

Position	Compound 12			
	δ_C [1)]	δ_C [a)]	δ_H [1)] (*J* in Hz)	δ_H [b)] (*J* in Hz)
1			8.34, s	8.14, s
1a	135.0, C	134.9, C		
2	49.8, CH	49.6, CH	6.67, d (9.7)	6.68, d (9.7)
3	166.1, C	166.0, C		
3a	59.1, CH	59.0, CH	4.14, dd (9.7, 9.8)	4.15, dd (9.6, 6.9)
4	29.1, CH_2	29.0, CH_2	2.42, m; 2.18, m	2.42, m; 2.14, m
5	22.2, CH_2	22.2, CH_2	2.07, m; 1.94, m	2.08, m; 1.96, m
6	45.3, CH_2	45.2, CH_2	3.72, m; 3.64, m	3.75, m; 3.68, m
7	159.8, C	159.7, C		
7a	123.0, C	123.0, C		
8	111.2, CH	111.1, CH	7.31, m	7.37, m
8a	107.0, C	107.1, C		
9	118.7, CH	118.7, CH	7.38, m	7.37, m
9a	124.4, C	124.3, C		
10	121.5, CH	121.5, CH	7.22, m	7.22, m
11	121.0, CH	121.0, CH	7.18, m	7.20, m
12	111.7, CH	111.6, CH	7.64, m	7.66, m
12a	136.8, C	136.6, C		
13	121.5, CH	121.5, CH	5.26, dt (9.8, 1.4)	5.26, dt (9.8, 1.3)
14	135.9, C	135.9, C		
15	25.8, CH_3	25.7, CH_3	1.64, s	1.65, s
16	18.8, CH_3	18.7, CH_3	2.02, s	2.02, s

a) Recorded at 150 MHz. b) Recorded at 400 MHz.

1) Reported ^1H and ^{13}C NMR data for **12**[(32)].

Table S13. NMR data of spirotryprostatin B (**13**) in $CDCl_3$.

Position	Compound 13	
	δ_H[1]（*J* in Hz）	δ_H[a]（*J* in Hz）
1	brs	7.52, s
2		
3		
3a		
4	7.06, d (7.6)	7.07, d (7.2)
5	6.99, td (7.6, 1.0)	7.00, t (7.1)
6	7.23, td (7.6, 1.0)	7.22, t (7.5)
7	6.89, d (7.6)	6.85, d (7.5)
7a		
8	5.79, s	5.78, s
9		
10		
11		
12	4.35, dd (10.5, 6.1)	4.33, dd (9.6, 6.2)
13	α 2.49, m; *β* 1.99, m	α 2.48, m; *β* 1.98, m
14	α 1.99, m; *β* 2.13, m	α 1.99, m; *β* 2.13, m
15	α 3.58, ddd (12.2, 9.3, 2.9); *β* 3.85, dt (12.2, 8.3)	α 3.57, dt (9.0, 3.5) ; *β* 3.85, dt (9.3, 3.0)
16		
17		
18	5.44, d (8.8)	5.43, d (9.2)
19	5.22, dm (8.8)	5.21, dt (8.8, 1.3)
20		
21	1.56, s	1.28, s
22	1.26, s	1.25, s

a) Recorded at 500 MHz.
1) Reported 1H and 13C NMR data for **13**[(8)].

Table S14. NMR data of **15** in $CDCl_3$.

Position	Compound 15			
	δ_C[1)]	δ_C[a)]	δ_H[1)] (*J* in Hz)	δ_H[b)] (*J* in Hz)
1			7.93, brs	7.93, brs
1a	133.6, C	133.6, C		
2	50.1, CH	50.0, CH	6.06, d (9.7)	6.06, d (9.8)
3	170.9, C	170.8, C		
3a	59.2, CH	59.1, CH	4.45, dd (10.3, 6.9)	4.45, dd (10.3, 6.9)
4	29.2, CH_2	29.2, CH_2	β 2.46, m; α 2.10, m	β 2.46, m; α 2.11, m
5	22.9, CH_2	22.8, CH_2	β 2.09, m; α 1.96, m	β 2.09, m; α 1.96, m
6	45.8, CH_2	45.7, CH_2	3.67, m	3.66, dd (8.5, 5.2)
7	163.9, C	163.8, C		
7a	84.5, C	84.4, C		
8	30.4, CH_2	30.3, CH_2	β 3.73, d (16.5); α 3.37, d (16.5)	β 3.73, d (16.5); α 3.37, d (16.5)
8a	103.4, C	103.4, C		
9	118.5, CH	118.5, CH	7.60, d (8.0)	7.60, d (8.0)
9a	127.5, C	127.4, C		
10	120.6, CH	120.5, CH	7.18, t (8.0)	7.18, t (8.0)
11	122.7, CH	122.7, CH	7.22, t (8.0)	7.23, t (8.0)
12	111.5, CH	111.4, CH	7.38, t (8.0)	7.38, t (8.0)
12a	136.8, C	136.7, C		
13	122.8, CH	122.8, CH	4.92, d (9.7)	4.92, d (9.8)
14	135.1, C	135.0, C		
15	25.9, CH_3	25.8, CH_3	1.65, s	1.66, s
16	18.4, CH_3	18.3, CH_3	2.01, s	2.02, s
7a-OH			n. d.	n. d.

a) Recorded at 150 MHz. b) Recorded at 400 MHz.
1) Reported ^1H and ^{13}C NMR data for **15**[(21)].

Table S15. NMR data of **16** in $CDCl_3$.

Position	Compound 16			
	δ_C[1)]	δ_C[a)]	δ_H[1)] (J in Hz)	δ_H[b)] (J in Hz)
1			7.79, brs	7.79, brs
1a	131.6, C	131.6, C		
2	50.3, CH	50.2, CH	5.92, d (9.7)	5.92, d (9.5)
3	171.1, C	171.1, C		
3a	58.9, CH	58.8, CH	4.44, dd (10.3, 6.9)	4.44, dd (9.8, 7.0)
4	29.3, CH_2	29.2, CH_2	β 2.49, m; α 2.08, m	β 2.49, m; α 2.08, m
5	22.7, CH_2	22.6, CH_2	β 2.11, m; α 1.97, m	β 2.11, m; α 1.97, m
6	45.4, CH_2	45.4, CH_2	3.63, m	3.66, m
7	166.3, C	166.2, C		
7a	83.1, C	83.0, C		
8	68.6, CH	68.7, CH	5.79, d (2.0)	5.79, d (1.8)
8a	105.5, C	105.5, C		
9	120.8, CH	120.8, CH	7.95, d (7.5)	7.95, d (7.4)
9a	126.5, C	126.5, C		
10	120.5, CH	120.4, CH	7.15, t (7.5)	7.15, t (7.4)
11	122.6, CH	122.6, CH	7.20, t (7.5)	7.20, t (7.4)
12	111.1, CH	111.1, CH	7.34, t (7.5)	7.34, t (7.4)
12a	136.8, C	136.7, C		
13	123.9, CH	123.9, CH	4.81, d (9.7)	4.81, d (9.5)
14	135.1, C	135.0, C		
15	25.9, CH_3	25.8, CH_3	1.67, s	1.67, s
16	18.5, CH_3	18.4, CH_3	2.02, s	2.03, s
7a-OH			4.13, s	4.16, s
8-OH			4.71, d (2.0)	4.71, d (2.0)

a) Recorded at 150 MHz. b) Recorded at 400 MHz.

1) Reported ^1H and ^{13}C NMR data for **16**[(21)].

Table S16. NMR data of spirotryprostatin A (**17**) in $CDCl_3$.

Position	Compound 17			
	δ_C[1)]	δ_C[a)]	δ_H[1)] (*J* in Hz)	δ_H[b)] (*J* in Hz)
1			7.64, brs	7.71, brs
2	180.9, C	180.9, C		
3	60.0, C	60.2, C		
3a	118.7, C	118.7, C		
4	127.2, CH	127.3, CH	6.93, d (8.5)	6.93, d (8.5)
5	106.7, CH	106.7, CH	6.50, dd (8.5, 2.4)	6.50, dd (8.7, 2.4)
6	160.4, C	160.4, C		
7	96.7, CH	96.7, CH	6.43, d (2.4)	6.43, d (2.2)
7a	141.7, C	141.7, C		
8	34.3, CH_2	34.3, CH_2	α 2.60, dd (13.2, 10.5); *β* 2.39, dd (13.2, 6.8)	α 2.61, dd (13.9, 10.1); *β* 2.39, dd (13.2, 7.5)
9	58.5, CH	58.5, CH	4.99, dd (10.5, 6.8)	5.00, m
10				
11	167.1, C	167.1, C		
12	61.0, CH	61.6, CH	4.28, dd (8.3, 7.8)	4.30, t (7.8)
13	27.4, CH_2	27.4, CH_2	α 2.31, *β* 2.27, m	α 2.30, *β* 2.26, m
14	45.2, CH_2	45.2, CH_2	α 2.07, *β* 1.97, m	α 2.04, *β* 1.97, m
15	23.7, CH_2	23.7, CH_2	α 3.61, *β* 3.58, m	α 3.61, *β* 3.58, m
16				
17	167.0, C	167.0, C		
18	64.1, CH	64.1, CH	4.78, d (10.0)	4.78, d (10.0)
19	121.3, CH	121.3, CH	5.03, dm (10.0)	5.03, dm (8.0)
20	138.4, C	138.4, C		
21	25.5, CH_3	25.5, CH_3	1.65, s	1.65, s
22	18.0, CH_3	18.0, CH_3	1.26, s	1.17, s
6-OCH_3	55.5, CH_3	55.5, CH_3	3.80, s	3.80, s

a) Recorded at 100 MHz. b) Recorded at 400 MHz.
1) Reported ^{1}H and ^{13}C NMR data for **17**[(8)].

Table S17. UPLC Conditions.

Chromatography Columns	C18 column, 2.6 μm, 2.1 × 50 nm
Mobile Phases	A aqueous phase: 1 ‰ HCOOH/H_2O<br>B organic phase: 1 ‰ HCOOH/MeCN
Flow Rate	0.4 mL/min
Elution condition	1 10-100% MeCN/H_2O，6.0 min<br>2 10-100% MeCN/H_2O，10.0 min
Sample concentration	0.5 mg/mL
Injection volume	1.0 μL
Detection wavelength	Full-wavelength scanning

Table S18. UPLC elution method 1.

Time (min)	Flow (mL/min)	% A	% B
0.00	0.40	90.0	10.0
0.50	0.40	90.0	10.0
4.00	0.40	5.0	95.0
5.00	0.40	5.0	95.0
5.10	0.40	90.0	10.0
6.00	0.40	90.0	10.0

Table S19. UPLC elution method 2.

Time (min)	Flow (mL/min)	% A	% B
0.00	0.40	90.0	10.0
0.50	0.40	90.0	10.0
6.00	0.40	50.0	50.0
8.00	0.40	5.0	95.0
9.00	0.40	5.0	95.0
10.00	0.40	90.0	10.0

Table S20. Medium composition.

Name of culture medium	Ingredient / L
Fungus No. 2	Maltose 20 g, mannitol 20 g, MSG 10 g, glucose 10 g, yeast extract 3 g, corn syrup 1 g, KH_2PO_4 0.5 g, $MgSO_4 \cdot 7 H_2O$ 0.3 g, tap water 1L, pH 6.5
Fungus No. 2 of sea water	Maltose 20 g, mannitol 20 g, MSG 10 g, glucose 10 g, yeast extract 3 g, corn syrup 1 g, KH_2PO_4 0.5 g, $MgSO_4 \cdot 7 H_2O$ 0.3 g, sea water 1L, pH 6.5
PDA	Potato 200g, glucose 20 g, agar 20g, tap water 1L
PDB	Potato 200g, glucose 20 g, tap water 1L
SDYS	Yeast extract 6g, Casein enzymatic hydrolysate 3g, Casein acids hydrolysate 3g, Sucrose 10g, tap water 1L
YPD	Yeast extract 10g, peptone 20g, dextrose 20g, agar 15g, tap water 1L
YPDS	Yeast extract 10g, peptone 20g, sorbitol 1M, dextrose 20g, agar 20g, tap water 1L
BMGY	Yeast extract 10g, peptone 20g, dextrose 20g, potassium phosphate buffer pH 6.0 100mM, YNB (containing ammonium sulfate) 13.4g, biotin 0.4g, glycerol 10g, tap water 1L
BMMY	Yeast extract 10g, peptone 20g, dextrose 20g, potassium phosphate buffer pH 6.0 100mM, YNB (containing ammonium sulfate) 13.4g, biotin 0.4g, methanol 5g, tap water 1L

Table S21. Primer name and sequence.

Primer name	Sequence (5'→3')
789-S-F	CATGCCTGCAGGTCGACGATAGGCCGACGGGTCTTTTACCAAGTC
789-S-R	AGAATGCACAGGTTTGGAAGCGTGGGCTGTTGCGGAAT
789-hph-F	CCACGCTTCCAAACCTGTGCATTCTGGGTAAACGACT
789-hph-R	CGATTTCATCTCTATGATCAGGCCTCGACAGAAGATGA
789-X-F	AGGCCTGATCATAGAGATGAAATCGTACATACCGCCA
789-X-R	CCCGGGGATCCTCTAGAGATAGTGCATCGTATGGAATAGATCCGG
789-yz-F	TTCTGACGATAGACCCGCATGCGCT
789-yz-R	GACGTCTCTCTGCTTATGGCAACAT
792-S-F	CGAGCTCATGCTCGGCTGGAGAACCATGAAAC
792-S-R	CGGGGTACCACGTCCCTTCTGTGCTATTTCGCAG
792-hph-F	ACGTGGTACCCCGAACCTGTGCATTCTGGGTAAACGACT
792-hph-R	TTTAAATCTCCGCTGATCAGGCCTCGACAGAAGATGA
792-X-F	GCGGAGATTTAAATGTCCAACTCGCGGTTGAAGCTCTCGA
792-X-R	ACGCGTCGACGTTCCTCCTTGGTAATTGAATCTCTA
792-yz-F	CATGCACCACACGCTGTCGTACAAT
792-yz-R	AGGAGAACGGCTGCTGGCGGACAAA
794-S-F	GCGGAGATTTAAATTTTTGCCTAAGGTGGCGTATGATCC
794-S-R	ACGCGTCGACGGGCTTTCTCTTTGAGCAACCATGT
794-hph-F	GCCCGTCGACGCGTCTGTGCATTCTGGGTAAACGACT
794-hph-R	CTCAAGGTACCCCGGATCAGGCCTCGACAGAAGATGA
794-X-F	CGGGGTACCTTGAGGATAAAGGCACCGTCCTCTT
794-X-R	AGGGCCCAGTACAGAGATACGGAGAGCCCTTT
792-yz-F	CTGTTCCTTGATTTCTCAACTGCGG
792-yz-R	GCGATGAAGCGAAACTCATTCTGTG
794-9k-F	AGAGAGGCTGAAGCTTACATGGAAACCCTCGATGCGGTTCAGC
794-9k-R	CGCGGCCGCCCTAGGGAACCCATGCGGTGCAGTTTCAGTC
alpha-Factor-F	TACTATTGCCAGCATTGCTGC
3´ AOX1	GCAAATGGCATTCTGACATCC
9K-F	TTCCCTAGGGCGGCCGCG
9K-R	GTAAGCTTCAGCCTCTCT
9k-794-F	AGAGAGGCTGAAGCTTACgccaccATGGAAACCCTCGATGCGGTT
442C-A-1-R	GGCATGCTTGCCGTGGCCAAAAGCG
442C-A-2-F	GGCCACGGCAAGCATGCCGCCCCGGGCCGCTTCCTGGCCTGCG
9k-794-R	CGCGGCCGCCCTAGGGAACCCATGCGGTGCAGTTTCAGTCCCC
292R-L-1-R	GCAGCTGCGCCATGGTAGCATCATA
292R-L-2-F	CTACCATGGCGCAGCTGCTAATTATTGCCGTTGCTTTTCAGACT

Table S22. Boltzmann distribution of secofumitremorgins.

method: m062x/6-311+g(2d,p) scrf=(smd,solvent=dmso) em=gd3			
Conf.	G(hartree)	ΔG(kcal/mol)	Pi
**4a**	-1316.92174	0.00000	0.84
**4b**	-1316.92016	0.99272	0.16
**5a**	-1356.18466	0.00000	0.66
**5b**	-1356.18403	0.39251	0.34
**9a**	-1316.92016	0.95871	0.17
**9b**	-1316.92174	0.00000	0.83
**10a**	-1551.37326	0.16830	0.48
**10b**	-1551.37353	0.00000	0.52

Table S23. Cytotoxic activity test.

Cell	Concentration (μM)	Dox. (10 μM) inhibition±SD (%)	inhibition±SD (%)
A549	20	3.51%±0.96%	90.64%±0.52%
MKN-45	20	-6.59%±2.93%	74.25%±1.20%
HCT 116	20	7.42%±1.90%	81.83%±0.60%
HeLa	20	5.87%±3.65%	98.94%±0.23%
K-562	20	5.01%±3.66%	58.87%±2.19%
786-O	20	0.10%±2.08%	82.50%±0.97%
TE-1	20	2.03%±2.54%	68.76%±2.08%
5637	20	1.31%±2.52%	98.75%±0.56%
GBC-SD	20	6.80%±1.95%	84.09%±0.40%
L-02	20	2.43%±2.57%	97.45%±1.04%
HOS	20	1.94%±2.53%	97.99%±0.59%
HepG2	20	-5.38%±1.22%	81.06%±1.14%
SF126	20	-2.03%±2.99%	97.97%±0.82%
DU145	20	-5.36%±0.41%	79.04%±0.27%
CAL-62	20	6.43%±2.67%	89.46%±2.13%
PATU8988T	20	-0.79%±6.03%	98.94%±0.42%
MCF7	20	8.40%±1.09%	41.76%±0.58%
A-375	20	-12.53%±0.27%	95.28%±1.55%
A-673	20	-3.56%±2.30%	97.57%±0.84%
293T	20	44.62%±1.72%	94.66%±0.47%
MV-4-11	20	4.20%±2.15%	30.12%±3.05%

Table S24. Gene sequence and numbering in OUCMDZ-5210.

Gene Name	Nucleotide sequence
0787	ATGGCTCTAGATGGTCGTTCGCCTTCTCCTTCTATCTCGGACCTAGAGTGTG<br>CGTGCCTTTACTTTGCTTACGGAAGCAACATGAACCTTACGCAAATGGCGC<br>AAAGATGTCCCGAAAGCGTCATCTTTGCGAAAGGAGCACTTTGCAACTATA<br>AATGGCAGACCAATAGCCGTGGTGGCGGAAATGTTATACAGGGAAACACC<br>GAGGACGTCGTGCACGGTATTGTCTTCATCATTTCCTCATCTGAGGTTGAAG<br>CTTTAAGGCGTTACGAAGGGGTCGACAAGCAATTCTTCGTTGAAAAAAAGC<br>TTGTTATTGAGCTAGAGGCCATCTCAGATCCGAGGTTTAGCCGTCAGAAGA<br>CTGCTGATGTGGTAAAAGTGCTGGCAACAAAGGATTCTGACTATGGGAAAC<br>ACACCATTTTAGCCACAAAGAGGCCGACCAGCTCAGATGAGATTTGCCAGG<br>TTGAAACTAGACTTTCAGGTACTCTCACTTTAAAGAGATGGGATCTTGTAG<br>ACATACTGACAGATCATGCTTAATTAGGATCCCATGAAGCCTTGGTCTACA<br>TCAGCACCCCCATGTACCGCTCGCCCAGTGCAATTACGAATGAGTATAGCA<br>GGAGGATGCAACAGGCAATAGCAGATGCGAGATTGCTTGGCTTGTCTGAG<br>GATTATTTGACATACACGTTAGGTCCCCTCATCTCCCGCAAGGAGACAGAT<br>GCCTA
0788<br>(*ftmA*)	ATGGCGATGGCTCTTGCGGTAGGCGCACCCTCCGAACAGAGAGGTAATCTT<br>CCATACAATACGCTCAAGGACGGTCCGACGGTGGACAGCATGAAAGCAAC<br>GACAGACGGCAAGAATGGACAGGGTGAATCCGCATTTTGGGACACTTGCG<br>TACATACCGTCTTTCGTGAGCATTGCCAGCGAGCTCCAAACTCACCCGCAG<br>TCAATGCATGGGACGGATCATTCACCTATGCCGAGCTCGACAGCCTGTCGG<br>ATGCCATTGCGTCAGTCTTGATCCTCTCTGGAGTCGGACCAGAGAGCATTA<br>TACCCATATACATGCAGAAGTCCCGATGGACAACGGTTGCAATATTGGGCG<br>TTCTCAAGAGCGGAGGAGCATTCACGCTTCTGGATCCATCCCATCCACGGA<br>GCAGGGTGGAGGAGATATCTAAGGAAATCCAGGCAAGGTTTATCCTTACG<br>AGTGAGAAACTCAGTAAACAGTGTTTGGAGATGTTTTCTGTCCTGGTCGTG<br>GAGCATCTAAGCCGGGCATGCTTGCCGAGGCCCGGCCAAGCTGGCCACAC<br>ACGATCTCGACCTGAAAACGCCGCTTATATCGCGTTCACGTCGGGTTCGAC<br>AGGAAAGCCTAAAGGCATTGTCATTGAGCATCGATCATATTGCTGGGAGCT<br>CGTTCACACCTGAAGGTATTTGGCATCATTCGACGTCGCGCGTTCTTCAATT<br>TGCCTCGTATGCCTTCGATGTCAGCATCATGGAGACGCTCAGTACTCTAAT<br>GGCGGGCGGCTGTTTATGCGTAATGAGCGAGTCGGAACGAAGCGACCCAA<br>ACCTCTTCGTTGTGTCTTATAAGAACCTACGGATCTCCCACTGCTTTATGAC<br>CCCATCCTTTGCGAGGACGGTGCCATGGACAGAATGTTGCAACCCGCCACC<br>CACCCTGATCGTGGGAGGCGAGTTGATGCGCCCCTCCGATGCAAGAGCATA<br>CAAAGAAATGGGAATTCGCTGCATGAACGCATACGGGCCAGCCGAATGTT<br>CCGTCAACGTGTCTGTACAGTCTAGGGTGGAGGCTGCGGTTGATCCTCGGA<br>ACATCGGGTACACCACAGGAGCGACAGCCTGGATTATTAGTCCGGAGAAC<br>CCAGAGGAGCTGATGCCAACCGGTACAGTCGGGGAACTCCTCGTGGAAGG<br>GCCCATTGTTGGTCGCGGCTATCTCAACGATCCCAAAGCCACTCGCCAGGC<br>ATTTATTGATACACCCGCATGGCTGCGCCGGCACCGCAAGGGCACCTCTTA<br>TCAGCACCGAGTCTACCGCACCGGGGATTTGGCCTCCCTGGACAGCATAAC<br>CGGTGCGCTGCTGCTACACGGCCGCAAAGATGCTCAAGTGAAGATTCGGG<br>GCCAGCGGGTCGAGCTCCCTGATATCGAACACCATCTCCAACTGACCCTCC<br>CGAACGACAATGCCGAGGTCATTGTCGAAAAGGTCACCTTTTCCGACGATG<br>GATCGGAAAAGCTCATTGCCTTTGTCTTAGTCCGTCCATCTAACACTGATTC<br>CGTCATAGGCAACACGGGAGATCGTCTCTTCCTGGCGCCGCAATCGCAGAT<br>CATGGAGCAATTCGCCATCAGCAAGAAGCATCTACAAACACATCTACCCAG<br>CTACATGGTTCCAGATATATTCATTCCTATATCAACTCTCCCTCAAACTGCA<br>TCCGGGAAAACAGACCGCAAAGCCTTGAGAACCCGAGCTGCTGCCCTCTCG<br>AGACGGGACGTTCAATGTTTTCTACTCTCGCCCGCGGGTGGTAAACGTCCT<br>CCATCCACACCCAAAGAAGCAACCATCCGAAGTCTGTATAGCAATGTGCTG<br>AACCTACCCATCGACTTGATAGGCATGGACGACACTTTCCTGCGCCTCGGG<br>GGGGATTCACTCCAAGCCATTCGCCTCGTTGCAGCGGCCAGGGCGGCCGGG

	CTTATACTACACGCCAAGGATATCCTTTCGTCGCAGAGTACGCTGGCTGAG<br>CAATCAAAACGTGCAGGTTTGATCCAAACAATAGACCGTACATGGGAATCT<br>TCACCTCCGTTTGCCCTCTTGCATGGGCCGACGAGGCATGCTATTGTCGACC<br>TGGCGCAGAAGCAGTGCCGTGTGCCTTCGAATCTCATTGAAGATATCTACC<br>CTTGTACTGCACTGCAGGAGGGCATGTTCATAACGTCTTTAAAACACCCAG<br>GCATGTATACCGGGCAGATAATATTTGATATTCCCGACAGAATGGAGTTAC<br>CCCGTTTGAGGGCCGCGTGGCTGTCGGTGGTGTCCGAGAATGCTGCTTTGC<br>GAACACGTATCATTGAAACCCACGAGGGACTCATGCAAGCGGTGATCGTC<br>GATGACTTCGTGTGGGAGGAAGAAACCGACGAGATGCTCCTCTCTAGCGAC<br>GGGGAAGCACTCGAGATCACCAAGATCGGTGTGCCTTTGGTTCGGTTTCGC<br>TATCGCCCTCGACATCGGCAGCTGATGATGACAATCCACCACTCAATCTGG<br>GATGGCTGGTCGCTCCGCTTGGTTCATGAGCAACTACATCGTGCCTACATT<br>GGGCGGGATCTGTTACCGAGCACCTCATACCGCTCTTTCATTCAATACACG<br>CAAGAGCTACAAGGGGCAGACGAGTTCTGGGCCTCCGAGCTCGCCGGCGT<br>GAATGCACCGATATTCCCGACCTTGCCATCTGGTAACTATCGGCCGCGCGT<br>AAACGCATCGCATCGCCATGTGGTGAGAAACTTGGCCTCAACCGGAAAGG<br>AGGAGCATACCGCCGCTACCTATATTCACCTTGCCTGGTCGTTACTCGTTGC<br>CCACTATACAGACGCGGACGAGACCGTATACGGGGTGACAGTCAACGGGC<br>GTAGTGCTGACGTACCGGGGGTTGAGAATATCGTGGGTCCAACAATTGCAA<br>CCGTCCCGACGCGCATTCGAGTCAACGAGGAAGATACCGTGGAGATGGCG<br>CTGGACCATGTCCAGGACGCTCTCGCACGCATGATTCCCTACGAACAGGCT<br>GGCCTGCAGCGCATCAGCCGGTGTAGTAGAGATGCCTCCGAAGCTTGCCGC<br>TTCCAGACTTTGCTTATCATTGAAGCTCCCACAGATAGTGACGTGGATTGTG<br>AGAAGAACGAAGCTGGAAACTTTTCCATTATCGGGGGAACGACACAGACT<br>GGGATGGACTACACCGCGTTCTCGTCATACGCGATGATGCTCGTCTTTCGT<br>ACTAGCGCTAACAAGAGCGCGATTTCGTTCGATATTACATATGACGCGCAA<br>GTAATAGGCCACGATGAGGTGGAGCGAATGGCTCATCAGTTCGAGCATGTC<br>TTGCGACACATCTATACGCTTGCAACAGGACGGATTGGAGATATTAGCTTT<br>ATCGGGCCACGGGACATCGAGCAAGTACAACAATGGAACAGTACCATGCC<br>CCCAGCCGACAATCGCTTCTTGCAGGAGTTGATTTTTGCTCAGTGCTCCCGT<br>CGACCTCAGGCGTCTGCTATTATTTCCTGGGATGGCTCATGGACGTATCGG<br>GAACTCTGGGCACACTCATCGTTCTTTGCGCGGCAACTACAGAGGTATGGG<br>GTGACCCGAGGAACTCCTGTAGCTGTCTGCCTGGACCGCAGCAGGTGGAGC<br>ATTGCAGTCATCCTGGGCGTTCTCCTCGCTAGAGGCACATGCGTGCTGATC<br>GATTTACTGGCTCCGCGACAAAGGGTGCGAGATATCCTTCAGATCGCCGGC<br>ACAGGTATCTTGGTCCACAGTCATGCAACTGCCACGCTGACGTCCGGTCTC<br>TGTCCCACTGTTGTCAATGTATCCTTCCTTGCGGCCCAGAGCGACTCTTCGC<br>AGCCGGAATTTCCCTTCACTTTGGAGACCTGGGGCGGAACCCCGGAAGACC<br>TCGCATTTATCATCTTTACGTCCGGTAGTACCGGTCATCCCAAAGGCATTGA<br>GATGCCCCATCGCACACTCTCGACGAGCATTTCCCATCACAGCGCGGGGAT<br>GAGAGTTACATCCAGCAGTCGTGTCCTGCATTTTTCCTCATACGCATTCGAC<br>GTGAGCATCTATGAAATCTTCACCACATTGGCTGCTGGTGGAACCATCTGC<br>GTCCCCTCCGAGTTTGACCGCATGAACAATCTCGCAGGCTTTATTCAAGAC<br>ACCCAAGTCAACTGGGCGTTTTTAACGCCTTCCACGGCTAGAAGCCTCAAC<br>CCCGCAGACGTTCCCTTGCTAACTACACTAGTTCTGGGAGGTGAAGCTGTG<br>ACCCACGAGAGCGTTGAGGTGTGGGCAAAAGGCCGCTCCCTCATCAACGG<br>CTATGGCCCTGCCGAGGCAACGATCTGCGGAGTGGGCAATATCCCCGAAGC<br>GGGATGGAAATCCGGCGTTGTCGGTCGAATCATTGGTGGTTTGGGCTGGGT<br>TACTGTGCCATCAGACCCGAACAGACTGGCAGCGGTTGGCGCCGTGGGTGA<br>ACTTCTCCTCGAAGGCCCCTTTCTGGCCCGAGGATACCTGAACCTGCCAGA<br>GGTCACGAAGGCCGCTTTCATTGATCCTCCTAGCTGGAGAACACGGATTCC<br>TGCTCCGTCGCCCTATTCTTTCCTCTACCGAACAGGCGACCTGGTCCGATAT<br>CAGCCGGACGGATCCATTCAGTATGTCGGTCGTAAAGACAGCCGTGTCAAA<br>CTTCGAGGTCAGCTTGTCGATCTGGGTGCAGTCGAGGCCAGTGTCATGAGA<br>GTGTATCCGGCTGCGGGTCAGGTAGTCGCAGACGTGTTGGTCTCAGAGAAT<br>ACTGCCAGACTGACTGCGATGGTGAAGCTCGGCCCCTCAGTGACTGAGAAC

	CACGATGGCCCTATGTTTGCGGCCCCAGACTTGGTTTTCAACGAGGCTGCT GCCTCTATCCAGGCTCGTCTTCGGGCCATTGTACCTGCTTATATGGTGCCGT CCATGTTCATTCCTCTACGGCACATCCCTCGCACCCTCACAGGCAAGACGG ATCGTCGTCGGCTCCGAGACAAAATACTCTCATTATCACATAGCGACCTCC AACGCTACATGATGAGCTCCTCGACCAAGACACCCATGTCTGATGACAATG AACGGAGGCTTCAAGAGATTTGGGCAGAGGTGCTTCAGCTCCCATGCGAA GCAATTGGGAGAGAGGATTCTTTCCTATCTCTTGGTGGTGAATCTCTGGCG ACGATGAAAATGGTAGCATTGGCAAGGAGGGTTGGGTTCATGTTTGCTGTG ACAGACGTTATGAATAACACGAGCCTGTCAACCCTTGCGCGGTCACGGCAC TTGATCACAGAGCAGGCCATTCTGACCTCCTCACCCTCTCTCTCACTGCCAA CCATTGAAGGCGAGTCCCTCCAGGAGATTCTTCGGCCATTACTGAACGCCG GCCACATCCAAGGAGGCAATGACATTGCTGCTATTCATCCTGTCACCGCTG CACAAGCATTCCTCGTACAGAGGTATCCGTGGTCCCATTTCCAGTTTGACTT GTCTGGCGCTGTCTCGCCCAGCAAGCTCCAAACCGCCTGCACCGCGCTGAT GGCCCGATTTACTATCCTTCGCACGGTATTTGTCGAACATGCGGGCTGTCTC TTGCAGCTTGTCTTGCGGGAGGTGCCCAATCGTGTCCATGAGATTACGACC AATGAGCCTCTTGATGACTTCTGCAATTCAGTTTGTCAGCAGCAGCAGGAT GTCTGTGTTGTTAACTCTACAACCCTGCCAACTCTGTTTACTCTCGTGTCCA ACCGCCAACTAAACAGACATCGACTTCTGCTCCGCCTCGCTCATGCACAGT ACGATCTCACCACAATCCCTCTAATTGTCCAATCCTTGGCCGACGAATACA ACAGAACCCTCCGTTCGGGTTTCTCCGCCGACTTCGGCTACTATCTCAGTCA TCATAAGCGGCAGAATAACGACGACAGGTCTCATAACTTCTGGAAGCGAT ACCTGTCAGGCTCCTCTATGATGTCTACGAATCAGACTGCAGATCCGACGA CGGTTCAAGAGCGCGTATTTCACGTCACAGGATCATGTATAATTATACCCA CATCACATCCCCCGGACATAACAATTGCCACCGCCGTGAAAGCCGCCGTCT GCCTGGTTCTAGCCGCAAGGACTGGCTGCAAAGATATCGTCATCGGTCAAA CAGTGGACGCCCGGTGTAGTTCGGCTGACAGCACTCTTGACCAAATTGTTG GCCCCTGCACGAACTATATCCCGTACCGGCTCAGCGTGTGCTGTTCTAAAA CTGCGCTGGAATACCTCCGCAGCGCGCAAGCCCAGCACACAACATGCCTCC GATACTCTTCCCTGGACTTCGACCAGATCGTGGCCAAGTGCACCAGCTGGC CAAGCAGTACTCAATTTGGATATATCGTCCAGCACCAGGACACTGGCGCAG AGTTGGCTCTCACGCTGGGAGGTGACACTACTTCCTTGCCGATGACTTCTTA CGGCCGTGTATTTCCTCAGGGTGAGGTCTGGATCGGTTCGACACCCTGTTCT ACTGGTCTGAGAATTGATGTCATTGCTCTGAGCGCAGTGCTAAGCCAGAAG GACGCTCAGACTATGGCTGAGGAGGTCGGCGCGGCGTTGGAGAAATTGCTT GGGTGCGGTTATCGCCGTCTTTCGCATCTGATAGGAAATACCTTCGCTACCT AA
0789 (*ftmC*)	ATGAAACCGAGTCACTCTGATACTCCCCTCATGATGCCATCGGTCATGAAG TGCGGGTATCTGGCGACGGCTGGCCTAATTGGCATTTGCACCCATCTCTCGT ATTTCCGATATGGGGAACACCATCTGTATCCCTGGCGGTATGTACGATTTC ATCTCTGCCTGACCATGGGCGTGGCAGCCCTACTATATGCCAAAAAGCCAC CCCAGTATACTCTTAGTTCAATGGACCTCGTCAAAGACGTCTCTCTGCTTAT GGCAACATATCTCGTTGGACTGTTCGCGTCGCTCCTCCTCTACCGAACATTG TTCCACCCCCTCCGCCAGATACGTGGCCCCTGGGCAGCCAAGATATCTTCA TTCTGGTTATCCTTCCGTCTGAGGCGTGGGCCATCATTCCGGATCCTTCATG AACTGCACGAGGAATACGGCCCCGTCGTGAGGGTTGGACCCTCTGAAGTGT CTATCATCCACCCCGAAGCCGTCCGGATGATCTACGGACCCAACTCCCGCT GTTCAAAGAACACCTTCTACGACAACGGCCATCCGATGATGTCGCTACATT CCTACCGGGACCGAATAGCCCACGACCAGCGCCGCCGGGTCTGGAGCGCG GGGTTCGGGGATCGGGCTCTGCGTGGTTACGAGCAGCGCATGCGGGTCTAT CGTCAGAAGCTCTTCCAGCGCTTGGAAGCGTGGGCTGTTGCGGAATCGGCA ATCAATATCAGCCAGTGGTTCAATTTTTACAGCTATGATACCATGGGCGAC CTGGCCTTTGCGCGCAGCTTTGACATGCTGGACGCAAGCCGGAATCATTGG GCGGTAGACATGCTCATGCATGGCATGATCGGGTATCGATATTTGTTTCCC AGCTGGTTCTTCCGACTACTCGCTACCATGCCCTCGTTGTCGAGCGACTGGC ATAAGTTCATCGGGTTTGCGACAGACACCATGCTGCGTCGCGTGGGTGTGG

	GTCGCCCTCTTCCTGACCCATCTGCCTGCCGTTTCTTCCGCTGAACATTGGT<br>TCTGTAGGAGCAAGTGGATGTTCCGGATATCTTCGCGTCCTTGCTGGCGCC<br>GCTAAATGGCCGGGAGCCTACCGAGGACGAACGCAACATGCTGATGGGAG<br>ATGCCATGCTCATCATCACAGCAGGAAGGTGAGTTGCTGCCCAGGCCAGAC<br>ACATTGGGATCCAAGTGGACTGACCACCAATGGATCGCCACGTCAATAGTG<br>ATACCACAGCAACGAGCCTGACGAGTATCGTGTACGAATTAGCCCGCCATC<br>TTGATGAGGTCGACAAACTACGAGCGGAGCTGGATCCTATCGAAGCAGAT<br>AGTGACGGCGAGTACCAGCACGACACCCTCGCCAAACTACCACATCTCAAT<br>GGCTTTATCAACGAGACCCTACGGTTGCACCCTCCCATCCCCGGTGTCATTC<br>CGCGAAAGACCCCACCAGAGGGGATCCATGTGAAGGATGTCTTCATTCCGG<br>GCAATATGACGGTGTTCAGTCCGCAATGGAGTATGGGTCGATGTACGAGAA<br>CCTTTCTACACTCGCTGAACTTGTGACATTTGACGTCTGACTCCGCACTTCT<br>CCCACCTTAGCCGAAGCGGCGTACATTGATCCTGAGATCTTCAACCCAGAA<br>CGGTGGTACAAGCACATGGACTTGGTAAAAGACCCGTCGGCCTTTGCCCCG<br>TTCTCCATCGGTTCGTACATCCGCTCAGATCTTGCGAGGTCATTATAATCGT<br>CACTCACTCTCTCTTTCAAATTGCTAGGTCCTTATTCATGTATTGGAAAGCC<br>ACTCGCCTTGATGAATATCCGCACAACGGTGGCTCGTCTGATCATGAGCTT<br>CGACGTACGGTTTCCAGAGGGCGAGGATGGGATCCGGTGGATGGACGCTG<br>CGGATGAGCACTTCGCCATGGGTATACATCAGATGCCGGTCGTTTTGACCA<br>GACGCCATTA
0790<br>(*ftmD*)	TTACTCGCAGATCCAACTGAGCATACTAGCATGGCTCCCAGGCGGAGTATA<br>CAATTTCTCAAACCGTAGCCGACTGTCACAATCTTCCACCAGCCGGCGCAT<br>TTGAGCCAGACTTCTCTCCTTGGCGGAGAAGAGCGTGTACATCCCAATATC<br>ACTCCGTCGCAACCGTCGCTCACTCTGCAGAGACCCTTCACCAGGCTCCGG<br>CAAGACGGCATCCATAATGAGCAGTCGCGCACCGGGCCGTAGAGCGGAGG<br>AGATCTGCCGGACGATTCGAGTGCAATCCGCATCGTTCCAGTCGTGGAGAA<br>TGAGACGAAGGAAGTAAGCATCTGCGATGCGCGGCTGGGGCGTAAAAAAA<br>TCATGCTCCTCGAAGATAATGTTTGGGTGTCGATCTCCCCGTTGACGAGCCT<br>TCTCGATGATTTCGGGCCGGTCTTGGACGGTGATTTGGATGTGAGGAAAGG<br>CATTGACAATGACTGCGGCGACGTGCCCTTCTGATCCTCCGATCTGAAAGA<br>CATGTGAGTAAAAAGAGTAGAACGGTTGTTGGTGTACAGCAACTCCCTTGA<br>CGAGAATAGCGGCCTTAGTAACTTACATCGACCAACGATTGAACCGTGCCC<br>CAGTCATAGGCGCCCGCTACGTGACGTACCATCGAGTCATTATGAACCGCC<br>ATCCCATCGTGCCATCGTTTTCGATTTACCGGGTCTTTCTCCAAGACCTCGA<br>AGAACGTCGGACCCTCGCGAGCCCCGGGCTCCCTGGCCATCAGGGCCAATG<br>GACCCTGGCGCGCCGCTTCGCCGCTGGGGTCCAATTTGATGGACTCGTATA<br>TGAACGAGCAGGGCCGAATGCCAAAGTCTGCCATGAAGCGAGCCTGACCG<br>GCAGTGTCCGGGTCATTCACGAAGATGGCGGACATGGGGCTGTGCTTCACG<br>GCACCCTCTGCATCTTCATCGAGGAATCCAACTAATCGGGTGAAGCGAATC<br>AGACGCTGAAGCAATTCCGGATCCACAGGAGTCTTCAGCTTCTTGACCAGT<br>GCATCGTATGGAATAGATCCGGTCAACGGGACCTGATCTGGGACTTCAAGC<br>CGGATCAAAGTCTGGATCGTGCCGATGTCGACTGCCTGTGTCA
0791<br>(*ftmB*)	TCAATTGGGGAACGACACATCCCCGACAGGGTTGAAACTGTGGTTGTAGAG<br>CGTCACGTAGGCACCGCCGTTCTTGTAGGCAAAGGAGACATAGGCGCAAA<br>GGTGCGCGGCCTTCTCAAAGTCCTCATCGGGGCTATCAGAGTATTGCATTA<br>ATGAGACTCCCCCTCGAAGCAGTAAATCCCGGGGAGGAACTGAAACTCAC<br>TAGTTCGCTAGAAAATTCGCCTGGTAGTGACTGGCCATCTCCTCCCAGCCA<br>ACCCTCCGGTAGAAGGTAGTTAGCCCCTCAACAAGCTTCCGCGAGTTCATC<br>CCAAACACACAGACATACATCTGGGGATCCGGAAACGGCGACTGACTCCC<br>GTCCAGGTGGAAGTGGAACATCATAGGCGCCTCGAACCCGGCCGACGACT<br>TCCCAAGCTCGCAGAAGCCCCGCGGCATGGTGTAGTAACCCTCGCGCATCT<br>GGATATCAGCCCAGAAGTGCCGGAGCAGTTCCAGACCCCTGAGTGCATCCG<br>GATCTCTTCGAAGGCCCCCAAAGGTCCAGATATCCTCCACCATCTGCAGAT<br>TCACATGCCGCTCGCTGGCGTAGAACTTCACCCGCGATTTGCCGGGTTCGA<br>CGAGGTCGGTGGACAGGAAATGAGGGAAGAACGAACACGCGTCGAACGCC<br>TTGTCTGCTGCGGTGGCCGGTGGCTTGTCCGCCGTGACCCCTGGGACATGG

	AGGCCCCGGCGACGCCGCTCGATGTACCGTTGCAGATTGGCCAGCTGCGTC TCGAAGCGCCCCTCGCGATCTACCTTGCGGATGGCATTGACCAGAAGATCC TCCGTCGAGCGGCCAGTCACTGCAGATTTGGGCTGCGGGTAGAAATACGCC TTGACCAGGACGGTCCCGGATTTCTGCAGGTCCATGGCGGTGAGGATCTGC GATTTCCAGGGGCTCCTGAACAGGTCCGGGTTGGCTTGGAGGACGGCTTCC TCTGCCTTGGTGATCACCAGCTCGGTCTCAAACTCGGAGAAGCGTGTCAAG TCAACCCCCGGCAGGAGCCGGGCGGCGGTCTCTAGATAGACCTTCGGACGC GTGGTGTTGAATGGGTCTTGTGCGGTGCCAGAGTCAGCCGTGACGGGATCA ACCCCAATTCGAATCAACGCCCGAGCGACGTTGTTGCTGAACTCGATGGGC AACCCGGTAAGAACCATTCCACTGCGCCATTGCGTCGCAATGATCCCCGTT TCCGGGTCCCGCTGGGGATAGGGTCCCAAGGCGGGGAGGACCAGGTGTCG ATACAGCGACAGGTACTTGTATTGTACGTCAACGTCGTAGTTGGAGGATAT GAGAAGCCTTGAGAGGATGGGCGCTGTGCTATGCCACCAGCGTTCCTCGTC AACACTTCCAAAGAGAATGCTTTCACTGAGGGCCCGGTACGGGGCGGGCT GCAGCTGGTGGCAGGGCTTCTGGTCCGGTGGTGCTGGCGGCATCCTTGCGT GCTGAGGAATCAGGAATAGTGACTGCAAATTGTAGTGGGAATCCTGCCTTT CGGTGGGATACTTCAGATGAGATGGAATTTTATCCAACCTCCGAGGGCATG CGAGCAGACGCCAGAGACGGCGATCCCGAGGCGCATGTAAGACACCCACA TCCTCTCTATGGTCGTCTCATTTTTGAACAATGCACGCCTCGACAGACTAGC TGCAAACATGCATATAATCTGATTTTCATGCATATGCACACTAATGCATACT CGTCACAAAAATGCATACAGTCTCATTTTCAAGCAAAGCATACTAGTTACA AACA
0792 (*ftmE*)	ATGGAGCGACTCCCGCTGTCCCCCGCTGTGCTCTTCCTCATCATCGCCCTCC CCATTCTATACCTTTGGATTCGATACACAGCACCCGCCAGGCCCCATGGGA AGCACCTTTCCCTCCCCCCGGGGCCTCCACGACTCCCTAAGATTGGCAACC TCCACCAGGTACCCCGCCAGATCCCATGGAAGAAGTACAAAGAGTGGTCC GACACTTACGGCCCCATCATGTCCGTCCAACTCGCCGACACCATCGCCGTC GTCTTCTCCTCCTGGGACCTCATCAAAAACCATATTGAACGCCGGAACACA ATCTACAGCAGCCGACCCTCCGTGCCCTTCTTCCTCCACGCGACAGGTGGC CTGAATGCGTCGATTCTTCCCTACGGTCCGGAATGGAAACTCCAGCGGGCC ATCCGCAGCTCCGTCCTCAAACCCTCCATGACGGTCAAGTACCGCGACGTC CAGCATGTGGAAACGACGCAGCTCCTGCACGAGCTTCTGTCCACAAACGAC TTCCCCGTTTGTCTGCGTCGATGTATTGCCAGTGTCTTTCTGACTGTGGCGT ATGGGGAGCGATGTGTCGATCATGCCGGGTTGGAGGCGATTGACCGGCTCG AGGAGCTCAATCGCGCTATCGCACTGCATGCGGAGGCGCTATTCTCTGGTG CTGCTGGTATTCTCACCCAGTTGGTTCTTCCCAAAGCACTGGTCGATCGGCT GCCGGTCCGGTGGAAGAAGGACGCTGATATGCTGCACAATCGGCTGACGG CTGACCTGGTGGCACGGACGAGGGCTGCGCTGGTCCGACCGGGCTGGAATT GGGTCAAGGAATTCTCCATGAAGGACGGAATTGGGAGTGGGGATGGCGAC GGGGAGCAGGGAAGTAAAGTCGGGTTGAAGCGTCTCGCCTATATGGTCGG GTCGCTGTATGAGGCCTCCATGGCTGCGTCACAGGCACTCCGAGTTATCAT CCTCGCTGGTCTCTTACACCCGGATGCCACGCGGCGCATGCACGACGAGCT AGACGCGGTGGTCGGGACGGCCGCCTTCCGGACTTCCACGATGCGGCCCA ATTGCCCTATACGCAAGCCTTCATCAAAGAAGCCATGCGTTGGCGCTCCCT AACACCCATGGGGTCACCCCGTGCCACATCCGACGAAGACGACTGCAGAG GCTACCATATCCCATGTGGCGCTACTGTCCTCGTCAACGTGTGGGCCATAA ACCACGACGAGGCAATATTTCTCGACCCCTTCGCGTTCCAGCCAGAGAGAT GGATCGAGAACCCAGACTTGCCGCAGTTGATGTATGGAATGGGCCAGCGC GCGTGTCCGGGGCGCCACATGGGCCAGGATTCGTTGTTCCTGGCCACTGCT CGTTTGTTTTGGGCGTTCGACATGGCCCTGCCGGATGGTGCGGACCCGATT GACCAGGAGCGGTTCTTGGATAGTGGGACGACACTGGCCGCGTTCCTACCG GACTTTGAAGTGAGATTCACGCCGAGGTCGGAGAAGTATCAGGAGGTCATT GAGAATAGCATGGCTGTGTTACCGGATGTCTTGTCGATTTCTGCGACTCCGT A
0793 (*ftmF*)	ATGACCGTCGACTCCAAGCCACAACTCCAGCGCCTCGCCGCCGATGCCGAC GTTGACCGGATGTGCAGGCTCCTCGAAGAGGACGGTGCCTTTATCCTCAAG

	GGCCTGCTGCCCTTCGACGTCGTCGAGAGCTTCAACCGCGAGTTGGACGTG<br>CAAATGGCCATCCCACCTCCCAAAGGAGAACGGCTGCTGGCGGACAAATA<br>CCCCCCTCACTTCAAATATGTCCCGAACGTCGCCACCACTTGCCCGACGTTC<br>CGCAACACCATTCTCATCAACCCGGTCATCCACGCCATCTGCGAGGCCTAC<br>TTCCAGCGCACGGGTGATTACTGGCTCAGCGCGGCCTTCCTCCGCGAAATT<br>GAATCGGGAATGCCCGCCCAGCCGTTCCACCGCGACGATGCCACCCACCCG<br>CTGATGCACTACCAGCCCTTGGAAGCACCGCCGATTTCCCTCAGCGTCATC<br>TTTCCCCTGACAGAGTTCACGGAGGAGAATGGTGCCACAGAGGTCATCCTC<br>GGCAGTCATCGGTGGACAGAGGTCGGGACGCCGGAGCGTGACCAGGCAGT<br>TCTCGCGACTATGGACCCGGGCGATGTGCTCATTGTACGACAGCGTGTGGT<br>GCATGCGGGCGGTGGGAATCGCACGACGGCCGGCAAACCTCGACGGGTTG<br>TTCTCGCGTACTTCAATAGCGTCCAGCTGACCCCATTCGAGACCTACCGGA<br>CGATGCCGCGCGAGATGGTCGAGTCGATGACTGTTCTCGGCCAGAGGATGC<br>TCGGCTGGAGAACCATGAAACCGTCCGATCCCAACATTGTGGGCATCAACC<br>TGATAGATGATAAGCGGCTGGAGAATGTGCTGCAGTTGAAGGCTGCTGATT<br>CGCCGGCTTA
0794<br>(*ftmG*)	ATGGAAACCCTCGATGCGGTTCAGCTGCCCTACCTCGGGGTCGTCGGCGCA<br>AGCTTGATCGTCATCCTGGGCATCATATTGTTGTTTCCCTTGGGCAGCGACC<br>CCTTTATAACCATCAACCAACACCCATGGGACTTGTTTCAGACAAAGGCCA<br>AGCAACAGTTTGAATATAATGCAGCTGCTCTACTTAACGAGGGCCTCCAAA<br>CAGTAAGCGCAGAGATCCGGTGTAGCTTCCGTATCTTCGCCAACAGGGGTT<br>AACATGCGTCCTGTAGGGCCACTCGGCGTTCCGGCTGGTGACCAACATGGT<br>CACCTATTTGATCTTGAAAGACCAGTATGCGGAGGAAATCAAGAATGATAG<br>TCGGTTTGGCGCTCATGAAGCAGTTGATCCCGTATGTCGCCCTCTTCATTGC<br>GCAGATTTCTCGCAGAACGATCTGGTTCTCATCCGTCTAGGTATTACTTGTG<br>GACCTGCCTGGCTTAGAGAGCATGTTCCAAGGTTCGCTGCATAATCAAGTG<br>CCCCCCATGGCGGTTCGTGCGTTAAACAAGGAGCTTGGTTAGTTTCCAGCC<br>TCTTCTCCTCGGGGAATTATTACTGAGCCCGGAATGTAGTACACTTGACAC<br>CTTCCCTTTCCGAGGAAGCTATGAACTGCCTGCAAACCAGGTGGACGGACT<br>CGACAGGTACGTGCGTTCCCGGTAGGCCAGTTGGACGACCGTCTGATCACA<br>GACAGAGTGGCATGGCGTCTCTATCCCGGAAACCGTACTGGCCTTGATTGC<br>CCAAATGACAACAAGAGCACTGCTGGGCCCTGAGCTCTGCCGCAACCCCG<br>AATGGCTGGACATTGCGAAATCCTTCACCACAAATCGAGCTATTGCTGTCG<br>CCGCCGTCCAGTCCTGGCCCAGCTTTCTTCAACCTGTGATCCATTGGTTCCT<br>GCCCCCGTGCCGGGCTCTTCGCCGTCAAATCCAGTGCGCCCGTAATATCAT<br>CCTTCCGGCCCTCGAGCGTGAACGGCGCGCGTATTGCAGCGATCAGCCAAC<br>GAAGCGGGAATTCTCGAACTTGGTGTTCATCGATCAGTATGCCAAGGGAGC<br>CCGCTATGATGCTACCATGGCGCAGCTGCGAATTATTGCCGTTGCTTTTCAG<br>ACTACGAGTGATTTGGTCGAAAAAGTGATAGCACGACTTTGCAAGCACCCC<br>GAGCTCATTGAGCCCTTGCGCGAGGAGGTGGTTTCGGTAGTTGGGAATCAT<br>GGCTTGCACAGCCACTCATTACGCAAACTCACGTTGATGGAGAGTGTGATG<br>AAGGAAACCCAGCGGCTTGAACCCGCTGTGATCAGTACGTTGCCTCCGAAT<br>ATAGTTATCTTCCTCCGTATGGGTCTCGTGCTGACTTGACGACCGATCTCAG<br>TCGGCATGTTCCGCCTCGCCAAAGAAAAAGTGACGTTGAAGGACGGCACT<br>GTCGTTCCCAAGGGTACCAATATCGCCTTTGCGAATGATCTCCGCTTCGATC<br>CGGAAATGTACCTGGAGCCGGAAACCTTCGATGGCTACCGGTTCCAGCGGA<br>TGAGAGAGGATCCTGCGAAGATTGACCTTGCGCCGTTCACCAAGACCAGG<br>ATGAGCCATCTCGCTTTTGGCCACGGCAAGCATGCCTGCCCGGGCCGCTTC<br>CTGGCCTGCGATGAAGCGAAACTCATTCTGTGTCATATTCTCCTCAATTACG<br>ACATTAGGGCTGTGGAGGGATCCCCTCCGGAATTGCGTGCGCGTGGGATGT<br>TTGTGCAGCTGGATCCTGGGGCAATGATGTCGGTGAGAAGACGGCGGGGG<br>ACTG
0795<br>(*ftmH*)	TCAAAAAGGCGAATAATAAACACTCAGGTAGGGCTTGGAGTGGCGATACG<br>AGAAGGAAACCCCCGAGTGAATGTAATTGGTTTTCGTAAAGTCAGGGCCG<br>GGGCTGTCAACGAGGTTAATTCCATTCCAGCCCGTTGCTGCATGCACCCAC<br>CAAACAGGATCACTTACAAGTATGAGGCCACTTTGTCCTTGTACGTGCTGG

	CCAGATCCGTCCAACCCAGAACCTCGTAGAACGCCACGAGTGCGTCCATGA<br>CGACGGCGTCATTCATCCCAACGGTCAGGAGGTATATCTGGGGGTCCGGAA<br>ATTTTTTCTTGGGAGATAGGGTCCAGTTCGCCATCATAACGGGACGGTAGT<br>CCTCGGGAGGAGGCGTCCCAAAAGCAAAGTCCGGCCGGATGTTGCTCCGA<br>TACCCTTCAGGGATCGCGAGGAGATCCCACAGTTTCCTCAGGAGGGCCAGA<br>CCATCGGCACATTGAGGATCTTCCAGACGTTTCCCGCGAAGAGTCCACATG<br>TCCGCTACCCGATCCCATGAAACCACCTGGTCGGTCACATAGAATTTCAGG<br>CGACTTCGTTCGGGAGTGCACAGGTCGACACCGAGAAGGAACGGGGCAAT<br>CGCGGTATCTGTCGGGTGCCCGTCGTCTTGCAGGCCGAGGGTGCTGCGGAG<br>GTGATGCACGGGTTCCTTCAATCCTGGGAGAGCGAGCCTCTCGATGGAGTC<br>AAGAATGAGCTTTCCTGGCGAGAGTCCAGTCGCCAGCGACTTCATGCCGGG<br>GAAGAAGTAGGCTTTGGCGCTGATGCCACCATTCTGGAATTCCACGGCCAG<br>GGTTTGCTGGCCTCGTCCAGACTTGGCGAGGGGGGCTTTCTCTTTGAGCAA<br>CCATGTTTCCTCCTCGGGAGTCACCAAAAGGTGGTGTTGGAAGTGTCTGAA<br>CCGCGCTAGATCGATGGTGTTGTCAAGACACGCTAGGCGGGAGACGCATTC<br>CTCTGGCGCTGTCTTGTTGAAAGCATCGACGCCGGATTCTGCCAAGTGTGA<br>GAGTGGTTCGAAGCCAATCCGCACGATATTATGCAGAAGATTCCAGCTGAG<br>CTCGAACGGAACGCCATAGGTGGTCACATTGCTCCACCAAGGCAGAGGTG<br>CTTGTCCCACGACTGGGAAGGTGCCGAGGTATGGAACCAGCAGTTGCTGCA<br>GGAGATAAAGGCAGGGAACCTGATCCTTCTGCGGATATTTAGCATCACGCA<br>GAAGCTTGAGCAGATAGGGTCCAGTATATTCCCACCAACGTCGCTGGTCGT<br>CGCTGGGAAACCATGAGAACTTGTCGAGGAGCTGGAATGCGTCTTCTTCAG<br>GGCACGAAATCTCAGTTGGTATGGTCA
0796<br>(*ftmI*)	ATGGGTCGCTGGTGGGGGCTGCCACTTCCCCCAGAGATTATTCTGGAACTG<br>TTTGAGTATTTGAGACCCCCGGATGTCGTGGCTCTCGCGCAAGCCGCGGAA<br>GGGTTCGCCCAGCTGGCTCTCTCGGTGTACTCATCTAGTCTCCGCGAAAAA<br>GGACAGACGATATTATTTTCTGCAGTCACATGTGGACATGTAAGCATTGTC<br>CTGCATTATCTGGAGCAAGGAGCCGATCCCTGCGCCGCTGATGATGAGGGA<br>TACACGCCGTTGCACTGGGCCGCTGCTTACGGTCACTACAATGTGGTGTCT<br>CTGCTCATTGATGTGGGTGCTGATATCAACGCACGGCAGAACAGCGGATTT<br>AGTCCGCTAGATTATGCCATTATAACAGGACATGATCGCGTTGTGGAGGTT<br>CTTTTGAAGCATGGAGCTACCATTACGGATGTGACAATTGGCCCATCTCAG<br>AGAACGACTCTGCATGCAGCTGCTATCAAGGGATATAGCAAGATTGCAAA<br>GATGTTACTGAGCCACGGTGCTCCTACTGATGTCAAGGATGCCCATGGTCA<br>TACGCCTCTCCATCTGGCTGTTTCGAAAGGGCATCTTGAGATCGTTCAGGC<br>GCTGCTTTGTGCTGGGGCCACCGTCGACATTCAAGACAAAGTAGGCGACTC<br>ACCGCTGCACCTTGCTGCCGGCAACGGTTATTTTGCTATCGTACAGGAGCTT<br>CTGAATAAGGGAGCAGACCCTTCTCTGCAAGGTCGCAAAAATGCTACGCCA<br>TTGCACCAGGCATCTTTAATGGGTTTCGTTGACGTGGTCCAGCTTCTTCTAG<br>AATCTGGGGCAAATGTGTCTGCTCAACGGTCAGATGGACAAACCCCTCTTC<br>TCCAAGCCAGCGGCGCCGGCCAGGTTGCCACCGTGCGGCTGCTTTTAGGTG<br>CGGGGAGTAGTCCTTCAATTCCGGATGAAGATGGGAACACACCACTCCACT<br>TTGCTGTCCTCTCAGAGAAAGCTACAATCGCTGAAATGCTGATAGAGGCTG<br>GAGCGCATGTCGATAGCGCAAATGACAAGAATCAGACCCCCTTACACTGG<br>GCTGCAAAAGGCCATGAAGAGATGGTCCCTACTTTGCTGAAGCACAAAGCT<br>GATACCCATGCTCGTAGCCATACAGGGTGGACTCCGCTACATTGGGCAGCG<br>AACGAGGGCCATGTTGGTATAACGACTGCTTTATTGGACGCCGGGGCTCGC<br>GACCAGATTCAAAATGAACATGGGGAATCAGCGCTGCACTTGGCCGTTCAG<br>AAAGGACATCAAGCCGTCGTCCAATTGCTTATCCAACGGGGCAGCAAGCC<br>ACATCTCACCGACAACAAACTTCGCACAGTCCTGCATTGTGCAGCTGACGT<br>AGGACATGAGGATGTGGTAAGAATATTGCTGAGTGTCCAAGCCAGATCGG<br>ACGTCAAAGATATCAATGGTCGGACTCCGCTGTACTATGCAGCACTCCAAG<br>GCCATGTGGTGATCGCAAAACTGCTTCTTGAATTTGGTACCGCCCTTGACG<br>AGTCTGTCAAGGAGGCCTTTTTAGAGGCTGCAGAAGCTGGGCACGAATTGA<br>TGGTTCAACTTCTCATCACACATGGCATTGATCTATCTTTCAAAGATACAAG<br>CGGATCTACAGCGTTGCACAGGGCCGTTCTCGGGGGCCAAATTGAAGTGGT

	CGAACTTCTTCTCGATACAGAGGCTGATATCTCTGCCAGGGATAACAGCGG GAAAACAGCTTTGCATCTTGCTGCCCAGGAGGGCGAGGATGAGATTGCCA AAGTTCTCTTGAGAAACAGTGAAATCAGAGATCTCCAGGATTGTGATGGCT GGACGGCTCTGCATTGGGCGGTTAATAACGAACATGAAAACACCGTCCAGT CTCTATTGGATGCTGGTGTTGATCCTGGTATTGCCTCCTTCGACGCATGCAC GCCGCTTGATTTGGCAGAAGTTGGGGCTCTAGAAACAATTGAGCAGATGCT ACGAGAGGCATTAGCAGCAACCGATAGACCAACCATTGGTGACGCGCCTC CATG
0797	ATGGCGGCAGATTCGTTATCCCCGACTAAAATCCGTCCAGATATCATGACC AATACTGGAGAATCCGGACCCCTGTTACGTTTCGATACCTCTCAGAATGAG TGAGACTGTGATTTATGTGACGCCACCGTTGGGCTTATCTACTGGATGACT AGGGCGCTATTTTAGTCTCGGTGAGGATTCCCCGACGGTCCATGTTTCCAG GCTGGCGAAGCCTTGGGAGATGGAAGCTTCTAGTGCTTTTGAACAAGGATG CATGGTCATTTTGTTGGTCTTGAACGAGTCTTGTTGTGGGAGCCTTCTGAGG TATTTCCGCAGTAAGGTTAGCCAGCATCCTTTAGCACCTGCCTATTGACAGC TAGGAATTGCTATTGGTGCTACCATCTCATTGTACATGTTCAGCTACATTGT ACTATTGCAAAATGATCTGTCCAGCCTGCCTACTCCGTAGAATACCGTGCT CCCAACCTGCAGCGGATCAAGTCACACATCGGCCCCGGCCTGGATGGCCCG GGGTAGAGAATGGTCCCTAGACCTTGTGTCACCGGAAGATGAATTGGCTAG GGAACAGGCTCAGCCTCGATTTACATGGGATATGCGCGACCCACCGTGGGC ACGTTCGATGAATGGTCCAATTTTGTTGGCGATGATTTCTGGTCCTGGAACA GCGTGTATCCAGCATACAAGAACAGTTGCAGCTTGACCGGGCCAACAATG ACAAGATCGATCCATCCTTCAACATTTCCTACGATGTGTTGCCCTTTGAGAG CGGTGGAGGACCCCTACAATTATCGTATGGCAACTATCTCGGCGCCTACGC CCGTACCTGGCAGAAGAGCAGGAGGAATACATTAGCAGATTCGACATTGA TAATCGCAGTGTCCTTGTCGAAGGTATCCGTACACCCTTGCAGGAAAAGGA CTGCAGATGGGTTATCCTCGCCCACGACTTTTCTCCCTCGAGTCAAGAAGT GTGGAGGCGGCTTCCCGCTCACAGCGTCTTCGTCTTAAATGAAACATTGCT CGATTCTGCACCAGACCCGTTGCCTTCTCTCGTGGTGCGTCATTCATGCCAA CCAGCGAAAAGACACTCCATACCGCGACGGTGTCTGACGCCCTCCAACTGG CGCTAGATCTCGTCCAGAAATACCCTGGGCTGACAAAGAGCGTCGCATCTG TGCACGATATCAGCGCATTCGGTCAGTCAACAACCTTCTCCACGCCATCCC TGCATCTAAAGTCGGTGTTGTCACCTACCGCTACCATGACAGCCGTTTACGT GTTAGCAGTATCTTGCTGATTTCGGCTGAGGACTTTGAGCTCGATGGACCCT CCGTTGCTAGCTATGGCCTTGACAGTATAATTGGTGCGGAGATGAAGAACT GGTCATTTAAGGAAGTTGGACTTGAGTTCTCATTCCAGAACTTGCTGTCGCC CTCGTTGACGTTTGGAGGATTGGCCGAGGTGGTGTCTATGCATTTGGGTGT CATTCCGGCAACTGATTA

Table S25. Plasmid name and sequence.

Gene Name	Nucleotide sequence
pMD19-T	TCGCGCGTTTCGGTGATGACGGTGAAAACCTCTGACACATGCAGCTCCCGG<br>AGACGGTCACAGCTTGTCTGTAAGCGGATGCCGGGAGCAGACAAGCCCGT<br>CAGGGCGCGTCAGCGGGTGTTGGCGGGTGTCGGGGCTGGCTTAACTATGCG<br>GCATCAGAGCAGATTGTACTGAGAGTGCACCATATGCGGTGTGAAATACCG<br>CACAGATGCGTAAGGAGAAAATACCGCATCAGGCGCCATTCGCCATTCAG<br>GCTGCGCAACTGTTGGGAAGGGCGATCGGTGCGGGCCTCTTCGCTATTACG<br>CCAGCTGGCGAAAGGGGGATGTGCTGCAAGGCGATTAAGTTGGGTAACGC<br>CAGGGTTTTCCCAGTCACGACGTTGTAAAACGACGGCCAGTGGGCGTAATC<br>ATGGTCATAGCTGTTTCCTGTGTGAAATTGTTATCCGCTCACAATTCCACAC<br>AACATACGAGCCGGAAGCATAAAGTGTAAAGCCTGGGGTGCCTAATGAGT<br>GAGCTAACTCACATTAATTGCGTTGCGCTCACTGCCCGCTTTCCAGTCGGG<br>AAACCTGTCGTGCCAGCTGCATTAATGAATCGGCCAACGCGCGGGGAGAG<br>GCGGTTTGCGTATTGGGCGCTCTTCCGCTTCCTCGCTCACTGACTCGCTGCG<br>CTCGGTCGTTCGGCTGCGGCGAGCGGTATCAGCTCACTCAAAGGCGGTAAT<br>ACGGTTATCCACAGAATCAGGGGATAACGCAGGAAAGAACATGTGAGCAA<br>AAGGCCAGCAAAAGGCCAGGAACCGTAAAAAGGCCGCGTTGCTGGCGTTT<br>TTCCATAGGCTCCGCCCCCCTGACGAGCATCACAAAAATCGACGCTCAAGT<br>CAGAGGTGGCGAAACCCGACAGGACTATAAAGATACCAGGCGTTTCCCCC<br>TGGAAGCTCCCTCGTGCGCTCTCCTGTTCCGACCCTGCCGCTTACCGGATAC<br>CTGTCCGCCTTTCTCCCTTCGGGAAGCGTGGCGCTTTCTCATAGCTCACGCT<br>GTAGGTATCTCAGTTCGGTGTAGGTCGTTCGCTCCAAGCTGGGCTGTGTGC<br>ACGAACCCCCCGTTCAGCCCGACCGCTGCGCCTTATCCGGTAACTATCGTC<br>TTGAGTCCAACCCGGTAAGACACGACTTATCGCCACTGGCAGCAGCCACTG<br>GTAACAGGATTAGCAGAGCGAGGTATGTAGGCGGTGCTACAGAGTTCTTG<br>AAGTGGTGGCCTAACTACGGCTACACTAGAAGAACAGTATTTGGTATCTGC<br>GCTCTGCTGAAGCCAGTTACCTTCGGAAAAAGAGTTGGTAGCTCTTGATCC<br>GGCAAACAAACCACCGCTGGTAGCGGTGGTTTTTTTGTTTGCAAGCAGCAG<br>ATTACGCGCAGAAAAAAAGGATCTCAAGAAGATCCTTTGATCTTTTCTACG<br>GGGTCTGACGCTCAGTGGAACGAAAACTCACGTTAAGGGATTTTGGTCATG<br>AGATTATCAAAAAGGATCTTCACCTAGATCCTTTTAAATTAAAAATGAAGT<br>TTTAAATCAATCTAAAGTATATATGAGTAAACTTGGTCTGACAGTTACCAA<br>TGCTTAATCAGTGAGGCACCTATCTCAGCGATCTGTCTATTTCGTTCATCCA<br>TAGTTGCCTGACTCCCCGTCGTGTAGATAACTACGATACGGGAGGGCTTAC<br>CATCTGGCCCCAGTGCTGCAATGATACCGCGAGACCCACGCTCACCGGCTC<br>CAGATTTATCAGCAATAAACCAGCCAGCCGGAAGGGCCGAGCGCAGAAGT<br>GGTCCTGCAACTTTATCCGCCTCCATCCAGTCTATTAATTGTTGCCGGGAAG<br>CTAGAGTAAGTAGTTCGCCAGTTAATAGTTTGCGCAACGTTGTTGCCATTG<br>CTACAGGCATCGTGGTGTCACGCTCGTCGTTTGGTATGGCTTCATTCAGCTC<br>CGGTTCCCAACGATCAAGGCGAGTTACATGATCCCCCATGTTGTGCAAAAA<br>AGCGGTTAGCTCCTTCGGTCCTCCGATCGTTGTCAGAAGTAAGTTGGCCGC<br>AGTGTTATCACTCATGGTTATGGCAGCACTGCATAATTCTCTTACTGTCATG<br>CCATCCGTAAGATGCTTTTCTGTGACTGGTGAGTACTCAACCAAGTCATTCT<br>GAGAATAGTGTATGCGGCGACCGAGTTGCTCTTGCCCGGCGTCAATACGGG<br>ATAATACCGCGCCACATAGCAGAACTTTAAAAGTGCTCATCATTGGAAAAC<br>GTTCTTCGGGGCGAAAACTCTCAAGGATCTTACCGCTGTTGAGATCCAGTT<br>CGATGTAACCCACTCGTGCACCCAACTGATCTTCAGCATCTTTTACTTTCAC<br>CAGCGTTTCTGGGTGAGCAAAAACAGGAAGGCAAAATGCCGCAAAAAAGG<br>GAATAAGGGCGACACGGAAATGTTGAATACTCATACTCTTCCTTTTTCAAT<br>ATTATTGAAGCATTTATCAGGGTTATTGTCTCATGAGCGGATACATATTTGA<br>ATGTATTTAGAAAAATAAACAAATAGGGGTTCCGCGCACATTTCCCCGAAA<br>AGTGCCACCTGACGTCTAAGAAACCATTATTATCATGACATTAACCTATAA<br>AAATAGGCGTATCACGAGGCCCTTTCGTC

pAG1-H3	CAAATTGACGCTTAGACAACTTAATAACACATTGCGGACGTTTTTAATGTA<br>CTGGGGTGGTTTTTCTTTTCACCAGTGAGACGGGCAACAGCGGCGCCATTC<br>GCCATTCAGGCTGCGCAACTGTTGGGAAGGGCGATCGGTGCGGGCCTCTTC<br>GCTATTACGCCAGCTGGCGAAAGGGGGATGTGCTGCAAGGCGATTAAGTT<br>GGGTAACGCCAGGGTTTTCCCAGTCACGACGTTGTAAAACGACGGCCAGTG<br>AATTCGAGCTCGGTACCAAGGCCCGGGCTGGCCACGGCCGCCTAGGCGCG<br>CAAGGATCCTCTAGATCTCGAGGCCTGATCATCGATGGGCCCATCGATGAT<br>CAGGCCTCGACAGAAGATGATATTGAAGGAGCACTTTTTGGGCTTGGCTGG<br>AGCTAGTGGAGGTCAACAATGAATGCCTATTTTGGTTTAGTCGTCCAGGCG<br>GTGAGCACAAAATTTGTGTCGTTTGACAAGATGGTTCATTTAGGCAACTGG<br>TCAGATCAGCCCCACTTGTAGCAGTAGCGGCGGCGCTCGAAGTGTGACTCT<br>TATTAGCAGACAGGAACGAGGACATTATTATCATCTGCTGCTTGGTGCACG<br>ATAACTTGGTGCGTTTGTCAAGCAAGGTAAGTGAACGACCCGGTCATACCT<br>TCTTAAGTTCGCCCTTCCTCCCTTTATTTCAGATTCAATCTGACTTACCTATT<br>CTACCCAAGCATCGATATGAAAAAGCCTGAACTCACCGCGACGTCTGTCGA<br>GAAGTTTCTGATCGAAAAGTTCGACAGCGTCTCCGACCTGATGCAGCTCTC<br>GGAGGGCGAAGAATCTCGTGCTTTCAGCTTCGATGTAGGAGGGCGTGGATA<br>TGTCCTGCGGGTAAATAGCTGCGCCGATGGTTTCTACAAAGATCGTTATGT<br>TTATCGGCACTTTGCATCGGCCGCGCTCCCGATTCCGGAAGTGCTTGACATT<br>GGGGAATTCAGCGAGAGCCTGACCTATTGCATCTCCCGCCGTGCACAGGGT<br>GTCACGTTGCAAGACCTGCCTGAAACCGAACTGCCCGCTGTTCTGCAGCCG<br>GTCGCGGAGGCCATGGATGCGATCGCTGCGGCCGATCTTAGCCAGACGAG<br>CGGGTTCGGCCCATTCGGACCGCAAGGAATCGGTCAATACACTACATGGCG<br>TGATTTCATATGCGCGATTGCTGATCCCCATGTGTATCACTGGCAAACTGTG<br>ATGGACGACACCGTCAGTGCGTCCGTCGCGCAGGCTCTCGATGAGCTGATG<br>CTTTGGGCCGAGGACTGCCCCGAAGTCCGGCACCTCGTGCACGCGGATTTC<br>GGCTCCAACAATGTCCTGACGGACAATGGCCGCATAACAGCGGTCATTGAC<br>TGGAGCGAGGCGATGTTCGGGGATTCCCAATACGAGGTCGCCAACATCTTC<br>TTCTGGAGGCCGTGGTTGGCTTGTATGGAGCAGCAGACGCGCTACTTCGAG<br>CGGAGGCATCCGGAGCTTGCAGGATCGCCGCGGCTCCGGGCGTATATGCTC<br>CGCATTGGTCTTGACCAACTCTATCAGAGCTTGGTTGACGGCAATTTCGAT<br>GATGCAGCTTGGGCGCAGGGTCGATGCGACGCAATCGTCCGATCCGGAGC<br>CGGGACTGTCGGGCGTACACAAATCGCCCGCAGAAGCGCGGCCGTCTGGA<br>CCGATGGCTGTGTAGAAGTACTCGCCGATAGTGGAAACCGACGCCCCAGC<br>ACTCGTCCGAGGGCAAAGGAATAGAGTAGATGCCGACCGGGATCCACTTA<br>ACGTTACTGAAATCATCAAACAGCTTGACGAATCTGGATATAAGATCGTTG<br>GTGTCGATGTCAGCTCCGGAGTTGAGACAAATGGTGTTCAGGATCTCGATA<br>AGATACGTTCATTTGTCCAAGCAGCAAAGAGTGCCTTCTAGTGATTTAATA<br>GCTCCATGTCAACAAGAATAAAACGCGTTTCGGGTTTACCTCTTCCAGATA<br>CAGCTCATCTGCAATGCATTAATGCATTGGACCTCGCAACCCTAGTACGCC<br>CTTCAGGCTCCGGCGAAGCAGAAGAATAGCTTAGCAGAGTCTATTTTCATT<br>TTCGGGAGACGAGATCAAGCAGATCAACGGTCGTCAAGAGACCTACGAGA<br>CTGAGGAATCCGCTCTTGGCTCCACGCGACTATATATTTGTCTCTAATTGTA<br>CTTTGACATGCTCCTCTTCTTTACTCTGATAGCTTGACTATGAAAATTCCGT<br>CACCAGCCCCTGGGTTCGCAAAGATAATTGCACTGTTTCTTCCTTGAACTCT<br>CAAGCCTACAGGACACACATTCATCGTAGGTATAAACCTCGAAAATCATTC<br>CTACTAAGATGGGTATACAATAGTAACCATGGTTGCCTAGTGAATGCTCCG<br>TAACACCCAATACGCCGGCCGAAACTTTTTTACAACTCTCCTATGAGTCGTT<br>TACCCAGAATGCACAGGTACACTTGTTTAGAGGTAATCCTTCTTTCTAGAG<br>GATCCTCTACGCCGGACGCATCGTGGCCGGCATCACCGGCGCCACAGGTGC<br>GGTTGCTGGCGCCTATATCGCCGACATCACCGATGGGGAAGATCGGGCTCG<br>CCACTTCGGGCTCATGAGCGCTTGTTTCGGCGTGGGTATGGTGGCAGGCCC<br>CGTGGCCGGGGGACTGTTGGGCGCCATCTCCTTGCATGCACCATTCCTTGC<br>GGCGGCGGTGCTCAACGGCCTCAACCTACTACTGGGCTGCTTCCTAATGCA<br>GGAGTCGCATAAGGGAGAGCGTCGAGATCTAGAGGATCCCCCGACTAGTG<br>CGCGATCGCGGCCGGCCGGCGCGCCGTTTAAACGGATTTAAATTAATTAAT

	GTCGACCTGCAGGCATGCAAGCTTCGTGACTCCCTTAATTCTCCGCTCATGA<br>TCAGATTGTCGTTTCCCGCCTTCAGTTTAAACTATCAGTGTTTGACAGGATA<br>TATTGGCGGGTAAACCTAAGAGAAAAGAGCGTTTATTAGAATAATCGGAT<br>ATTTAAAAGGGCGTGAAAAGGTTTATCCGTTCGTCCATTTGTTTGTTCATGC<br>CAACCACAGGGTTCCAGATCCGACGAGCAAGGCAAGACCGAGCGCCTTTG<br>CGACGCTCACCGGGCTGGTTGCCCTCGCCGCTGGGCTGGCGGCCGTCTATG<br>GCCCTGCAAACGCGCCAGAAACGCCGTCGAAGCCGTGTGCGAGACACCGC<br>GGCCGCCGGCGTTGTGGATACCTCGCGGAAAACTTGGCCCTCACTGACAGA<br>TGAGGGGCGGACGTTGACACTTGAGGGGCCGACTCACCCGGCGCGGCGTT<br>GACAGATGAGGGGCAGGCTCGATTTCGGCCGGCGACGTGGAGCTGGCCAG<br>CCTCGCAAATCGGCGAAAACGCCTGATTTTACGCGAGTTTCCCACAGATGA<br>TGTGGACAAGCCTGGGGATAAGTGCCCTGCGGTATTGACACTTGAGGGGCG<br>CGACTACTGACAGATGAGGGGCGCGATCCTTGACACTTGAGGGGCAGAGT<br>GCTGACAGATGAGGGGCGCACCTATTGACATTTGAGGGGCTGTCCACAGGC<br>AGAAAATCCAGCATTTGCAAGGGTTTCCGCCCGTTTTTCGGCCACCGCTAA<br>CCTGTCTTTTAACCTGCTTTTAAACCAATATTTATAAACCTTGTTTTTAACCA<br>GGGCTGCGCCCTGTGCGCGTGACCGCGCACGCCGAAGGGGGGTGCCCCCC<br>CTTCTCGAACCCTCCCGGCCCGCTAACGCGGGCCTCCCATCCCCCCAGGCG<br>TACGCCACTGGAGCACCTCAAAAACACCATCATACACTAAATCAGTAAGTT<br>GGCAGCATCACCCATAATTGTGGTTTCAAAATCGGCTCCGTCGATACTATG<br>TTATACGCCAACTTTGAAAACAACTTTGAAAAAGCTGTTTTCTGGTATTTAA<br>GGTTTTAGAATGCAAGGAACAGTGAATTGGAGTTCGTCTTGTTATAATTAG<br>CTTCTTGGGGTATCTTTAAATACTGTAGAAAAGAGGAAGGAAATAATAAAT<br>GGCTAAAATGAGAATATCACCGGAATTGAAAAAACTGATCGAAAAATACC<br>GCTGCGTAAAAGATACGGAAGGAATGTCTCCTGCTAAGGTATATAAGCTGG<br>TGGGAGAAAATGAAAACCTATATTTAAAAATGACGGACAGCCGGTATAAA<br>GGGACCACCTATGATGTGGAACGGGAAAAGGACATGATGCTATGGCTGGA<br>AGGAAAGCTGCCTGTTCCAAAGGTCCTGCACTTTGAACGGCATGATGGCTG<br>GAGCAATCTGCTCATGAGTGAGGCCGATGGCGTCCTTTGCTCGGAAGAGTA<br>TGAAGATGAACAAAGCCCTGAAAAGATTATCGAGCTGTATGCGGAGTGCA<br>TCAGGCTCTTTCACTCCATCGACATATCGGATTGTCCCTATACGAATAGCTT<br>AGACAGCCGCTTAGCCGAATTGGATTACTTACTGAATAACGATCTGGCCGA<br>TGTGGATTGCGAAAACTGGGAAGAAGACACTCCATTTAAAGATCCGCGCG<br>AGCTGTATGATTTTTTAAAGACGGAAAAGCCCGAAGAGGAACTTGTCTTTT<br>CCCACGGCGACCTGGGAGACAGCAACATCTTTGTGAAAGATGGCAAAGTA<br>AGTGGCTTTATTGATCTTGGGAGAAGCGGCAGGGCGGACAAGTGGTATGA<br>CATTGCCTTCTGCGTCCGGTCGATCAGGGAGGATATCGGGGAAGAACAGTA<br>TGTCGAGCTATTTTTTGACTTACTGGGGATCAAGCCTGATTGGGAGAAAAT<br>AAAATATTATATTTTACTGGATGAATTGTTTTAGTACCTAGATGTGGCGCAA<br>CGATGCCGGCGACAAGCAGGAGCGCACCGACTTCTTCCGCATCAAGTGTTT<br>TGGCTCTCAGGCCGAGGCCCACGGCAAGTATTTGGGCAAGGGGTCGCTGGT<br>ATTCGTGCAGGGCAAGATTCGGAATACCAAGTACGAGAAGGACGGCCAGA<br>CGGTCTACGGGACCGACTTCATTGCCGATAAGGTGGATTATCTGGACACCA<br>AGGCACCAGGCGGGTCAAATCAGGAATAAGGGCACATTGCCCCGGCGTGA<br>GTCGGGGCAATCCCGCAAGGAGGGTGAATGAATCGGACGTTTGACCGGAA<br>GGCATACAGGCAAGAACTGATCGACGCGGGGTTTTCCGCCGAGGATGCCG<br>AAACCATCGCAAGCCGCACCGTCATGCGTGCGCCCCGCGAAACCTTCCAGT<br>CCGTCGGCTCGATGGTCCAGCAAGCTACGGCCAAGATCGAGCGCGACAGC<br>GTGCAACTGGCTCCCCCTGCCCTGCCCGCGCCATCGGCCGCCGTGGAGCGT<br>TCGCGTCGTCTCGAACAGGAGGCGGCAGGTTTGGCGAAGTCGATGACCATC<br>GACACGCGAGGAACTATGACGACCAAGAAGCGAAAAACCGCCGGCGAGG<br>ACCTGGCAAAACAGGTCAGCGAGGCCAAGCAGGCCGCGTTGCTGAAACAC<br>ACGAAGCAGCAGATCAAGGAAATGCAGCTTTCCTTGTTCGATATTGCGCCG<br>TGGCCGGACACGATGCGAGCGATGCCAAACGACACGGCCCGCTCTGCCCT<br>GTTCACCACGCGCAACAAGAAAATCCCGCGCGAGGCGCTGCAAAACAAGG<br>TCATTTTCCACGTCAACAAGGACGTGAAGATCACCTACACCGGCGTCGAGC

	TGCGGGCCGACGATGACGAACTGGTGTGGCAGCAGGTGTTGGAGTACGCG<br>AAGCGCACCCCTATCGGCGAGCCGATCACCTTCACGTTCTACGAGCTTTGC<br>CAGGACCTGGGCTGGTCGATCAATGGCCGGTATTACACGAAGGCCGAGGA<br>ATGCCTGTCGCGCCTACAGGCGACGGCGATGGGCTTCACGTCCGACCGCGT<br>TGGGCACCTGGAATCGGTGTCGCTGCTGCACCGCTTCCGCGTCCTGGACCG<br>TGGCAAGAAAACGTCCCGTTGCCAGGTCCTGATCGACGAGGAAATCGTCGT<br>GCTGTTTGCTGGCGACCACTACACGAAATTCATATGGGAGAAGTACCGCAA<br>GCTGTCGCCGACGGCCCGACGGATGTTCGACTATTTCAGCTCGCACCGGGA<br>GCCGTACCCGCTCAAGCTGGAAACCTTCCGCCTCATGTGCGGATCGGATTC<br>CACCCGCGTGAAGAAGTGGCGCGAGCAGGTCGGCGAAGCCTGCGAAGAGT<br>TGCGAGGCAGCGGCCTGGTGGAACACGCCTGGGTCAATGATGACCTGGTG<br>CATTGCAAACGCTAGGGCCTTGTGGGGTCAGTTCCGGCTGGATCTGCTCTC<br>CCGCTGACGCCGTCCCGGACTGATGGGCTGCCTGTATCGAGTGGTGATTTT<br>GTGCCGAGCTGCCGGTCGGGGAGCTGTTGGCTGGCTGGTGGCAGGATATAT<br>TGTGGTGTAAA
pPIC9K	AGATCTAACATCCAAAGACGAAAGGTTGAATGAAACCTTTTTGCCATCCGA<br>CATCCACAGGTCCATTCTCACACATAAGTGCCAAACGCAACAGGAGGGGA<br>TACACTAGCAGCAGACCGTTGCAAACGCAGGACCTCCACTCCTCTTCTCCT<br>CAACACCCACTTTTGCCATCGAAAAACCAGCCCAGTTATTGGGCTTGATTG<br>GAGCTCGCTCATTCCAATTCCTTCTATTAGGCTACTAACACCATGACTTTAT<br>TAGCCTGTCTATCCTGGCCCCCCTGGCGAGGTTCATGTTTGTTTATTTCCGA<br>ATGCAACAAGCTCCGCATTACACCCGAACATCACTCCAGATGAGGGCTTTC<br>TGAGTGTGGGGTCAAATAGTTTCATGTTCCCCAAATGGCCCAAAACTGACA<br>GTTTAAACGCTGTCTTGGAACCTAATATGACAAAAGCGTGATCTCATCCAA<br>GATGAACTAAGTTTGGTTCGTTGAAATGCTAACGGCCAGTTGGTCAAAAAG<br>AAACTTCCAAAAGTCGCCATACCGTTTGTCTTGTTTGGTATTGATTGACGAA<br>TGCTCAAAAATAATCTCATTAATGCTTAGCGCAGTCTCTCTATCGCTTCTGA<br>ACCCCGGTGCACCTGTGCCGAAACGCAAATGGGGAAACACCCGCTTTTTGG<br>ATGATTATGCATTGTCTCCACATTGTATGCTTCCAAGATTCTGGTGGGAATA<br>CTGCTGATAGCCTAACGTTCATGATCAAAATTTAACTGTTCTAACCCCTACT<br>TGACAGCAATATATAAACAGAAGGAAGCTGCCCTGTCTTAAACCTTTTTTT<br>TTATCATCATTATTAGCTTACTTTCATAATTGCGACTGGTTCCAATTGACAA<br>GCTTTTGATTTTAACGACTTTTAACGACAACTTGAGAAGATCAAAAAACAA<br>CTAATTATTCGAAGGATCCAAACGATGAGATTTCCTTCAATTTTTACTGCAG<br>TTTTATTCGCAGCATCCTCCGCATTAGCTGCTCCAGTCAACACTACAACAGA<br>AGATGAAACGGCACAAATTCCGGCTGAAGCTGTCATCGGTTACTCAGATTT<br>AGAAGGGGATTTCGATGTTGCTGTTTTGCCATTTTCCAACAGCACAAATAA<br>CGGGTTATTGTTTATAAATACTACTATTGCCAGCATTGCTGCTAAAGAAGA<br>AGGGGTATCTCTCGAGAAAAGAGAGGCTGAAGCTTACGTAGAATTCCCTA<br>GGGCGGCCGCGAATTAATTCGCCTTAGACATGACTGTTCCTCAGTTCAAGT<br>TGGGCACTTACGAGAAGACCGGTCTTGCTAGATTCTAATCAAGAGGATGTC<br>AGAATGCCATTTGCCTGAGAGATGCAGGCTTCATTTTTGATACTTTTTTATT<br>TGTAACCTATATAGTATAGGATTTTTTTTGTCATTTTGTTTCTTCTCGTACGA<br>GCTTGCTCCTGATCAGCCTATCTCGCAGCTGATGAATATCTTGTGGTAGGG<br>GTTTGGGAAAATCATTCGAGTTTGATGTTTTTCTTGGTATTTCCCACTCCTCT<br>TCAGAGTACAGAAGATTAAGTGAGAAGTTCGTTTGTGCAAGCTTATCGATA<br>AGCTTTAATGCGGTAGTTTATCACAGTTAAATTGCTAACGCAGTCAGGCAC<br>CGTGTATGAAATCTAACAATGCGCTCATCGTCATCCTCGGCACCGTCACCC<br>TGGATGCTGTAGGCATAGGCTTGGTTATGCCGGTACTGCCGGGCCTCTTGC<br>GGGATATCGTCCATTCCGACAGCATCGCCAGTCACTATGGCGTGCTGCTAG<br>CGCTATATGCGTTGATGCAATTTCTATGCGCACCCGTTCTCGGAGCACTGTC<br>CGACCGCTTTGGCCGCCGCCCAGTCCTGCTCGCTTCGCTACTTGGAGCCACT<br>ATCGACTACGCGATCATGGCGACCACACCCGTCCTGTGGATCTATCGAATC<br>TAAATGTAAGTTAAAATCTCTAAATAATTAAATAAGTCCCAGTTTCTCCAT<br>ACGAACCTTAACAGCATTGCGGTGAGCATCTAGACCTTCAACAGCAGCCAG<br>ATCCATCACTGCTTGGCCAATATGTTTCAGTCCCTCAGGAGTTACGTCTTGT

	GAAGTGATGAACTTCTGGAAGGTTGCAGTGTTAACTCCGCTGTATTGACGG<br>GCATATCCGTACGTTGGCAAAGTGTGGTTGGTACCGGAGGAGTAATCTCCA<br>CAACTCTCTGGAGAGTAGGCACCAACAAACACAGATCCAGCGTGTTGTACT<br>TGATCAACATAAGAAGAAGCATTCTCGATTTGCAGGATCAAGTGTTCAGGA<br>GCGTACTGATTGGACATTTCCAAAGCCTGCTCGTAGGTTGCAACCGATAGG<br>GTTGTAGAGTGTGCAATACACTTGCGTACAATTTCAACCCTTGGCAACTGC<br>ACAGCTTGGTTGTGAACAGCATCTTCAATTCTGGCAAGCTCCTTGTCTGTCA<br>TATCGACAGCCAACAGAATCACCTGGGAATCAATACCATGTTCAGCTTGAG<br>ACAGAAGGTCTGAGGCAACGAAATCTGGATCAGCGTATTTATCAGCAATA<br>ACTAGAACTTCAGAAGGCCCAGCAGGCATGTCAATACTACACAGGGCTGA<br>TGTGTCATTTTGAACCATCATCTTGGCAGCAGTAACGAACTGGTTTCCTGGA<br>CCAAATATTTTGTCACACTTAGGAACAGTTTCTGTTCCGTAAGCCATAGCA<br>GCTACTGCCTGGGCGCCTCCTGCTAGCACGATACACTTAGCACCAACCTTG<br>TGGGCAACGTAGATGACTTCTGGGGTAAGGGTACCATCCTTCTTAGGTGGA<br>GATGCAAAAACAATTTCTTTGCAACCAGCAACTTTGGCAGGAACACCCAGC<br>ATCAGGGAAGTGGAAGGCAGAATTGCGGTTCCACCAGGAATATAGAGGCC<br>AACTTTCTCAATAGGTCTTGCAAAACGAGAGCAGACTACACCAGGGCAAGT<br>CTCAACTTGCAACGTCTCCGTTAGTTGAGCTTCATGGAATTTCCTGACGTTA<br>TCTATAGAGAGATCAATGGCTCTCTTAACGTTATCTGGCAATTGCATAAGTT<br>CCTCTGGGAAAGGAGCTTCTAACACAGGTGTCTTCAAAGCGACTCCATCAA<br>ACTTGGCAGTTAGTTCTAAAAGGGCTTTGTCACCATTTTGACGAACATTGTC<br>GACAATTGGTTTGACTAATTCCATAATCTGTTCCGTTTTCTGGATAGGACGA<br>CGAAGGGCATCTTCAATTTCTTGTGAGGAGGCCTTAGAAACGTCAATTTTG<br>CACAATTCAATACGACCTTCAGAAGGGACTTCTTTAGGTTTGGATTCTTCTT<br>TAGGTTGTTCCTTGGTGTATCCTGGCTTGGCATCTCCTTTCCTTCTAGTGACC<br>TTTAGGGACTTCATATCCAGGTTTCTCTCCACCTCGTCCAACGTCACACCGT<br>ACTTGGCACATCTAACTAATGCAAAATAAAATAAGTCAGCACATTCCCAGG<br>CTATATCTTCCTTGGATTTAGCTTCTGCAAGTTCATCAGCTTCCTCCCTAATT<br>TTAGCGTTCAACAAAACTTCGTCGTCAAATAACCGTTTGGTATAAGAACCT<br>TCTGGAGCATTGCTCTTACGATCCCACAAGGTGGCTTCCATGGCTCTAAGA<br>CCCTTTGATTGGCCAAAACAGGAAGTGCGTTCCAAGTGACAGAAACCAAC<br>ACCTGTTTGTTCAACCACAAATTTCAAGCAGTCTCCATCACAATCCAATTCG<br>ATACCCAGCAACTTTTGAGTTGCTCCAGATGTAGCACCTTTATACCACAAA<br>CCGTGACGACGAGATTGGTAGACTCCAGTTTGTGTCCTTATAGCCTCCGGA<br>ATAGACTTTTTGGACGAGTACACCAGGCCCAACGAGTAATTAGAAGAGTCA<br>GCCACCAAAGTAGTGAATAGACCATCGGGGCGGTCAGTAGTCAAAGACGC<br>CAACAAAATTTCACTGACAGGGAACTTTTTGACATCTTCAGAAAGTTCGTA<br>TTCAGTAGTCAATTGCCGAGCATCAATAATGGGGATTATACCAGAAGCAAC<br>AGTGGAAGTCACATCTACCAACTTTGCGGTCTCAGAAAAAGCATAAACAGT<br>TCTACTACCGCCATTAGTGAAACTTTTCAAATCGCCCAGTGGAGAAGAAAA<br>AGGCACAGCGATACTAGCATTAGCGGGCAAGGATGCAACTTTATCAACCA<br>GGGTCCTATAGATAACCCTAGCGCCTGGGATCATCCTTTGGACAACTCTTTC<br>TGCCAAATCTAGGTCCAAAATCACTTCATTGATACCATTATTGTACAACTTG<br>AGCAAGTTGTCGATCAGCTCCTCAAATTGGTCCTCTGTAACGGATGACTCA<br>ACTTGCACATTAACTTGAAGCTCAGTCGATTGAGTGAACTTGATCAGGTTG<br>TGCAGCTGGTCAGCAGCATAGGGAAACACGGCTTTTCCTACCAAACTCAAG<br>GAATTATCAAACTCTGCAACACTTGCGTATGCAGGTAGCAAGGGAAATGTC<br>ATACTTGAAGTCGGACAGTGAGTGTAGTCTTGAGAAATTCTGAAGCCGTAT<br>TTTTATTATCAGTGAGTCAGTCATCAGGAGATCCTCTACGCCGGACGCATC<br>GTGGCCGACCTGCAGGGGGGGGGGGGGCGCTGAGGTCTGCCTCGTGAAGA<br>AGGTGTTGCTGACTCATACCAGGCCTGAATCGCCCCATCATCCAGCCAGAA<br>AGTGAGGGAGCCACGGTTGATGAGAGCTTTGTTGTAGGTGGACCAGTTGGT<br>GATTTTGAACTTTTGCTTTGCCACGGAACGGTCTGCGTTGTCGGGAAGATG<br>CGTGATCTGATCCTTCAACTCAGCAAAAGTTCGATTTATTCAACAAAGCCG<br>CCGTCCCGTCAAGTCAGCGTAATGCTCTGCCAGTGTTACAACCAATTAACC<br>AATTCTGATTAGAAAAACTCATCGAGCATCAAATGAAACTGCAATTTATTC

	ATATCAGGATTATCAATACCATATTTTTGAAAAAGCCGTTTCTGTAATGAA<br>GGAGAAAACTCACCGAGGCAGTTCCATAGGATGGCAAGATCCTGGTATCG<br>GTCTGCGATTCCGACTCGTCCAACATCAATACAACCTATTAATTTCCCCTCG<br>TCAAAAATAAGGTTATCAAGTGAGAAATCACCATGAGTGACGACTGAATC<br>CGGTGAGAATGGCAAAAGCTTATGCATTTCTTTCCAGACTTGTTCAACAGG<br>CCAGCCATTACGCTCGTCATCAAAATCACTCGCATCAACCAAACCGTTATT<br>CATTCGTGATTGCGCCTGAGCGAGACGAAATACGCGATCGCTGTTAAAAGG<br>ACAATTACAAACAGGAATCGAATGCAACCGGCGCAGGAACACTGCCAGCG<br>CATCAACAATATTTTCACCTGAATCAGGATATTCTTCTAATACCTGGAATGC<br>TGTTTTCCCGGGGATCGCAGTGGTGAGTAACCATGCATCATCAGGAGTACG<br>GATAAAATGCTTGATGGTCGGAAGAGGCATAAATTCCGTCAGCCAGTTTAG<br>TCTGACCATCTCATCTGTAACATCATTGGCAACGCTACCTTTGCCATGTTTC<br>AGAAACAACTCTGGCGCATCGGGCTTCCCATACAATCGATAGATTGTCGCA<br>CCTGATTGCCCGACATTATCGCGAGCCCATTTATACCCATATAAATCAGCA<br>TCCATGTTGGAATTTAATCGCGGCCTCGAGCAAGACGTTTCCCGTTGAATA<br>TGGCTCATAACACCCCTTGTATTACTGTTTATGTAAGCAGACAGTTTTATTG<br>TTCATGATGATATATTTTTATCTTGTGCAATGTAACATCAGAGATTTTGAGA<br>CACAACGTGGCTTTCCCCCCCCCCCCCTGCAGGTCGGCATCACCGGCGCCAC<br>AGGTGCGGTTGCTGGCGCCTATATCGCCGACATCACCGATGGGGAAGATCG<br>GGCTCGCCACTTCGGGCTCATGAGCGCTTGTTTCGGCGTGGGTATGGTGGC<br>AGGCCCCGTGGCCGGGGGACTGTTGGGCGCCATCTCCTTGCATGCACCATT<br>CCTTGCGGCGGCGGTGCTCAACGGCCTCAACCTACTACTGGGCTGCTTCCT<br>AATGCAGGAGTCGCATAAGGGAGAGCGTCGAGTATCTATGATTGGAAGTA<br>TGGGAATGGTGATACCCGCATTCTTCAGTGTCTTGAGGTCTCCTATCAGATT<br>ATGCCCAACTAAAGCAACCGGAGGAGGAGATTTCATGGTAAATTTCTCTGA<br>CTTTTGGTCATCAGTAGACTCGAACTGTGAGACTATCTCGGTTATGACAGC<br>AGAAATGTCCTTCTTGGAGACAGTAAATGAAGTCCCACCAATAAAGAAATC<br>CTTGTTATCAGGAACAAACTTCTTGTTTCGAACTTTTTCGGTGCCTTGAACT<br>ATAAAATGTAGAGTGGATATGTCGGGTAGGAATGGAGCGGGCAAATGCTT<br>ACCTTCTGGACCTTCAAGAGGTATGTAGGGTTTGTAGATACTGATGCCAAC<br>TTCAGTGACAACGTTGCTATTTCGTTCAAACCATTCCGAATCCAGAGAAAT<br>CAAAGTTGTTTGTCTACTATTGATCCAAGCCAGTGCGGTCTTGAAACTGAC<br>AATAGTGTGCTCGTGTTTTGAGGTCATCTTTGTATGAATAAATCTAGTCTTT<br>GATCTAAATAATCTTGACGAGCCAAGGCGATAAATACCCAAATCTAAAACT<br>CTTTTAAAACGTTAAAAGGACAAGTATGTCTGCCTGTATTAAACCCCAAAT<br>CAGCTCGTAGTCTGATCCTCATCAACTTGAGGGGCACTATCTTGTTTTAGAG<br>AAATTTGCGGAGATGCGATATCGAGAAAAAGGTACGCTGATTTTAAACGTG<br>AAATTTATCTCAAGATCTCTGCCTCGCGCGTTTCGGTGATGACGGTGAAAA<br>CCTCTGACACATGCAGCTCCCGGAGACGGTCACAGCTTGTCTGTAAGCGGA<br>TGCCGGGAGCAGACAAGCCCGTCAGGGCGCGTCAGCGGGTGTTGGCGGGT<br>GTCGGGGCGCAGCCATGACCCAGTCACGTAGCGATAGCGGAGTGTATACT<br>GGCTTAACTATGCGGCATCAGAGCAGATTGTACTGAGAGTGCACCATATGC<br>GGTGTGAAATACCGCACAGATGCGTAAGGAGAAAATACCGCATCAGGCGC<br>TCTTCCGCTTCCTCGCTCACTGACTCGCTGCGCTCGGTCGTTCGGCTGCGGC<br>GAGCGGTATCAGCTCACTCAAAGGCGGTAATACGGTTATCCACAGAATCAG<br>GGGATAACGCAGGAAAGAACATGTGAGCAAAAGGCCAGCAAAAGGCCAG<br>GAACCGTAAAAAGGCCGCGTTGCTGGCGTTTTTCCATAGGCTCCGCCCCCC<br>TGACGAGCATCACAAAAATCGACGCTCAAGTCAGAGGTGGCGAAACCCGA<br>CAGGACTATAAAGATACCAGGCGTTTCCCCCTGGAAGCTCCCTCGTGCGCT<br>CTCCTGTTCCGACCCTGCCGCTTACCGGATACCTGTCCGCCTTTCTCCCTTC<br>GGGAAGCGTGGCGCTTTCTCAATGCTCACGCTGTAGGTATCTCAGTTCGGT<br>GTAGGTCGTTCGCTCCAAGCTGGGCTGTGTGCACGAACCCCCCGTTCAGCC<br>CGACCGCTGCGCCTTATCCGGTAACTATCGTCTTGAGTCCAACCCGGTAAG<br>ACACGACTTATCGCCACTGGCAGCAGCCACTGGTAACAGGATTAGCAGAG<br>CGAGGTATGTAGGCGGTGCTACAGAGTTCTTGAAGTGGTGGCCTAACTACG<br>GCTACACTAGAAGGACAGTATTTGGTATCTGCGCTCTGCTGAAGCCAGTTA

	CCTTCGGAAAAAGAGTTGGTAGCTCTTGATCCGGCAAACAAACCACCGCTG<br>GTAGCGGTGGTTTTTTTGTTTGCAAGCAGCAGATTACGCGCAGAAAAAAAG<br>GATCTCAAGAAGATCCTTTGATCTTTTCTACGGGGTCTGACGCTCAGTGGA<br>ACGAAAACTCACGTTAAGGGATTTTGGTCATGAGATTATCAAAAAGGATCT<br>TCACCTAGATCCTTTTAAATTAAAAATGAAGTTTTAAATCAATCTAAAGTAT<br>ATATGAGTAAACTTGGTCTGACAGTTACCAATGCTTAATCAGTGAGGCACC<br>TATCTCAGCGATCTGTCTATTTCGTTCATCCATAGTTGCCTGACTCCCCGTC<br>GTGTAGATAACTACGATACGGGAGGGCTTACCATCTGGCCCCAGTGCTGCA<br>ATGATACCGCGAGACCCACGCTCACCGGCTCCAGATTTATCAGCAATAAAC<br>CAGCCAGCCGGAAGGGCCGAGCGCAGAAGTGGTCCTGCAACTTTATCCGC<br>CTCCATCCAGTCTATTAATTGTTGCCGGGAAGCTAGAGTAAGTAGTTCGCC<br>AGTTAATAGTTTGCGCAACGTTGTTGCCATTGCTGCAGGCATCGTGGTGTC<br>ACGCTCGTCGTTTGGTATGGCTTCATTCAGCTCCGGTTCCCAACGATCAAGG<br>CGAGTTACATGATCCCCCATGTTGTGCAAAAAAGCGGTTAGCTCCTTCGGT<br>CCTCCGATCGTTGTCAGAAGTAAGTTGGCCGCAGTGTTATCACTCATGGTT<br>ATGGCAGCACTGCATAATTCTCTTACTGTCATGCCATCCGTAAGATGCTTTT<br>CTGTGACTGGTGAGTACTCAACCAAGTCATTCTGAGAATAGTGTATGCGGC<br>GACCGAGTTGCTCTTGCCCGGCGTCAACACGGGATAATACCGCGCCACATA<br>GCAGAACTTTAAAAGTGCTCATCATTGGAAAACGTTCTTCGGGGCGAAAAC<br>TCTCAAGGATCTTACCGCTGTTGAGATCCAGTTCGATGTAACCCACTCGTGC<br>ACCCAACTGATCTTCAGCATCTTTTACTTTCACCAGCGTTTCTGGGTGAGCA<br>AAAACAGGAAGGCAAAATGCCGCAAAAAAGGGAATAAGGGCGACACGGA<br>AATGTTGAATACTCATACTCTTCCTTTTTCAATATTATTGAAGCATTTATCA<br>GGGTTATTGTCTCATGAGCGGATACATATTTGAATGTATTTAGAAAAATAA<br>ACAAATAGGGGTTCCGCGCACATTTCCCCGAAAAGTGCCACCTGACGTCTA<br>AGAAACCATTATTATCATGACATTAACCTATAAAAATAGGCGTATCACGAG<br>GCCCTTTCGTCTTCAAGAATTAATTCTCATGTTTGACAGCTTATCATCGATA<br>AGCTGACTCATGTTGGTATTGTGAAATAGACGCAGATCGGGAACACTGAAA<br>AATAACAGTTATTATTCG

Table S26. DP4+ analysis of compound **4** (major).

B3LYP		PCM		6-31G(d)		Shielding Tensors	
		DP4+	98.54%	1.46%	-	-	-
Nuclei	sp2?	Experimental	Isomer 1	Isomer 2	Isomer 3	Isomer 4	Isomer 5
C		20.49	20.3	20.7			
C		25.23	27.1	24.1			
C		26.96	27.8	27.1			
C		28.72	28.5	35.0			
C		49.62	52.3	50.3			
C		55.5	53.3	53.3			
C		59.81	64.1	65.1			
C	x	94.74	88.8	88.8			
C	x	110.74	106.0	105.8			
C	x	113.58	110.2	110.3			
C	x	114.79	112.7	111.6			
C	x	117.76	116.18	116.15			
C	x	123.23	118.29	118.35			
C	x	129.06	123.83	123.79			
C	x	134.2	129.21	129.13			
C	x	137.84	133.37	133.27			
C	x	143.2	134.98	134.97			
C	x	143.55	135.90	135.98			
C	x	156.15	139.78	139.98			
C	x	161.22	153.48	152.28			
C	x	165.46	160.22	158.44			
C	x	173.38	163.81	167.25			

Functional	Solvent?		Basis Set		Type of Data	
B3LYP	PCM		6-31G(d)		Shielding Tensors	
	Isomer 1	Isomer 2	Isomer 3	Isomer 4	Isomer 5	Isomer 6
sDP4+ (H data)	-	-	-	-	-	-
sDP4+ (C data)	98.89%	1.11%	-	-	-	-
sDP4+ (all data)	98.89%	1.11%	-	-	-	-
uDP4+ (H data)	-	-	-	-	-	-
uDP4+ (C data)	43.17%	56.83%	-	-	-	-
uDP4+ (all data)	43.17%	56.83%	-	-	-	-
DP4+ (H data)	-	-	-	-	-	-
DP4+ (C data)	98.54%	1.46%	-	-	-	-
DP4+ (all data)	98.54%	1.46%	-	-	-	-

Experimental represented the experimental data of major. **Isomer 1** represented the calculated **4a**; **Isomer 2** represented the calculated **4b**.

Table S27. DP4+ analysis of compound **4** (minor).

B3LYP		PCM		6-31G(d)		Shielding Tensors	
		DP4+	0.16%	99.84%	-	-	-
Nuclei	sp2?	Experimenta	Isomer 1	Isomer 2	Isomer 3	Isomer 4	Isomer 5
C		20.32	20.3	20.7			
C		22	27.1	24.1			
C		26.83	27.8	27.1			
C		31.26	28.5	35.0			
C		47.57	52.3	50.3			
C		55.45	53.3	53.3			
C		60.49	64.1	65.1			
C	x	94.74	88.8	88.8			
C	x	110.32	106.0	105.8			
C	x	113.28	110.2	110.3			
C	x	114.79	112.7	111.6			
C	x	117.81	116.18	116.15			
C	x	123.54	118.29	118.35			
C	x	129.37	123.83	123.79			
C	x	134.1	129.21	129.13			
C	x	137.81	133.37	133.27			
C	x	143.2	134.98	134.97			
C	x	143.2	135.90	135.98			
C	x	154.74	139.78	139.98			
C	x	160.95	153.48	152.28			
C	x	166.44	160.22	158.44			
C	x	173.71	163.81	167.25			

Functional	Solvent?		Basis Set		Type of Data	
B3LYP	PCM		6-31G(d)		Shielding Tensors	
	Isomer 1	Isomer 2	Isomer 3	Isomer 4	Isomer 5	Isomer 6
sDP4+ (H data)	-	-	-	-	-	-
sDP4+ (C data)	0.22%	99.78%	-	-	-	-
sDP4+ (all data)	0.22%	99.78%	-	-	-	-
uDP4+ (H data)	-	-	-	-	-	-
uDP4+ (C data)	42.83%	57.17%	-	-	-	-
uDP4+ (all data)	42.83%	57.17%	-	-	-	-
DP4+ (H data)	-	-	-	-	-	-
DP4+ (C data)	0.16%	99.84%	-	-	-	-
DP4+ (all data)	0.16%	99.84%	-	-	-	-

Experimental represented the experimental data of minor. **Isomer 1** represented the calculated **4a**; **Isomer 2** represented the calculated **4b**.

Table S28. DP4+ analysis of compound **5** (major).

Functional		Solvent?		Basis Set		Type of Data	
B3LYP		PCM		6-31G(d)		Shielding Tensors	
		DP4+	97.77%	2.23%	-	-	-
Nuclei	sp2?	Experimenta	Isomer 1	Isomer 2	Isomer 3	Isomer 4	Isomer 5
C	x	128.1	121.5	121.6			
C	x	134.6	127.0	125.8			
C	x	114.9	108.9	107.8			
C	x	142.3	133.3	133.9			
C	x	122.7	114.9	115.7			
C	x	109.6	99.1	98.5			
C	x	160.3	152.0	152.0			
C	x	94.8	89.4	88.1			
C	x	138.5	130.0	130.9			
C	x	141.9	135.7	135.2			
C	x	113.3	107.7	107.4			
C	x	166.7	157.86	156.31			
C	x	119.5	112.02	113.98			
C		55.3	49.12	48.94			
C		59.6	58.57	59.24			
C		28.3	26.15	29.39			
C		25.2	22.39	18.13			
C		49.5	46.49	45.04			
C	x	141.1	140.40	138.35			
C		20.2	22.62	22.21			
C		27.1	15.08	15.39			
C	x	172.6	168.22	168.07			
C		51.6	47.03	47.03			

Functional	Solvent?		Basis Set		Type of Data	
B3LYP	PCM		6-31G(d)		Shielding Tensors	
	Isomer 1	Isomer 2	Isomer 3	Isomer 4	Isomer 5	Isomer 6
sDP4+ (H data)	-	-	-	-	-	-
sDP4+ (C data)	97.18%	2.82%	-	-	-	-
sDP4+ (all data)	97.18%	2.82%	-	-	-	-
uDP4+ (H data)	-	-	-	-	-	-
uDP4+ (C data)	56.00%	44.00%	-	-	-	-
uDP4+ (all data)	56.00%	44.00%	-	-	-	-
DP4+ (H data)	-	-	-	-	-	-
DP4+ (C data)	97.77%	2.23%	-	-	-	-
DP4+ (all data)	97.77%	2.23%	-	-	-	-

Experimental represented the experimental data of major. **Isomer 1** represented the calculated **5a**; **Isomer 2** represented the calculated **5b**.

Table S29. DP4+ analysis of compound **5** (minor).

Functional		Solvent?		Basis Set		Type of Data	
B3LYP		PCM		6-31G(d)		Shielding Tensors	
		DP4+	0.29%	99.71%	-	-	-
Nuclei	sp2?	Experimental	Isomer 1	Isomer 2	Isomer 3	Isomer 4	Isomer 5
C	x	128.3	121.5	121.6			
C	x	134.4	127.0	125.8			
C	x	114.9	108.9	107.8			
C	x	142.5	133.3	133.9			
C	x	122.7	114.9	115.7			
C	x	109.6	99.1	98.5			
C	x	160.3	152.0	152.0			
C	x	94.8	89.4	88.1			
C	x	138.2	130.0	130.9			
C	x	141.8	135.7	135.2			
C	x	113.4	107.7	107.4			
C	x	166.2	157.86	156.31			
C	x	120	112.02	113.98			
C		55.3	49.12	48.94			
C		60.7	58.57	59.24			
C		31.3	26.15	29.39			
C		21.6	22.39	18.13			
C		47.8	46.49	45.04			
C	x	140.4	140.40	138.35			
C		19.9	22.62	22.21			
C		26.5	15.08	15.39			
C	x	172.9	168.22	168.07			
C		51.4	47.03	47.03			

Functional	Solvent?		Basis Set		Type of Data	
B3LYP	PCM		6-31G(d)		Shielding Tensors	
	Isomer 1	Isomer 2	Isomer 3	Isomer 4	Isomer 5	Isomer 6
sDP4+ (H data)	-	-	-	-	-	-
sDP4+ (C data)	0.23%	99.77%	-	-	-	-
sDP4+ (all data)	0.23%	99.77%	-	-	-	-
uDP4+ (H data)	-	-	-	-	-	-
uDP4+ (C data)	55.66%	44.34%	-	-	-	-
uDP4+ (all data)	55.66%	44.34%	-	-	-	-
DP4+ (H data)	-	-	-	-	-	-
DP4+ (C data)	0.29%	99.71%	-	-	-	-
DP4+ (all data)	0.29%	99.71%	-	-	-	-

Experimental represented the experimental data of minor. **Isomer 1** represented the calculated **5a**; **Isomer 2** represented the calculated **5b**.

Table S30. DP4+ analysis of compound **9** (major).

Functional		Solvent?		Basis Set		Type of Data	
B3LYP		PCM		6-31G(d)		Shielding Tensors	
		DP4+	0.03%	99.97%	–	–	–
Nuclei	sp2?	Experimental	Isomer 1	Isomer 2	Isomer 3	Isomer 4	Isomer 5
C		18.55	18.9	18.6			
C		20.22	20.0	20.2			
C		23.38	25.9	24.3			
C		25.53	27.3	26.0			
C		26.01	28.1	26.5			
C		32.6	28.5	34.4			
C		43.92	45.0	44.2			
C		49.85	52.4	50.0			
C		56.12	53.3	53.3			
C		64.09	63.9	67.4			
C	x	94.61	89.2	90.0			
C	x	111.3	104.31	103.23			
C	x	114.42	110.13	110.33			
C	x	116.16	112.87	111.89			
C	x	121.9	117.51	117.69			
C	x	122.81	119.00	118.65			
C	x	123.68	119.77	119.18			
C	x	131.73	124.74	124.54			
C	x	136.01	131.16	131.23			
C	x	136.24	131.75	131.80			
C	x	140.58	134.35	134.38			
C	x	142.53	135.56	134.76			
C	x	143.76	137.15	136.92			
C	x	145.36	139.21	140.49			
C	x	162.98	153.39	153.28			
C	x	170.11	160.196658	161.802682			
C	x	176.45	163.772604	166.97074			

Functional	Solvent?		Basis Set		Type of Data	
B3LYP	PCM		6-31G(d)		Shielding Tensors	
	Isomer 1	Isomer 2	Isomer 3	Isomer 4	Isomer 5	Isomer 6
sDP4+ (H data)	–	–	–	–	–	–
sDP4+ (C data)	0.06%	99.94%	–	–	–	–
sDP4+ (all data)	0.06%	99.94%	–	–	–	–
uDP4+ (H data)	–	–	–	–	–	–
uDP4+ (C data)	31.98%	68.02%	–	–	–	–
uDP4+ (all data)	31.98%	68.02%	–	–	–	–
DP4+ (H data)	–	–	–	–	–	–
DP4+ (C data)	0.03%	99.97%	–	–	–	–
DP4+ (all data)	0.03%	99.97%	–	–	–	–

Experimental represented the experimental data of major. **Isomer 1** represented the calculated **9a**; **Isomer 2** represented the calculated **9b**.

Table S31. DP4+ analysis of compound **9** (minor).

Functional		Solvent?		Basis Set		Type of Data	
B3LYP		PCM		6-31G(d)		Shielding Tensors	
		DP4+	99.28%	0.72%	-	-	-
Nuclei	sp2?	Experimenta	Isomer 1	Isomer 2	Isomer 3	Isomer 4	Isomer 5
C		18.55	18.9	18.6			
C		20.22	20.0	20.2			
C		26.45	25.9	24.3			
C		26.11	27.3	26.0			
C		26.41	28.1	26.5			
C		30.41	28.5	34.4			
C		44.11	45.0	44.2			
C		51.56	52.4	50.0			
C		56.12	53.3	53.3			
C		61.35	63.9	67.4			
C	x	94.61	89.2	90.0			
C	x	111.22	104.31	103.23			
C	x	114.53	110.13	110.33			
C	x	116.3	112.87	111.89			
C	x	121.95	117.51	117.69			
C	x	123.21	119.00	118.65			
C	x	123.48	119.77	119.18			
C	x	131.26	124.74	124.54			
C	x	136.18	131.16	131.23			
C	x	136.32	131.75	131.80			
C	x	140.64	134.35	134.38			
C	x	142.1	135.56	134.76			
C	x	143.43	137.15	136.92			
C	x	145.18	139.21	140.49			
C	x	162.89	153.39	153.28			
C	x	169.98	160.196658	161.802682			
C	x	172.11	163.772604	166.97074			

Functional	Solvent?		Basis Set		Type of Data	
B3LYP	PCM		6-31G(d)		Shielding Tensors	
	Isomer 1	Isomer 2	Isomer 3	Isomer 4	Isomer 5	Isomer 6
sDP4+ (H data)	-	-	-	-	-	-
sDP4+ (C data)	99.65%	0.35%	-	-	-	-
sDP4+ (all data)	99.65%	0.35%	-	-	-	-
uDP4+ (H data)	-	-	-	-	-	-
uDP4+ (C data)	32.59%	67.41%	-	-	-	-
uDP4+ (all data)	32.59%	67.41%	-	-	-	-
DP4+ (H data)	-	-	-	-	-	-
DP4+ (C data)	99.28%	0.72%	-	-	-	-
DP4+ (all data)	99.28%	0.72%	-	-	-	-

Experimental represented the experimental data of minor. **Isomer 1** represented the calculated **9a**; **Isomer 2** represented the calculated **9b**.

Table S32. DP4+ analysis of compound **10** (major).

Functional		Solvent?		Basis Set		Type of Data	
B3LYP		PCM		6-31G(d)		Shielding Tensors	
		DP4+	3.37%	96.63%	-	-	-
Nuclei	sp2?	Experimental	Isomer 1	Isomer 2	Isomer 3	Isomer 4	Isomer 5
C		18.55	18.7	18.1			
C		20	20.1	20.6			
C		22.89	26.0	23.9			
C		25.64	27.3	26.0			
C		26.01	27.9	26.5			
C		32.67	30.8	35.1			
C		43.9	45.1	43.8			
C		49.85	51.4	50.1			
C		52.67	51.7	51.6			
C		56.14	53.2	53.3			
C	x	63.29	63.8	64.1			
C	x	94.8	87.37	91.68			
C	x	111.34	106.38	102.09			
C	x	115.11	109.89	110.92			
C	x	116.4	111.22	111.48			
C	x	121.85	117.20	116.79			
C	x	123.43	119.05	117.64			
C	x	123.49	119.60	118.80			
C	x	131.54	124.72	125.26			
C	x	136.01	130.83	130.79			
C	x	136.53	132.02	132.01			
C	x	140.32	133.87	134.28			
C	x	141.56	136.44	136.24			
C	x	142.58	137.53	137.14			
C	x	145.27	138.81	140.19			
C	x	162.92	153.311455	152.937411			
C	x	168.98	158.264128	156.032983			
C	x	175.11	167.451205	168.038189			

Functional	Solvent?		Basis Set		Type of Data	
B3LYP	PCM		6-31G(d)		Shielding Tensors	
	Isomer 1	Isomer 2	Isomer 3	Isomer 4	Isomer 5	Isomer 6
sDP4+ (H data)	-	-	-	-	-	-
sDP4+ (C data)	23.33%	76.67%	-	-	-	-
sDP4+ (all data)	23.33%	76.67%	-	-	-	-
uDP4+ (H data)	-	-	-	-	-	-
uDP4+ (C data)	10.29%	89.71%	-	-	-	-
uDP4+ (all data)	10.29%	89.71%	-	-	-	-
DP4+ (H data)	-	-	-	-	-	-
DP4+ (C data)	3.37%	96.63%	-	-	-	-
DP4+ (all data)	3.37%	96.63%	-	-	-	-

Experimental represented the experimental data of major. **Isomer 1** represented the calculated **10a**; **Isomer 2** represented the calculated **10b**.

Table S33. DP4+ analysis of compound **10** (minor).

Functional		Solvent?		Basis Set		Type of Data	
B3LYP		PCM		6-31G(d)		Shielding Tensors	
		DP4+	99.61%	0.39%	-	-	-
Nuclei	sp2?	Experimenta	Isomer 1	Isomer 2	Isomer 3	Isomer 4	Isomer 5
C		18.57	18.7	18.1			
C		20.21	20.1	20.6			
C		26.37	26.0	23.9			
C		25.65	27.3	26.0			
C		26.13	27.9	26.5			
C		30.03	30.8	35.1			
C		44.13	45.1	43.8			
C		51.44	51.4	50.1			
C		52.76	51.7	51.6			
C		56.14	53.2	53.3			
C	x	61.63	63.8	64.1			
C	x	94.71	87.37	91.68			
C	x	111.41	106.38	102.09			
C	x	114.38	109.89	110.92			
C	x	116.38	111.22	111.48			
C	x	121.92	117.20	116.79			
C	x	123.57	119.05	117.64			
C	x	123.26	119.60	118.80			
C	x	131.48	124.72	125.26			
C	x	136.18	130.83	130.79			
C	x	136.53	132.02	132.01			
C	x	140.91	133.87	134.28			
C	x	142.17	136.44	136.24			
C	x	142.16	137.53	137.14			
C	x	145.25	138.81	140.19			
C	x	163	153.311455	152.937411			
C	x	168.98	158.264128	156.032983			
C	x	174.55	167.451205	168.038189			

Functional	Solvent?		Basis Set		Type of Data	
B3LYP	PCM		6-31G(d)		Shielding Tensors	
	Isomer 1	Isomer 2	Isomer 3	Isomer 4	Isomer 5	Isomer 6
sDP4+ (H data)	-	-	-	-	-	-
sDP4+ (C data)	99.95%	0.05%	-	-	-	-
sDP4+ (all data)	99.95%	0.05%	-	-	-	-
uDP4+ (H data)	-	-	-	-	-	-
uDP4+ (C data)	10.52%	89.48%	-	-	-	-
uDP4+ (all data)	10.52%	89.48%	-	-	-	-
DP4+ (H data)	-	-	-	-	-	-
DP4+ (C data)	99.61%	0.39%	-	-	-	-
DP4+ (all data)	99.61%	0.39%	-	-	-	-

Experimental represented the experimental data of minor. **Isomer 1** represented the calculated **10a**; **Isomer 2** represented the calculated **10b**.

Table S34. DFT-optimized structures for low-energy conformers of **4a**.

**Conformers**	**Conf. A**	**Conf. B**
DFT-optimized structures	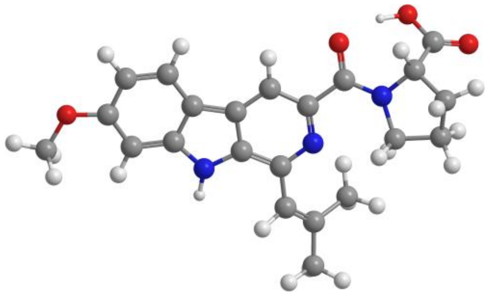	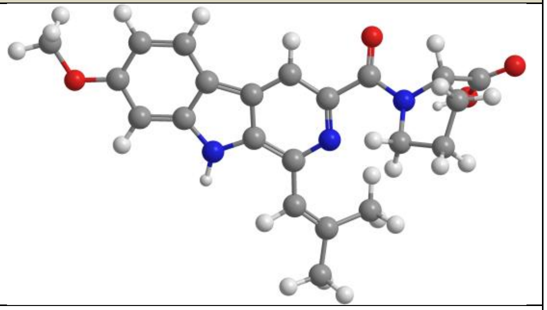
Population	61.81%	27.48%
**Conformers**	**Conf. C**	
DFT-optimized structures	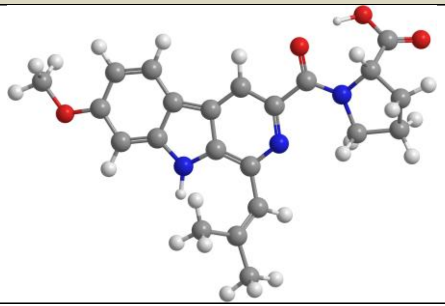	
Population	10.71%	

Table S35. DFT-optimized structures for low-energy conformers of **4b**.

**Conformers**	**Conf. A**	**Conf. B**
DFT-optimized structures	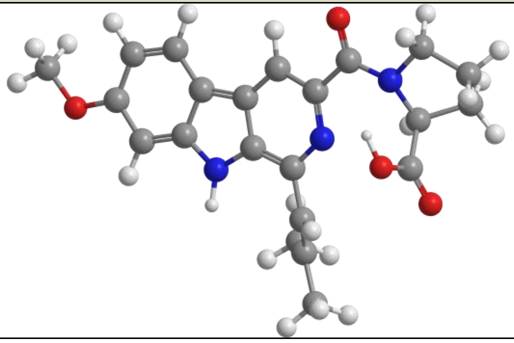	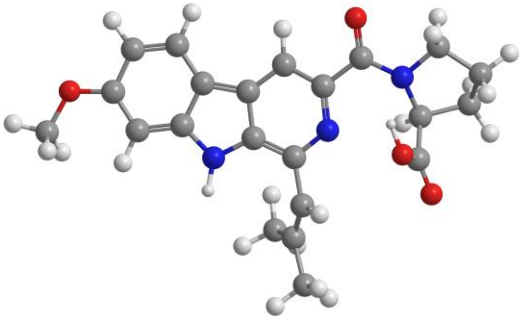
Population	56.71%	32.06%
**Conformers**	**Conf. C**	
DFT-optimized structures	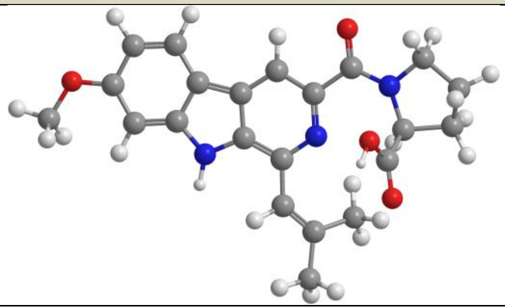	
Population	11.23%	

Table S36. DFT-optimized structures for low-energy conformers of **5a**.

**Conformers**	**Conf. A**	**Conf. B**
DFT-optimized structures	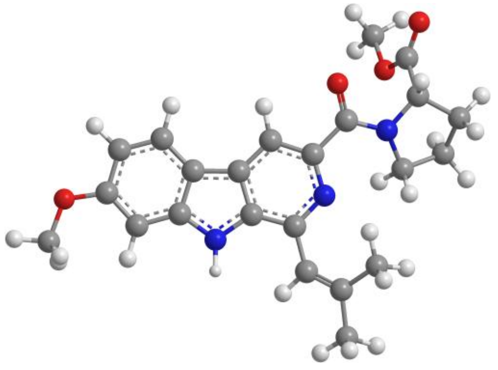	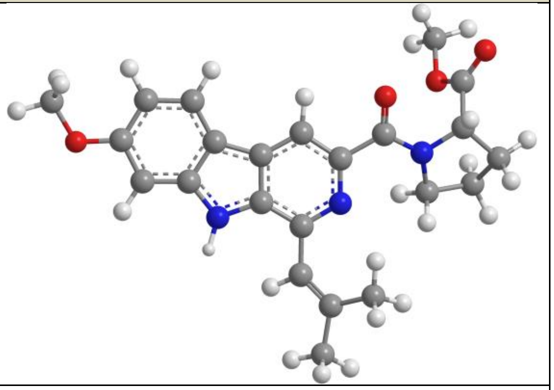
Population	42.13%	15.74%
**Conformers**	**Conf. C**	
DFT-optimized structures	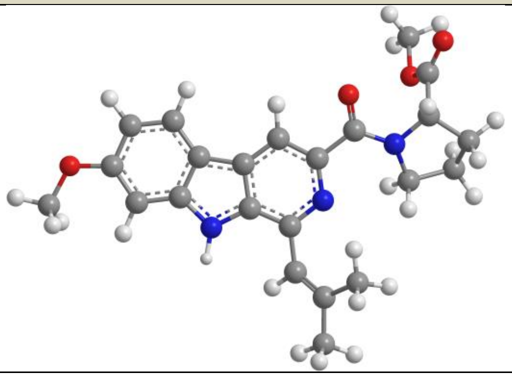	
Population	42.13%	

Table S37. DFT-optimized structures for low-energy conformers of **5b**.

**Conformers**	**Conf. A**	**Conf. B**
DFT-optimized structures	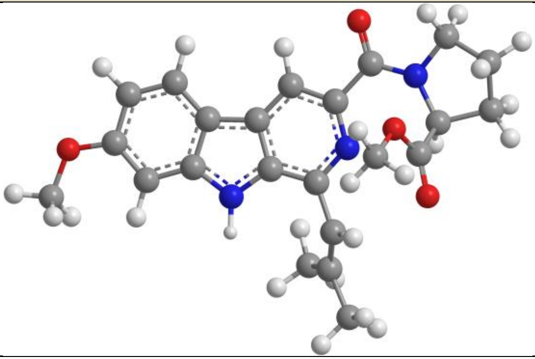	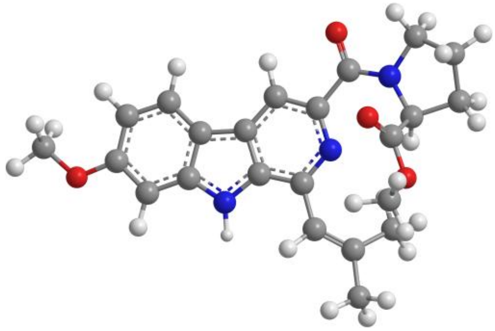
Population	15.81%	16.04%
**Conformers**	**Conf. C**	**Conf. D**
DFT-optimized structures	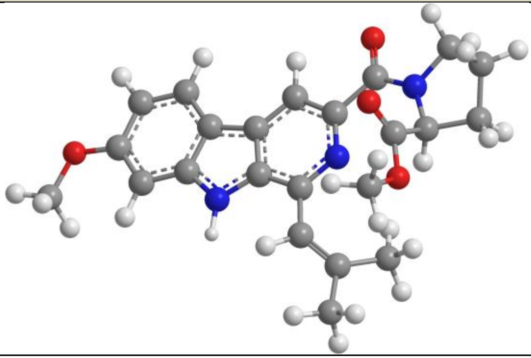	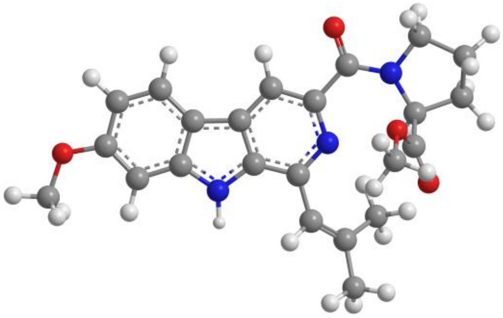
Population	42.39%	25.77%

Table S38. DFT-optimized structures for low-energy conformers of **9a**.

**Conformers**	**Conf. A**	**Conf. B**
DFT-optimized structures	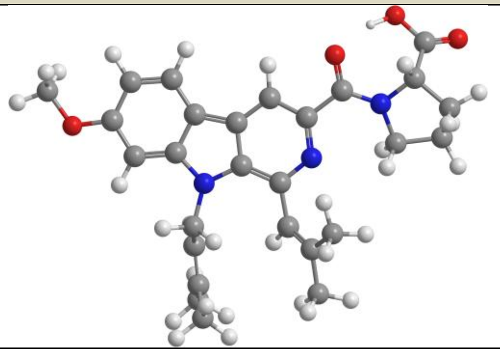	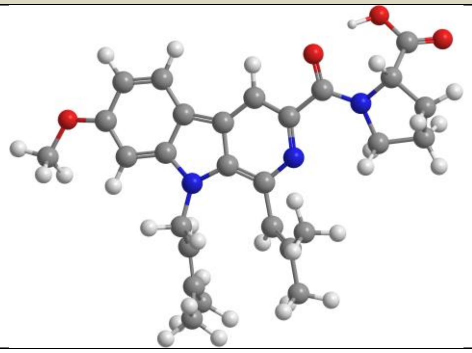
Population	62.70%	24.78%
**Conformers**	**Conf. C**	
DFT-optimized structures	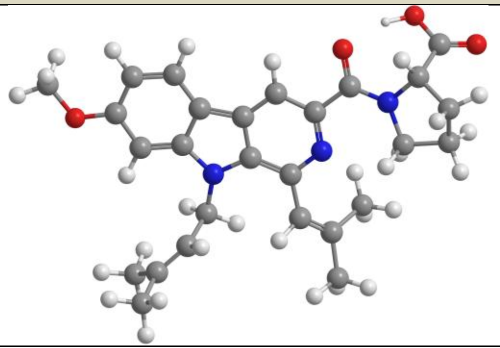	
Population	12.52%	

Table S39. DFT-optimized structures for low-energy conformers of **9b**.

**Conformers**	**Conf. A**	**Conf. B**
DFT-optimized structures	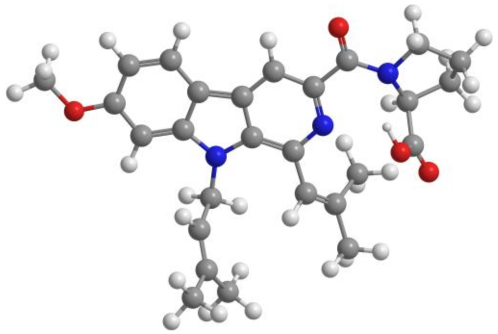	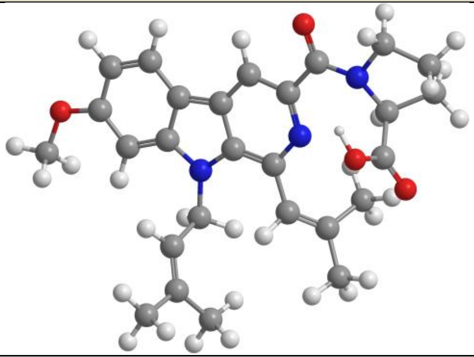
Population	47.85%	34.09%
**Conformers**	**Conf. C**	
DFT-optimized structures	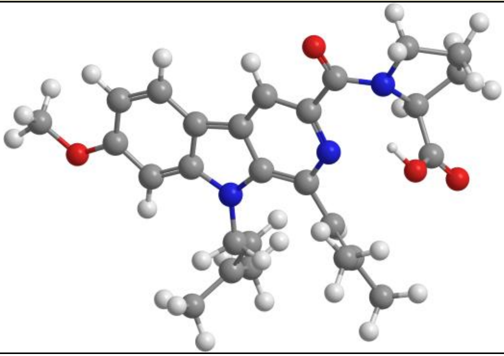	
Population	18.06%	

Table S40. DFT-optimized structures for low-energy conformers of **10a**.

**Conformers**	**Conf. A**	**Conf. B**
DFT-optimized structures	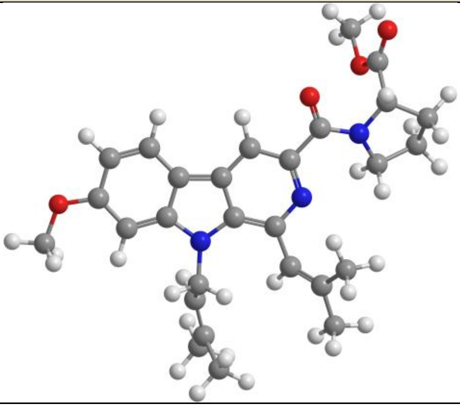	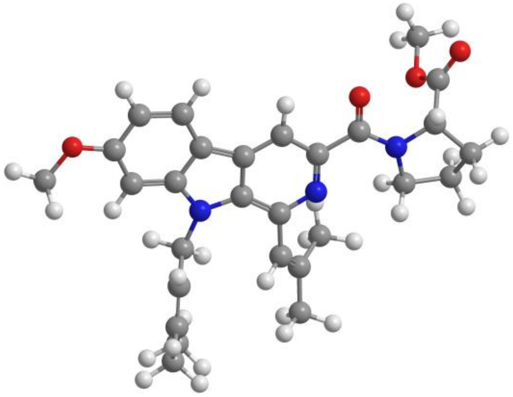
Population	48.33%	42.02%
**Conformers**	**Conf. C**	
DFT-optimized structures	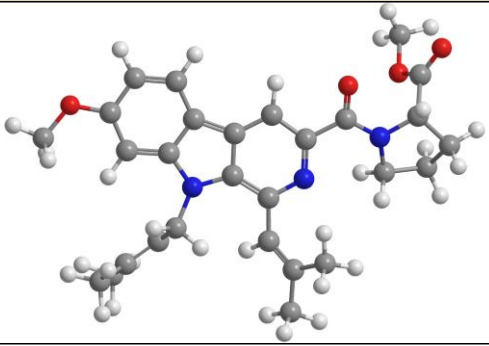	
Population	9.65%	

Table S41. DFT-optimized structures for low-energy conformers of **10b**.

**Conformers**	**Conf. A**	**Conf. B**
DFT-optimized structures	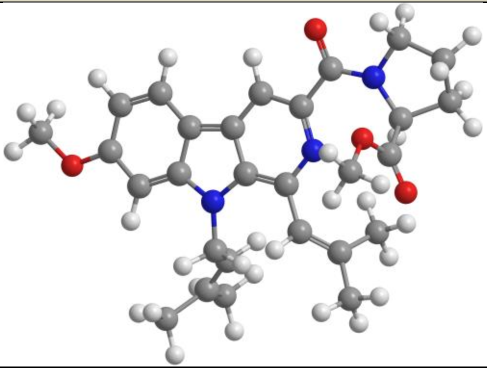	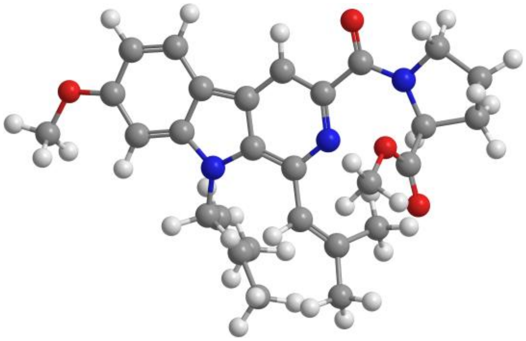
Population	52.63%	34.74%
**Conformers**	**Conf. C**	
DFT-optimized structures	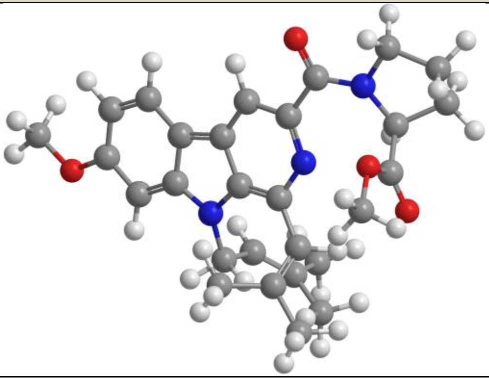	
Population	12.63%	

REFERENCES AND NOTES

1. P. F. Rosales, G. S. Bordin, A. E. Gower, S. Moura, Indole alkaloids: 2012 until now, highlighting the new chemical structures and biological activities. *Fitoterapia* **143**, 104558 (2020).

2. Y. M. Ma, X. A. Liang, Y. Kong, B. Jia, Structural diversity and biological activities of indole diketopiperazine alkaloids from fungi. *J. Agric. Food Chem.* **64**, 6659–6671 (2016).

3. S. Z. Zulkifli, N. H. Pungot, A. S. Saaidin, N. A. Jani, M. F. Mohammat, Synthesis and diverse biological activities of substituted indole β-carbolines: A review. *Nat. Prod. Res.* **38**, 3793–3806 (2024).

4. P. O. Venkataramana Reddy, M. Hridhay, K. Nikhil, S. Khan, P. N. Jha, K. Shah, D. Kumar, Synthesis and investigations into the anticancer and antibacterial activity studies of β-carboline chalcones and their bromide salts. *Bioorg. Med. Chem. Lett.* **28**, 1278–1282 (2018).

5. Y. Yang, Y. Huang, H. Song, Y. Liu, L. Wang, Q. Wang, Skeletal modifications of β-carboline alkaloids and their antiviral activity profile. *Mol. Divers.* **20**, 829–835 (2016).

6. G. M. Olmedo, L. Cerioni, M. M. González, F. M. Cabrerizo, V. A. Rapisarda, S. I. Volentini, Antifungal activity of β-carbolines on Penicillium digitatum and Botrytis cinerea. *Food Microbiol.* **62**, 9–14 (2017).

7. P. Piechowska, R. Zawirska-Wojtasiak, S. Mildner-Szkudlarz, Bioactive β-carbolines in food: A review. *Nutrients* **11**, 814 (2019).

8. C. Cui, H. Kakeya, H. Osada, Novel mammalian cell cycle inhibitors, spirotryprostatins A and B, produced by *Aspergillus fumigatus*, which inhibit mammalian cell cycle at G2/M phase. *Tetrahedron* **52**, 12651–12666 (1996).

9. Y. Ma, C. Fan, B. Jia, P. Cheng, J. Liu, Y. Ma, K. Qiao, Total synthesis and biological evaluation of spirotryprostatin A analogs. *Chirality* **29**, 737–746 (2017).

10. C. B. Cui, H. Kakeya, H. Osada, Spirotryprostatin B, a novel mammalian cell cycle inhibitor produced by Aspergillus fumigatus. *J. Antibiot.* **49**, 832–835 (1996).

11. J. J. Maresh, L.-A. Giddings, A. Friedrich, E. A. Loris, S. Panjikar, B. L. Trout, J. Stöckigt, B. Peters, S. E. O'Connor, Strictosidine synthase: Mechanism of a Pictet−Spengler catalyzing enzyme. *J. Am. Chem. Soc.* **130**, 710–723 (2008).

12. T. M. Kutchan, Strictosidine: From alkaloid to enzyme to gene. *Phytochemistry* **32**, 493–506 (1993).

13. J. Stöckigt, Enzymatic formation of intermediates in the biosynthests of ajmalicine: Strictosidine and cathenamine. *Phytochemistry* **18**, 965–971 (1979).

14. J. Stöckigt, A. P. Antonchick, F. Wu, H. Waldmann, Die Pictet-Spengler-Reaktion in der Natur und der organischen Chemie. *Angew. Chem.* **123**, 8692–8719 (2011).

15. J. Stöckigt, M. H. Zenk, Strictosidine (isovincoside): The key intermediate in the biosynthesis of monoterpenoid indole alkaloids. *J. Chem. Soc. Chem. Commun.* , 646–648 (1977).

16. J. Stöckigt, M. H. Zenk, Isovincoside (strictosidine), the key intermediate in the enzymatic formation of indole alkaloids. *FEBS Lett.* **79**, 233–237 (1977).

17. N. Cao, C.-H. Wang, Strictosidine synthase, an indispensable enzyme involved in the biosynthesis of terpenoid indole and β-carboline alkaloids. *Chin. J. Nat. Med.* **19**, 591–607 (2021).

18. Q. Chen, C. Ji, Y. Song, H. Huang, J. Ma, X. Tian, J. Ju, Discovery of McbB, an enzyme catalyzing the β-carboline skeleton construction in the marinacarboline biosynthetic pathway. *Angew. Chem. Int. Ed. Engl.* **52**, 9980–9984 (2013).

19. S. Ueda, H. Ikeda, T. Namba, Y. Ikejiri, Y. Nishimoto, M. Arai, T. Nihira, S. Kitani, Identification of biosynthetic genes for the β-carboline alkaloid kitasetaline and production of the fluorinated derivatives by heterologous expression. *J. Ind. Microbiol. Biotechnol.* **46**, 739–750 (2019).

20. Z. Zheng, H. Choi, H.-w. Liu, In vitro characterization of kitasetaline biosynthesis reveals a bifunctional P450 decarboxylase and a vinyl β-carboline intermediate susceptible to nonenzymatic thiol addition. *J. Am. Chem. Soc.* **146**, 28553–28560 (2024).

21. Y. Tsunematsu, N. Ishikawa, D. Wakana, Y. Goda, H. Noguchi, H. Moriya, K. Hotta, K. Watanabe, Distinct mechanisms for spiro-carbon formation reveal biosynthetic pathway crosstalk. *Nat. Chem. Biol.* **9**, 818–825 (2013).

22. L.-H. Yan, X.-M. Li, L.-P. Chi, X. Li, B.-G. Wang, Six new antimicrobial metabolites from the deep-sea sediment-derived fungus *Aspergillus fumigatus* SD-406. *Mar. Drugs* **20**, 4 (2022).

23. A. Grundmann, T. Kuznetsova, S. S. Afiyatullov, S.-M. Li, FtmPT2, an *N*-prenyltransferase from *Aspergillus fumigatus*, catalyses the last step in the biosynthesis of fumitremorgin B. *Chembiochem* **9**, 2059–2063 (2008).

24. G.-Z. Qiu, M.-Z. Jin, J.-X. Dai, W. Sun, J.-H. Feng, W.-L. Jin, Reprogramming of the tumor in the hypoxic niche: The emerging concept and associated therapeutic strategies. *Trends Pharmacol. Sci.* **38**, 669–686 (2017).

25. Y. A. Ragulin, O. S. Izmestieva, V. V. Vapnyar, N. V. Severskaya, M. V. Poluektova, S. E. Glebova, I. L. Ershova, V. N. Derbugov, L. P. Zhavoronkov, Erythropoiesis reaction to tumor growth in lung cancer patients. *Sib. J. Oncol.* **16**, 53–58 (2017).

26. G. Jiménez-Valerio, O. Casanovas, Antiangiogenic resistance: Novel angiogenesis axes uncovered by antiangiogenic therapies research. *Curr. Drug Targets* **17**, 1728–1734 (2016).

27. P. J. Kallio, I. Pongratz, K. Gradin, J. McGuire, L. Poellinger, Activation of hypoxia-inducible factor 1α: Posttranscriptional regulation and conformational change by recruitment of the Arnt transcription factor. *Proc. Natl. Acad. Sci. U.S.A.* **94**, 5667–5672 (1997).

28. S. Salceda, J. Caro, Hypoxia-inducible factor 1α (HIF-1α) protein is rapidly degraded by the ubiquitin-proteasome system under normoxic conditions: Its stabilization by hypoxia depends on redox-induced changes. *J. Biol. Chem.* **272**, 22642–22647 (1997).

29. G. N. Masoud, W. Li, HIF-1α pathway: Role, regulation and intervention for cancer therapy. *Acta Pharm. Sin. B* **5**, 378–389 (2015).

30. J. J. Valdes, J. E. Cameron, R. J. Cole, Aflatrem: A tremorgenic mycotoxin with acute neurotoxic effects. *Environ. Health Perspect.* **62**, 459–463 (1985).

31. S. K. Rabindran, D. D. Ross, L. A. Doyle, W. Yang, L. M. Greenberger, Fumitremorgin C reverses multidrug resistance in cells transfected with the breast cancer resistance protein. *Cancer Res.* **60**, 47–50 (2000).

32. Y. Xi, H. Zhang, R. X. Li, S. Y. Kang, J. Li, Y. Li, Total synthesis of spirotryprostatins through organomediated intramolecular umpolung cyclization. *Chemistry* **25**, 3005–3009 (2019).

33. H. Tominaga, M. Ishiyama, F. Ohseto, K. Sasamoto, T. Hamamoto, K. Suzuki, M. Watanabe, A water-soluble tetrazolium salt useful for colorimetric cell viability assay. *Anal. Commun.* **36**, 47–50 (1999).

34. X. Liu, W. Wei, C. Wang, H. Yue, D. Ma, C. Zhu, G. Ma, Y. Du, Apoferritin-camouflaged Pt nanoparticles: Surface effects on cellular uptake and cytotoxicity. *J. Mater. Chem.* **21**, 7105–7110 (2011).

35. S.-Z. Lin, W.-T. Wei, H. Chen, K.-J. Chen, H.-F. Tong, Z.-H. Wang, Z.-L. Ni, H.-B. Liu, H.-C. Guo, D.-L. Liu, Antitumor activity of emodin against pancreatic cancer depends on its dual role: Promotion of apoptosis and suppression of angiogenesis. *PLOS ONE* **7**, e42146 (2012).

36. X. Li, S.-L. Zheng, X. Li, J.-L. Li, O. Qiang, R. Liu, L. He, Synthesis and anti-breast cancer activity of new indolylquinone derivatives. *Eur. J. Med. Chem.* **54**, 42–48 (2012).

37. M.-Y. Xu, S. Y. Lee, S. S. Kang, Y. S. Kim, Antitumor activity of jujuboside B and the underlying mechanism via induction of apoptosis and autophagy. *J. Nat. Prod.* **77**, 370–376 (2014).

38. J.-T. Wu, S.-M. Lv, C.-H. Lu, J. Gong, J.-B. An, Effect of 3,3′-biisofraxidin on apoptosis of human gastric cancer BGC-823 cells. *Trop. J. Pharm. Res.* **14**, 1803–1811 (2015).

39. Y. Wang, Z.-L. Li, J. Bai, L.-M. Zhang, X. Wu, L. Zhang, Y.-H. Pei, Y.-K. Jing, H.-M. Hua, 2,5-diketopiperazines from the marine-derived fungus *Aspergillus fumigatus* YK-7. *Chem. Biodivers.* **9**, 385–393 (2012).
